# Catalytically Promiscuous PLP-Dependent Aminotransferases Are Biocatalysts for C–C Bond Formation

**DOI:** 10.1021/acscentsci.6c00531

**Published:** 2026-06-29

**Authors:** Alexander T. Kim, James R. Howard, Andrés G. Cuba Cáceres, Kyle I. Chong, William A. Aye, Matthew S. Sigman, Alison R. H. Narayan

**Affiliations:** †Life Sciences Institute, ‡Program in Chemical Biology, §Department of Chemistry, University of Michigan, Ann Arbor, Michigan 48109, United States; ∥ Department of Chemistry, 7060 University of Utah, Salt Lake City, Utah 84112, United States

## Abstract

Access to noncanonical amino acids is increasingly important in natural products synthesis and drug discovery. Enzymatic synthesis has emerged as an efficient strategy for simplifying approaches toward these molecules. Pyridoxal-5′-phosphate (PLP)-dependent enzymes have been extensively studied for the biocatalytic functionalization of α-amino acids. However, the high substrate specificity of these enzymes has limited the substrate scope of PLP-mediated biocatalysis. Efforts to identify generalist catalysts may be enabled by catalytic promiscuity, a property well-known in the PLP-dependent enzyme family. In pursuit of PLP-catalyzed C–C bond formation, we leverage a cofactor-centric approach with PLP-dependent enzymes not previously known to mediate C–C bond formation to access key nucleophilic intermediates. In this work, we show that aminotransferases Aro8 and TyrB can mediate C–C bond formation, the first such activity reported for aminotransferases. Further, this non-native function can be improved by the addition of a sacrificial ketoacid. Interestingly, Aro8 and TyrB exhibit distinct conformational dynamics that impact the diastereoselectivity of the reaction. These results indicate that aminotransferases are an untapped resource for the discovery of new biocatalysts for C–C bond formation and demonstrate the value of cofactor-guided reaction discovery.

## Graphical abstract

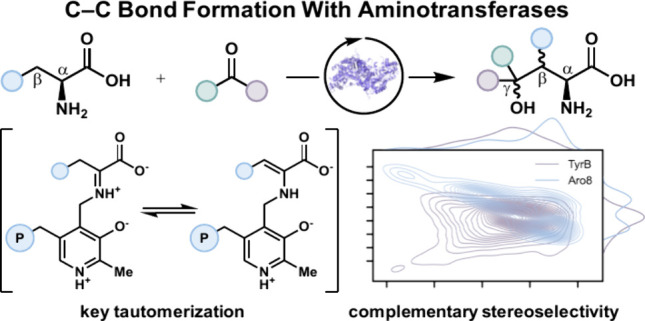

## Introduction

Amino acids are increasingly important building blocks for drug discovery, with a premium on noncanonical amino acids that can be accessed through de novo synthesis or modification of available amino acids.^1^ For example, the antifungal natural products polyoxin D and nikkomycin Z contain γ-hydroxy-α-amino acid moieties that are critical for binding to their target, chitin synthetase (Figure 1a).^2–4^ The amino acid side chain of these molecules presents a significant synthetic challenge; the most efficient total synthesis of nikkomycin Z constructs the γ-hydroxy-α-amino acid motif in eight steps, which includes protecting group manipulations.^5^ The interest in noncanonical amino acids has resulted in the development of a suite of methods to build and elaborate amino acids as well as strategies to tame the defining functional groups of these compounds.^6–8^ Hydroxy amino acids are typically accessed through acylation reactions,^9^ [3 + 2] cycloadditions strategies involving nitrones,^10,11^ conjugate addition into acrylic acids,^12^ and Mannich reactions.^13^ However, these strategies often require carefully designed protecting group choreography to contend with the amino and carboxylate groups. In contrast, nature has evolved enzymes that allow for the modification of amino acid substrates in a site-, chemo-, and stereoselective manner.^14^ Harnessing these enzymes for synthetic chemistry can sidestep the need for protecting group manipulations. For example, stereoselective hydroxylation and halogenation with nonheme iron-dependent enzymes,^15–17^ C–C bond formation with tryptophan synthases,^18,19^ and alkylation with SAM-dependent methyltransferases^20,21^ have been extensively investigated. One versatile enzyme family for amino acid synthesis and functionalization is the pyridoxal-5′-phosphate (PLP)-dependent enzymes.^22^

**Figure 1:**
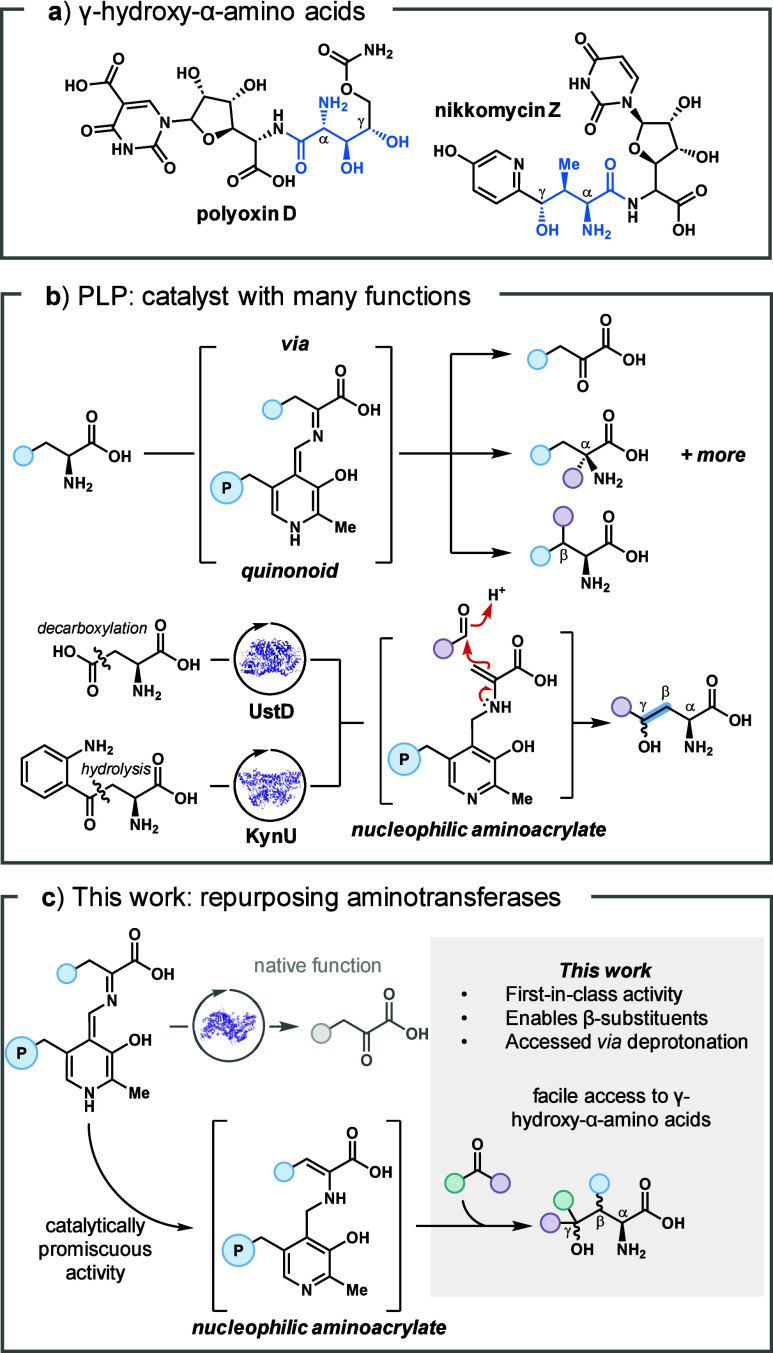
PLP-dependent enzymes and catalytic promiscuity. (a) Examples of γ-hydroxy-α-amino acids in natural products. (b) Key reactive intermediates for PLP-dependent enzymes. (c) Cofactor-centric reaction discovery uncovers previously unknown C–C bond formation catalyzed by PLP-dependent aminotransferases.

PLP-dependent enzymes are integral in both primary and secondary metabolism, including the synthesis of amino acids.^23,24^ These enzymes most commonly catalyze functional group interconversion; however, this enzyme family at large possesses an enormous range of chemistries that allow for the modification of amino acid substrates (Figure 1b). Amino acids can be transformed by PLP-dependent enzymes through decarboxylation, transamination, racemization, aldol and retro-aldol, and Claisen condensation reactions, among others,^25,26^ leading to the development of biocatalytic syntheses of noncanonical amino acids.^27^

Of particular interest are PLP-dependent enzymes that catalyze C–C bond formation, which provide an opportunity to build onto the carbon skeleton of amino acids, enabling access to more complex scaffolds. The two most common sites of C–C bond formation are the α- and β-positions (Figure 1b). Functionalization of the α-carbon is archetypal of threonine aldolases^28^ and serine hydroxy­methyl­trans­ferases.^29^ These enzymes have been used to synthesize β-hydroxy-α-amino acids, enabling biocatalytic access to chloramphenicol and related molecules.^30,31^ Recent work by Hyster^32^ and Yang^33^ have extended the reactivity of threonine aldolases to radical-based α-alkylation, enabling the photobiocatalytic synthesis of tetrasubstituted α-stereocenters. β-substitution, in contrast, can be accomplished through either electrophilic or nucleophilic mechanisms. Electrophilic β-substitution is best exemplified by the native reaction of tryptophan synthase. An initial dehydration of serine leads to an electrophilic aminoacrylate species to which nucleophiles, such as indoles, can add to produce tryptophan or derivatives thereof.^34,100^ Alternatively, nucleophilic β-substitution of amino acids can be mediated by enzymes UstD and KynU. Both UstD and KynU are proposed to mediate this chemistry by accessing a nucleophilic aminoacrylate intermediate following decarboxylation of aspartate^35^ or hydrolysis of kynurenine, respectively (Figure 1b).^36^ The nucleophilic aminoacrylate can then engage with carbonyl electrophiles to form γ-hydroxy-α-amino acid products.^37–39^ However, enzymatic C–C bond formation at either the α- or β-position is somewhat limited with regards to the amino acid substrate scope. For example, the requirement to first cleave a C–C bond to access the operative nucleophilic aminoacrylate for β-substitution reactions prevents the use of substrates that have a substituent at the β-carbon. Use of threonine aldolases, meanwhile, remains limited to short, alkyl amino acids like glycine or l-alanine.^40^ Thus, the identification of enzymes and new mechanisms that can lead to α- and β-functionalization of amino acid scaffolds remains valuable. More specifically, discovering new paths toward nucleophilic intermediates in PLP-mediated reactions could provide direct access to a broader set of noncanonical amino acid building blocks.

PLP-dependent enzymes are well-suited for this paradigm of mechanism-based reaction discovery due to catalytic promiscuity—the ability for a single enzyme to access different intermediates and catalyze mechanistically distinct reactions.^41^ This is illustrated by the diverse chemistry that is accessible from common intermediates in the PLP mechanism, such as the quinonoid intermediate (Figure 1b). The fate of the quinonoid generally dictates the type of reaction the enzyme catalyzes. For example, quinonoid intermediates can directly engage with electrophiles leading to bond formation at the α-position, as with threonine aldolases, or eliminate the β-hydroxy group from serine, as with tryptophan synthases. In addition, there are examples of the quinonoid intermediate being diverted to access non-native transformations. Some examples include an alanine racemase that can catalyze both racemization and retro-aldol chemistry,^42,43^ an aspartate aminotransferase that can mediate decarboxylation reactions,^44^ and aminotransferase CVTA which can carry out C–F bond cleavage.^45^ Thus far, investigations into C–C bond formation with PLP-dependent enzymes have focused on threonine aldolase-like chemistry, i.e., α-functionalization. Few examples of catalytically promiscuous PLP-dependent enzymes functionalizing more remote carbons have been reported.^46–103^ Moreover, while aminotransferases are widely known to be catalytically promiscuous, C–C bond formation with this class remains elusive. Thus, the extent to which PLP-dependent enzymes can access non-native intermediates remains under investigation.

We posit that PLP-dependent enzymes could provide a playground for development of C–C bond forming reactions based on the rich history of their catalytic promiscuity. In principle, a PLP-dependent enzyme that can access the quinonoid intermediate could be repurposed for C–C bond formation regardless of the annotated function of the enzyme. Consequently, the true diversity of PLP-dependent enzymes that are available to forge new C–C bonds is much larger than homology-based bioinformatic analyses may suggest. The use of catalytically promiscuous enzymes as catalysts is an underexplored strategy that complements previously reported methods using threonine aldolase, UstD, and KynU. Therefore, as a proof of concept, we sought to identify PLP-dependent enzymes without previously reported lyase activity that could nonetheless be competent catalysts for C–C bond formation. We turned our attention to PLP-dependent aminotransferases, as this class of enzyme readily accesses the quinonoid intermediate^47–49^ but to our knowledge has not been shown to catalyze C–C bond formation. We hypothesized that aminotransferases could provide the catalytic promiscuity necessary for mediating C–C bond formation chemistry by diverting the quinonoid toward nucleophilic intermediates that are off-pathway from transamination.

## Results and Discussion

### Discovery of Catalytically Promiscuous Aminotransferases

We sought to cast a wide net in the search for catalytically promiscuous aminotransferases. Previously, we described the construction of a functionally broad PLP-dependent enzyme library, which contained a subset of enzymes known to function as aminotransferases (53 enzymes from the 139-enzyme library).^50^ To test this panel of aminotransferases for C–C bond forming activity, a series of analytical reactions were conducted in 96-well plate format. Specifically, l-alanine (**1**) or l-homoalanine (**2**) were tested as the amino acid substrate in reactions with either isatin (**3**) or benzaldehyde (**4**) as electrophiles (Figure 2a). As is common in non-native reactions with PLP-dependent enzymes,^40,51^ a high concentration of substrate (∼100 mM amino acid, 50 mM electrophile) was used. Two aromatic aminotransferases, Aro8 and TyrB, most consistently generated measurable product, though only Aro8 was active across all substrate combinations. Aro8 was chosen as the model enzyme for subsequent experiments based on this demonstrated generality and higher activity compared to TyrB (∼2×).

**Figure 2:**
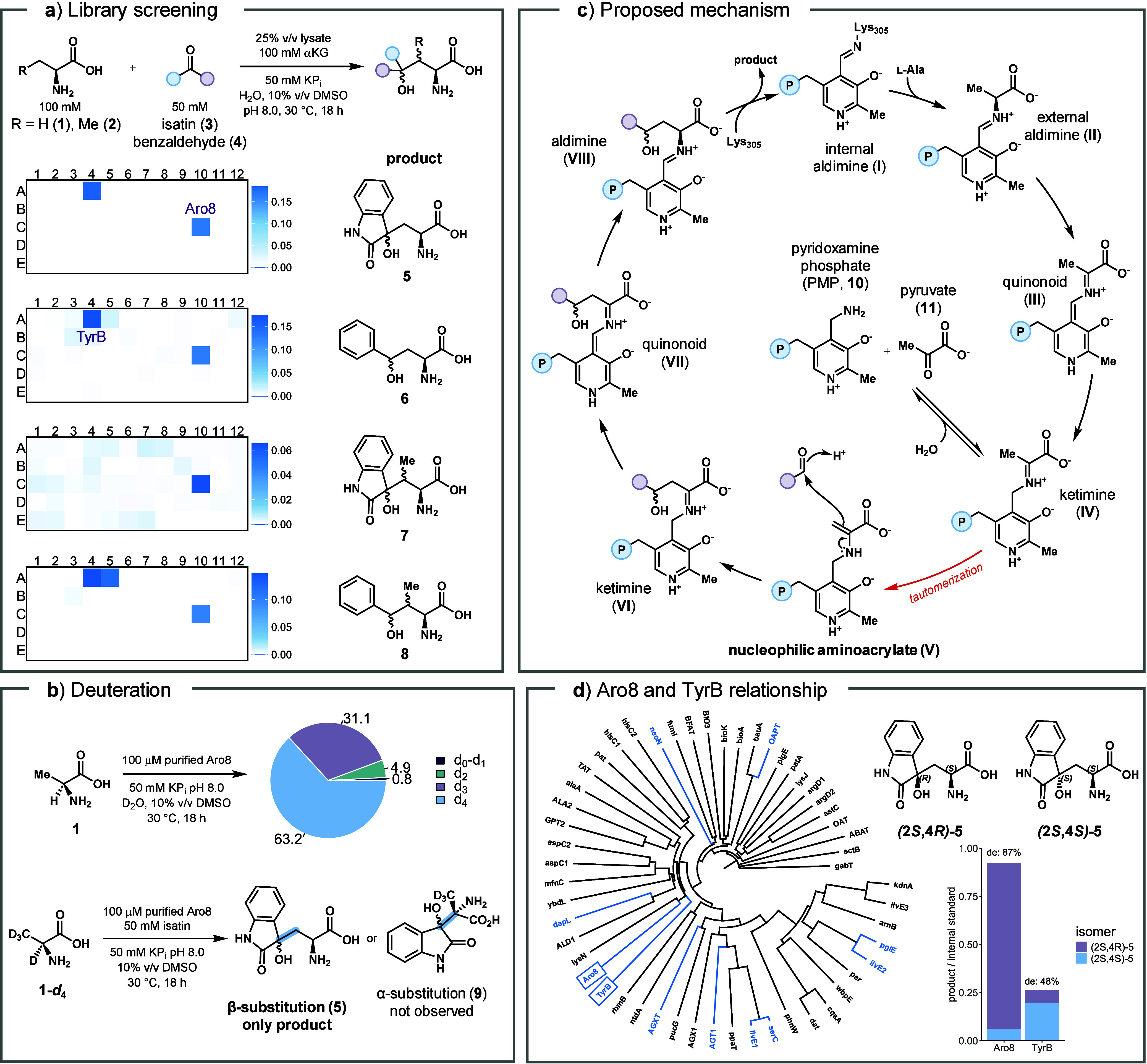
(a) Screening various amino acid and electrophile combinations across the library. Each cell represents a different enzyme, and the color scale indicates product formation (calculated as LC-MS product peak area divided by LC-MS peak area of l-tryptophan methyl ester internal standard) for **5**–**8**. (b) Deuteration experiments to determine site-selectivity. Distribution of deuterated materials was determined by LC-MS. (c) Proposed mechanism for PLP-dependent aminotransferase-catalyzed C–C bond formation. (d) Sequence identity across the aminotransferase library is displayed as a phylogenetic tree. Enzymes with >10% activity relative to Aro8 (as determined in panel (b)) are highlighted in blue. A bar chart shows that Aro8 and TyrB are stereodivergent and form complementary diastereomers of **5**. The diastereomeric excess (de) was determined by LC-MS.

To differentiate between α-substitution (threonine aldolase-like) and β-substitution (UstD/KynU-like) products, a series of deuteration experiments were performed. First, purified Aro8 was incubated with l-alanine (**1**) in deuterated buffer and end point H-D exchange was measured by LC-MS. We observed >90% incorporation of at least two deuterium atoms per molecule, indicating that Aro8 catalyzed H-D exchange not only at the expected α-position, but also at the β-position. In a second experiment, **1-** ***d***_**4**_ was incubated with purified Aro8 and isatin (**3**) in phosphate buffer. We anticipated that these conditions could lead to two possible products. If substitution occurred at the α-carbon, similar to threonine aldolase-like reactions, we would expect an [M + 3] mass (*m*/*z* 240, **9**) as the β-deuterium atoms would be retained. Instead, we only observed the parent mass (*m*/*z* 237, **5**), indicating that all deuterium atoms were exchanged over the course of the reaction and that the β-carbon is a potential site of reaction. Comparison to an authentic standard of **5** by LC-MS/MS supported this conclusion (Supporting Information, Figure S8). Based on these observations, we hypothesized that C–C bond formation occurs at the β-carbon, marking Aro8 and TyrB as mechanistically distinct from threonine aldolase and related enzymes. As such, we propose that C–C bond formation could be mediated through the mechanism shown in Figure 2c. As initial conversion of the internal aldimine **I** to ketimine **IV** is on-pathway for transamination, the native reaction of Aro8,^52^ these steps likely commence the catalytic cycle. From **IV**, subsequent hydrolysis to release pyridoxamine (PMP, **10**) and pyruvate (**11**) would complete the first half-reaction toward a transamination pathway. However, tautomerization of **IV** to the nucleophilic aminoacrylate **V** instead would enable an aldol-like reaction to form intermediate ketimine **VI**. A series of proton transfers, then transimination of aldimine **VIII** by an internal lysine, could complete the catalytic cycle and release the product, a γ-hydroxy-α-amino acid. Based on this discovery, we anticipated that Aro8 and TyrB could be useful catalysts to complement UstD and KynU, by providing complementary access to a nucleophilic aminoacrylate intermediate by deprotonation rather than C–C bond cleavage of a preactivated substrate. Indeed, tautomerization of **IV** was not observed with UstD,^37^ illustrating a potentially unique mode of substrate activation with aminotransferases. Both Aro8 and TyrB natively catalyze the reversible conversion of aromatic α-amino acids to α-keto acids.^52,53^ A multiple-sequence alignment of all the aminotransferases in the library was used to construct a phylogenetic tree, which illustrates the close relationship of Aro8 and TyrB (Figure 2d). Interestingly, product formation was observed in reactions with enzymes located across this phylogenetic tree, although at varying levels, suggesting that this non-native activity is widely distributed among aminotransferases, rather than limited to a single clade. Interestingly, comparison of the Aro8- and TyrB-catalyzed reactions showed that the two enzymes produced **5** with stereodivergence: Aro8 favors the (2*S*,4*R*)-diastereomer in 87% de, whereas TyrB favors the (2*S*,4*S*)-diastereomer in 48% de. The absolute configuration of the products was determined by comparison to synthetic standards using chiral LC-MS (Supporting Information, Figure S18). Consistent with many PLP-dependent enzymes, which are highly stereoselective at the α-carbon, both enzymes are highly enantioselective and set the α-stereocenter in the (*S*)-configuration in >99% ee.^54^

### Reaction Optimization

The yield of **5** under the initial conditions was low (3%, as determined by LC-MS comparison to a calibration curve of an authentic standard of **5**). We anticipated that the hydrolysis of **IV** was a competing process that could stall the reaction by sequestering the cofactor as PMP (**10**). Incubating Aro8 with l-alanine (**1**) led to the disappearance of a UV–vis peak at ∼350 nm and the appearance of a new peak at ∼330 nm, consistent with the hydrolysis of ketimine **VI** and formation of **10** (Figure 3a).^52^ Subsequent addition of **11** restores the 350 nm peak, indicating turnover of **10** back to the ketimine. This turnover is necessary for the enzyme to recycle PLP, which allows for C–C bond formation to take place. Indeed, in reactions with purified Aro8, addition of stoichiometric **11** improved the yield of the reaction 24-fold vs no additive (Figure 3b). Addition of exogenous **11** to drive the reaction forward precluded the use of l-homoalanine (**2**) as a substrate because **11** cannibalizes PMP to form ketimine **IV**, leading only to formation of **3** instead of the desired homoalanine-derived product. To circumvent this issue, we used an alternative α-ketoacid to reintroduce PMP into the catalytic cycle without further participation in the C–C bond forming step. Because α-ketoglutarate (αKG) is the native substrate for Aro8,^55^ we used two equivalents of αKG and observed a similar yield enhancement relative to **11** in reactions with both Aro8 and TyrB. Thus, αKG was chosen as a PMP recycling agent for subsequent experiments. While addition of αKG improved yield to 15%, increasing the concentration of l-alanine to 1 M improved the yield to 63%. The combination of both excess l-alanine and αKG improved the assay yield further to 93%.

**Figure 3:**
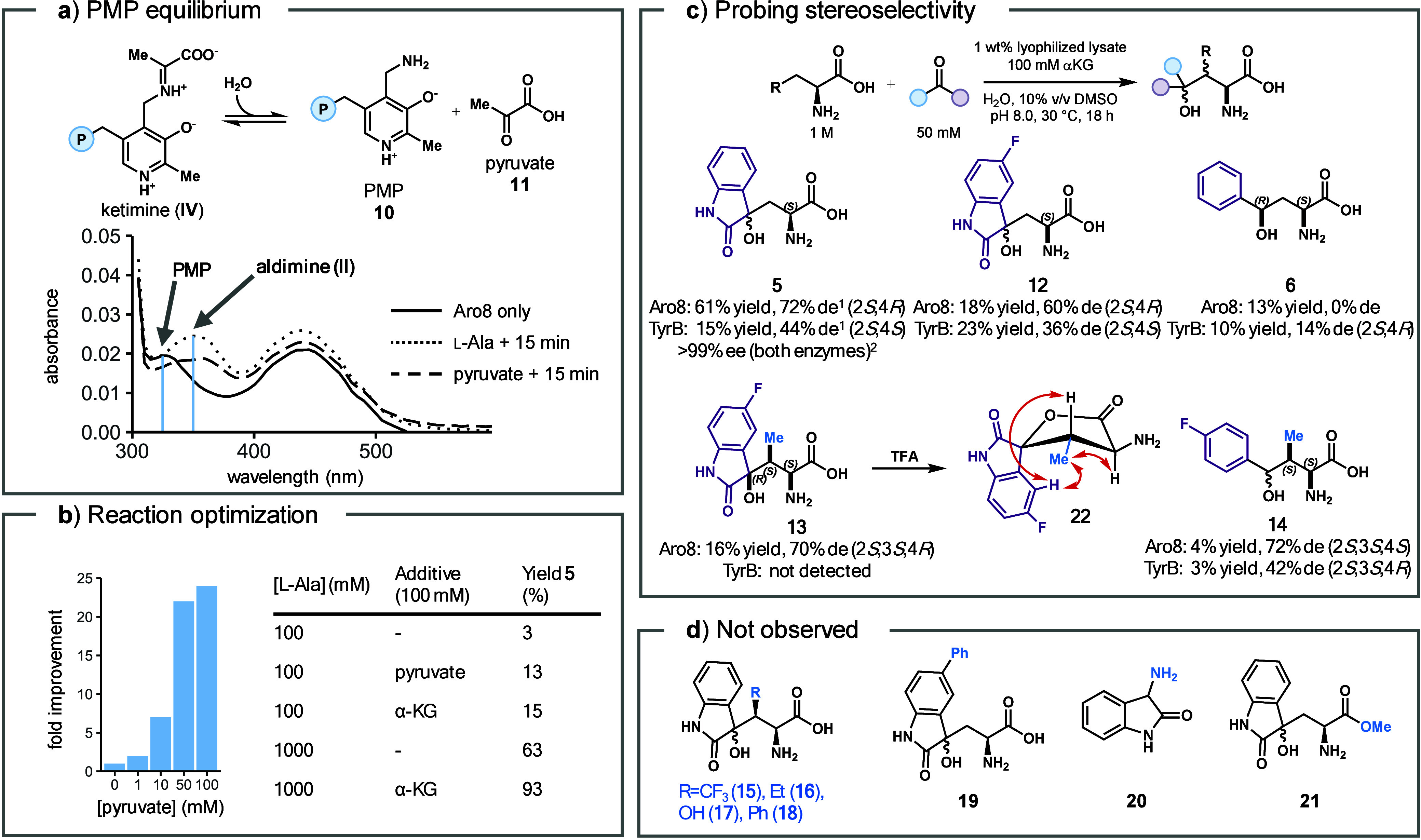
(a) Reversible conversion of aldimine **II** to PMP (**10**) is observable by UV–vis spectroscopy. Conditions: 15 μM purified Aro8, 2.5 mM l-alanine (**1**), 50 mM KP_i_, pH 8.0, 1 mL total volume. 2.5 μL of 1 M sodium pyruvate (2.5 mM final concentration) was added after 15 min. (b) Formation of **5** was tracked by LC-MS. Reaction conditions: 100 μM purified Aro8, 50 mM isatin (**3**), 50 mM KP_i_, pH 8.0, 30 °C, 18 h. Yield was determined by comparison to calibration curve on LC-MS. (c) Preparative-scale synthesis with Aro8 and TyrB. Yields are isolated yields; diastereoselectivity was determined by NMR spectroscopy. ^1^Determined by LC-MS (Supporting Information, Table S4). ^2^Determined by chiral LC-MS (Supporting Information, Figure S18). (d) Products that were not detected under reaction conditions.

### Investigation of Complementary Selectivity between Aro8 and TyrB

The stereoselectivity of the Aro8- and TyrB-catalyzed preparative-scale reactions was determined by NMR comparison to authentic standards (Supporting Information, Chemical Synthesis section). The reactions were performed with 1 wt % lyophilized lysate as the enzyme source, which is approximately 10–30 μM enzyme in the reaction (Supporting Information, Table S3). Lyophilized lysate allows the same batch of enzyme to be used over long periods of time and is commonly used in process chemistry.^56^ We compared the stereochemical outcome for a small panel of substrates (Figure 3c). Both enzymes produced **5** in modest to good yields (61% and 15% for Aro8/TyrB, respectively) but favored different diastereomers (72% de (2*S*,4*R*-**5**) and 44% de (2*S*,4*S*-**5**), respectively; Supporting Information, Table S4). Using benzaldehyde as the electrophile resulted in reduced yields (18% and 23% yield for Aro8/TyrB, respectively) of **6**. Interestingly, the Aro8-catalyzed reaction was nonselective and formed a 1:1 mixture of diastereomers, while the TyrB-catalyzed reaction was only slightly selective (14% de) for the (2*S*,4*R*)-diastereomer, a reversal of the selectivity with isatin (Supporting Information, Figure S25). We ascribe this difference to the additional amide functionality of isatin, which may hydrogen bond to nearby polar residues and bias the attack of a single prochiral face of the electrophilic carbon. We suspect that the lack of polar functionality and increased flexibility of benzaldehyde is responsible for the reduced diastereoselectivity. Fluorinated substrates were well-tolerated by both enzymes, and formation of **12** was similar to formation of **5** with both enzymes, albeit with reduced yield. 5-Fluoroisatin and *p*-fluorobenzaldehyde were used to characterize product distributions using ^19^F-NMR spectroscopy (Supporting Information, Schemes S3, S4, and S7). l-Homoalanine (**2**) was also used as a substrate to determine if Aro8 or TyrB could set three contiguous stereocenters. Only Aro8 produced appreciable amounts of **13** (16% yield) with modest diastereoselectivity (70% de (2*S*,3*S*,4*R*); Supporting Information, Figure S21). The relative configuration of the major isomer of **13** was determined by converting the free amino acid into the lactone **22** and NOE analysis (Supporting Information, Scheme S5). The configuration of the α- and γ-stereocenters of **13** is therefore consistent with **5**, when synthesized with Aro8. Both Aro8 and TyrB accepted *p*-fluorobenzaldehyde in low yield (4% and 3%, respectively) with low to modest diastereoselectivity (72% de (2*S*,3*S*,4*S*) and 42% de (2*S*,3*S*,4*R*), respectively; Supporting Information, Figure S26). The α- and γ-stereocenters of **14** in the TyrB-catalyzed reaction are consistent with the stereocenters of **6**. These results indicate that the relative configuration of the α-amino and β-methyl groups are fixed, and the difference in diastereoselectivity between Aro8 and TyrB arises from the C4 stereocenter. No reaction (Figure 3d) was observed with any other amino acids, including l-norvaline (**16**), serine (**17**), or phenylalanine (1**8**), nor with l-alanine methyl ester (**21**). Addition of a phenyl group on isatin (**19**) was not tolerated. Transamination of isatin to **20** was not observed.

To further investigate the divergent stereoselectivity between Aro8 and TyrB, we turned to molecular docking to identify the noncovalent interactions (NCIs) responsible for selectivity (Supporting Information, Computational Details section). We opted to dock ketimine **VI** as it would enable additional NCIs involving the imine that would not be present in aldimine **VIII**. Additionally, we hypothesized that similar poses between the two enzymes could give insight into the residues responsible for the stereospecific protonation to set the α-stereocenter in both enzymes.

Using AutoDock Vina, poses for ketimines (*R*)-**VI** and (*S*)-**VI** were generated with a low root-mean-square deviation (RMSD) of atomic positions to PMP within the cocrystal structure of Aro8 (Figure 4a).^55,57^ For both stereoisomers, many of the interactions between the active site residues of Aro8 and the cocrystallized PMP are retained in the docked poses. Residues in the phosphate binding region (e.g., Thr142, Ser304, and Arg312) hydrogen bond with the 5′-phosphate group of PMP. The pyridinium N–H participates in hydrogen bonding with Asp248 for both stereoisomers, which is consistent with the cocrystal structure. Hydrogen bonding is present between Arg470 and the carboxylate of both ketimine diastereomers. Finally, each minimum RMSD pose orients the imine *Re* face toward Lys305, which is consistent with the observed selectivity and suggests Lys305 may be responsible for setting the α-stereocenter. Both (*S*)-**VI** and (*R*)-**VI** exhibit a donor–acceptor interaction between the aromatic surface of Tyr105 and the oxindole ring. Despite this common feature between the two diastereomers, the orientation of the oxindole ring is flipped between (*S*)-**VI** and (*R*)-**VI**. In both poses, the oxindole ring participates in donor–acceptor interactions with Tyr105, but the orientation of the amide nitrogen and carbonyl of (*R*)-**VI** (the major product of Aro8) is favorably positioned for hydrogen bonding with Gln335. This pose also projects the benzene ring toward a predominately nonpolar region of the active site near Ile29 and Leu44. In contrast, the pose for (*S*)-**VI** (the minor product of Aro8) unfavorably orients the polar functional groups of the oxindole toward the nonpolar region of the active site. The Vina scores for the poses reflect these disparate interactions and are −10.5 and −11.8 kcal mol^–1^ for (*S*)-**VI** and (*R*)-**VI**, respectively. Despite the small difference in Vina scores, we suspect that interactions with Gln335, Ile29, and Leu44 bias the approach of the prochiral faces of isatin toward the aminoacrylate **V** and thus the stereoselectivity of the reaction.

**Figure 4:**
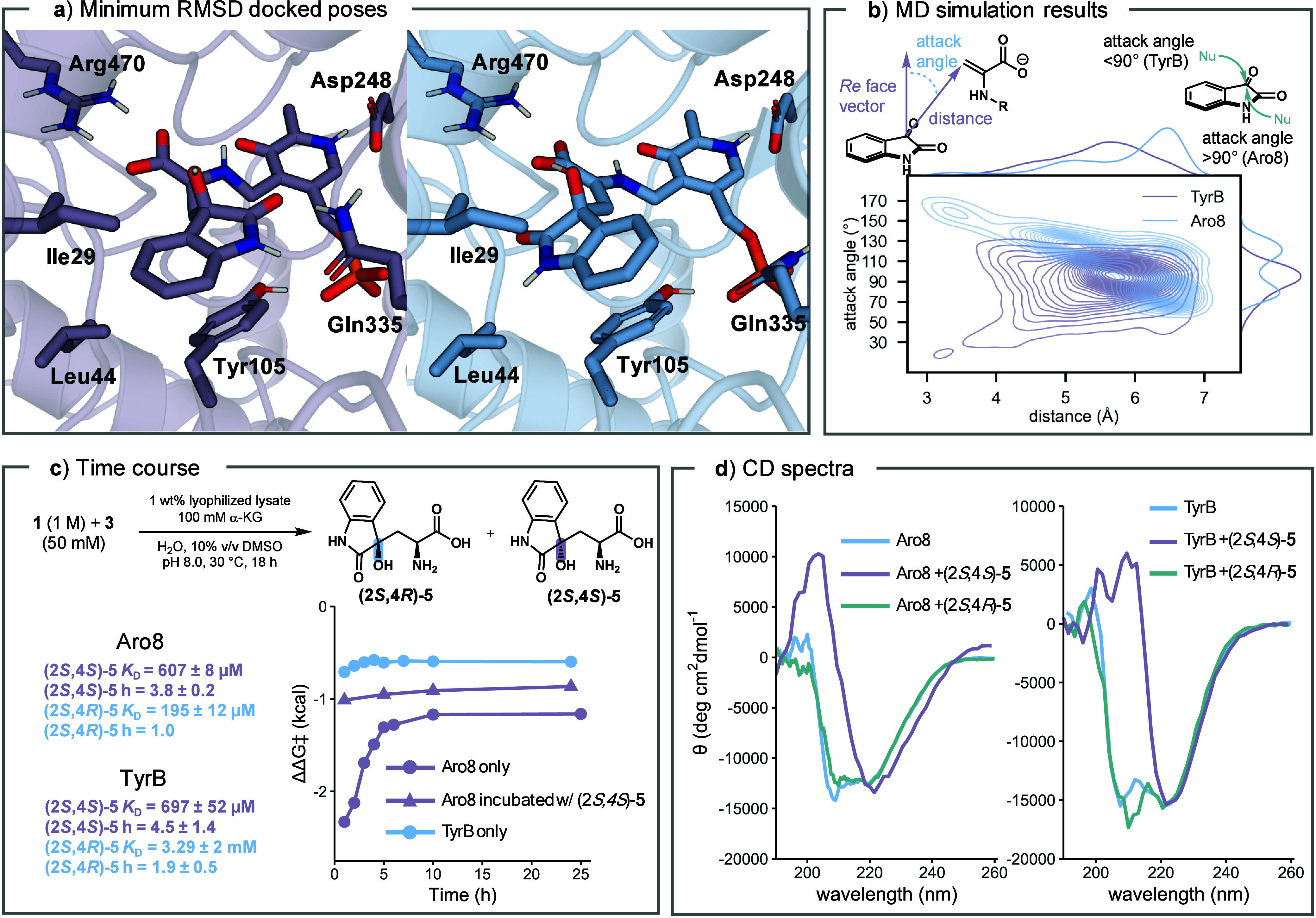
(a) Active site of Aro8 with minimum RMSD docked poses of ketimines (*R*)-**VI** (left) and (*S*)-**VI** (right). (b) Definitions and contour plot of two descriptors (distance and attack angle) computed over the course of the 200 ns GaMD simulation. (c) Time course study of both yield and selectivity for Aro8 (purple traces) and TyrB (blue trace), *n* = 3. Dissociation constants and Hill coefficients for Aro8 and TyrB determined by UV–vis spectroscopy titrations. Conditions: 20 μM purified enzyme in 50 mM KP_i_, pH 8.0, 1 mL total volume; absorbance at 353 nm (Aro8) and 364 nm (TyrB) was monitored with addition of isomers of **5** (*n* = 3). Total volume of **5** added was negligible. (d) CD spectra of Aro8 (left) and TyrB (right) showing a conformational change upon binding of (2*S*,4*S*)-**5**. Conditions: 10 μM purified enzyme in 50 mM KP_i_, pH 8.0, 300 μL total volume, 100 nm/min scan, *n* = 3.

For TyrB, many of the same interactions are observed for both (*S*)-**VI** and (*R*)-**VI** (Supporting Information, Figure S38).^58^ Interactions between the 5′-phosphate and Ser105, Ser246, and Arg255 are analogous to those seen in Aro8. Asp212 hydrogen bonds with the pyridinium N–H for both stereoisomers. The region surrounding the oxindole ring differs only slightly from Aro8. In the pose for (*S*)-**VI**, the benzene ring of the oxindole moiety is situated between Tyr66 and Met69. The pose for (*R*)-**VI** positions the amide functionality of the oxindole between the same residues, which may be responsible for the opposite selectivity of TyrB. The oxindole is positioned further away from surrounding residues in the crystal structure with less prominent NCIs. We suspect that the lack of NCIs between the two poses contributes to the lower diastereoselectivity of TyrB compared to Aro8.

To complement these docking results, we performed 200 ns Gaussian accelerated molecular dynamics (GaMD) simulations for both Aro8 and TyrB with docked structures of aminoacrylate **V** and **3**.^59^ Briefly, each system was equilibrated with conventional MD followed by equilibration with GaMD until all boost parameters converged. A single flat-bottom potential was applied between aminoacrylate **V** and **3** to prevent diffusion of **3** into the bulk solvent. For Aro8, **3** explored predominately pro-*R* poses and accessed near attack conformations (NACs) with a distance between the terminal aminoacrylate methylene and isatin carbonyl <3.4 Å (Figure 4b).^60^ Conversely, the TyrB simulation explored predominately pro-(*S*) NACs. The total volume explored by isatin in Aro8 and TyrB was 1222 and 2034 Å^3^, respectively. The substantially larger volume explored by isatin in the TyrB simulation suggests that the orientation of isatin is weakly controlled relative to Aro8, leading to reduced stereoselectivity. These data in combination with our docking studies suggest that the overall active site shape influences the facial approach of **3** to promote stereoselective nucleophilic addition.

To further investigate the differences between Aro8 and TyrB, we monitored the stereoselectivity of the reaction as it progressed (Figure 4c). Whereas TyrB exhibited a slight but constant preference for (2*S*,4*S*)-**5**, Aro8 exhibited high selectivity for (2*S*,4*R*)-**5** only at early reaction time points. However, this preference eroded over the course of the reaction. Incubation of (2*S*,4*R*)-**5** with Aro8 or TyrB only resulted in modest changes in de, and incubation of pure (2*S*,4*S*)-**5** with either Aro8 or TyrB did not lead to formation of (2*S*,4*R*)-**5**, suggesting that epimerization of the product was not sufficient to explain the loss in selectivity over time (Supporting Information, Figure S7). However, preincubation of 5 mM pure (2*S*,4*S*)-**5** before addition of isatin and l-alanine gave consistent diastereoselectivity over the course of the reaction, as was the case with TyrB. Intrigued by this result, we measured the binding affinities (*K*_D_) of the diastereomers of **5** by UV–vis spectroscopy to assess whether competitive inhibition contributed to the changing selectivity. In good agreement with the reaction outcome, the *K*_D_ for (2*S*,4*R*)-**5**—the major product—was slightly lower than the *K*_D_ for the minor diastereomer with Aro8, indicating that the major diastereomer has a more favorable binding mode to the enzyme. The difference in *K*_D_ values of the diastereomers with TyrB was even more extreme: the calculated *K*_D_ was 32 times greater for the minor diastereomer (2*S*,4*S*)-**5** than the major diastereomer. However, the Hill coefficients revealed that for TyrB, both diastereomers were positively cooperative (*h* > 1), whereas for Aro8, the major diastereomer was noncooperative (*h* = 1) and the minor diastereomer was positively cooperative (*h* > 1). These results were corroborated by circular dichroism (CD) spectroscopy, where major secondary structure changes were observed upon incubation of Aro8 with (2*S*,4*S*)-**5**. However, no major change in the CD spectrum was induced by the addition of (2*S*,4*R*)-**5** (Figure 4d). Based on CD spectroscopy, Aro8 is composed of 70.8% helices, 28.1% antiparallel sheets, and 1.1% parallel sheets. Upon binding (2*S*,4*S*)-**5**, helices are destabilized to 42.1%, antiparallel sheets are stabilized to 19.1%, and parallel sheets are stabilized to 38.8%. Similar results were obtained with TyrB, though the binding of the major (2*S*,4*S*) diastereomer is much stronger, indicating that the pro-(2*S*,4*S*) configuration is stabilized in the active site.

These results indicate that Aro8 can adopt two different conformations: one that is highly selective for formation of (2*S*,4*R*)-**5**, and one that is only slightly selective for (2*S*,4*R*)-**5**. Cooperative binding of (2*S*,4*S*)-**5** leads to a positive feedback loop in which, over time, Aro8 is converted to the latter conformer, reducing diastereoselectivity of the reaction but not swapping it entirely. This effect is not seen with TyrB because TyrB adopts a pro-(2*S*,4*S*) conformation and thus cooperativity reinforces the inherent selectivity rather than opposing it. Given this cooperativity, we anticipate TyrB will be amenable to further engineering to improve substrate compatibility and improve diastereoselectivity.

## Conclusion

In summary, we show that aminotransferases can catalyze a non-native C–C bond formation with unprotected α-amino acid substrates. Aro8 and TyrB are shown to be catalytically promiscuous aminotransferases that functionalize the β-carbon of l-alanine and l-homoalanine via formation of a nucleophilic aminoacrylate intermediate, which can be intercepted with electrophiles to form γ-hydroxy-α-amino acids. To our knowledge, such reactivity represents the first investigations of C–C bond formation catalyzed by aminotransferases, and a rare example of nucleophilic β-substitution with PLP-dependent enzymes. The outcome of this reaction is complicated by both competing transamination and conformational dynamics, which can be overcome by careful manipulation of reaction conditions. Ongoing studies into the structure of Aro8 conformers, as well as engineering efforts with TyrB, are underway in our lab. PLP-dependent aminotransferases are a promising, but underexplored, class of enzymes for C–C bond formation, and these results uncover the utility of catalytic promiscuity-guided reaction development for noncanonical amino acid synthesis. We anticipate that the wide availability of PLP-dependent aminotransferases renders this class accessible for catalytically promiscuous transformations, including C–C bond formation.

## Supplementary Material



## Notes

Experimental details, characterization data, reaction optimization conditions, computational details, and NMR spectra (PDF)

## Abbreviations

αKG: α-ketoglutarate
CD: circular dichroism
GaMD: Gaussian accelerated molecular dynamics
PLP: pyridoxal-5′-phosphate
PMP: pyridoxamine-5′-phosphate
MD: molecular dynamics
NAC: near attack conformation
NCI: noncovalent interaction
RMSD: root-mean-square deviation
