# Supplementary material for: Catalytically Promiscuous PLP-Dependent Aminotransferases Are Biocatalysts for C–C Bond Formation
Source: ACS Cent Sci. 2026 Jun 29;12(7):1029–37. doi: 10.1021/acscentsci.6c00531 (PMC13397446; doi:10.1021/acscentsci.6c00531)

Supporting Information for

Catalytically promiscuous PLP-dependent aminotransferases are biocatalysts for C–C bond formation

Alexander T. Kim[1,2], James R. Howard[4], Andrés G. Cuba Cáceres[1], Kyle I. Chong[1,3], William A. Aye[1], Matthew S. Sigman[4*], Alison R. H. Narayan[1,2,3*]

***Corresponding authors:**
Alison R. H. Narayan
Email: arhardin@umich.edu

Matthew S. Sigman
matt.sigman@utah.edu

[1]Life Sciences Institute, University of Michigan, Ann Arbor, Michigan 48109 USA.
[2]Program in Chemical Biology, University of Michigan, Ann Arbor, Michigan 48109 USA.
[3]Department of Chemistry, University of Michigan, Ann Arbor, Michigan 48109 USA.
[4]Department of Chemistry, University of Utah, Salt Lake City, Utah 84112 USA.

Table of Contents

General Information ... 3

Analytical Methods ... 3

Equipment and Materials ... 4

DNA and Protein Sequences ... 6

Aro8 and TyrB Sequences ... 6

PLP-Dependent Aminotransferase Library Composition ... 10

Library Profiling and Analytical-Scale Biocatalytic Reactions ... 20

Reactions in 96-Well Plates ... 20

Analytical-Scale Biocatalytic Reactions ... 23

UV-Vis and CD Spectroscopy ... 26

Determination of Dissociation Constants ... 26

Circular Dichroism (CD) Spectroscopy ... 29

Preparative-Scale Biocatalytic Reactions ... 29

Protein Production and Purification ... 29

Biocatalytic Synthesis of γ-Hydroxy-α-Amino Acids ... 32

Chemical Synthesis ... 40

Synthesis of dioxindolyl-L-alanine (**5**) standards ... 40

Synthesis of **6** standards ... 42

Synthesis of (2*S*,3*R*,4*S*)-**14** and (2*S*,3*R*,4*R*)-**14** standards ... 45

Synthesis of (2*S*,3*S*,4*S*)-**14** and (2*R*,3*S*,4*S*)-**14** standards ... 49

Synthesis of (2S,3S,4R)-**14** standard ... 52

Computational Details ... 54

General Computational Details ... 54

Preparation of Docking Hosts for Aro8 and TyrB ... 54

Flexible Docking of Ketimines to Aro8 (PDB 4JE5) ... 55

Flexible Docking of Ketimines to TyrB (PDB 3TAT) ... 56

Preparation of Forcefield Parameters for Nonstandard Residues ... 56

Preparing and relaxing explicitly solvated Aro8 and TyrB ... 56

Gaussian accelerated molecular dynamics (GaMD) ... 57

References ... 63

NMR Spectra ... 64

General Information

Analytical Methods

Thin Layer Chromatography:

TLC was performed on SiliCycle 60A F254 pre-coated silica TLC plates (0.25 mm). Samples were visualized under UV light, then optionally stained.

Thin Layer Chromatography Stains:

Permanganate: $KMnO_4$ (1 g) and K_2CO_3 (2 g) were dissolved in 100 mL water.

Ninhydrin: Ninhydrin (300 mg) and acetic acid (3 mL) were dissolved in 100 mL *n*-butanol.

Cerium ammonium molybdate (CAM): $Ce(NH_4)_4(SO_4)_4 \cdot 2H_2O$ (0.5 g), $(NH_4)_6Mo_7O_{24} \cdot 4H_2O$ (12 g), and concentrated sulfuric acid (15 mL) were dissolved in 235 mL water.

Nuclear Magnetic Resonance Spectroscopy:

Proton (^1H), carbon (^{13}C), and fluorine (^{19}F) spectra were obtained on a Bruker 600 MHz spectrometer. For quantitative ^{19}F NMR, the relaxation time was set to 30 s. Internal calibration was performed using solvent resonances (^1H NMR: CDCl3 at δ 7.26 ppm, D_2O at 4.79 ppm, CD_3CN at 1.94 ppm; ^{13}C NMR: $CDCl_3$ at δ 77.0 ppm, CD_3CN at 118.26 ppm; ^{19}F NMR: fluoroacetonitrile at -217.0 ppm). The following format is used for the reporting of ^1H NMR data: analysis solvent, magnet strength, chemical shift (ppm), multiplicity (s = singlet, br s = broad singlet, d = doublet, t = triplet, q = quartet, m = multiplet), *J*-coupling constants (Hz), and integration.

Liquid Chromatography-Mass Spectrometry:

Analysis was performed on the following instruments: Agilent 1260 UPLC/LC MSD, Agilent 1290 UPLC/6230B TOF, and Agilent 1260 UPLC/6530 Q-TOF with electrospray ionization and external calibration. All samples were prepared with LCMS-grade solvents (Millipore Sigma, LC-MS water from LC-Pak Polisher for Milli-Q) with 10 μM L-tryptophan methyl ester as internal standard, unless otherwise stated. UPLC separation was performed using an Acquity HSS T3 1.8 mm, 2.1 x 75 mm column.

The following UPLC solvents were used for separations:

(Solvent A) 0.1% formic acid in water.

(Solvent B) 95% acetonitrile, 5% water with 0.1% formic acid.

UV-Vis Spectroscopy:

Spectra were obtained using a Spectramax M5 Multi-Mode Microplate Reader (Molecular Devices). Optical Density (OD) measurements were obtained using an Implen OD600.

Equipment and Materials

Chemicals and Reagents

All chemicals and reagents were used as received from vendors unless otherwise stated. Chemicals and reagents were purchased from Sigma Aldrich, Fisher Scientific, Oakwood Chemical, Ambeed, GoldBio, Research Products International, Dot Scientific, SiliCycle, New England Biosciences, and Invitrogen.

Bacterial Cultures

96-well plates were incubated using ISF1-Z Incubator Shakers (Kuhner Shaker Inc). Large-scale cultures were incubated in Innova 44R Incubator Shakers (Eppendorf).

Media was prepared as follows:

> Terrific Broth (TB): tryptone (12.0 g, Research Products International), yeast extract (24.0 g, Dot Scientific), and 80 mL of 50% glycerol in water were dissolved in 820 mL Milli-Q water and sterilized by autoclave. A 10X phosphate buffer (0.89 M) was prepared using 94 g K_2HPO_4 and 17 g KH_2PO_4 and sterilized by autoclave. Phosphate buffer was added to 1X concentration. Kanamycin was added to a final concentration of 50 μg/mL.
>
> Luria Broth (LB): LB powder (20.0 g, Research Products International) was dissolved in 1 L Milli-Q water and sterilized by autoclave. Kanamycin was added to a final concentration of 50 μg/mL.

Molecular Biology

SDS-PAGE gel electrophoresis was performed with a Mini Protean Tetra Cell (Bio-Rad). DNA gel electrophoresis was performed with an Owl EasyCast B1A Mini Gel Electrophoresis System (Thermo Fisher Scientific).

Gels for electrophoresis were prepared as follows:

> SDS-PAGE: 3.8 mL Milli-Q water, 2 mL 40% acrylamide, 2 mL 1.5 M Tris pH 8.8, 80 μL 10% SDS, 80 μL 10% APS, and 8 μL TEMED were dissolved and added to casting plate to make the resolving gel. A layer of isopropanol was added to keep the surface of the gel uniform. After polymerization and removal of isopropanol, 3.1 mL Milli-Q water, 0.5 mL 40% acrylamide, 1.25 mL 0.5 M Tris pH 6.8, 50 μL 10% SDS, 50 μL 10% APS, and 5 μL TEMED were dissolved and added to casting plate to make the stacking gel.
>
> 0.8% Agarose: 0.4 g agarose (low electroendosmosis, molecular biology grade, GoldBio) was dissolved in 50 mL 1X TAE buffer. 2 μL SYBR Safe (Thermo Fisher Scientific) was added to this solution before pouring into casting mold.

Buffers for electrophoresis were prepared as follows:

> SDS-PAGE: Dilute to 1X from 10X Tris/Glycine/SDS premixed electrophoresis buffer (25 mM Tris, 192 mM glycine, 0.1% SDS, pH 8.3, Bio-Rad).
>
> DNA gels: Dilute to 1X from 50X Tris-Acetate-EDTA (TAE) buffer.

Minipreps were performed using QIAprep Spin Miniprep Kit (Qiagen).

High purity water (18.2 MΩ·cm @ 25 °C) was obtained from Milli-Q Reference Water Purification System (EMD Millipore).

Centrifugation

Microcentrifuge tubes were centrifuged using an accuSpin Micro 17R Microcentrifuge (Thermo Fisher Scientific) at 17,000×*g* unless otherwise stated. Larger vessels and volumes were centrifuged using a Sorvall LYNX 6000 Superspeed Centrifuge with variable centrifugal baskets (Thermo Fisher Scientific).

Flash Chromatography

Normal-phase chromatography was performed using a Biotage Isolera One and Biotage Sfär columns with SiliaFlash irregular silica gel P60 (40-63 µm, 60 Å, SiliCycle). Reverse-phase chromatography was performed on either a Waters 1525 HPLC with a Kinetex 5 µm C18 100 Å preparative HPLC column, or a CombiFlash RF200 (Teledyne ISCO) with a SiliaSep C4 80 g 40-63 µm, 60 Å column (SiliCycle).

The following flash chromatography solvents were used for reverse-phase separations:

(Solvent C) 0.1% trifluoroacetic acid in water.
(Solvent D) Methanol.

Dry Column Vacuum Chromatography

Dry column vacuum chromatography (DCVC) was performed using silica gel 60 (0.015-0.040 mm, Merck KGaA). Silica was packed into a 102 mm quick separation funnel with 30 mm coarse frit (ChemGlass) and solvent was collected into a 60 mL separatory funnel.

DNA and Protein Sequences

Aro8 and TyrB Sequences

Aro8

Full plasmid sequence (Aro8 gene in red):

ATGACCCTTCCGGAAAGCAAGGACTTTAGTTACCTGTTCAGTGACGAAACCAATGCGCGCAAACCGAGCCCGTTGAAAACGTGCATTC
ATCTGTTCCAAGACCCGAATATCATCTTTCTTGGCGGTGGGCTTCCGTTGAAAGACTACTTCCCGTGGGACAACTTGAGCGTCGATTCGC
CGAAGCCACCGTTCCCGCAAGGCATTGGCGCGCCGATCGATGAACAGAACTGCATCAAATATACCGTTAACAAAGATTACGCCGACA
AGTCGGCGAACCCTTCGAACGACATTCCGTTATCACGCGCCTTACAGTATGGTTTTTCCGCGGGTCAACCTGAGCTGCTGAACTTCATT
CGTGATCACACGAAAATTATTCACGATCTGAAATATAAAGATTGGGATGTATTGGCAACAGCTGGTAATACCAACGCGTGGGAAAGTACGC
TGCGGGTGTTTTGCAACCGTGGTGACGTAATTCTGGTAGAAGCCCATTCATTTAGTTCGAGTTTAGCAAGCGCAGAGGCGCAGGGTGTC
ATTACATTTCCCGTTCCTATCGATGCCGATGGGATCATCCCTGAGAAACTGGCGAAAGTTATGGAAAACTGGACTCCGGGCGCCCCAAA
ACCAAAACTGCTGTACACCATTCCAACGGGTCAAAACCCGACCGGAACGTCGATCGCCGATCATCGCAAAGAGGCTATTTATAAAATC
GCCCAGAAATACGACTTTCTGATCGTGGAAGATGAACCTTACTATTTTCTGCAAATGAACCCGTACATTAAAGATCTGAAAGAGCGTGAAA
AAGCCCAGAGCTCCCCTAAACAGGATCATGATGAATTCCTGAAGTCTCTTGCTAACACCTTTCTGAGTCTTGACACCGAGGGACGTGTCA
TTCGCATGGATTCTTTTAGCAAAGTTCTTGCACCGGGTACCCGCCTGGGTTGGATTACCGGGTCGTCTAAGATCTTAAAACCATATCTGTC
GCTTCACGAAATGACGATCCAAGCCCCGGCTGGTTTTACCCAGGTGCTCGTGAACGCAACCCTCTCTCGCTGGGGTCAAAAAGGCTA
CCTGGATTGGCTGCTCGGCCTGCGTCACGAATACACCCTTAAACGGGATTGCGCCATTGATGCTCTGTACAAATACTTACCTCAAAGCG
ATGCATTCGTCATCAACCCGCCTATTGCAGGAATGTTTTTCACGGTCAATATCGACGCAAGCGTTCATCCAGAATTTAAAACGAAATACAA
CAGTGACCCTTATCAGCTGGAGCAAAGCCTTTATCACAAAGTGGTTGAACGCGGCGTTTTAGTGGTCCCCGGTAGTTGGTTTAAATCCGA
AGGGGAGACCGAACCGCCCCAGCCTGCCGAATCTAAAGAAGTTAGTAATCCGAACATTATCTTTTTTCGGGGTACCTATGCAGCGGTGA
GCCCGGAGAAGTTAACTGAAGGCCTGAAACGCCTGGGTGACACGTTATATGAGGAATTTGGTATCAGTAAACTCGAGCACCACCACCA
CCACCACTGAGATCCGGCTGCTAACAAAGCCCGAAAGGAAGCTGAGTTGGCTGCTGCCACCGCTGAGCAATAACTAGCATAACCCCT
TGGGGCCTCTAAACGGGTCTTGAGGGGTTTTTTGCTGAAAGGAGGAACTATATCCGGATTGGCGAATGGGACGCGCCCTGTAGCGGC
GCATTAAGCGCGGCGGGTGTGGTGGTTACGCGCAGCGTGACCGCTACACTTGCCAGCGCCCTAGCGCCCGCTCCTTTCGCTTTCTT
CCCTTCCTTTCTCGCCACGTTCGCCGGCTTTCCCCGTCAAGCTCTAAATCGGGGGCTCCCTTTAGGGTTCCGATTTAGTGCTTTACGGC
ACCTCGACCCCAAAAAACTTGATTAGGGTGATGGTTCACGTAGTGGGCCATCGCCCTGATAGACGGTTTTTCGCCCTTTGACGTTGGAG
TCCACGTTCTTTAATAGTGGACTCTTGTTCCAAACTGGAACAACACTCAACCCTATCTCGGTCTATTCTTTTGATTTATAAGGGATTTTGCCG
ATTTCGGCCTATTGGTTAAAAAATGAGCTGATTTAACAAAAATTTAACGCGAATTTTAACAAAATATTAACGTTTACAATTTCAGGTGGCACTTT
TCGGGGAAATGTGCGCGGAACCCCTATTTGTTTATTTTTCTAAATACATTCAAATATGTATCCGCTCATGAATTAATTCTTAGAAAAACTCATC
GAGCATCAAATGAAACTGCAATTTATTCATATCAGGATTATCAATACCATATTTTTGAAAAAGCCGTTTCTGTAATGAAGGAGAAAACTCACC
GAGGCAGTTCCATAGGATGGCAAGATCCTGGTATCGGTCTGCGATTCCGACTCGTCCAACATCAATACAACCTATTAATTTCCCCTCGTC
AAAAATAAGGTTATCAAGTGAGAAATCACCATGAGTGACGACTGAATCCGGTGAGAATGGCAAAAGTTTATGCATTTCTTTCCAGACTTGTT
CAACAGGCCAGCCATTACGCTCGTCATCAAAATCACTCGCATCAACCAAACCGTTATTCATTCGTGATTGCGCCTGAGCGAGACGAAAT
ACGCGATCGCTGTTAAAAGGACAATTACAAACAGGAATCGAATGCAACCGGCGCAGGAACACTGCCAGCGCATCAACAATATTTTCAC
CTGAATCAGGATATTCTTCTAATACCTGGAATGCTGTTTTCCCGGGGATCGCAGTGGTGAGTAACCATGCATCATCAGGAGTACGGATAAA
ATGCTTGATGGTCGGAAGAGGCATAAATTCCGTCAGCCAGTTTAGTCTGACCATCTCATCTGTAACATCATTGGCAACGCTACCTTTGCCA
TGTTTCAGAAACAACTCTGGCGCATCGGGCTTCCCATACAATCGATAGATTGTCGCACCTGATTGCCCGACATTATCGCGAGCCCATTTA
TACCCATATAAATCAGCATCCATGTTGGAATTTAATCGCGGCCTAGAGCAAGACGTTTCCCGTTGAATATGGCTCATAACACCCCTTGTATT
ACTGTTTATGTAAGCAGACAGTTTTATTGTTCATGACCAAAATCCCTTAACGTGAGTTTTCGTTCCACTGAGCGTCAGACCCCGTAGAAAAG
ATCAAAGGATCTTCTTGAGATCCTTTTTTTCTGCGCGTAATCTGCTGCTTGCAAACAAAAAAACCACCGCTACCAGCGGTGGTTTGTTTGC
CGGATCAAGAGCTACCAACTCTTTTTCCGAAGGTAACTGGCTTCAGCAGAGCGCAGATACCAAATACTGTCCTTCTAGTGTAGCCGTAGT
TAGGCCACCACTTCAAGAACTCTGTAGCACCGCCTACATACCTCGCTCTGCTAATCCTGTTACCAGTGGCTGCTGCCAGTGGCGATAAG
TCGTGTCTTACCGGGTTGGACTCAAGACGATAGTTACCGGATAAGGCGCAGCGGTCGGGCTGAACGGGGGGTTCGTGCACACAGCC
CAGCTTGGAGCGAACGACCTACACCGAACTGAGATACCTACAGCGTGAGCTATGAGAAAGCGCCACGCTTCCCGAAGGGAGAAAGG
CGGACAGGTATCCGGTAAGCGGCAGGGTCGGAACAGGAGAGCGCACGAGGGAGCTTCCAGGGGGAAACGCCTGGTATCTTTATAGT
CCTGTCGGGTTTCGCCACCTCTGACTTGAGCGTCGATTTTTGTGATGCTCGTCAGGGGGGCGGAGCCTATGGAAAAACGCCAGCAAC
GCGGCCTTTTTACGGTTCCTGGCCTTTTGCTGGCCTTTTGCTCACATGTTCTTTCCTGCGTTATCCCCTGATTCTGTGGATAACCGTATTAC
CGCCTTTGAGTGAGCTGATACCGCTCGCCGCAGCCGAACGACCGAGCGCAGCGAGTCAGTGAGCGAGGAAGCGGAAGAGCGCCT
GATGCGGTATTTTCTCCTTACGCATCTGTGCGGTATTTCACACCGCATATATGGTGCACTCTCAGTACAATCTGCTCTGATGCCGCATAGTT

AAGCCAGTATACACTCCGCTATCGCTACGTGACTGGGTCATGGCTGCGCCCCGACACCCGCCAACACCCGCTGACGCGCCCTGAC
GGGCTTGTCTGCTCCCGGCATCCGCTTACAGACAAGCTGTGACCGTCTCCGGGAGCTGCATGTGTCAGAGGTTTTCACCGTCATCACC
GAAACGCGCGAGGCAGCTGCGGTAAAGCTCATCAGCGTGGTCGTGAAGCGATTCACAGATGTCTGCCTGTTCATCCGCGTCCAGCTC
GTTGAGTTTCTCCAGAAGCGTTAATGTCTGGCTTCTGATAAAGCGGGCCATGTTAAGGGCGGTTTTTTCCTGTTTGGTCACTGATGCCTCC
GTGTAAGGGGGATTTCTGTTCATGGGGGTAATGATACCGATGAAACGAGAGAGGATGCTCACGATACGGGTTACTGATGATGAACATGCC
CGGTTACTGGAACGTTGTGAGGGTAAACAACTGGCGGTATGGATGCGGCGGGACCAGAGAAAAATCACTCAGGGTCAATGCCAGCGC
TTCGTTAATACAGATGTAGGTGTTCCACAGGGTAGCCAGCAGCATCCTGCGATGCAGATCCGGAACATAATGGTGCAGGGCGCTGACTT
CCGCGTTTCCAGACTTTACGAAACACGGAAACCGAAGACCATTCATGTTGTTGCTCAGGTCGCAGACGTTTTGCAGCAGCAGTCGCTTC
ACGTTCGCTCGCGTATCGGTGATTCATTCTGCTAACCAGTAAGGCAACCCCGCCAGCCTAGCCGGGTCCTCAACGACAGGAGCACGA
TCATGCGCACCCGTGGGGCCGCCATGCCGGCGATAATGGCCTGCTTCTCGCCGAAACGTTTGGTGGCGGGACCAGTGACGAAGGC
TTGAGCGAGGGCGTGCAAGATTCCGAATACCGCAAGCGACAGGCCGATCATCGTCGCGCTCCAGCGAAAGCGGTCCTCGCCGAAA
ATGACCCAGAGCGCTGCCGGCACCTGTCCTACGAGTTGCATGATAAAGAAGACAGTCATAAGTGCGGCGACGATAGTCATGCCCCGC
GCCCACCGGAAGGAGCTGACTGGGTTGAAGGCTCTCAAGGGCATCGGTCGAGATCCCGGTGCCTAATGAGTGAGCTAACTTACATTAA
TTGCGTTGCGCTCACTGCCCGCTTTCCAGTCGGGAAACCTGTCGTGCCAGCTGCATTAATGAATCGGCCAACGCGCGGGGAGAGGC
GGTTTGCGTATTGGGCGCCAGGGTGGTTTTTCTTTTCACCAGTGAGACGGGCAACAGCTGATTGCCCTTCACCGCCTGGCCCTGAGAG
AGTTGCAGCAAGCGGTCCACGCTGGTTTGCCCCAGCAGGCGAAAATCCTGTTTGATGGTGGTTAACGGCGGGATATAACATGAGCTGT
CTTCGGTATCGTCGTATCCCACTACCGAGATGTCCGCACCAACGCGCAGCCCGGACTCGGTAATGGCGCGCATTGCGCCCAGCGCC
ATCTGATCGTTGGCAACCAGCATCGCAGTGGGAACGATGCCCTCATTCAGCATTTGCATGGTTTGTTGAAAACCGGACATGGCACTCCA
GTCGCCTTCCCGTTCCGCTATCGGCTGAATTTGATTGCGAGTGAGATATTTATGCCAGCCAGCCAGACGCAGACGCGCCGAGACAGAA
CTTAATGGGCCCGCTAACAGCGCGATTTGCTGGTGACCCAATGCGACCAGATGCTCCACGCCCAGTCGCGTACCGTCTTCATGGGAG
AAAATAATACTGTTGATGGGTGTCTGGTCAGAGACATCAAGAAATAACGCCGGAACATTAGTGCAGGCAGCTTCCACAGCAATGGCATC
CTGGTCATCCAGCGGATAGTTAATGATCAGCCCACTGACGCGTTGCGCGAGAAGATTGTGCACCGCCGCTTTACAGGCTTCGACGCC
GCTTCGTTCTACCATCGACACCACCACGCTGGCACCCAGTTGATCGGCGCGAGATTTAATCGCCGCGACAATTTGCGACGGCGCGTG
CAGGGCCAGACTGGAGGTGGCAACGCCAATCAGCAACGACTGTTTGCCCGCCAGTTGTTGTGCCACGCGGTTGGGAATGTAATTCAG
CTCCGCCATCGCCGCTTCCACTTTTTCCCGCGTTTTCGCAGAAACGTGGCTGGCCTGGTTCACCACGCGGGAAACGGTCTGATAAGA
GACACCGGCATACTCTGCGACATCGTATAACGTTACTGGTTTCACATTCACCACCCTGAATTGACTCTCTTCCGGGCGCTATCATGCCAT
ACCGCGAAAGGTTTTGCGCCATTCGATGGTGTCCGGGATCTCGACGCTCTCCCTTATGCGACTCCTGCATTAGGAAGCAGCCCAGTAG
TAGGTTGAGGCCGTTGAGCACCGCCGCCGCAAGGAATGGTGCATGCAAGGAGATGGCGCCCAACAGTCCCCCGGCCACGGGGCC
TGCCACCATACCCACGCCGAAACAAGCGCTCATGAGCCCGAAGTGGCGAGCCCGATCTTCCCCATCGGTGATGTCGGCGATATAGG
CGCCAGCAACCGCACCTGTGGCGCCGGTGATGCCGGCCACGATGCGTCCGGCGTAGAGGATCGAGATCGATCTCGATCCCGCGAA
ATTAATACGACTCACTATAGGGGAATTGTGAGCGGATAACAATTCCCCTCTAGAAATAATTTTGTTTAACTTTAAGAAGGAGATATACAT

Aro8 protein sequence:

MTLPESKDFSYLFSDETNARKPSPLKTCIHLFQDPNIIFLGGGLPLKDYFPWDNLSVDSPKPPFPQGIGAPIDEQNCIKYTVNKDYADKSANP
SNDIPLSRALQYGFSAGQPELLNFIRDHTKIIHDLKYKDWDVLATAGNTNAWESTLRVFCNRGDVILVEAHSFSSSLASAEAQGVITFPVPIDA
DGIIPEKLAKVMENWTPGAPKPKLLYTIPTGQNPTGTSIADHRKEAIYKIAQKYDFLIVEDEPYYFLQMNPYIKDLKEREKAQSSPKQDHDEFLK
SLANTFLSLDTEGRVIRMDSFSKVLAPGTRLGWITGSSKILKPYLSLHEMTIQAPAGFTQVLVNATLSRWGQKGYLDWLLGLRHEYTLKRDCA
IDALYKYLPQSDAFVINPPIAGMFFTVNIDASVHPEFKTKYNSDPYQLEQSLYHKVVERGVLVVPGSWFKSEGETEPPQPAESKEVSNPNIIFF
RGTYAAVSPEKLTEGLKRLGDTLYEEFGISK

TyrB

Full plasmid sequence (TyrB gene in red):

ATGTTCCAGAAAGTAGACGCCTATGCGGGCGACCCGATTCTCACACTGATGGAGCGGTTCAAAGAAGACCCACGTAGCGATAAAGTGA
ACCTGAGCATTGGCTTGTATTACAACGAGGATGGTATTATCCCGCAATTGCAAGCGGTCGCGGAAGCAGAGGCGCGCCTGAACGCCC
AGCCGCACGGTGCGTCCCTGTACCTGCCGATGGAGGGCCTGAACTGCTATCGTCACGCCATTGCGCCGCTGCTGTTTGGCGCAGAC
CATCCAGTTCTGAAACAGCAGCGTGTGGCCACCATTCAGACCTTGGGTGGTTCCGGTGCGCTCAAGGTGGGCGCTGACTTTTTAAAAC
GCTACTTTCCGGAGTCTGGTGTGTGGGTGTCAGATCCGACCTGGGAAAACCACGTCGCAATTTTTGCCGGGGCGGGTTTCGAGGTCAG
CACATATCCGTGGTACGATGAAGCCACCAATGGTGTTCGTTTCAATGACTTACTGGCCACGCTGAAAACTCTTCCCGCCCGTTCAATTGT
CCTGTTGCATCCATGCTGTCACAACCCTACCGGCGCGGATTTAACAAACGACCAGTGGGACGCGGTAATTGAGATCCTCAAAGCTCGC
GAGCTGATCCCGTTCCTCGATATTGCATATCAGGGCTTCGGGGCGGGCATGGAAGAAGACGCATACGCCATTCGCGCCATCGCGAGC
GCGGGGTTGCCGGCCTTAGTGTCCAACAGTTTCTCGAAGATTTTCAGCCTGTATGGAGAACGGGTTGGCGGCCTGTCGGTCATGTGTG
AAGACGCTGAAGCAGCGGGCCGCGTTCTCGGTCAGCTGAAAGCAACGGTACGGCGTAATTATAGCTCCCCCCCAAATTTCGGCGCG

CAGGTCGTCGCCGCCGTCCTTAATGATGAAGCTCTCAAAGCGTCCTGGCTGGCAGAAGTCGAAGAGATGCGTACCCGTATTCTGGCCA
TGCGGCAAGAACTGGTGAAAGTTTTGAGCACGGAAATGCCGGAACGCAACTTTGACTACTTGCTGAATCAACGTGGTATGTTCAGCTATA
CTGGGCTGTCTGCTGCACAGGTGGATCGCCTGCGCGAAGAATTTGGTGTTTATCTGATTGCCTCTGGTCGCATGTGTGTGGCAGGTCTG
AATACCGCAAACGTCCAGCGCGTTGCTAAAGCCTTTGCCGCGGTCATGCTCGAGCACCACCACCACCACCACTGAGATCCGGCTGC
TAACAAAGCCCGAAAGGAAGCTGAGTTGGCTGCTGCCACCGCTGAGCAATAACTAGCATAACCCCTTGGGGCCTCTAAACGGGTCTT
GAGGGGTTTTTTGCTGAAAGGAGGAACTATATCCGGATTGGCGAATGGGACGCGCCCTGTAGCGGCGCATTAAGCGCGGCGGGTGTG
GTGGTTACGCGCAGCGTGACCGCTACACTTGCCAGCGCCCTAGCGCCCGCTCCTTTCGCTTTCTTCCCTTCCTTTCTCGCCACGTTC
GCCGGCTTTCCCCGTCAAGCTCTAAATCGGGGGCTCCCTTTAGGGTTCCGATTTAGTGCTTTACGGCACCTCGACCCCAAAAAACTTG
ATTAGGGTGATGGTTCACGTAGTGGGCCATCGCCCTGATAGACGGTTTTTCGCCCTTTGACGTTGGAGTCCACGTTCTTTAATAGTGGACT
CTTGTTCCAAACTGGAACAACACTCAACCCTATCTCGGTCTATTCTTTTGATTTATAAGGGATTTTGCCGATTTCGGCCTATTGGTTAAAAAA
TGAGCTGATTTAACAAAAATTTAACGCGAATTTTAACAAAATATTAACGCTTACAATTTAGGTGGCACTTTTCGGGGAAATGTGCGCGGAAC
CCCTATTTGTTTATTTTTCTAAATACATTCAAATATGTATCCGCTCATGAATTAATTCTTAGAAAAACTCATCGAGCATCAAATGAAACTGCAATT
TATTCATATCAGGATTATCAATACCATATTTTTGAAAAAGCCGTTTCTGTAATGAAGGAGAAAACTCACCGAGGCAGTTCCATAGGATGGCA
AGATCCTGGTATCGGTCTGCGATTCCGACTCGTCCAACATCAATACAACCTATTAATTTCCCCTCGTCAAAAATAAGGTTATCAAGTGAGAA
ATCACCATGAGTGACGACTGAATCCGGTGAGAATGGCAAAAGTTTATGCATTTCTTTCCAGACTTGTTCAACAGGCCAGCCATTACGCTC
GTCATCAAAATCACTCGCATCAACCAAACCGTTATTCATTCGTGATTGCGCCTGAGCGAGACGAAATACGCGATCGCTGTTAAAAGGACA
ATTACAAACAGGAATCGAATGCAACCGGCGCAGGAACACTGCCAGCGCATCAACAATATTTTCACCTGAATCAGGATATTCTTCTAATAC
CTGGAATGCTGTTTTCCCGGGGATCGCAGTGGTGAGTAACCATGCATCATCAGGAGTACGGATAAAATGCTTGATGGTCGGAAGAGGCA
TAAATTCCGTCAGCCAGTTTAGTCTGACCATCTCATCTGTAACATCATTGGCAACGCTACCTTTGCCATGTTTCAGAAACAACTCTGGCGC
ATCGGGCTTCCCATACAATCGATAGATTGTCGCACCTGATTGCCCGACATTATCGCGAGCCCATTTATACCCATATAAATCAGCATCCATG
TTGGAATTTAATCGCGGCCTAGAGCAAGACGTTTCCCGTTGAATATGGCTCATAACACCCCTTGTATTACTGTTTATGTAAGCAGACAGTTT
TATTGTTCATGACCAAAATCCCTTAACGTGAGTTTTCGTTCCACTGAGCGTCAGACCCCGTAGAAAAGATCAAAGGATCTTCTTGAGATCC
TTTTTTTCTGCGCGTAATCTGCTGCTTGCAAACAAAAAAACCACCGCTACCAGCGGTGGTTTGTTTGCCGGATCAAGAGCTACCAACTCT
TTTTCCGAAGGTAACTGGCTTCAGCAGAGCGCAGATACCAAATACTGTCCTTCTAGTGTAGCCGTAGTTAGGCCACCACTTCAAGAACTC
TGTAGCACCGCCTACATACCTCGCTCTGCTAATCCTGTTACCAGTGGCTGCTGCCAGTGGCGATAAGTCGTGTCTTACCGGGTTGGACT
CAAGACGATAGTTACCGGATAAGGCGCAGCGGTCGGGCTGAACGGGGGGTTCGTGCACACAGCCCAGCTTGGAGCGAACGACCTA
CACCGAACTGAGATACCTACAGCGTGAGCTATGAGAAAGCGCCACGCTTCCCGAAGGGAGAAAGGCGGACAGGTATCCGGTAAGCG
GCAGGGTCGGAACAGGAGAGCGCACGAGGGAGCTTCCAGGGGGAAACGCCTGGTATCTTTATAGTCCTGTCGGGTTTCGCCACCTCT
GACTTGAGCGTCGATTTTTGTGATGCTCGTCAGGGGGGCGGAGCCTATGGAAAAACGCCAGCAACGCGGCCTTTTTACGGTTCCTGG
CCTTTTGCTGGCCTTTTGCTCACATGTTCTTTCCTGCGTTATCCCCTGATTCTGTGGATAACCGTATTACCGCCTTTGAGTGAGCTGATACC
GCTCGCCGCAGCCGAACGACCGAGCGCAGCGAGTCAGTGAGCGAGGAAGCGGAAGAGCGCCTGATGCGGTATTTTCTCCTTACGC
ATCTGTGCGGTATTTCACACCGCAATGGTGCACTCTCAGTACAATCTGCTCTGATGCCGCATAGTTAAGCCAGTATACACTCCGCTATCG
CTACGTGACTGGGTCATGGCTGCGCCCCGACACCCGCCAACACCCGCTGACGCGCCCTGACGGGCTTGTCTGCTCCCGGCATCC
GCTTACAGACAAGCTGTGACCGTCTCCGGGAGCTGCATGTGTCAGAGGTTTTCACCGTCATCACCGAAACGCGCGAGGCAGCTGCG
GTAAAGCTCATCAGCGTGGTCGTGAAGCGATTCACAGATGTCTGCCTGTTCATCCGCGTCCAGCTCGTTGAGTTTCTCCAGAAGCGTTAA
TGTCTGGCTTCTGATAAAGCGGGCCATGTTAAGGGCGGTTTTTTCCTGTTTGGTCACTGATGCCTCCGTGTAAGGGGGATTTCTGTTCATG
GGGGTAATGATACCGATGAAACGAGAGAGGATGCTCACGATACGGGTTACTGATGATGAACATGCCCGGTTACTGGAACGTTGTGAGGG
TAAACAACTGGCGGTATGGATGCGGCGGGACCAGAGAAAAATCACTCAGGGTCAATGCCAGCGCTTCGTTAATACAGATGTAGGTGTTC
CACAGGGTAGCCAGCAGCATCCTGCGATGCAGATCCGGAACATAATGGTGCAGGGCGCTGACTTCCGCGTTTCCAGACTTTACGAAA
CACGGAAACCGAAGACCATTCATGTTGTTGCTCAGGTCGCAGACGTTTTGCAGCAGCAGTCGCTTCACGTTCGCTCGCGTATCGGTGAT
TCATTCTGCTAACCAGTAAGGCAACCCCGCCAGCCTAGCCGGGTCCTCAACGACAGGAGCACGATCATGCGCACCCGTGGGGCCG
CCATGCCGGCGATAATGGCCTGCTTCTCGCCGAAACGTTTGGTGGCGGGACCAGTGACGAAGGCTTGAGCGAGGGCGTGCAAGATT
CCGAATACCGCAAGCGACAGGCCGATCATCGTCGCGCTCCAGCGAAAGCGGTCCTCGCCGAAAATGACCCAGAGCGCTGCCGGC
ACCTGTCCTACGAGTTGCATGATAAAGAAGACAGTCATAAGTGCGGCGACGATAGTCATGCCCCGCGCCCACCGGAAGGAGCTGACT
GGGTTGAAGGCTCTCAAGGGCATCGGTCGAGATCCCGGTGCCTAATGAGTGAGCTAACTTACATTAATTGCGTTGCGCTCACTGCCCG
CTTTCCAGTCGGGAAACCTGTCGTGCCAGCTGCATTAATGAATCGGCCAACGCGCGGGGAGAGGCGGTTTGCGTATTGGGCGCCAG
GGTGGTTTTTCTTTTCACCAGTGAGACGGGCAACAGCTGATTGCCCTTCACCGCCTGGCCCTGAGAGAGTTGCAGCAAGCGGTCCAC
GCTGGTTTGCCCCAGCAGGCGAAAATCCTGTTTGATGGTGGTTAACGGCGGGATATAACATGAGCTGTCTTCGGTATCGTCGTATCCCA
CTACCGAGATGTCCGCACCAACGCGCAGCCCGGACTCGGTAATGGCGCGCATTGCGCCCAGCGCCATCTGATCGTTGGCAACCAG
CATCGCAGTGGGAACGATGCCCTCATTCAGCATTTGCATGGTTTGTTGAAAACCGGACATGGCACTCCAGTCGCCTTCCCGTTCCGCTA
TCGGCTGAATTTGATTGCGAGTGAGATATTTATGCCAGCCAGCCAGACGCAGACGCGCCGAGACAGAACTTAATGGGCCCGCTAACAG
CGCGATTTGCTGGTGACCCAATGCGACCAGATGCTCCACGCCCAGTCGCGTACCGTCTTCATGGGAGAAAATAATACTGTTGATGGGT

GTCTGGTCAGAGACATCAAGAAATAACGCCGGAACATTAGTGCAGGCAGCTTCCACAGCAATGGCATCCTGGTCATCCAGCGGATAGT
TAATGATCAGCCCACTGACGCGTTGCGCGAGAAGATTGTGCACCGCCGCTTTACAGGCTTCGACGCCGCTTCGTTCTACCATCGACAC
CACCACGCTGGCACCCAGTTGATCGGCGCGAGATTTAATCGCCGCGACAATTTGCGACGGCGCGTGCAGGGCCAGACTGGAGGTG
GCAACGCCAATCAGCAACGACTGTTTGCCCGCCAGTTGTTGTGCCACGCGGTTGGGAATGTAATTCAGCTCCGCCATCGCCGCTTCC
ACTTTTTCCCGCGTTTTCGCAGAAACGTGGCTGGCCTGGTTCACCACGCGGGAAACGGTCTGATAAGAGACACCGGCATACTCTGCGA
CATCGTATAACGTTACTGGTTTCACATTCACCACCCTGAATTGACTCTCTTCCGGGCGCTATCATGCCATACCGCGAAAGGTTTTGCGCC
ATTCGATGGTGTCCGGGATCTCGACGCTCTCCCTTATGCGACTCCTGCATTAGGAAGCAGCCCAGTAGTAGGTTGAGGCCGTTGAGCA
CCGCCGCCGCAAGGAATGGTGCATGCAAGGAGATGGCGCCCAACAGTCCCCCGGCCACGGGGCCTGCCACCATACCCACGCCG
AAACAAGCGCTCATGAGCCCGAAGTGGCGAGCCCGATCTTCCCCATCGGTGATGTCGGCGATATAGGCGCCAGCAACCGCACCTGT
GGCGCCGGTGATGCCGGCCACGATGCGTCCGGCGTAGAGGATCGAGATCGATCTCGATCCCGCGAAATTAATACGACTCACTATAGG
GGAATTGTGAGCGGATAACAATTCCCCTCTAGAAATAATTTTGTTTAACTTTAAGAAGGAGATATACAT

TyrB protein sequence:

MFQKVDAYAGDPILTLMERFKEDPRSDKVNLSIGLYYNEDGIIPQLQAVAEAEARLNAQPHGASLYLPMEGLNCYRHAIAPLLFGADHPVLK
QQRVATIQTLGGSGALKVGADFLKRYFPESGVWVSDPTWENHVAIFAGAGFEVSTYPWYDEATNGVRFNDLLATLKTLPARSIVLLHPCCHN
PTGADLTNDQWDAVIEILKARELIPFLDIAYQGFGAGMEEDAYAIRAIASAGLPALVSNSFSKIFSLYGERVGGLSVMCEDAEAAGRVLGQLKA
TVRRNYSSPPNFGAQVVAAVLNDEALKASWLAEVEEMRTRILAMRQELVKVLSTEMPERNFDYLLNQRGMFSYTGLSAAQVDRLREEFGVY
LIASGRMCVAGLNTANVQRVAKAFAAVM

PLP-Dependent Aminotransferase Library Composition

Table S1: Enzymes in this study

Well	Gene	Enzyme	Uniprot ID
A1	mfnC	(5-formylfuran-3-yl)methyl phosphate transaminase	Q58097
A2	kdnA	8-amino-3,8-dideoxy-alpha-D-manno-octulosonate transaminase	Q8EEB1
A3	AGX1	Alanine-glyoxylate aminotransferase 1	P43567
A4	TyrB	aromatic-amino-acid aminotransferase	P04693
A5	ilvE	branched-chain-amino-acid aminotransferase	P0AB80
A6	ectB	Diaminobutyrate--2-oxoglutarate transaminase	Q9ZEU7
A7	OAT	Ornithine aminotransferase	P04181
A8	pucG	(S)-ureidoglycine--glyoxylate transaminase	O32148
A9	argD	Acetylornithine aminotransferase	Q9X2A5
A10	aspC	Aspartate aminotransferase	Q9X0Y2
A11	bioK	L-Lysine--8-amino-7-oxononanoate transaminase	P53555
A12	argD	Acetylornithine/succinyldiaminopimelate aminotransferase	P40732
B1	aspC	Aspartate/prephenate aminotransferase	Q56232
B2	cqsA	CAI-1 autoinducer synthase	Q9KM65
B3	lysJ	[LysW]-aminoadipate semialdehyde transaminase	Q5SHH5
B4	hisC	Histidinol-phosphate aminotransferase	Q9X0D0
B5	pat	Putative phenylalanine aminotransferase	P9WML5
B6	ntdA	3-oxo-glucose-6-phosphate:glutamate aminotransferase	O07566
B7	fumI	Aminopentol aminotransferase	D2D3B2
B8	bauA	Beta-alanine--pyruvate aminotransferase	Q9I700
B9	per	GDP-perosamine synthase	Q9A9H3
B10	patA	Putrescine aminotransferase	P42588
B11	gabT	4-aminobutyrate aminotransferase GabT	P22256
B12	bioA	Adenosylmethionine-8-amino-7-oxononanoate aminotransferase	P12995
C1	ALD1	Aminotransferase ALD1, chloroplastic	Q9ZQI7
C2	BFAT	Beta-phenylalanine transaminase	H8WR05
C3	ppaT	Pyridoxamine--pyruvate transaminase	Q988B8
C4	lysN	2-aminoadipate transaminase	Q72LL6

C5	GPT2	Alanine aminotransferase 2	Q8TD30
C6	pigE	Aminotransferase PigE	A0A0J9X1Q5
C7	BIO3	Bifunctional dethiobiotin synthetase/7,8-diamino-pelargonic acid aminotransferase, mitochondrial	B0F481
C8	dat	D-alanine aminotransferase	P19938
C9	phnW	2-aminoethylphosphonate--pyruvate transaminase	P96060
C10	Aro8	Aromatic/aminoadipate aminotransferase 1	P53090
C11	alaA	Glutamate-pyruvate aminotransferase AlaA	P0A959
C12	hisC	Histidinol-phosphate aminotransferase	Q39YP6
D1	wbpE	UDP-2-acetamido-2-deoxy-3-oxo-D-glucuronate aminotransferase	Q9HZ76
D2	rbmB	L-glutamine:2-deoxy-scyllo-inosose aminotransferase	Q4R0W2
D3	astC	Succinylornithine transaminase	P77581
D4	arnB	UDP-4-amino-4-deoxy-L-arabinose--oxoglutarate aminotransferase	Q8ZNF3
D5	dapL	LL-diaminopimelate aminotransferase	O84395
D6	OAPT	Omega-amino acid--pyruvate aminotransferase	P28269
D7	pglE	UDP-N-acetylbacillosamine transaminase	Q0P9D3
D8	ALA2	Alanine aminotransferase 2	P52894
D9	serC	Phosphoserine aminotransferase	Q9RME2
D10	AGT1	Serine--glyoxylate aminotransferase	Q56YA5
D11	TAT	Tyrosine aminotransferase	P17735
D12	ABAT	4-aminobutyrate aminotransferase, mitochondrial	P80147
E1	ybdL	Methionine aminotransferase	P77806
E2	ilvE	Probable branched-chain-amino-acid aminotransferase	P74921
E3	AGXT	Serine--pyruvate aminotransferase	P21549
E4	neoN	Neamine transaminase NeoN	Q53U08
E5	ilvE	Putative branched-chain-amino-acid aminotransferase	O29329

A1: Q58097

MLSKRLLNFESFEVMDILALAQKLESEGKKVIHLEIGEPDFNTPKPIVDEGIKSLKEGKTHYTDSRGILELREKISELYKDKYKADIIP
DNIIITGGSSLGLFFALSSIIDDGDEVLIQNPCYPCYKNFIRFLGAKPVFCDFTVESLEEALSDKTKAIIINSPSNPLGEVIDREIYEFA
YENIPYIISDEIYNGLVYEGKCYSAIEFDENLEKTILINGFSKLYAMTGWRIGYVISNDEIIEAILKLQQNLFISAPTISQYAALKAFEKE
TEREINSMIKEFDRRRRLVLKYVKDFGWEVNNPIGAYYVFPNIGEDGREFAYKLLKEKFVALTPGIGFGSKGKNYIRISYANSYENI
KEGLERIKEFLNK

A2: Q8EEB1

MPGFELFGPEEKQEVADVMEHGFTFRYNFDHMRNDRWKTRDMEQLLCEKMNVKHAHLLSSGTAALQTAMMAAGIGAGDEV
IVPPFTFVASVEAIFMAGAVPIFAEIDETLCLSPEGIEAVITPRTKAINLVHMCGSMAKMDEIKAICKKHNLVLLEDACQAIGGSYK
GQALGTIGDVGCYSFDSVKTITCGEGGAVITNNTEIYDNAHMFSDHGHDHIGKDRGAESHPIMGLNFRISEMNAALGLAQLRK
LDTIIDIQRKNKKAIKDAMASIPEVSFREIPDPEGDSAGFLSFMLPTEARTQEISKKLAANGVDGCFYWYVNNWHYLKNWKHIQ
ELKAPAALPITLIADRPDYTQISVPKSDAIMSRTISMLIKLSWTDAQIAERIENIKKAFAQ

A3: P43567

MTKSVDTLLIPGPIILSGAVQKALDVPSLGHTSPEFVSIFQRVLKNTRAVFKSAAASKSQPFVLAGSGTLGWDIFASNFILSKAPNK
NVLVVSTGTFSDRFADCLRSYGAQVDVVRPLKIGESVPLELITEKLSQNSYGAVTVTHVDTSTAVLSDLKAISQAIKQTSPETFFVV
DAVCSIGCEEFEFDEWGVDFALTASQKAIGAPAGLSISLCSSRFMDYALNDSKNGHVHGYFSSLRRWTPIMENYEAGKGAYFA
TPPVQLINSLDVALKEILEEGLHKRWDLHREMSDWFKDSLVNGLQLTSVSRYPSNMSAHGLTAVYVADPPDVIAFLKSHGVVIA
GGIHKDIGPKYIRIGHMGVTACNKNLPYMKNCFDLIKLALQRKK

A4: P04693

MFQKVDAYAGDPILTLMERFKEDPRSDKVNLSIGLYYNEDGIIPQLQAVAEAEARLNAQPHGASLYLPMEGLNCYRHAIAPLLF
GADHPVLKQQRVATIQTLGGSGALKVGADFLKRYFPESGVWVSDPTWENHVAIFAGAGFEVSTYPWYDEATNGVRFNDLLATL
KTLPARSIVLLHPCCHNPTGADLTNDQWDAVIEILKARELIPFLDIAYQGFGAGMEEDAYAIRAIASAGLPALVSNSFSKIFSLYGE
RVGGLSVMCEDAEAAGRVLGQLKATVRRNYSSPPNFGAQVVAAVLNDEALKASWLAEVEEMRTRILAMRQELVKVLSTEMPE
RNFDYLLNQRGMFSYTGLSAAQVDRLREEFGVYLIASGRMCVAGLNTANVQRVAKAFAAVM

A5: P0AB80

MTTKKADYIWFNGEMVRWEDAKVHVMSHALHYGTSVFEGIRCYDSHKGPVVFRHREHMQRLHDSAKIYRFPVSQSIDELME
ACRDVIRKNNLTSAYIRPLIFVGDVGMGVNPPAGYSTDVIIAAFPWGAYLGAEALEQGIDAMVSSWNRAAPNTIPTAAKAGGNY
LSSLLVGSEARRHGYQEGIALDVNGYISEGAGENLFEVKDGVLFTPPFTSSALPGITRDAIIKLAKELGIEVREQVLSRESLYLADE
VFMSGTAAEITPVRSVDGIQVGEGRCGPVTKRIQQAFFGLFTGETEDKWGWLDQVNQ

A6: Q9ZEU7

MQTQILERMESEVRTYSRSFPTVFTEAKGARLHAEDGNQYIDFLAGAGTLNYGHNHPKLKQALADYIASDGIVHGLDMWSAAK
RDYLETLEEVILKPRGLDYKVHLPGPTGTNAVEAAIRLARNAKGRHNIVTFTNGFHGVTMGALATTGNRKFREATGGIPTQGASF
MPFDGYMGEGVDTLSYFEKLLGDNSGGLDVPAAVIIETVQGEGGINPAGIPWLQRLEKICRDHDMLLIVDDIQAGCGRTGKFF
SFEHAGITPDIVTNSKSLSGFGLPFAHVLMRPELDIWKPGQYNGTFRGFNLAFVTAAAAMRHFWSDDTFERDVQRKGRVVED
RFQKLASFMTEKGHPASERGRGLMRGLDVGDGDMADKITAQAFKNGLIIETSGHSGQVIKCLCPLTITDEDLVGGLDILEQSVK
EVFGQA

A7: P04181

MFSKLAHLQRFAVLSRGVHSSVASATSVATKKTVQGPPTSDDIFEREYKYGAHNYHPLPVALERGKGIYLWDVEGRKYFDFLSS
YSAVNQGHCHPKIVNALKSQVDKLTLTSRAFYNNVLGEYEEYITKLFNYHKVLPMNTGVEAGETACKLARKWGYTVKGIQKYKA
KIVFAAGNFWGRTLSAISSSTDPTSYDGFGPFMPGFDIIPYNDLPALERALQDPNVAAFMVEPIQGEAGVVVPDPGYLMGVREL
CTRHQVLFIADEIQTGLARTGRWLAVDYENVRPDIVLLGKALSGGLYPVSAVLCDDDIMLTIKPGEHGSTYGGNPLGCRVAIAAL
EVLEEENLAENADKLGIILRNELMKLPSDVVTAVRGKGLLNAIVIKETKDWDAWKVCLRLRDNGLLAKPTHGDIIRFAPPLVIKED
ELRESIEIINKTILSF

A8: O32148

MSGRRELCTPLRTIMTPGPVEVDPRVLRVMSTPVVGQFDPAFTGIMNETMEMLRELFQTKNRWAYPIDGTSRAGIEAVLASVIE
PEDDVLIPIYGRFGYLLTEIAERYGANVHMLECEWGTVFDPEDIIREIKKVKPKIVAMVHGETSTGRIHPLKAIGEACRTEDALFIVD
AVATIGGCEVKVDEWKIDAAIGGTQKCLSVPSGMAPITYNERVADVIAARKKVERGIATQADRAALSGNRPITSNYFDLSQLEDY
WSERRLNHHTEATTMLYALREGVRLVLEEGLETRFERHRHHEAALAAGIKAMGLRLFGDDSCKMPVVTCVEIPGGIDGESVRD
MLLAQFGIEIASSFGPLAGKIWRIGTMGYSCRKENVLFVLAGLEAVLLRHNAGIEAGKALQAALDVYENAGRQAAV

A9: Q9X2A5

MYLMNTYSRFPATFVYGKGSWIYDEKGNAYLDFTSGIAVNVLGHSHPRLVEAIKDQAEKLIHCSNLFWNRPQMELAELLSKNTF
GGKVFFANTGTEANEAAIKIARKYGKKKSEKKYRILSAHNSFHGRTLGSLTATGQPKYQKPFEPLVPGFEYFEFNNVEDLRRKMS
EDVCAVFLEPIQGESGIVPATKEFLEEARKLCDEYDALLVFDEVQCGMGRTGKLFAYQKYGVVPDVLTTAKGLGGGVPIGAVIVN
ERANVLEPGDHGTTFGGNPLACRAGVTVIKELTKEGFLEEVEEKGNYLMKKLQEMKEEYDVVADVRGMGLMIGIQFREEVSNR
EVATKCFENKLLVVPAGNNTIRFLPPLTVEYGEIDLAVETLKKVLQGI

A10: Q9X0Y2

MVSRRISEIPISKTMELDAKAKALIKKGEDVINLTAGEPDFPTPEPVVEEAVRFLQKGEVKYTDPRGIYELREGIAKRIGERYKKDIS
PDQVVVTNGAKQALFNAFMALLDPGDEVIVFSPVWVSYIPQIILAGGTVNVVETFMSKNFQPSLEEVEGLLVGKTKAVLINSPN
NPTGVVYRREFLEGLVRLAKKRNFYIISDEVYDSLVYTDEFTSILDVSEGFDRIVYINGFSKSHSMTGWRVGYLISSEKVATAVSKI
QSHTTSCINTVAQYAALKALEVDNSYMVQTFKERKNFVVERLKKMGVKFVEPEGAFYLFFKVRGDDVKFCERLLEEKKVALVPG
SAFLKPGFVRLSFATSIERLTEALDRIEDFLNSR

A11: P53555

MTHDLIEKSKKHLWLPFTQMKDYDENPLIIESGTGIKVKDINGKEYYDGFSSVWLNVHGHRKKELDDAIKKQLGKIAHSTLLGM
TNVPATQLAETLIDISPKKLTRVFYSDSGAEAMEIALKMAFQYWKNIGKPEKQKFIAMKNGYHGDTIGAVSVGSIELFHHVYGPL
MFESYKAPIPYVYRSESGDPDECRDQCLRELAQLLEEHHEEIAALSIESMVQGASGMIVMPEGYLAGVRELCTTYDVLMIVDEV
ATGFGRTGKMFACEHENVQPDLMAAGKGITGGYLPIAVTFATEDIYKAFYDDYENLKTFFHGHSYTGNQLGCAVALENLALFES
ENIVEQVAEKSKKLHFLLQDLHALPHVGDIRQLGFMCGAELVRSKETKEPYPADRRIGYKVSLKMRELGMLTRPLGDVIAFLPPL
ASTAEELSEMVAIMKQAIHEVTSLED

A12: P40732

MATEQTAITRATFDEVILPVYAPADFIPVKGKGSRVWDQQGKEYIDFAGGIAVTALGHCHPALVEALKSQGETLWHTSNVFTNEP
ALRLGRKLIDATFAERVLFMNSGTEANETAFKLARHYACVRHSPFKTKIIAFHNAFHGRSLFTVSVGGQPKYSDGFGPKPADIIH
VPFNDLHAVKAVMDDHTCAVVVEPIQGEGGVQAATPEFLKGLRDLCDEHQALLVFDEVQCGMGRTGDLFAYMHYGVTPDILT
SAKALGGGFPVSAMLTTQEIASAFHVGSHGSTYGGNPLACAVAGAAFDIINTPEVLQGIHTKRQQFVQHLQAIDEQFDIFSDIR
GMGLLIGAELKPKYKGRARDFLYAGAEAGVMVLNAGADVMRFAPSLVVEEADIHEGMQRFAQAVGKVVA

B1: Q56232

MRGLSRRVQAMKPSATVAVNAKALELRRQGVDLVALTAGEPDFDTPEHVKEAARRALAQGKTKYAPPAGIPELREALAEKFRRE
NGLSVTPEETIVTVGGKQALFNLFQAILDPGDEVIVLSPYWVSYPEMVRFAGGVVVEVETLPEEGFVPDPERVRRAITPRTKALVV
NSPNNPTGAVYPKEVLEALARLAVEHDFYLVSDEIYEHLLYEGEHFSPGRVAPEHTLTVNGAAKAFAMTGWRIGYACGPKEVIKA
MASVSSQSTTSPDTIAQWATLEALTNQEASRAFVEMAREAYRRRRDLLLEGLTALGLKAVRPSGAFYVLMDTSPIAPDEVRAAER
LLEAGVAVVPGTDFAAFGHVRLSYATSEENLRKALERFARVLGRA

B2: Q9KM65

MNKPQLPDFIQNKIDHYIENYFDINKNGKHLVLGKQASPDDIILQSNDYLALANHPLIKARLAKSLLEEQQSLFMSASFLQNDY
DKPMIEKRLAKFTGFDECLLSQSGWNANVGLLQTICQPNTNVYIDFFAHMSLWEGARYANAQAHPFMHNNCDHLRMLIQR
HGPGIIVVDSIYSTLGTIAPLAELVNISKEFGCALLVDESHSLGTHGPNGAGLLAELGLTREVHFMTASLAKTFAYRAGAIWCNNE
VNRCVPFISYPAIFSSTLLPYEAAGLETTLEIIESADNRRQHLDRMARKLRIGLSQLGLTIRSESQIIGLETGDERNTEKVRDYLESN
GVFGSVFCRPATSKNKNIIRLSLNSDVNDEQIAKIIEVCSDAVNYGDFYFR

B3: Q5SHH5

METRTLEDWRALLEAEKTLDSGVYNKHDLLIVRGQGARVWDAEGNEYIDCVGGYGVANLGHGNPEVVEAVKRQAETLMAMP
QTLPTPMRGEFYRTLTAILPPELNRVFPVNSGTEANEAALKFARAHTGRKKFVAAMRGFSGRTMGSLSVTWEPKYREPFLPLVEP
VEFIPYNDVEALKRAVDEETAAVILEPVQGEGGVRPATPEFLRAAREITQEKGALLILDEIQTGMGRTGKRFAFEHFGIVPDILTLAK
ALGGGVPLGAAVMREEVARSMPKGGHGTTFGGNPLAMAAGVAAIRYLERTRLWERAAELGPWFMEKLRAIPSPKIREVRGMG
LMVGLELKEKAAPYIARLEKEHRVLALQAGPTVIRFLPPLVIEKEDLERVVEAVRAVLA

B4: Q9X0D0

MNPLDLIAKRAYPYETEKRDKTYLALNENPFPFPEDLVDEVFRRLNSDALRIYYDSPDEELIEKILSYLDTDFLSKNNVSVGNGAD
EIIYVMMLMFDRSVFFPPTYSCYRIFAKAVGAKFLEVPLTKDLRIPEVNVGEGDVVFIPNPNNPTGHVFEREEIERILKTGAFVALD

EAYYEFHGESYVDFLKKYENLAVIRTFSKAFSLAAQRVGYVVASEKFIDAYNRVRLPFNVSYVSQMFAKVALDHREIFEERTKFIVE
ERERMKSALREMGYRITDSRGNFVFVFMEKEEKERLLEHLRTKNVAVRSFREGVRITIGKREENDMILRELEVFK

B5: P9WML5

MTARLRPELAGLPVYVPGKTVPGAIKLASNETVFGPLPSVRAAIDRATDTVNRYPDNGCVQLKAALARHLGPDFAPEHVAVGC
GSVSLCQQLVQVTASVGDEVVFGWRSFELYPPQVRVAGAIPIQVPLTDHTFDLYAMLATVTDRTRLIFVCNPNNPTSTVVGPDA
LARFVEAVPAHILIAIDEAYVEYIRDGMRPDSLGLVRAHNNVVVLRTFSKAYGLAGLRIGYAIGHPDVITALDKVYVPFTVSSIGQA
AAIASLDAADELLARTDTVVAERARVSAELRAAGFTLPPSQANFVWLPLGSRTQDFVEQAADARIVVRPYGTDGVRVTVAAPEE
NDAFLRFARRWRSDQ

B6: O07566

MQKQVKISGKSKENMSLLKHLKGDVQGKELVIEDSIVNERWKQVLKEKIDIEHDLFNYQKNREISKVPFLPVDRLITNDEVEDIL
NTLTEVLPTGKFTSGPYLEQFEKVLSTYLHKRYVIATSSGTDAIMIGLLALGLNPGDEVIMPANSFSATENAVLASGGVPIYVDINP
QTFCIDPDKIEEAITPYTKFILPVHLYGKHSDMQHIRQIANRYKLKVIEDACQGIGLTDLGKYADITTLSFNPYKNFGVCGKAGAIAT
DNEELAKKCIQFSYHGFEVNVKNKKVINFGFNSKMDNLQAAIGLERMKYLSLNNFKRLFLADRYITQLAELQNKGYIELPELSE
DHVWHLFPIKVRTEDRADIMTKLNEDFGVQTDVYYPILSHMQKTPLVQDKYAGLQLVHTEKAHSQVLHLPLYPSFTLEEQDRV
MEGLFHVIKQEIGV

B7: D2D3B2

MANGTRQKDLRERAERVIPGGMYGHESTRLLPPEFPQFFRRALGARIWDADEQPYIDYMCAYGPNLLGYRQSEIEAAADAQRL
LGDTMTGPSEIMVNLAEAFVGMVRHADWAMFCKNGSDATSTAMVLARAHTGRKTILCAKGAYHGASPWNTPHTAGILASDRV
HVAYYTYNDAQSLSDAFKAHDGDIAAVFATPFRHEVFEDQALAQLEFARTARKCCDETGALLVVDDVRAGFRVARDCSWTHL
GIEPDLSCWGKCFANGYPISALLGSNKARDAARDIFVTGSFWFSAVPMAAAIETLRIIRETPYLETLIASGAALRAGLEAQSQRH
GLELKQTGPAQMPQIFFADDPDFRIGYAWAAACLKGGVYVHPYHNMFLSAAHTVDDVTETLEATDRAFSAVLRDFASLQPHPI
LMQLAGA

B8: Q9I700

MNQPLNVAPPVSSELNLRAHWMPFSANRNFQKDPRIIVAAEGSWLTDDKGRKVYDSLSGLWTCGAGHSRKEIQEAVARQLG
TLDYSPGFQYGHPLSFQLAEKIAGLLPGELNHVFFTGSGSECADTSIKMARAYWRLKGQPQKTKLIGRARGYHGVNVAGTSLG
GIGGNRKMFGQLMDVDHLPHTLQPGMAFTRGMAQTGGVELANELLKLIELHDASNIAAVIVEPMSGSAGVLVPPVGYLQRLR
EICDQHNILLIFDEVITAFGRLGTYSGAEYFGVTPDLMNVAKQVTNGAVPMGAVIASSEIYDTFMNQALPEHAVEFSHGYTYSAH
PVACAAGLAALDILARDNLVQQSAELAPHFEKGLHGLQGAKNVIDIRNCGLAGAIQIAPRDGDPTVRPFEAGMKLWQQGFYV
RFGGDTLQFGPTFNARPEELDRLFDAVGEALNGIA

B9: Q9A9H3

MSDLPRISVAAPRLDGNERDYVLECMDTTWISSVGRFIVEFEKAFADYCGVKHAIACNNGTTALHLALVAMGIGPGDEVIVPSLT
YIASANSVTYCGATPVLVDNDPRTFNLDAAKLEALITPRTKAIMPVHLYGQICDMDPILEVARRHNLLVIEDAAEAVGATYRGKKS
GSLGDCATFSFFGNKIITTGEGGMITTNDDDLAAKMRLLRGQGMDPNRRYWFPIVGFNYRMTNIQAAIGLAQLERVDEHLAAR
ERVVGWYEQKLARLGNRVTKPHVALTGRHVFWMYTVRLGEGLSTTRDQVIKDLDALGIESRPVFHPMHIMPPYAHLATDDLKI
AEACGVDGLNLPTHAGLTEADIDRVIAALDQVLV

B10: P42588

MNRLPSSASALACSAHALNLIEKRTLDHEEMKALNREVIEYFKEHVNPGFLEYRKSVTAGGDYGAVEWQAGSLNTLVDTQGQE
FIDCLGGFGIFNVGHRNPVVVSAVQNQLAKQPLHSQELLDPLRAMLAKTLAALTPGKLKYSFFCNSGTESVEAALKLAKAYQSP
RGKFTFIATSGAFHGKSLGALSATAKSTFRKPFMPLLPGFRHVPFGNIEAMRTALNECKKTGDDVAAVILEPIQGEGGVILPPPGY
LTAVRKLCDEFGALMILDEVQTGMGRTGKMFACEHENVQPDILCLAKALGGGVMPIGATIATEEVFSVLFDNPFLHTTTFGGNP
LACAAALATINVLLEQNLPAQAEQKGDMLLDGFRQLAREYPDLVQEARGKGMLMAIEFVDNEIGYNFASEMFRQRVLVAGTLN
NAKTIRIEPPLTLTIEQCELVIKAARKALAAMRVSVEEA

B11: P22256

MNSNKELMQRRSQAIPRGVGQIHPIFADRAENCRVWDVEGREYLDFAGGIAVLNTGHLHPKVVAAVEAQLKKLSHTCFQVLA
YEPYLELCEIMNQKVPGDFAKKTLLVTTGSEAVENAVKIARAATKRSGTIAFSGAYHGRTHYTLALTGKVNPYSAGMGLMPGHVY

RALYPCPLHGISEDDAIASIHRIFKNDAAPEDIAAIVIEPVQGEGGFYASSPAFMQRLRALCDEHGIMLIADEVQSGAGRTGTLFA
MEQMGVAPDLTTFAKSIAGGFPLAGVTGRAEVMDAVAPGGLGGTYAGNPIACVAALEVLKVFEQENLLQKANDLGQKLKDGLL
AIAEKHPEIGDVRGLGAMIAIELFEDGDHNKPDAKLTAEIVARARDKGLILLSCGPYYNVLRILVPLTIEDAQIRQGLEIISQCFDEA
KQ

B12: P12995

MTTDDLAFDQRHIWHPYTSMTSPLPVYPVVSAEGCELILSDGRRLVDGMSSWWAAIHGYNHPQLNAAMKSQIDAMSHVMF
GGITHAPAIELCRKLVAMTPQPLECVFLADSGSVAVEVAMKMALQYWQAKGEARQRFLTFRNGYHGDTFGAMSVCDPDNSM
HSLWKGYLPENLFAPAPQSRMDGEWDERDMVGFARLMAAHRHEIAAVIIEPIVQGAGGMRMYHPEWLKRIRKICDREGILLIA
DEIATGFGRTGKLFACEHAEIAPDILCLGKALTGGTMTLSATLTTREVAETISNGEAGCFMHGPTFMGNPLACAAANASLAILESG
DWQQQVADIEVQLREQLAPARDAEMVADVRVLGAIGVVETTHPVNMAALQKFFVEQGVWIRPFGKLIYLMPPYIILPQQLQRL
TAAVNRAVQDETFFCQ

C1: Q9ZQI7

MVSLMFFSSASPLCSSPSKIPKASLDFEMKKLGGSTKLVRNVNLEKLKNNYLFPEINRRELEHIEKHPNVQLISLGTGDTTEPIPE
QITSHMSNFAHGLSTVEGYRGYGLEQGNKTLRKAIAETFYRDLHVKSNEVFVSDGAQSDISRLQLLLGSNVTIAVQDPTFPAYID
SSVIIGQTGHFHEKTKKYQNVVYMPCGPNNSFFPDLAMTPRTDVIFFCSPNNPTGYVASRKQLHQLVDFAKTNGSIIIFDSAYAA
FIEDGSPRSIYEIPGAREVAIEVSSFSKFAGFTGVRLGWSIIPDELLYSNGFPIINDFHRIVTTSFNGASNIAQAGGLACLSSGGLKE
IRSVNNYYKENRKILMDTLVSLGLKVYGGVNAPYLWVHFKGSKSWDVFNEILENTHIITVPGSGFGPGGEEYLRISGFGRRDHIV
EASKRLQNFFNTRTKHFTYLSSTSNTN

C2: H8WR05

MTHAAIDQALADAYRRFTDANPASQRQFEAQARYMPGANSRSVLFYAPFPLTIARGEGAALWDADGHRYADFIAEYTAGVYGH
SAPEIRDAVIEAMQGGINLTGHNLLEGRLARLICERFPQIEQLRFTNSGTEANLMALTAALHFTGRRKIVVFSGGYHGGVLGFGA
RPSPTTVPFDFLVLPYNDAQTARAQIERHGPEIAVVLVEPMQGASGCIPGQPDFLQALRESATQVGALLVFDEVMTSRLAPHGL
ANKLGIRSDLTTLGKYIGGGMSFGAFGGRADVMALFDPRTGPLAHSGTFNNNVMTMAAGYAGLTKLFTPEAAGALAERGEALR
ARLNALCANEGVAMQFTGIGSLMNAHFVQGDVRSSEDLAAVDGRLRQLLFFHLLNEDIYSSPRGFVVLSLPLTDADIDRYVAAI
GSFIGGHGALLPRAN

C3: Q988B8

MMRYPEHADPVITLTAGPVNAYPEVLRGLGRTVLYDYDPAFQLLYEKVVDKAQKAMRLSNKPVILHGEPVLGLEAAAASLISPDD
VVLNLASGVYGKGFGYWAKRYSPHLLEIEVPYNEAIDPQAVADMLKAHPEITVVSVCHHDTPSGTINPIDAIGALVSAHGAYLIV
DAVSSFGGMKTHPEDCKADIYVTGPNKCLGAPPGLTMMGVSERAWAKMKANPLAPRASMLSIVDWENAWSRDKPFPFTPSV
SEINGLDVALDLYLNEGPEAVWARHALTAKAMRAGVTAMGLSVWAASDSIASPTTTAVRTPDGVDEKALRQAARARYGVVFSS
GRGETLGKLTRIGHMGPTAQPIYAIAALTALGGAMNAAGRKLAIGKGIEAALAVIDADA

C4: Q72LL6

MKPLSWSEAFGKGAGRIQASTIRELLKLTQRPGILSFAGGLPAPELFPKEEAAEAAARILREKGEVALQYSPTEGYAPLRAFVAEWI
GVRPEEVLITTGSQQALDLVGKVFLDEGSPVLLEAPSYMGAIQAFRLQGPRFLTVPAGEEGPDLDALEEVLKRERPRFLYLIPSFQ
NPTGGLTPLPARKRLLQMVMERGLVVVEDDAYRELYFGEARLPSLFELAREAGYPGVIYLGSFSKVLSPGLRVAFAVAHPEALQK
LVQAKQGADLHTPMLNQMLVHELLKEGFSERLERVRRVYREKAQAMLHALDREVPKEVRYTRPKGGMFVWMELPKGLSAEG
LFRRALEENVAFVPGGPFFANGGGENTLRLSYATLDREGIAEGVRRLGRALKGLLALV

C5: Q8TD30

MQRAAALVRRGCGPRTPSSWGRSQSSAAAEASAVLKVRPERSRRERILTLESMNPQVKAVEYAVRGPIVLKAGEIELELQRGIK
KPFTEVIRANIGDAQAMGQQPITFLRQVMALCTYPNLLDSPSFPEDAKKRARRILQACGGNSLGSYSASQGVNCIREDVAAYIT
RRDGGVPADPDNIYLTTGASDGISTILKILVSGGGKSRTGVMIPIPQYPLYSAVISELDAIQVNYYLDEENCWALNVNELRRAVQE
AKDHCDPKVLCIINPGNPTGQVQSRKCIEDVIHFAWEEKLFLLADEVYQDNVYSPDCRFHSFKKVLYEMGPEYSSNVELASFH
STSKGYMGECGYRGGYMEVINLHPEIKGQLVKLLSVRLCPPVSGQAAMDIVVNPPVAGEESFEQFSREKESVLGNLAKKAKLTE
DLFNQVPGIHCNPLQGAMYAFPRIFIPAKAVEAAQAHQMAPDMFYCMKLLEETGICVVPGSGFGQREGTYHFRMTILPPVEKL
KTVLQKVKDFHINFLEKYA

C6: A0A0J9X1Q5

MKFGFIAHPTSLGLKRYVKMLDLLQRNSTEQHSGYTRELWERQNLVPFMNFARITSATGATCEGVIKYMPLVADEMLADARGIA
ARVVQGIEELAGDGAELVGLGGFTSIVGRRGEATAEKSPVPVTSGNSLTTYAGYKALMQIQSWLEIRPEEEPVAIVGYPGSICLAL
SRLLLAHGFSLHLLHRAGNHDRSELLSHLPEEYHSRVTLTSDPEDLYPRCKLFAAATSAGGVIDPARLQPGSIFIDVALPRDIASE
TRPARDDILIIDGGCVTATDAVKLGGESLNVTIKQQLNGCMAETIVLALENRRENFSLGRYLAPEKVLEIGEIAERHGFFAYPLASY
GERIDRQSVTNLKRYYHHDIYAGESADAALPASRLAFIDAVIAQTPAREDTLDRYHQYINPMMVDFLKLQRCDNVFRSAAGTQL
YDDAGEAFLDMVAGYGCLNLGHNPQPVVNALKNYLDAQGPNFIQYISIPEQTAKLAEVLCRLAPGNMGRVFFSNSGTEAVEA
AMKIAKASTGKPGIAYLRNSYHGKTLGALSITGRDKHRRYFTPLLDAMVEVPFGDLAALREALNREDVGALMIEPIQGEGGVHIP
PAGYLQAVQQLCRETGVLLMVDEVQTGLGRTGKLFACEWDGIEPDVLMLSKSLSGGLIPIGATLCRADLWQKAYGTADRFLVH
SSTYGGGNLASVVALSALREILAQDLVGHAERMGAYFKQALSEIAARYPFVSEVRGRGLMLGIQFDQAFTGAVNASAREFATRL
PGDWHTTWKFLPDPVQAHLRAAMDRMEQALGEMFCMKFVTKLCQDHKILTFITANSSTVIRIQPPLIISKAEIDRFVGAFATVCE
ELSTFLD

C7: B0F481

MIPVTATLIRHRLRHLRHRIRFKSTSVSPFHLPLNHPTYLIWSANTSLGKTLVSTGIAASFLLQQPSSSATKLLYLKPIQTGFPSDSD
SRFVFSKLDSLSLRRQIPISISNSVLHSSLPAAKSLGLNVEVSESGMCSLNFRDEKTVTGAPELLCKTLYAWEAAISPHLAAEREN
ATVEDSVVLQMIEKCLKEEMECGVKSEKSDLLCLVETAGGVASPGPSGTLQCDLYRPFRLPGILVGDGRLGGISGTIAAYESLKL
RGYDIAAVVFEDHGLVNEVPLTSYLRNKVPVLVLPPVPKDPSDDLIEWFVESDGVFKALKETMVLANLERLERLNGMAKLAGEV
FWWPFTQHKLVHQETVTVIDSRCGENFSIYKASDNSSLSQQFDACASWWTQGPDPTFQAELAREMGYTAARFGHVMFPEN
VYEPALKCAELLLDGVGKGWASRVYFSDNGSTAIEIALKMAFRKFCVDHNFCEATEEEKHIVVKVIALRGSYHGDTLGAMEAQA
PSPYTGFLQQPWYTGRGLFLDPPTVFLSNGSWNISLPESFSEIAPEYGTFTSRDEIFDKSRDASTLARIYSAYLSKHLQEHSGVR
QSAHVGALIIEPVIHGAGGMHMVDPLFQRVLVNECRNRKIPVIFDEVFTGFWRLGVETTTELLGCKPDIACFAKLLTGGMVPLA
VTLATDAVFDSFSGDSKLKALLHGHSYSAHAMGCATAAKAIQWFKDPETNHNITSQGKTLRELWDEELVQQISSHSAVQRVVV
IGTLFALELKADASNSGYASLYAKSLLIMLREDGIFTRPLGNVIYLMCGPCTSPEICRRLLTKLYKRLGEFNRT

C8: P19938

MGYTLWNDQIVKDEEVKIDKEDRGYQFGDGVYEVVKVYNGEMFTVNEHIDRLYASAEKIRITIPYTKDKFHQLLHELVEKNELNT
GHIYFQVTRGTSPRAHQFPENTVKPVIIGYTKENPRPLENLEKGVKATFVEDIRWLRCDIKSLNLLGAVLAKQEAHEKGCYEAILH
RNNTVTEGSSSNVFGIKDGILYTHPANNMILKGITRDVVIACANEINMPVKEIPFTTHEALKMDELFVTSTTSEITPVIEIDGKLIRD
GKVGEWTRKLQKQFETKIPKPLHI

C9: P96060

MTSRNYLLLTPGPLTTSRTVKEAMLFDSCTWDDDYNIGVVEQIRQQLTALATASEGYTSVLLQGSGSYAVEAVLGSALGPQDKVL
IVSNGAYGARMVEMAGLMGIAHHAYDCGEVARPDVQAIDAILNADPTISHIAMVHSETTTGMLNPIDEVGALAHRYGKTYIVDA
MSSFGGIPMDIAALHIDYLISSANKCIQGVPGFAFVIAREQKLAACKGHSRSLSLDLYAQWRCMEDNHGKWRFTSPTHTVLAF
AQALKELAKEGGVAARHQRYQQNQRSLVAGMRALGFNTLLDDELHSPIITAFYSPEDPQYRFSEFYRRLKEQGFVIYPGKVSQS
DCFRIGNIGEVYAADITALLTAIRTAMYWTK

C10: P53090

MTLPESKDFSYLFSDETNARKPSPLKTCIHLFQDPNIIFLGGGLPLKDYFPWDNLSVDSPKPPFPQGIGAPIDEQNCIKYTVNKD
YADKSANPSNDIPLSRALQYGFSAGQPELLNFIRDHTKIIHDLKYKDWDVLATAGNTNAWESTLRVFCNRGDVILVEAHSFSSS
LASAEAQGVITFPVPIDADGIIPEKLAKVMENWTPGAPKPKLLYTIPTGQNPTGTSIADHRKEAIYKIAQKYDFLIVEDEPYYFLQM
NPYIKDLKEREKAQSSPKQDHDEFLKSLANTFLSLDTEGRVIRMDSFSKVLAPGTRLGWITGSSKILKPYLSLHEMTIQAPAGFTQ
VLVNATLSRWGQKGYLDWLLGLRHEYTLKRDCAIDALYKYLPQSDAFVINPPIAGMFFTVNIDASVHPEFKTKYNSDPYQLEQS
LYHKVVERGVLVVPGSWFKSEGETEPPQPAESKEVSNPNIIFFRGTYAAVSPEKLTEGLKRLGDTLYEEFGISK

C11: P0A959

MSPIEKSSKLENVCYDIRGPVLKEAKRLEEEGNKVLKLNIGNPAPFGFDAPDEILVDVIRNLPTAQGYCDSKGLYSARKAIMQHY
QARGMRDVTVEDIYIGNGVSELIVQAMQALLNSGDEMLVPAPDYPLWTAAVSLSSGKAVHYLCDESSDWFPDLDDIRAKITPR
TRGIVIINPNNPTGAVYSKELLMEIVEIARQHNLIIFADEIYDKILYDDAEHHSIAPLAPDLLTITFNGLSKTYRVAGFRQGWMVLNG

PKKHAKGYIEGLEMLASMRLCANVPAQHAIQTALGGYQSISEFITPGGRLYEQRNRAWELINDIPGVSCVKPRGALYMFPKIDAK
RFNIHDDQKMVLDFLLQEKVLLVQGTAFNWPWPDHFRIVTLPRVDDIELSLSKFARFLSGYHQL

C12: Q39YP6
MIPLRQNIASMKGYIPGYQPPDIASWIKLNTNENPYPPSPEVVKAILEELGPDGAALRIYPSASSQKLREVAGELYGFDPSWIIMA
NGSDEVLNNLIRAFAAEGEEIGYVHPSYSYYGTLAEVQGARVRTFGLTGDFRIAGFPERYEGKVFFLTTPNAPLGPSFPLEYIDEL
ARRCAGMLVLDETYAEFAESNALELVRRHENVVVTRTLSKSYSLAGMRIGLAIARPEVIAALDKIRDHYNLDRLAQAACVAALRD
QAYLSECCRRIRETREWFTTELRSIGYDVIPSQGNYLFATPPDRDGKRVYDGLYARKVLVRHFSDPLLAHGMRISIGTREEMEQT
LAALKEIG

D1: Q9HZ76
MIEFIDLKNQQARIKDKIDAGIQRVLRHGQYILGPEVTELEDRLADFVGAKYCISCANGTDALQIVQMALGVGPGDEVITPGFTY
VATAETVALLGAKPVYVDIDPRTYNLDPQLLEAAITPRTKAIIPVSLYGQCADFDAINAIASKYGIPVIEDAAQSFGASYKGKRSCNL
STVACTSFFPSKPLGCYGDGGAIFTNDDELATAIRQIARHGQDRRYHHIRVGVNSRLDTLQAAILLPKLEIFEEEIALRQKVAAEY
DLSLKQVGIGTPFIEVNNISVYAQYTVRMDNRESVQASLKAAGVPTAVHYPIPLNKQPAVADEKAKLPVGDKAATQVMSLPMHP
YLDTASIKIICAALTN

D2: Q4R0W2
MVSQLAVKGGEALRTRPWPAWPQPAPGVPDAVADVLGSGRWSISGPYRGTESYERRFARAFAAYNGVPHCVPAASGTASLM
LALEACGIGAGDEVIVPGLSWVASGSTILGVNAVPIFCDVDPDTLCLSPEAVEAAITEHTRAIVVVHLYSALADMDALSAIAERHG
LPLIEDCAQAHGATYRGVKVGALATAGTFSMQHSKVLTSGEGGAVITRDEDFARRVEHLRADGRCLSAVPPAPGAMELVETGEL
MGNNRCLSEFQAAILAEQLTILDEQNETRRANAAHLDGLLGELGLRPQTTSDGTTSRTYYTYAVRLPDGVLEDVPVTDVSCALT
AELGFPVLPSYAPIPANRLYTPHTRRRYTLGLDHERRIDPKRFALPVCEDAARRTVTLHHAALLGDADDMGDIAAAFAKVLRHGA
GLMH

D3: P77581
MSQPITRENFDEWMIPVYAPAPFIPVRGEGSRLWDQQGKEYIDFAGGIAVNALGHAHPELREALNEQASKFWHTGNGYTNEP
VLRLAKKLIDATFADRVFFCNSGAEANEAALKLARKFAHDRYGSHKSGIVAFKNAFHGRTLFTVSAGGQPAYSQDFAPLPADIR
HAAYNDINSASALIDDSTCAVIVEPIQGEGGVVPASNAFLQGLRELCNRHNALLIFDEVQTGVGRTGELYAYMHYGVTPDLLTTA
KALGGGFPVGALLATEECARVMTVGTHGTTYGGNPLASAVAGKVLELINTPEMLNGVKQRHDWFVERLNTINHRYGLFSEVRG
LGLLIGCVLNADYAGQAKQISQEAAKAGVMVLIAGGNVVRFAPALNVSEEEVTTGLDRFAAACEHFVSRGSS

D4: Q8ZNF3
MAEGKMMSDFLPFSRPAMGAEELAAVKTVLDSGWITTGPKNQELEAAFCRLTGNQYAVAVSSATAGMHIALMALGIGEGDEVI
TPSMTWVSTLNMIVLLGANPVMVDVDRDTLMVTPEHIEAAITPQTKAIIPVHYAGAPADLDAIYALGERYGIPVIEDAAHATGTSYK
GRHIGARGTAIFSFHAIKNITCAEGGIVVTDNPQFADKLRSLKFHGLGVDAWDRQSGGRAPQAEVLAPGYKYNLPDLNAAIALA
QLQKLDALNARRAAIAAQYHQAMADLPFQPLSLPSWEHIHAWHLFIIRVDEARCGITRDALMASLKTKGIGTGLHFRAAHTQKY
YRERFPTLTLPDTEWNSERICSLPLFPDMTESDFDRVITALHQIAGQ

D5: O84395
MKRNPHFVSLTKNYLFADLQKRVAQFRLENPQHTVINLSIGDTTQPLNASVAEAFASSIARLSSPTTCRGYGPDFGLPALRQKLS
EDFYRGFVDAKEIFISDGAKVDLFRLLSFFGPNQTVAIQDPSYPAYLDIARLTGAKEIIALPCLQENAFFPEFPEDTHIDILCLCSPN
NPTGTVLNKDQLRAIVHYAIEHEILILFDAAYSTFISDPSLPKSIFEIPDARFCAIEINSFSKPLGFAGIRLGWTVIPQELTYADGHFVI
QDWERFLSTTFNGASIPAQEAGVAGLSILPQLEAIHYYRENSDLLRKALLATGFEVFGGEHAPYLWVKPTQANISDRDLFDFFLR
EYHIAITPGIGFGRSGSGFVRFSSLGKREDILAACERLQMAPALQS

D6: P28269
NMPEHAGASLASQLKLDAHWMPYTANRNFLRDPRLIVAAEGSWLVDDKGRKVYDSLSGLWTCGAGHTRKEIQEAVAKQLSTL
DYSPGFQYGHPLSFQLAEKITDLTPGNLNHVFFTDSGSECALTAVKMVRAYWRLKGQATKTKMIGRARGYHGVNIAGTSLGGV
NGNRKLFGQPMQDVDHLPHTLLASNAYSRGMPKEGGIALADELLKLIELHDASNIAAVFVEPLAGSAGVLVPPEGYLKRNREI
CNQHNILLVFDEVITGFGRTGSMFGADSFGVTPDLMCIAKQVTNGAIPMGAVIASTEIYQTFMNQPTPEYAVEFPHGYTYSAHP

VACAAGLAALCLLQKENLVQSVAEVAPHFEKALHGIKGAKNVIDIRNFGLAGAIQIAPRDGDAIVRPFEAGMALWKAGFYVRFG
GDTLQFGPTFNSKPQDLDRLFDAVGEVLNKLLD

D7: Q0P9D3

MRFFLSPPHMGGNELKYIEEVFKSNYIAPLGEFVNRFEQSVKAYSKSENALALNSATAALHLALRVAGVKQDDIVLASSFTFIASV
APICYLKAKPVFIDCDETYNIDVDLLKLAIKECEKKPKALILTHLYGNAAKMDEIVEICKENEIVLIEDAAEALGSFYKNKALGTFGE
FGAYSYNGNKIITTSGGGMLIGKNKEKIEKARFYSTQARENCLHYEHLDYGYNYRLSNVLGAIGVAQMEVLEQRVLKKREIYEWY
KEFLGECFSFLDELENSRSNRWLSTALIDFDKNELNSCQKDINISQKNITLHPKISKLIEDLKNEQIETRPLWKAMHAQEVFKGA
KAYLNGNSELFFQKGICLPSGTAMSKDDVYEISKLILKSIKA

D8: P52894

MAATVAVDNLNPKVLKCEYAVRGEIVIHAQRLQEQLKTQPGSLPFDEILYCNIGNPQSLGQQPVTFFREVLALCDHPDLLQREE
IKTLFSADSISRAKQILAMIPGRATGAYSHSQGIKGLRDAIASGIASRDGFPANADDIFLTDGASPGVHLMMQLLIRNEKDGILVPI
PQYPLYSASIALHGGALVPYYLNESTGWGLETSDVKKQLEDARSRGINVRALVVINPGNPTGQVLAEENQYDIVKFCKNEGLVL
LADEVYQENIYVDNKKFHSFKKIVRSLGYGEEDLPLVSYQSVSKGYYGECGKRGGYFEITGFSAPVREQIYKIASVNLCSNITGQI
LASLVMNPPKASDESYASYKAEKDGILASLARRAKALEHAFNKLEGITCNEAEGAMYVFPQICLPQKAIEAAKAANKAPDAFYAL
RLLESTGIVVVPGSGFGQVPGTWHFRCTILPQEDKIPAVISRFTVFHEAFMSEYRD

D9: Q9RME2

MVKQVFNFNAGPSALPKPALERAQKELLNFNDTQMSVMELSHRSQSYEEVHEQAQNLLRELLQIPNDYQILFLQGGASLQFT
MLPMNLLTKGTIGNYVLTGSWSEKALKEAKLLGETHIAASTKANSYQSIPDFSEFQLNENDAYLHITSNNTIYGTQYQNFPEINHA
PLIADMSSDILSRPLKVNQFGMIYAGAQKNLGPSGVTVVIVKKDLLNTKVEQVPTMLQYATHIKSDSLYNTPPTFSIYMLRNVLD
WIKDLGGAEAIAKQNEEKAKIIYDTIDESNGFYVGHAEKGSRSLMNVTFNLRNEELNQQFLAKAKEQGFVGLNGHRSVGGCR
ASIYNAVPIDACIALRELMIQFKENA

D10: Q56YA5

MDYMYGPGRHHLFVPGPVNIPEPVIRAMNRNNEDYRSPAIPALTKTLLEDVKKIFKTTSGTPFLFPTTGTGAWESALTNTLSPGD
RIVSFLIGQFSLLWIDQQKRLNFNVDVVESDWGQGANLQVLASKLSQDENHTIKAICIVHNETATGVTNDISAVRTLLDHYKHP
ALLLVDGVSSICALDFRMDEWGVDVALTGSQKALSLPTGLGIVCASPKALEATKTSKSLKVFFDWNDYLKFYKLGTYWPYTPSIQ
LLYGLRAALDLIFEEGLENIIARHARLGKATRLAVEAWGLKNCTQKEEWISNTVTAVMVPPHIDGSEIVRRAWQRYNLSLGLGLN
KVAGKVFRIGHLGNVNELQLLGCLAGVEMILKDVGYPVVMGSGVAAASTYLQHHIPLIPSRI

D11: P17735

MDPYMIQMSSKGNLPSILDVHVNVGGRSSVPGKMKGRKARWSVRPSDMAKKTFNPIRAIVDNMKVKPNPNKTMISLSIGDPT
VFGNLPTDPEVTQAMKDALDSGKYNGYAPSIGFLSSREEIASYYHCPEAPLEAKDVILTSGCSQAIDLCLAVLANPGQNILVPRP
GFSLYKTLAESMGIEVKLYNLLPEKSWEIDLKQLEYLIDEKTACLIVNNPSNPCGSVFSKRHLQKILAVAARQCVPILADEIYGDM
VFSDCKYEPLATLSTDVPILSCGGLAKRWLVPGWRLGWILIHDRRDIFGNEIRDGLVKLSQRILGPCTIVQGALKSILCRTPGEFY
HNTLSFLKSNADLCYGALAAIPGLRPVRPSGAMYLMVGIEMEHFPEFENDVEFTERLVAEQSVHCLPATCFEYPNFIRVVITVPE
VMMLEACSRIQEFCEQHYHCAEGSQEECDK

D12: P80147

MASVLLTRRLACSFRHNHRLLVPGWRHISQAAAKVDVEFDYDGPLMKTEVPGPRSRELMKQLNIIQNAEAVHFFCNYEESRG
NYLVDVDGNRMLDLYSQISSIPIGYSHPALVKLVQQPQNVSTFINRPALGILPPENFVEKLRESLLSVAPKGMSQLITMACGSCS
NENAFKTIFMWYRSKERGQSAFSKEELETCMINQAPGCPDYSILSFMGAFHGRTMGCLATTHSKAIHKIDIPSFDWPIAPFPRL
KYPLEEFVKENQQEEARCLEEVEDLIVKYRKKKKTVAGIIVEPIQSEGGDNHASDDFFRKLRDISRKHGCAFLVDEVQTGGGST
GKFWAHEHWGLDDPADVMTFSKKMMTGGFFHKEEFRPNAPYRIFNTWLGDPSKNLLLAEVINIIKREDLLSNAAHAGKVLLT
GLLDLQARYPQFISRVRGRGTFCSFDTPDESIRNKLISIARNKGVMLGGCGDKSIRFRPTLVFRDHHAHLFLNIFSDILADFK

E1: P77806

MTNNPLIPQSKLPQLGTTIFTQMSALAQQHQAINLSQGFPDFDGPRYLQERLAHHVAQGANQYAPMTGVQALREAIAQKTER
LYGYQPDADSDITVTAGATEALYAAITALVRNGDEVICFDPSYDSYAPAIALSGGIVKRMALQPPHFRVDWQEFAALLSERTRLVIL
NTPHNPSATVWQQADFAALWQAIAGHEIFVISDEVYEHINFSQQGHASVLAHPQLRERAVAVSSFGKTYHMTGWKVGYCVAP

APISAEIRKVHQYLTFSVNTPAQLALADMLRAEPEHYLALPDFYRQKRDILVNALNESRLEILPCEGTYFLLVDYSAVSTLDDVEF
CQWLTQEHGVAAIPLSVFCADPFPHKLIRLCFAKKESTLLAAAERLRQL

E2: P74921

MLIWWRGKFRRADEISLDFSLFEKSLQGAVYETLRTYSRAPFAAYKHYTRLKRSADFFNLPLSLSFDEFTKVLKAGADEFKQEVRI
KVYLFPDSGEVLFVFSPLNIPDLETGVEVKISNVRRIPDLSTPPALKITGRTDIVLARREIVDCYDVILLGLNGQVCEGSFSNVFLVK
EGKLITPSLDSGILDGITRENVIKLAKSLEIPVEERVVWVWELFEADEMFLTHTSAGVVPVRRLNEHSFFEEEPGPVTATLMENFE
PFVLNLEENWVGI

E3: P21549

MASHKLLVTPPKALLKPLSIPNQLLLGPGPSNLPPRIMAAGGLQMIGSMSKDMYQIMDEIKEGIQYVFQTRNPLTLVISGSGHC
ALEAALVNVLEPGDSFLVGANGIWGQRAVDIGERIGARVHPMTKDPGGHYTLQEVEEGLAQHKPVLLFLTHGESSTGVLQPLD
GFGELCHRYKCLLLVDSVASLGGTPLYMDRQGIDILYSGSQKALNAPPGTSLISFSDKAKKKMYSRKTKPFSFYLDIKWLANFW
GCDDQPRMYHHTIPVISLYSLRESLALIAEQGLENSWRQHREAAAYLHGRLQALGLQLFVKDPALRLPTVTTVAVPAGYDWRDI
VSYVIDHFDIEIMGGLGPSTGKVLRIGLLGCNATRENVDRVTEALRAALQHCPKKKL

E4: Q53U08

MTKNSSLLAEFPTCPRDEKDRPRVFTAASGAWLTDESGFRWIDFDNARGSILLGHGDPVVAEAVARAATGADGTATGWSRRV
DAVLERLHALCGGEVVGLFRSGTAAVRAAVLAVREATGRPLLLSAGYHGYDPMWYPSEAPLEPNADGVVDFFFDLGLLRELLR
APERVAAVVVSPDHMHLSPGWYRELRRLCSAAGVVLVADEVKVGLRYAPGLSTAELLAPDVWVVAKGMANGHAVSAVGGSR
RLLKPLKEVSFTSFFEPTILAAADAALARVATGEPQRAVREAGDRFLRHARKALDDASLPVEIAGDGTFFQFVPATEELEEALYGA
ANAEGLLFYAGDNQGVSAAFDEAVLGEAERRFARVCERLAPYAGGEPVGDAARYRVAWNVMDGLRQAPRDREETTGLLARLL
DD

E5: O29329

MLYVYMDGEFVPENEAKVSIFDHGFLYGDGVFEGIRAYNGRVFRLKEHIDRLYDSAKAIDLEIPITKEEFMEIILETLRKNNLRDAY
IRPIVTRGIGDLGLDPRKCQNPSIIVITKPWGKLYGDLYEKGLTAITVAVRRNSFDALPPNIKSLNYLNNILAKIEANAKGGDEAIFL
DRNGYVSEGSGDNIFVVKNGAITTPPTINNLRGITREAVIEIINRLGIPFKETNIGLYDLYTADEVFVTGTAAEIAPIVVIDGRKIGDG
KPGEITRKLMEEFSKLTESEGVPIYE

Library Profiling and Analytical-Scale Biocatalytic Reactions

Reactions in 96-Well Plates

Plate Expression

To each well of a 96-well deep-well plate was added 500 µL of LB_{Kan}. Each well was inoculated with 5 µL from a BL21(DE3) *E. coli* glycerol stock of the library. This starter culture plate was incubated at 37 °C at 200 rpm overnight. To make expression cultures, 485 µL of TB_{Kan} was added to each well of a 96-well deep-well plate. The expression culture was inoculated with 5 µL starter culture and incubated at 37 °C at 350 rpm until OD_{600} of ~0.8 was reached. Addition of 10 µL 5 mM IPTG (final concentration: 0.1 mM) into each well induced protein expression, and the expression culture was further incubated at 20 °C at rpm RPM overnight. The expression culture was centrifuged at 900 ×*g* for 30 min and supernatant discard to yield *E. coli* whole-cell pellets. The plates could be used immediately or stored at -80 °C.

Plate Reactions

Lysis: Plates containing pelleted cells (prepared as described above) were lysed by three rounds of freeze-thaw (immersion in liquid nitrogen bath for 1 minute, then thaw in water bath for 10 minutes). Then, the cell pellets were resuspended in 200 µL lysis solution to induce chemical lysis (1 mg/mL lysozyme, 4 mM $MgCl_2$, 3 U/mL DNAse) and incubated at room temperature for 30 min at 350 rpm. The resulting unclarified lysate was used directly for analytical-scale reactions.

Reaction general procedure: A master mix containing amino acid (154 mM), α-ketoglutarate (as disodium salt dihydrate, 154 mM), PLP (154 µM), and potassium phosphate buffer (50 mM, pH 8.0) was prepared in a 50 mL conical tube. To each well of a deep-well 96-well plate was added 50 µL unclarified cell lysate, 130 µL master mix, and 20 µL of electrophile (0.5 M stock in DMSO). Final concentrations: 100 mM amino acid, 100 mM α-ketoglutarate, 50 mM KP_i, 100 µM PLP, 50 mM electrophile, 10% DMSO v/v, 200 µL total reaction volume. The plate was incubated at 30 °C at 350 rpm for 18 h.

Reaction analysis: At the end of the reaction, 1 µL of reaction was diluted 1000X with quench solution (1:1 solution of acetonitrile/0.1% formic acid in water). 200 µL diluted solution was centrifuged at 900 ×*g* through a 96-well filter plate to remove precipitate and the filtrate was collected into a 96-well assay plate. The filtrate was analyzed by LC-MS and contains a theoretical maximum product concentration of 50 µM based on limiting electrophile.

Library Profiling LC-MS Chromatograms

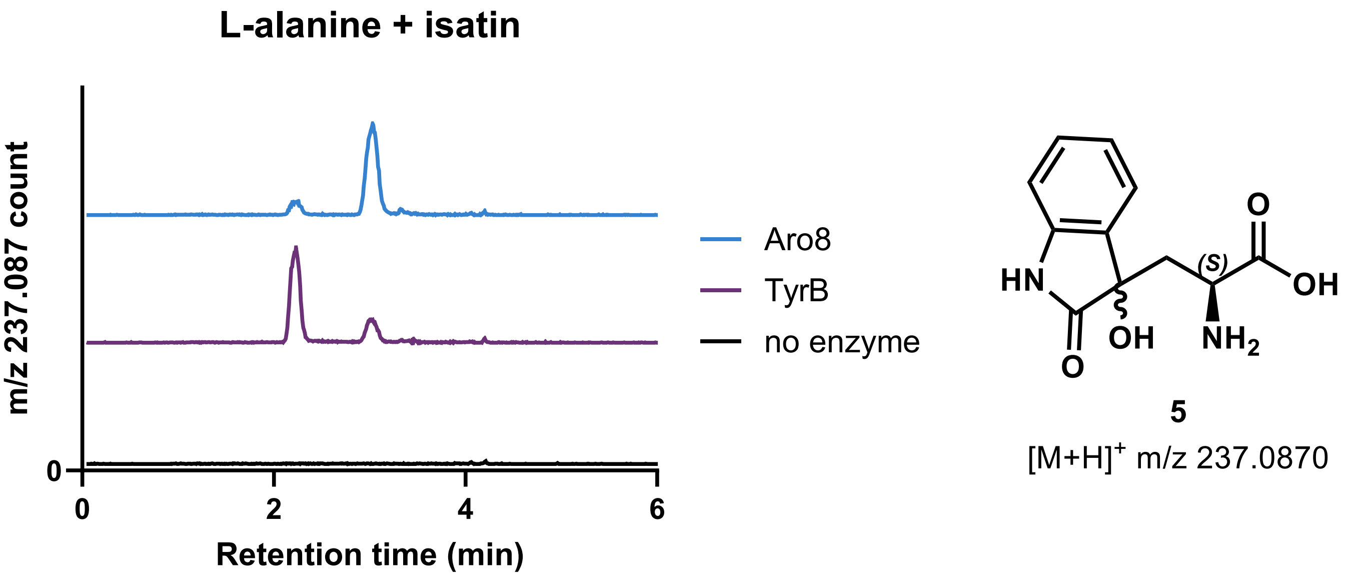

Figure S1. LC-MS chromatograms of Aro8- and TyrB-catalyzed reactions between L-alanine and isatin to form product **5**. Chromatograms are on same scale. Conditions: 50 mM isatin, 100 mM l-alanine, 50 mM KPi pH 8.0, 10% v/v DMSO, 25% v/v lysate, 30 °C, 18 h. Diluted 1:1000 before LC-TOF analysis.

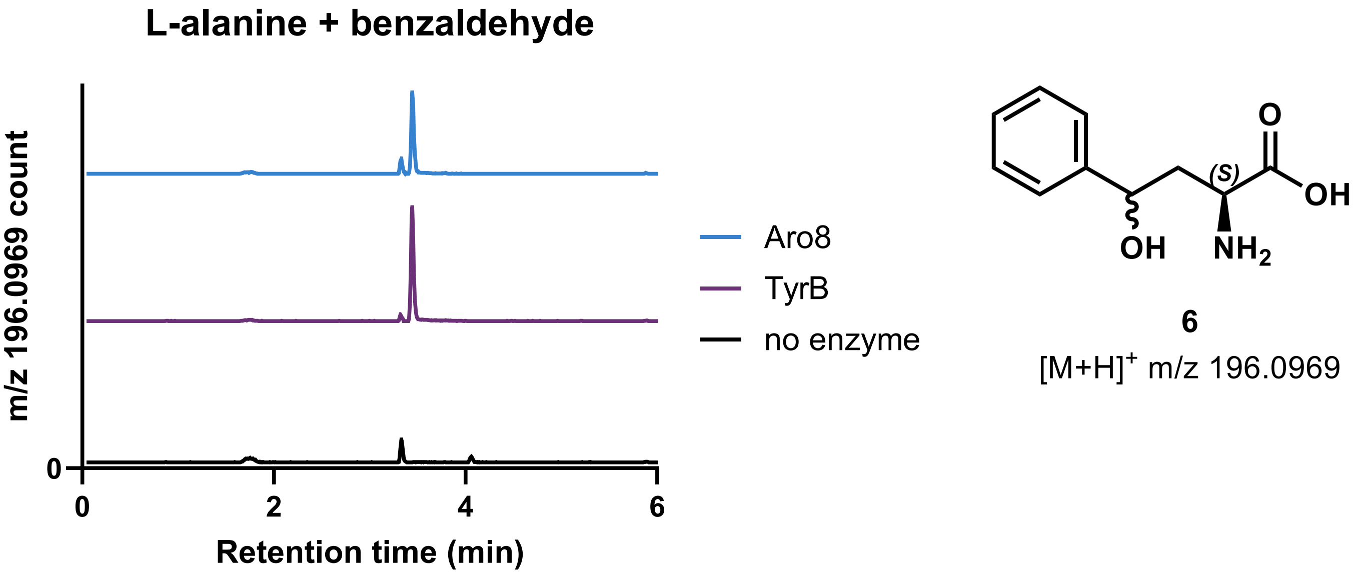

Figure S2. LC-MS chromatograms of Aro8- and TyrB-catalyzed reactions between L-alanine and benzaldehyde to form product **6**. Chromatograms are on same scale. Conditions: 50 mM isatin, 100 mM l-alanine, 50 mM KPi pH 8.0, 10% v/v DMSO, 25% v/v lysate, 30 °C, 18 h. Diluted 1:1000 before LC-TOF analysis.

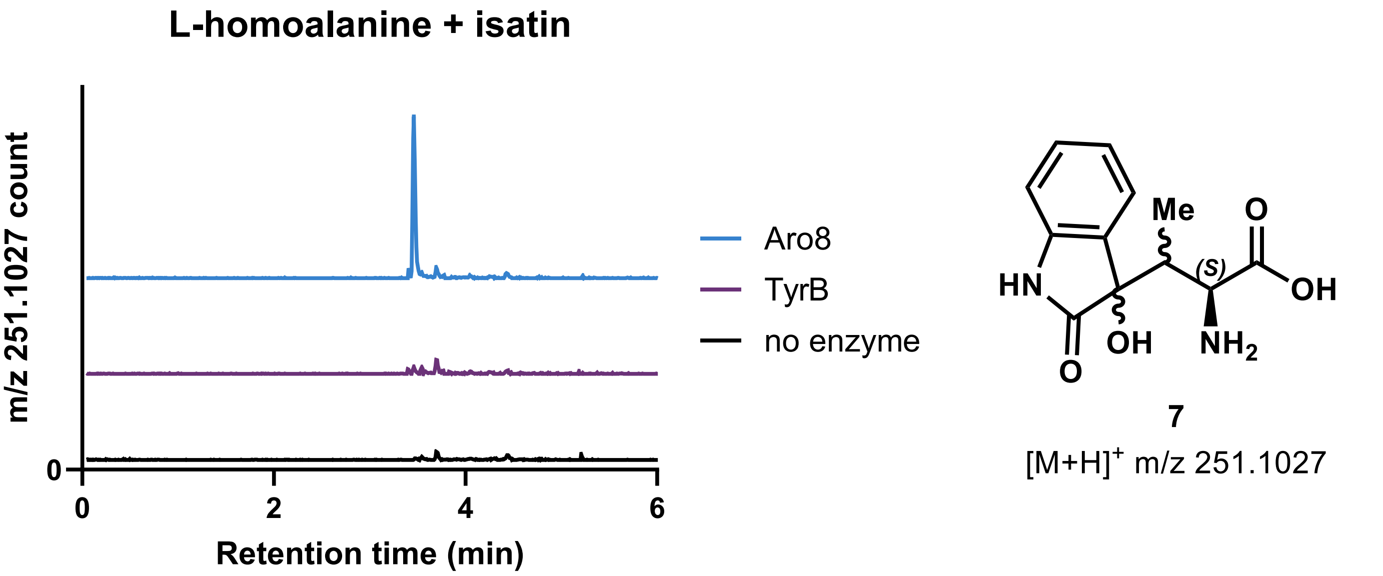

Figure S3. LC-MS chromatograms of Aro8- and TyrB-catalyzed reactions between L-homoalanine and isatin to form product **7**. Chromatograms are on same scale. Conditions: 50 mM isatin, 100 mM l-alanine, 50 mM KPi pH 8.0, 10% v/v DMSO, 25% v/v lysate, 30 °C, 18 h. Diluted 1:1000 before LC-TOF analysis.

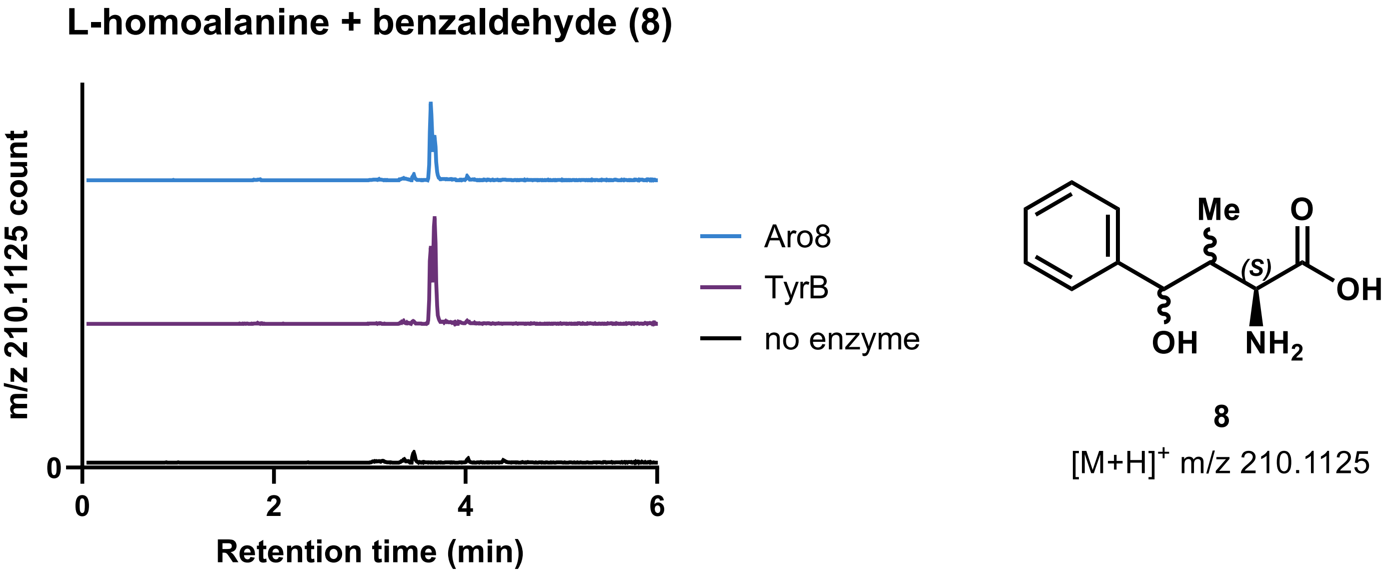

Figure S4. LC-MS chromatograms of Aro8- and TyrB-catalyzed reactions between L-homoalanine and benzaldehyde to form product **8**. Chromatograms are on same scale. Conditions: 50 mM isatin, 100 mM l-alanine, 50 mM KPi pH 8.0, 10% v/v DMSO, 25% v/v lysate, 30 °C, 18 h. Diluted 1:1000 before LC-TOF analysis.

Analytical-Scale Biocatalytic Reactions

Deuteration of L-alanine (1)

A master mix containing L-alanine (599 mM), PLP (120 µM) and potassium phosphate buffer (50.0 mM, pH 8.0) was prepared in a 1 mL microcentrifuge tube. To a 1 mL microcentrifuge tube was added 417 µL master mix, purified Aro8 (final concentration = 100 µM), isatin (50.0 µL of 500 mM solution in DMSO) and buffer (to 500 µL). Final concentrations: 100 µM Aro8, 500 mM L-alanine, 50.0 mM isatin, 100 µM PLP, 50.0 mM KP_i, 500 µL total reaction volume. The tube was incubated at 30 °C at 900 rpm in an Eppendorf ThermoMixer for 18 h. A 1.00 µL aliquot was added to 999 µL of quench solution (1:1 acetonitrile to 0.1% formic acid in water, 10 µM L-tryptophan methyl ester). The analyte was filtered before LC-TOF analysis.

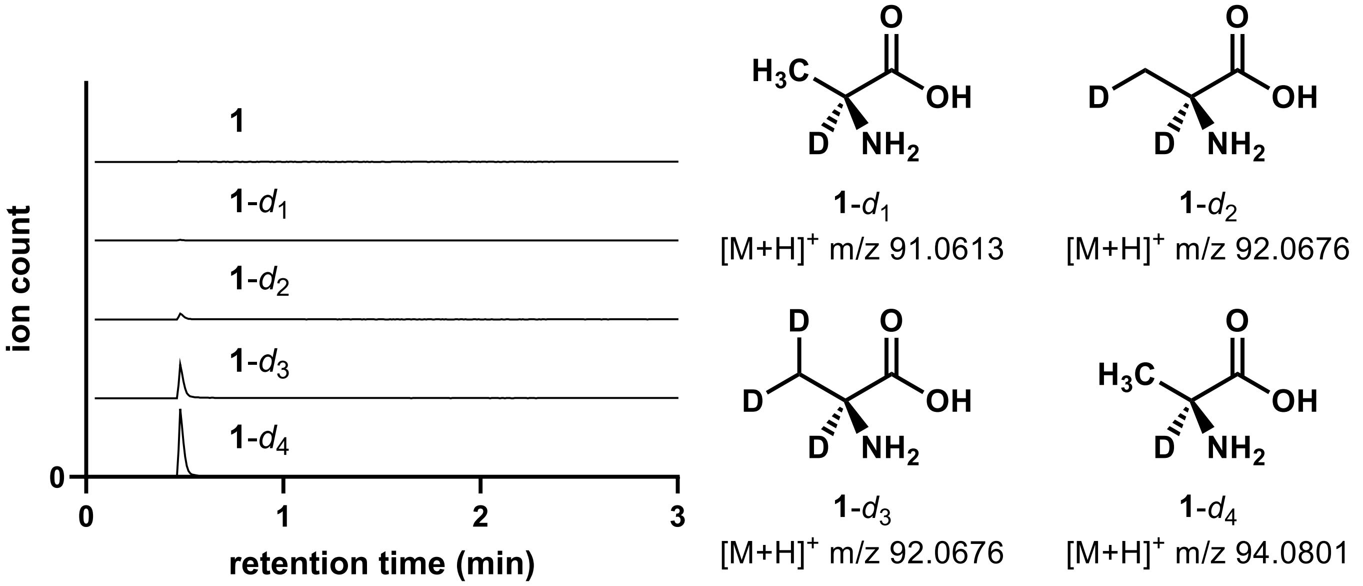

Figure S5. Extracted ion chromatograms (EICs) of L-alanine (**1**) deuteration with Aro8. $[M+H]^+$ **1**: 90.0550. $[M+H]^+$ **1**-d_1: 91.0613. $[M+H]^+$ **1**-d_2: 92.0676. $[M+H]^+$ **1**-d_3: 90.0550. $[M+H]^+$ **1**-d_4: 94.0801. All chromatograms are on same scale.

Reaction with d₄-L-alanine (1-d₄)

To a 1 mL microcentrifuge tube was added 0.89 mL potassium phosphate buffer (50 mM, pH 8.0), **1**-d_4 (9.3 mg), purified Aro8 (final concentration = 100 μM), PLP (10 μL of 10 mM solution in buffer) and isatin (100 μL of 500 mM solution in DMSO). Final concentrations: 100 μM Aro8, 100 mM **1**-d_4, 50 mM isatin, 100 μM PLP, 50 mM KP_i, 1.0 mL total reaction volume. The tube was incubated at 30 °C at 900 rpm in an Eppendorf ThermoMixer for 18 h. A 1.0 μL aliquot was added to 999 μL of quench solution (1: acetonitrile to 0.1% formic acid in water, 10 μM l-tryptophan methyl ester). The analyte was filtered before LC-TOF analysis.

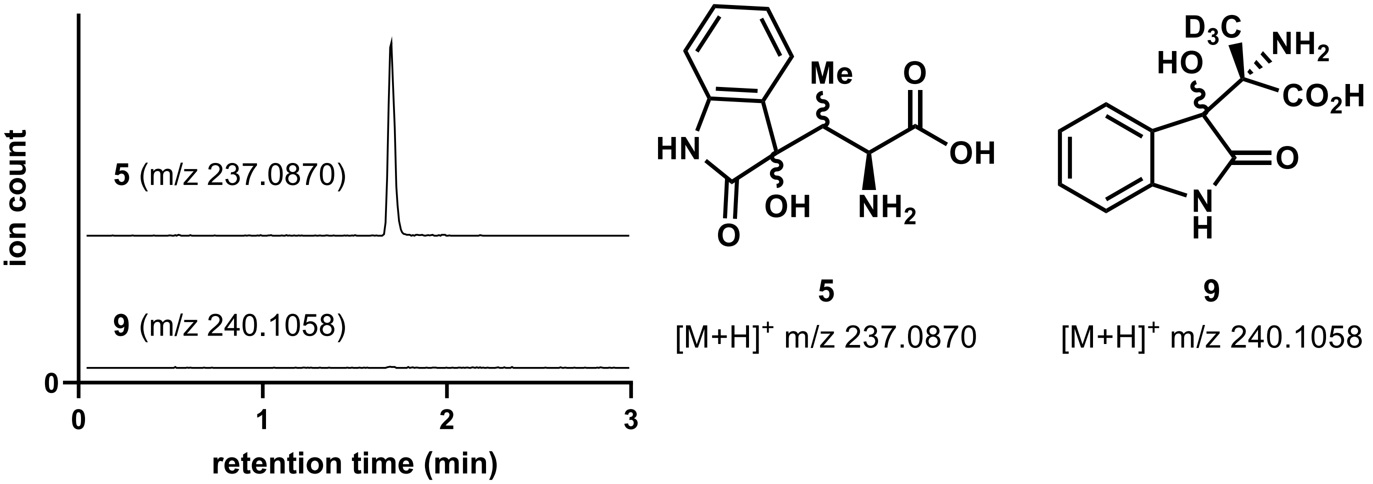

Figure S6. Extracted ion chromatograms (EICs) of possible products **5** and **9** after incubation of Aro8 with **1**-d_4.

Incubation of (R,S)-5 and (S,S)-5 with Aro8 and TyrB

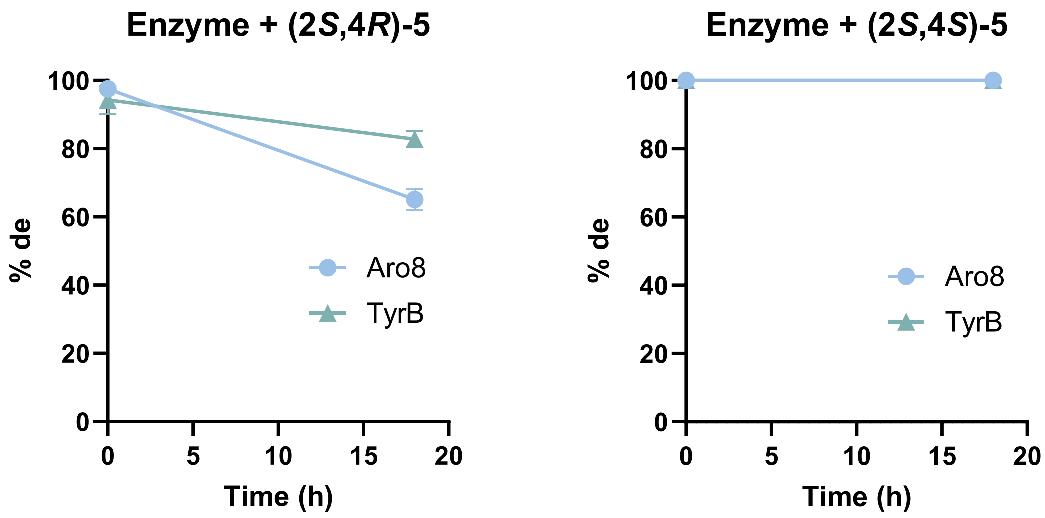

Figure S7. % de of **5** over time. Conditions: 5 mM (2*S*,4*R*)-**5** or (2*S*,4*S*)-**5**, 100 μM Aro8 or TyrB, 50 mM KPi pH 8.0, 30 °C. N=3. Reactions were monitored by LC-MS.

LC-MS/MS of 5

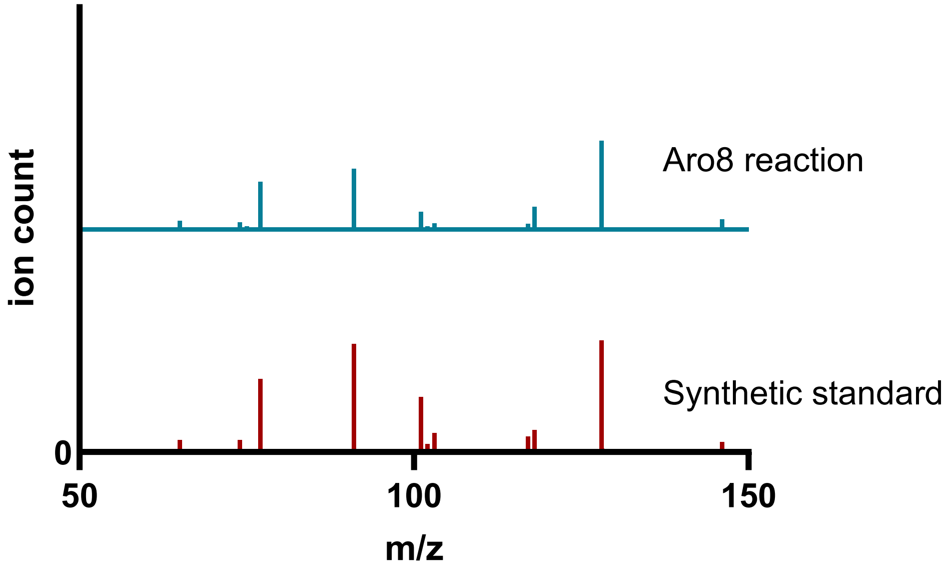

Figure S8. qTOF LC-MS/MS of **5** from synthetic standard and Aro8-catalyzed reaction. 100 V collision, 50 V fragmenter.

UV-Vis and CD Spectroscopy

Determination of Dissociation Constants

General Procedure

Protein was titrated with amino acid to determine dissociation constants (K_d). Purified Aro8 and TyrB were prepared in 50 mM phosphate buffer (pH 8.0) to a concentration of 20 μM enzyme in 1 mL total volume in a 1 mL cuvette. Stocks of substrates were prepared in the same buffer and titrated into the enzyme solution. Absorbance at λ = 353 nm (w/ Aro8) or λ = 364 nm (w/ TyrB) was measured 5 min after each addition. Full-spectrum absorbance was measured with addition of L-alanine; single-wavelength absorbance was measured for all subsequent substrates. Total volume added was negligible (< 60 μL). Absorbance of substrate-only sample was subtracted from each enzyme + substrate value. Binding of substrate to the enzyme was measured by monitoring loss in absorbance after each addition of substrate. Data was collected in triplicate and dissociation constant was calculated by fitting specific binding with Hill slope in GraphPad Prism.

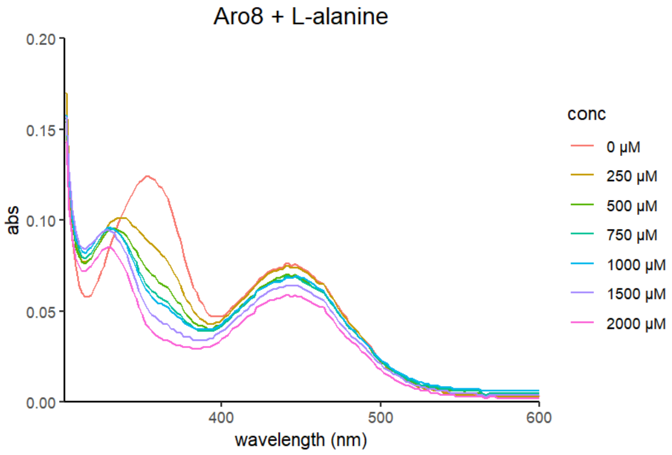

Figure S9. Absorbance of Aro8 with increasing L-alanine concentration. Change in signal at 353 nm was used for subsequent determinations of K_D, including for L-homoalanine and **5**.

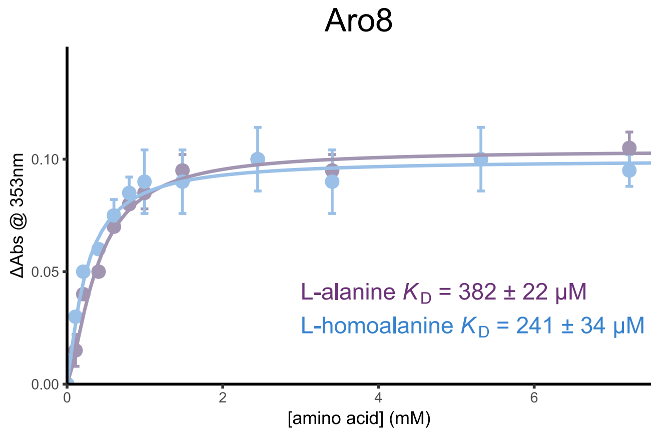

Figure S10. K_D of L-alanine and L-homoalanine with Aro8. n=3. Error bars represent standard deviation.

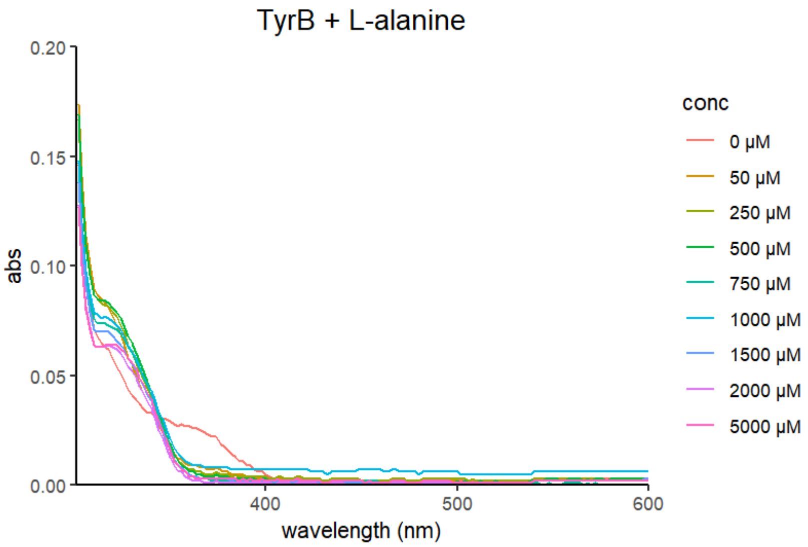

Figure S11. Absorbance of TyrB with increasing L-alanine concentration. Change in signal at 364 nm was used for subsequent determinations of K_D, including for L-homoalanine and **5**.

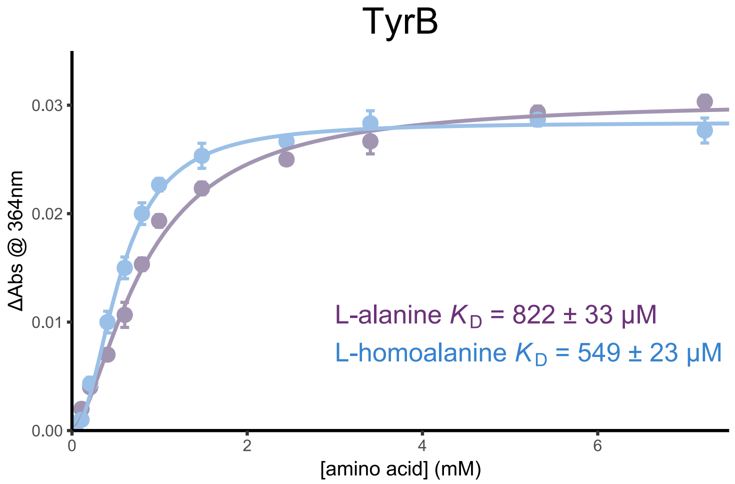

Figure S12. K_D of L-alanine and L-homoalanine with TyrB. n=3. Error bars represent standard deviation.

Dissociation constants of (2*S*,4*R*)-5 and (2*S*,4*S*)-5

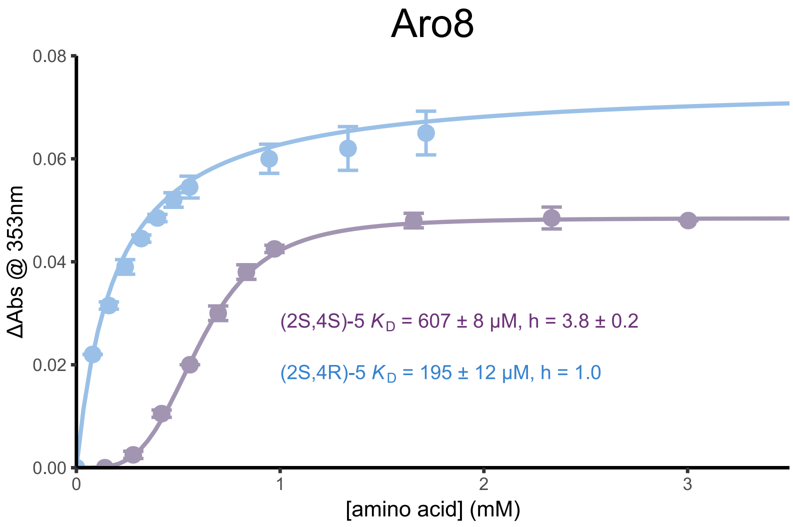

Figure S13. K_D of isomers of **5** with Aro8. h = Hill Coefficient. n = 3. Error bars represent standard deviation.

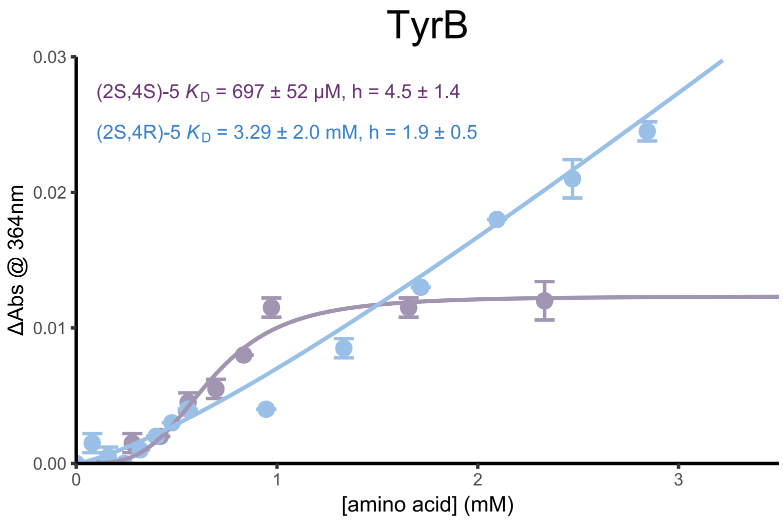

Figure S14. K_D of isomers of **5** with TyrB. h = Hill Coefficient. n=3. Error bars represent standard deviation.

Circular Dichroism (CD) Spectroscopy

General Procedure

To a 300 µL cuvette (path length 0.1 cm) was added a solution of enzyme (10 µM), PLP (10 µM), buffer (KP_i pH 8.0), and (2*S*,4*R*)-**5** or (2*S*,2*S*)-**5** (up to 1 mM). Absorbance between λ = 190 nm and λ = 260 nm was measured at 0.25 nm intervals, 100 nm/min scan rate. Spectrum of **5**-only was subtracted from enzyme + substrate spectra. Secondary structure prediction was performed on BeStSel (https://bestsel.elte.hu/index.php) using single spectrum analysis.

Preparative-Scale Biocatalytic Reactions

Protein Production and Purification

Batch-Scale Overexpression of PLP-Dependent Enzymes

15 mL of LB_{Kan} in a 50 mL conical tube was inoculated with BL21(DE3) *E. coli* harboring the plasmid of interest and incubated at 37 °C at 200 rpm overnight. 10 mL of this starter culture was used to inoculate 1 L of TB_{Kan}, then incubated at 37 °C, 250 rpm, until OD_{600} of ~0.8 was reached. The culture was cooled to 20 °C and protein expression was induced by addition of 1 mL of 1 M IPTG (final concentration: 1 mM). The culture was further incubated at 20 °C, 250 rpm, overnight. The culture was subsequently centrifuged at 3,500 ×*g* for 30 min. The supernatant was discarded and the cell pellet was collected, yielding an average of 25 g of cell pellet per liter of culture. The pellet could be lysed immediately, or flash-frozen in liquid nitrogen and stored at -80 °C. Typical yield: 20-25 g pellet per liter of culture.

Purification of PLP-Dependent Enzymes

BL21(DE3) *E. coli* cell pellet (prepared as described above) was resuspended in lysis buffer (50 mL/25 g pellet, 50 mM potassium phosphate pH 8.0, 150 mM NaCl, 30 mM imidazole, 10% v/v glycerol). The suspension was sonicated for a total of 3 min sonication time on ice (3 s pulse, 7 s no pulse). The lysate was clarified by centrifugation at 40,000 ×*g* for 30 min. The soluble fraction was separated and combined with Ni·NTA resin (1 mL per 25 mL lysate) and rocked at 4 °C for 1 h. The resin was subsequently collected on a fritted column and washed with wash buffer (3 mL, 50 mM potassium phosphate pH 8.0, 150 mM NaCl, 50 mM imidazole, 10% v/v glycerol). Protein was eluted with elution buffer (2.5 mL, 50 mM potassium phosphate pH 8.0, 150 mM NaCl, 300 mM imidazole, 10% v/v glycerol) into a 30 kDa molecular weight cutoff spin concentrator. After concentration to 2 mL concentrate per 25 g pellet used in the lysis, the protein was desalted on a PD10 desalting column using storage buffer (3.5 mL, 50 mM potassium phosphate, 150 mM NaCl, pH 8.0, 10% v/v glycerol). Protein concentration was determined by measuring absorbance at 280 nm. Extinction coefficients of enzymes were estimated with ProtParam (https://web.expasy.org/protparam).

Preparation of Lyophilized Lysate

BL21(DE3) *E. coli* cell pellet (prepared as described above) was resuspended in lysis buffer (50 mL/25 g pellet, 50 mM potassium phosphate pH 8.0, 150 mM NaCl, 30 mM imidazole). **Note the omission of glycerol.** The suspension was sonicated for a total of 3 min sonication time on ice (3 s pulse, 7 s no pulse). The lysate was clarified by centrifugation at 40,000 g for 30 minutes. The soluble fraction was transferred to a 50 mL conical tube (maximum 20 mL lysate per tube) and flash-frozen in liquid nitrogen. The frozen lysate was lyophilized for 48 h to yield ~3 g of yellow powder per 25 g cell pellet, which was suitable for preparative-scale reactions.

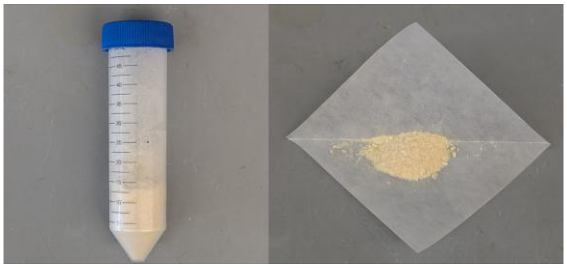

Figure S15. Lyophilized Aro8 lysate.

Estimation of enzyme content in lyophilized lysate

1 wt% lyophilized lysate (as prepared above) was diluted in buffer (50 mM potassium phosphate, pH 8.0). Aro8 lysate was diluted 1:10 and TyrB lysate was diluted 1:50. 25 µL of protein sample was mixed with 25 µL of loading dye (NEB, Purple, 6X, diluted to 2X) and boiled for 10 min. Meanwhile, purified Aro8 and TyrB were serially diluted with buffer (50 mM potassium phosphate, pH 8.0). Samples were analyzed by SDS-PAGE (3x replicates, 10 µL samples, 180 V, 400 A, 50 min). Lanes were converted to densitograms and band intensity was determined with ImageJ to produce calibration curves, which were subsequently used to estimate Aro8 or TyrB content in the original lysate solution.

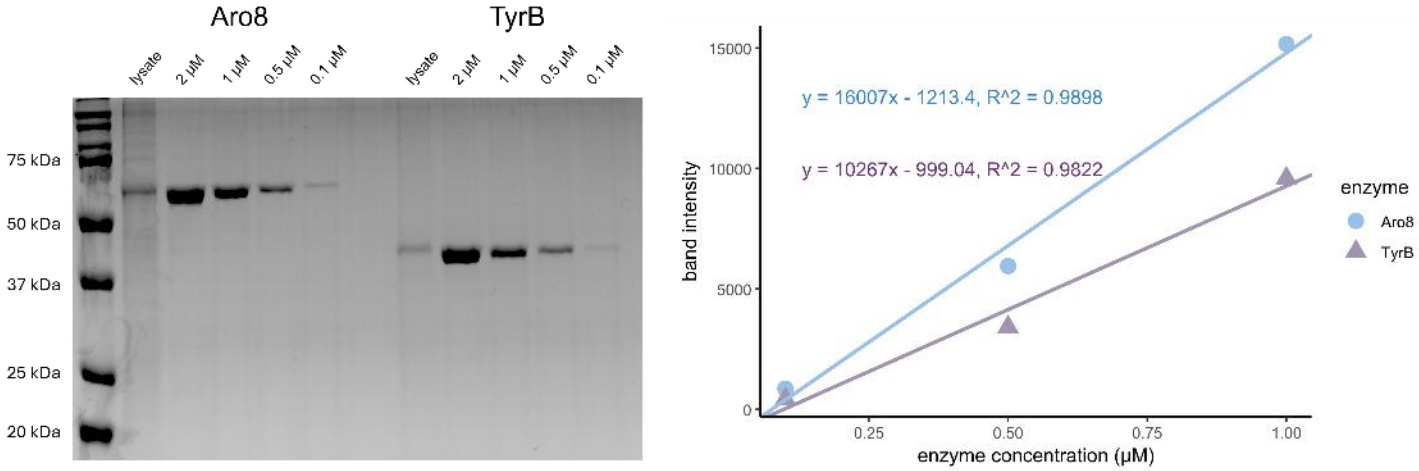

Figure S16. Representative SDS-PAGE gel (left) and calibration curve (right). Average across three replicates was used to estimate enzyme concentration in lysate. His-Aro8: 57.24 kDa. His-TyrB: 44.73 kDa. The 2 µM datapoints were omitted as these were outside the linear range.

	Aro8 band intensity			TyrB band intensity		
	replicate 1	replicate 2	replicate 3	replicate 1	replicate 2	replicate 3
lysate	6641.51	10367.865	5069.196	2141.033	2474.276	1673.376
1 μM	12320.933	21182.823	15169.296	9220.518	10598.246	9587.033
0.5 μM	5392.962	9116.024	5944.033	4246.619	4688.933	3415.962
0.1 μM	923.991	1280.719	857.234	610.577	551.284	426.749

Table S2. Band intensity of gels of purified Aro8 or TyrB, and lyophilized lysate.

	Aro8	TyrB
replicate 1 (μM)	11.34155	26.70559
replicate 2 (μM)	10.52385	28.26511
replicate 3 (μM)	7.849811	26.02918
average (μM)	9.90507	26.99996
std. dev. (μM)	1.82626	1.146664

Table S3. Estimated concentration of enzyme content in lyophilized lysate. Aro8: 10 ± 2 μM (0.57 ± 0.11 mg/mL). TyrB: 27 ± 1 μM (1.2 ± 0.045 mg/mL).

Biocatalytic Synthesis of γ-Hydroxy-α-Amino Acids

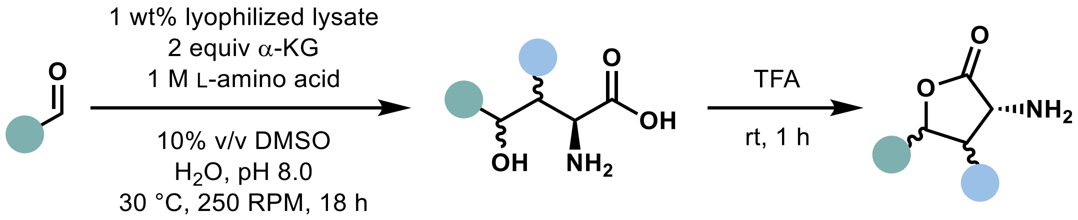

Scheme S1. Conditions for preparative-scale biocatalytic synthesis of γ-hydroxy-α-amino acids.

General Procedure

To a 50 mL conical tube was added L-amino acid (5 mmol, 20 equiv), α-ketoglutaric acid disodium salt dihydrate (113 mg, 0.5 mmol, 2 equiv), PLP (0.1 mg, 0.2 mol %), and water up to 4.5 mL. The pH was adjusted to 8.0 using 10 M NaOH. Then, lyophilized enzyme lysate (50 mg, 1 wt%) was added. To this suspension was added electrophile (0.5 mL of 0.5 M solution in DMSO, 0.25 mmol, 1 equiv). The reaction was incubated in a shaker at 30 °C, 200 rpm, for 18 h. After the reaction finished, the pH was adjusted to ~4 with 1 M HCl, then the reaction was centrifuged at 3,500 ×g for 10 min. The supernatant was purified by reverse-phase flash chromatography (CombiFlash, SiliaSep C4 80 g 40-63 µm, 60 Å column, 3 column volumes Solvent C, then 0-100% Solvent D over 10 column volumes). Fractions were analyzed by LC-MS and fractions containing product were pooled, concentrated, and lyophilized to white solids. Further purification by preparative HPLC was performed as needed.

To build calibration curves, each isolated product was dissolved in D_2O and analyzed by NMR with 1 mM maleic acid (for ^1H NMR) or 1 mM fluoroacetonitrile (for ^{19}F NMR) as internal standard to determine concentration of product. This solution was serially diluted to produce a calibration curve for LC-MS. The assay yield of biocatalytic reactions was thus determined by comparison to the calibration curve.

Lactonization

Isolated material, prepared as described above, was dissolved in 0.5 mL trifluoroacetic acid and stirred at room temperature for 1 h. Solvent was removed *in vacuo*, then the resulting residue was re-dissolved in CD_3CN and analyzed by NMR.

1-D NOESY parameters: 298.0 K, 64 scans, relaxation delay = 2 s.

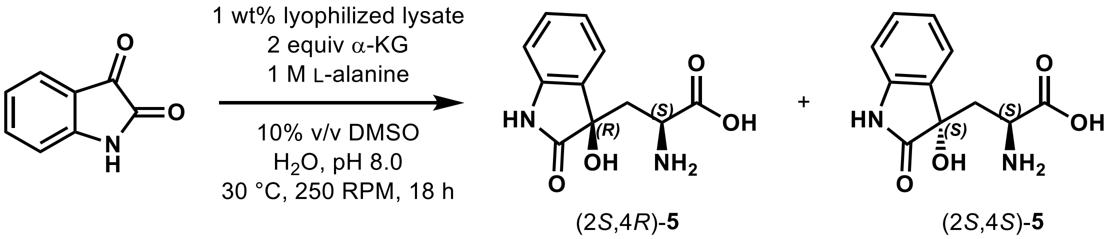

Scheme S2. Synthesis of **5** from isatin and L-alanine. Aro8-catalyzed reaction: 36.2 mg white solid (61% yield). TyrB-catalyzed reaction: 8.8 mg white solid (15% yield).

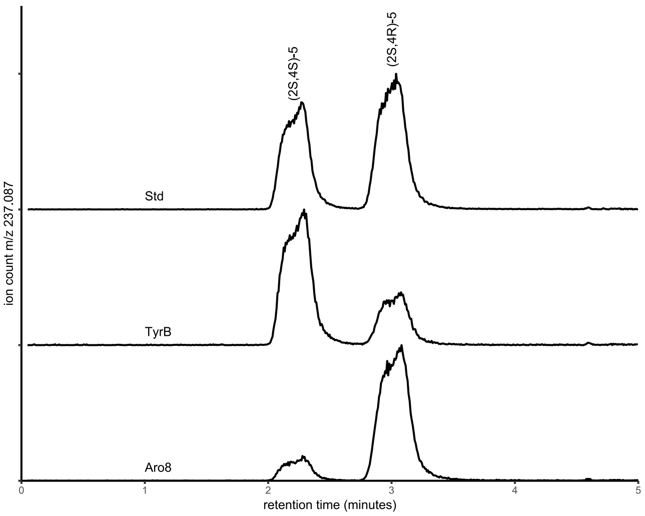

Figure S17. Achiral LC-MS extracted ion chromatogram (EIC) of synthetic standard, TyrB-catalyzed, and Aro8-catalyzed reactions. Column: Waters Acquity HSS t3 2.1x75 mm. Gradient: 0-1 min 100% Solvent A, 1-3 min 0-100% Solvent B, 3-3.5 min 100% Solvent B.

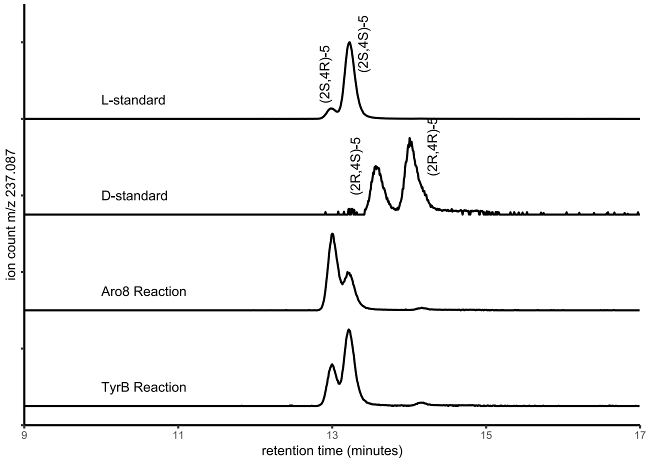

Figure S18. Chiral LC-MS extracted ion chromatogram (EIC) of synthetic standard, TyrB-catalyzed, and Aro8-catalyzed reactions. Column: Astec CHIROBIOTIC T2. Gradient: 0-6 min 90% Solvent B, 6-25 min 10-90% Solvent A. No D-**5** is detected in the enzymatic reactions.

Peak	Peak area	Area %
Aro8 (2*S*,4*S*)-**5**	561963.94	14
Aro8 (2*S*,4R)-**5**	3569154.20	86
TyrB (2*S*,4*S*)-**5**	2351943.35	72
TyrB (2*S*,4R)-**5**	915967.49	28

Table S4. Tabulated data from Fig. S17 to determine de of Aro8- and TyrB-catalyzed reactions.

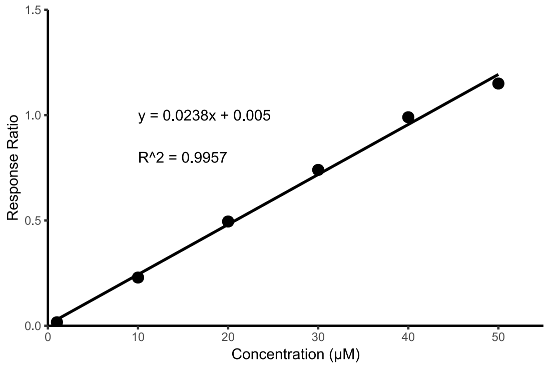

Figure S19. LC-MS calibration curve of **5**. Response ratio = product area / TrpOMe area. n=3

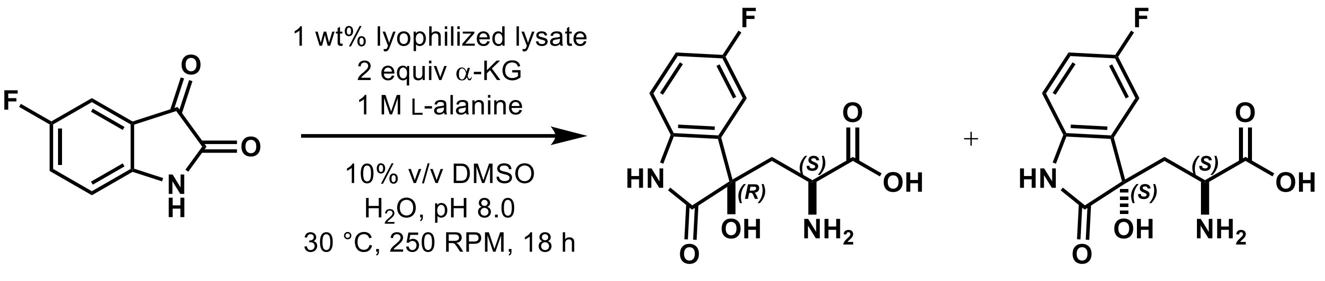

(2*S*,4*R*)-**12** (2*S*,4*S*)-**12**

Scheme S3. Synthesis of **12** from isatin and L-alanine. Aro8-catalyzed reaction: 11.6 mg white solid (18% yield). TyrB-catalyzed reaction: 14.7 mg white solid (23% yield).

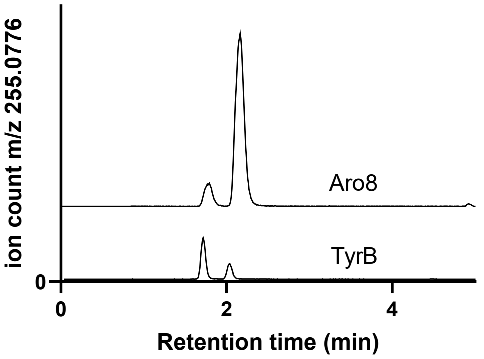

Figure S20. LC-MS extracted ion chromatogram (EIC) of **12** from Aro8-catalyzed and TyrB-catalyzed reactions. Column: Waters Acquity HSS t3 2.1x75 mm. Gradient: 0-1 min 100% Solvent A, 1-4 min 0-100% Solvent B, 4-5 min 100% Solvent B.

(2*S*,4*R*)-12 data:

^{1}H NMR (D_2O, 599 MHz): δ 7.10 (dd, *J* = 7.9, 2.8 Hz, 1H), 7.01 – 6.94 (m, 1H), 6.85 (dt, *J* = 8.3, 3.6 Hz, 1H), 4.48 (dd, *J* = 9.5, 3.4 Hz, 1H), 2.41 (dd, *J* = 15.6, 9.6 Hz, 0H), 2.29 (dd, *J* = 15.6, 3.3 Hz, 1H);

^{13}C NMR (D_2O, 151 MHz): δ 178.4, 156.6, 155.1, 140.3 (d, *J* = 2.1 Hz), 126.6 (d, *J* = 6.5 Hz), 119.4 (d, *J* = 7.7 Hz), 115.6 (d, *J* = 22.3 Hz), 113.5 (d, *J* = 24.6 Hz), 78.6 (d, *J* = 1.7 Hz), 54.4, 39.6;

^{19}F NMR (D_2O, 564 MHz): δ -119.86 (ddd, J = 9.5, 7.6, 4.2 Hz);

HRMS (ESI+): $[M+H]^+ = C_{11}H_{12}FN_2O_4^+$. Calculated m/z: 255.0776, found 255.0775.

(2*S*,4*S*)-12 data:

^{1}H NMR (D_2O, 599 MHz): δ 7.17 (d, *J* = 7.4 Hz, 1H), 7.05 (td, *J* = 9.0, 2.7 Hz, 1H), 6.89 (d, *J* = 4.2 Hz, 1H), 4.80 (t, *J* = 9.2 Hz, 1H), 2.95 (dd, *J* = 14.1, 9.6 Hz, 1H), 2.70 (dd, *J* = 14.1, 9.0 Hz, 1H);

^{13}C NMR (D_2O, 151 MHz): δ 180.1, 160.2, 158.6, 135.6 (d, *J* = 2.1 Hz), 131.8 (d, *J* = 8.1 Hz), 116.7 (d, *J* = 23.9 Hz), 112.1 (d, *J* = 8.2 Hz), 111.5 (d, *J* = 25.4 Hz), 74.7, 49.1, 36.1;

^{19}F NMR (D_2O, 564 MHz): δ -119.15 (td, J = 8.9, 4.4 Hz);

HRMS (ESI+): $[M+H]^+ = C_{11}H_{12}FN_2O_4^+$. Calculated m/z: 255.0776, found 255.0774.

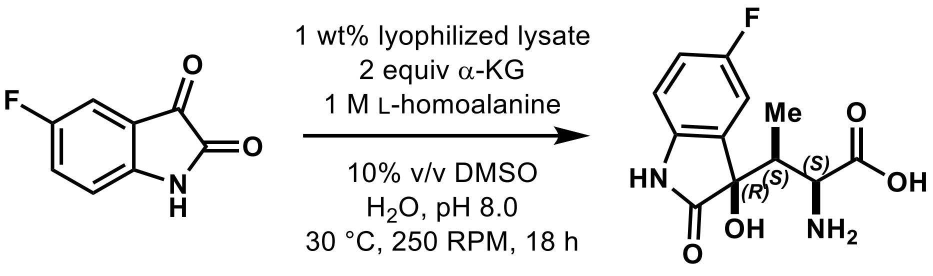

(2*S*,3*S*,4*R*)-**13**

Scheme S4. Synthesis of **13** from isatin and L-alanine. Aro8-catalyzed reaction: 10.7 mg white solid (16% yield).

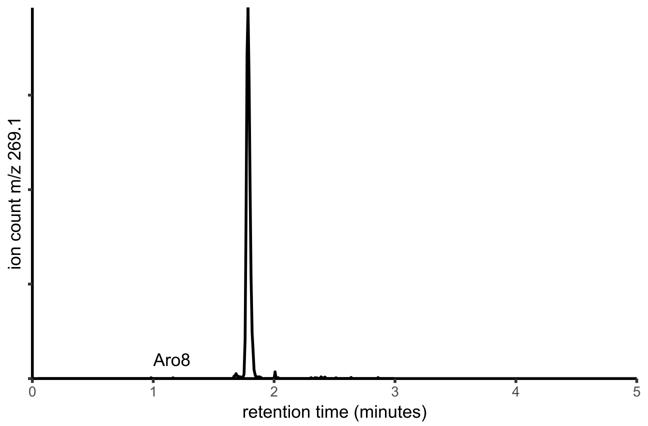

Figure S21. LC-MS extracted ion chromatogram (EIC) of **13** from Aro8-catalyzed reaction. Column: Waters Acquity HSS t3 2.1x75 mm. Gradient: 0-1 min 100% Solvent A, 1-4 min 0-100% Solvent B, 4-5 min 100% Solvent B.

^{1}H NMR (D_2O/LiOH, 599 MHz): δ 6.76 (td, J = 8.5, 2.7 Hz, 1H), 6.68 (dd, J = 8.8, 5.1 Hz, 1H), 6.53 (dd, J = 10.4, 2.9 Hz, 1H), 3.66 (d, J = 6.0 Hz, 1H), 2.66 (p, J = 6.9 Hz, 1H), 0.80 (d, J = 7.1 Hz, 3H);

^{13}C NMR (D_2O/LiOH, 151 MHz) δ 180.85, 177.80, 156.77, 155.21, 141.10, 130.07, 119.22 (d, J = 7.8 Hz), 117.19, 115.25, 114.26 (d, J = 22.0 Hz), 112.56 (d, J = 23.5 Hz), 83.61, 59.24, 43.06, 9.34;

^{19}F NMR (D_2O/LiOH, 563 MHz) δ -124.32 (td, J = 9.3, 4.7 Hz), -126.20 (td, J = 9.3, 5.3 Hz).

HRMS (ESI+): $[M+H]^+ = C_{12}H_{14}FN_2O_4^+$. Calculated m/z: 269.0933, found 269.0934.

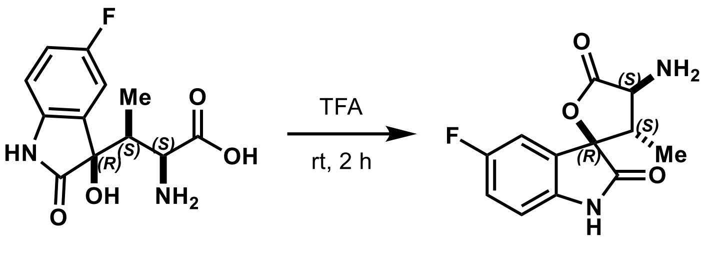

Scheme S5. Conversion of **13** to **22** for NOE analysis.

^{1}H NMR (CD_3CN, 599 MHz): δ 8.85 (s, 1H), 7.38 – 6.85 (m, 3H), 4.47 (d, *J* = 11.3 Hz, 1H), 3.18 (dd, *J* = 11.3, 6.1 Hz, 1H), 1.02 (d, *J* = 6.3 Hz, 3H);

^{13}C NMR (CD_3CN, 151 MHz): δ 173.9, 171.0, 160.6, 159.0, 139.3, 125.0 (d, *J* = 8.0 Hz), 119.2 (d, *J* = 23.7 Hz), 114.7 (d, *J* = 25.9 Hz), 113.4 (d, *J* = 8.0 Hz), 85.8, 55.1, 41.7, 12.6;

^{19}F NMR (CD_3CN, 563 MHz): δ -120.96 (td, *J* = 9.0, 4.5 Hz);

HRMS (ESI+): $[M+H]^+ = C_{12}H_{12}FN_2O_3^+$. Calculated m/z: 251.0827, found 251.0826.

1-D NOESY: (CD_3CN, 699 MHz):

Excite @ 7.29 ppm: δ 4.50 (d, *J* = 11.4 Hz), 1.05 (s);

Excite @ 4.50 ppm: δ 7.29 (d, *J* = 8.1 Hz), 1.05 (d, J = 6.8 Hz);

Excite @ 1.05 ppm: δ 7.28 (s), 4.50 (d, *J* = 11.6 Hz), 3.21 (s).

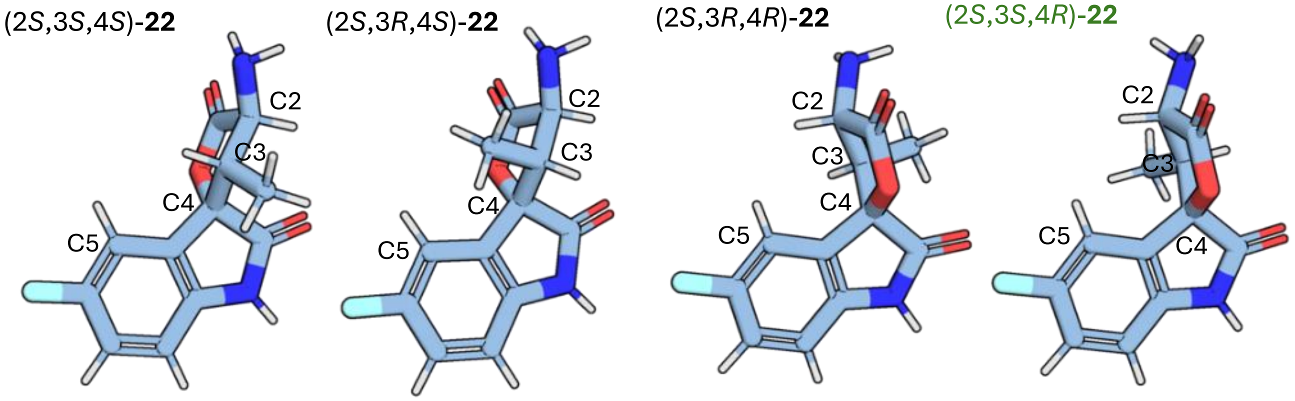

Figure S22. Energy minimized structures (https://rowansci.com)[1]. Engine: AIMNet2[2]. Mode: Careful

Table S5. Relevant bond lengths from **Figure S22**.

Isomer	C5-H to C2-H (Å)	C5-H to C6-H (Å)	C2-H to C6-H (Å)
(2*S*,3*S*,4*R*)-**22**	2.5	3.0	2.6
(2*S*,3*R*,4*R*)-**22**	2.3	5.5	3.7
(2*S*,3*R*,4*S*)-**22**	5.3	2.5	3.7
(2*S*,3*S*,4*S*)-**22**	5.2	4.0	2.5

Only the (2*S*,3*S*,4*R*)-**22** isomer has bond lengths that are reasonable for NOE analysis.

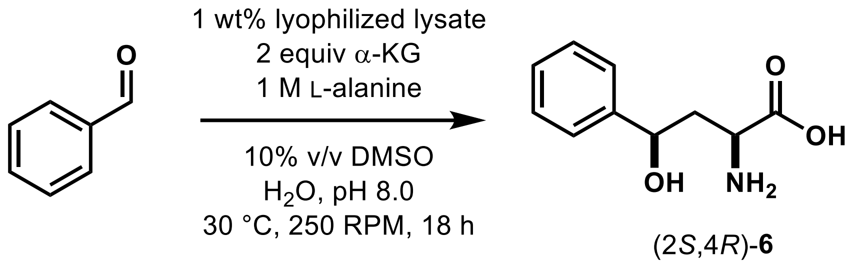

Scheme S6. Synthesis of **16** from isatin and L-alanine. Aro8-catalyzed reaction: 11.6 mg white solid (18% yield). TyrB-catalyzed reaction: 14.7 mg white solid (23% yield).

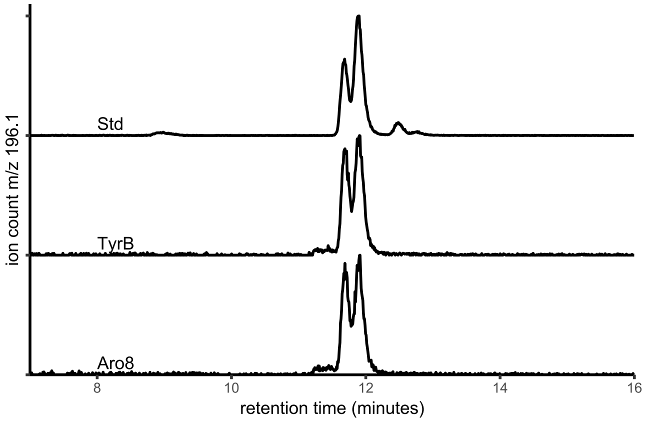

Figure S23. LC-MS extracted ion chromatogram (EIC) of authentic standard, TyrB-catalyzed, and Aro8-catalyzed reactions. Astec CHIROBIOTIC T2. Gradient: 0-10 min 100% Solvent B, 10-25 min 0-100% Solvent A. **Not used to determine de.**

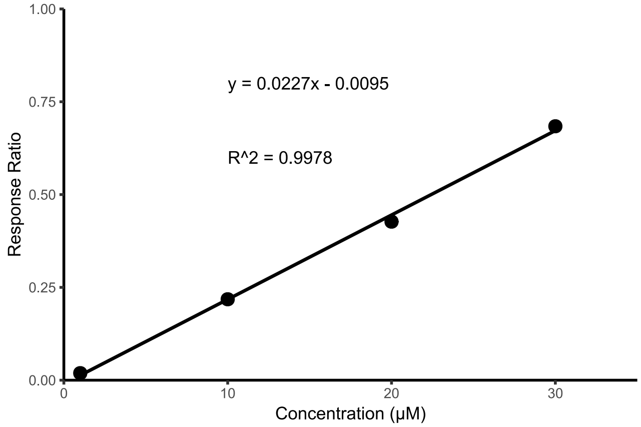

Figure S24. LC-MS calibration curve. Response ratio = product area / TrpOMe area. N=3

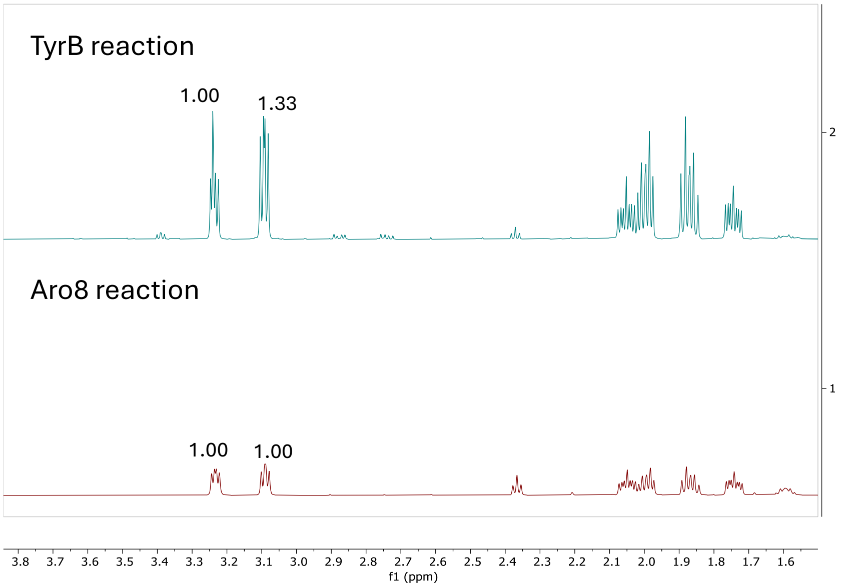

Figure S25. Isolated ^{1}H NMR of TyrB- (top) and Aro8-catalyzed (bottom) reactions. de was determined by comparing peak area of δ 3.24-3.21 ((2*S*,4*S*)-**6**) and δ 3.11-3.08 ((2*S*,4*R*)-**6**).

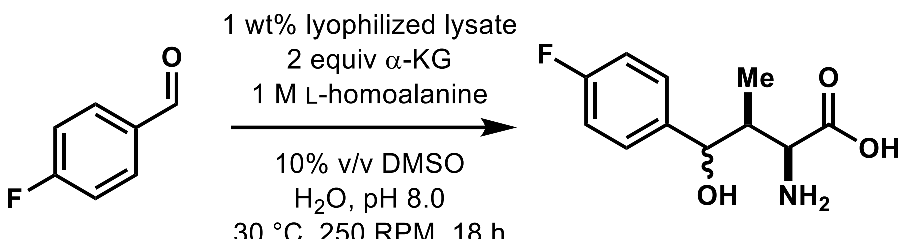

14

Scheme S7. Synthesis of **14** from 4-fluorobenzaldehyde and L-homoalanine. Aro8-catalyzed reaction: 14.8 mg white solid (4% yield). TyrB-catalyzed reaction: 11.4 mg white solid (3% yield).

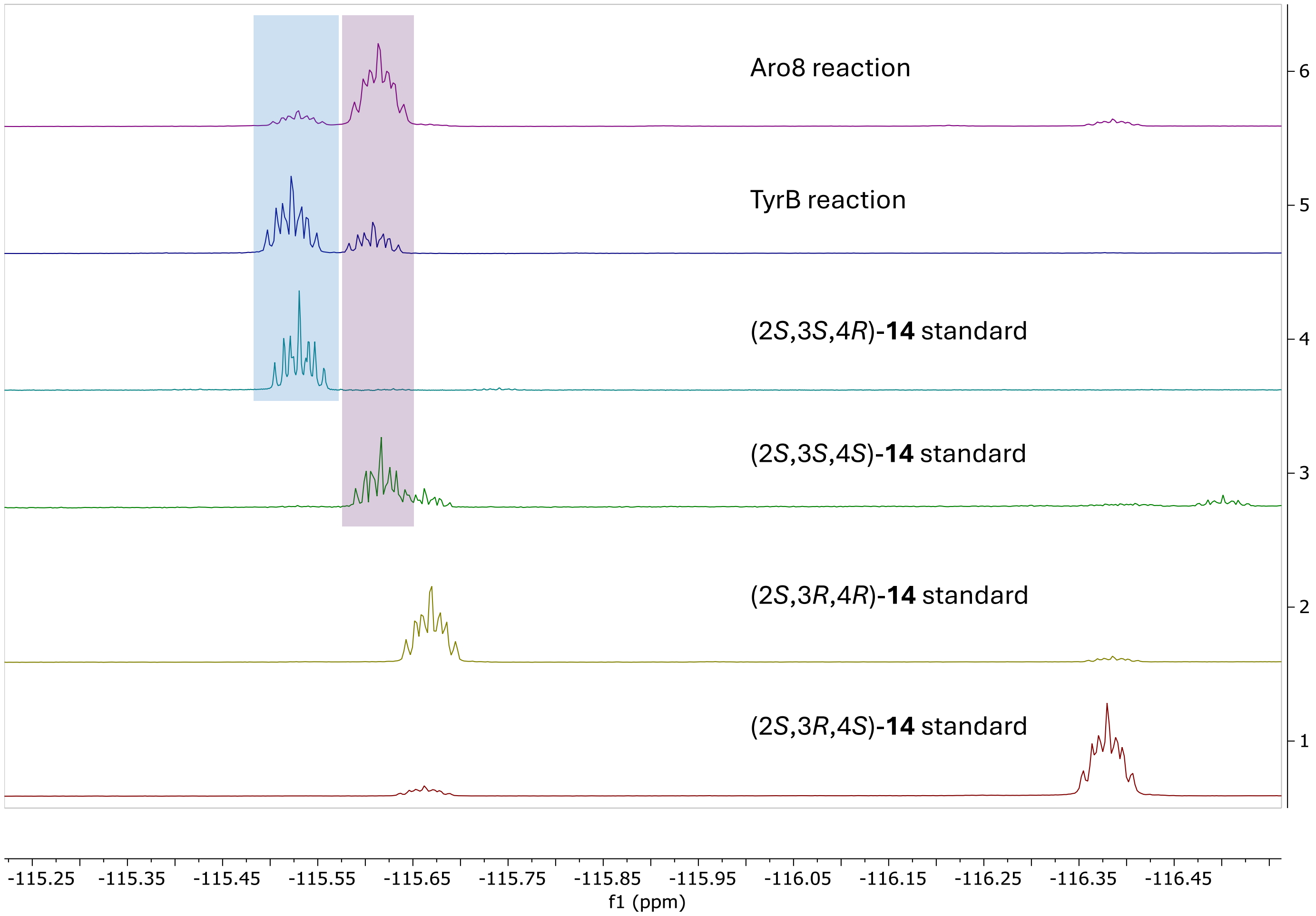

Figure S26. ^{19}F NMR (D_2O/LiOH, 563 MHz) of isolated **14** from Aro8 and TyrB reactions, compared to synthetic standards. Shifts normalized to fluoroacetonitrile internal standard (δ -217.0).

Aro8 Reaction: δ -115.49 – -115.57 (m), -115.61 (dq, *J* = 15.0, 7.2 Hz), -116.38 (q, *J* = 6.3 Hz).

TyrB Reaction: δ -115.52 (td, *J* = 9.5, 4.4 Hz), -115.60 (td, *J* = 9.4, 4.5 Hz).

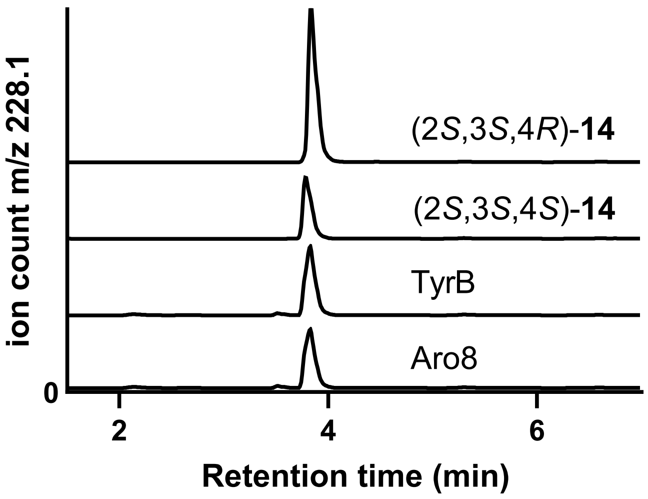

Figure S27. LC-MS extracted ion chromatogram (EIC) of **14** from synthetic standards, Aro8-catalyzed, and TyrB-catalyzed reactions. Column: Waters Acquity HSS t3 2.1x75 mm. Gradient: 0-2 min 100% Solvent A, 2-6 min 0-100% Solvent B, 6-7 min 100% Solvent B. Further LC method development was unable to separate the diastereomers chromatographically.

Chemical Synthesis

Synthesis of dioxindolyl-L-alanine (**5**) standards

Authentic standards of dioxindolyl-L-alanine (**5**) were prepared by method of Graboski et al.[3] Dioxindolyl-D-alanine (D-**5**) were prepared using D-tryptophan.

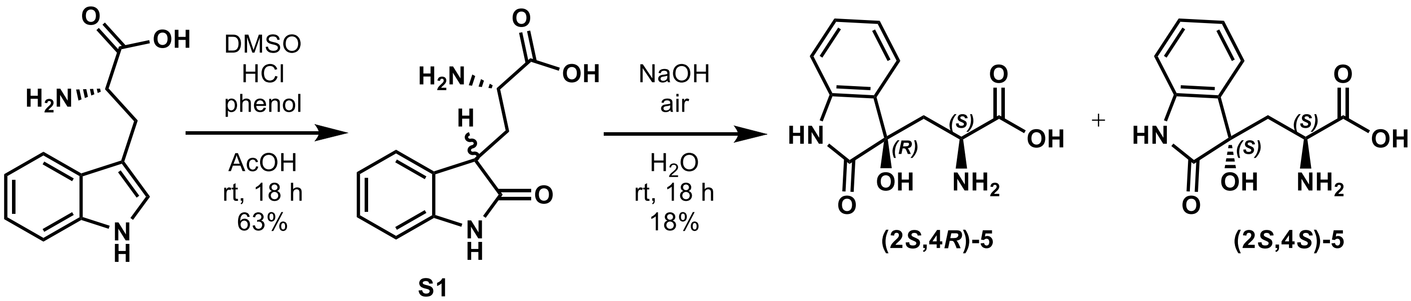

Scheme S8. Synthesis of **5**.

Oxindolyl-L-alanine (S1): L-tryptophan (1.0 g, 4.9 mmol, 1.0 equiv) was dissolved in glacial acetic acid (15 mL). To the stirred suspension was added a solution of phenol (92 mg, 0.98 mmol, 0.20 equiv), DMSO (1.0 mL, 0.010 mol, 3.0 equiv), and concentrated HCl (1.0 mL). Reaction was stirred at room temperature for 18 h, then concentrated *in vacuo* to a syrup. After re-dissolving in minimal water, the residue was purified by flash chromatography (reverse phase on CombiFlash, SiliaSep C4 80 g 40-63 µm, 60 Å column, 100% Solvent C for 3 column volumes, then 0-100% Solvent D over 10 column volumes). Product was lyophilized to a white solid (0.69 g, 63% yield as a 1.46:1 mixture of diastereomers).

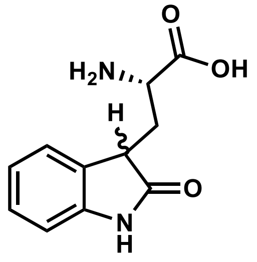

S-1

TLC: eluted in 3:1:1 *n*-butanol:glacial acetic acid:water, stained in ninhydrin. R_f = 0.67, orange.

^{1}H NMR (D_2O, 599 MHz): δ 7.36 (mixture of isomers, dd, *J* = 16.05, 7.47 Hz, 1H), 7.33 – 7.29 (mixture of isomers, m, 1H), 7.14 (mixture of isomers, q, *J* = 7.10 Hz, 1H), 7.00 (mixture of isomers, t, *J* = 7.94 Hz, 1H), 4.14 (major, t, *J* = 5.48 Hz, 1H), 4.02 (minor, dd, *J* = 8.80, 5.13 Hz, 1H), 2.57 (major, dd, *J* = 15.11, 5.13 Hz, 1H), 2.51 (major, dd, *J* = 15.26, 6.13 Hz, 1H), 2.23 (minor, dd, *J* = 15.22, 4.68 Hz, 1H), 2.16 (minor, dd, *J* = 15.12, 8.90 Hz, 1H);

^{13}C NMR (D_2O, 151 MHz): δ 181.6, 181.4, 171.9, 171.8, 141.3, 128.7, 128.7, 124.1, 124.0, 123.0, 110.6, 110.5, 51.7, 51.6, 38.7, 30.0, 29.9;

LRMS (ESI+): $[M+H]^+ = C_{11}H_{13}N_2O_3^+$. Calculated m/z: 221.1, found 221.1.

Dioxindolyl-L-alanine (5): Oxindolyl-L-alanine **S1** (679 mg, 3.08 mmol, 1.00 equiv) was dissolved in water (5.0 mL). To this solution was added 9.25 mL of 1.0 M NaOH (9.25 mmol, 3.00 equiv). Reaction was stirred at room temperature with bubbling air for 18 h. The pH was adjusted to ~7 with glacial acetic acid, then concentrated *in vacuo* to a syrup. After re-dissolving in minimal water, the residue was purified by flash chromatography (reverse phase on CombiFlash, SiliaSep C4 80 g 40-63 μm, 60 Å column, 100% Solvent C for 3 column volumes, then 0-100% Solvent D over 10 column volumes). Product was lyophilized to a brown solid (250 mg). The solid was re-dissolved in 5.0 mL water and purified by preparative HPLC (Kinetex 5 μm C18 100 Å column, 100% Solvent C for 15 min, then 0-5% Solvent D over 15 min) to separate the diastereomers. The products were lyophilized to white solids (36 mg (2*S*,4*R*), 98 mg (2*S*,4*S*), 134 mg total, 0.57 mmol, 18% total yield).

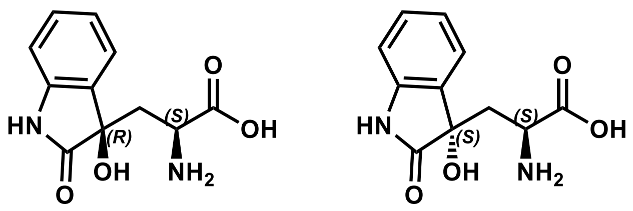

dioxindolyl-L-alanine (**5**)

(2*S,*4*R*)-**5** ^{1}H NMR (D_2O/LiOH, 600 MHz): δ 7.45 (d, *J* = 7.9 Hz, 1H), 7.39 (t, *J* = 7.7 Hz, 0H), 7.19 (t, *J* = 7.6 Hz, 1H), 7.04 (d, *J* = 7.8 Hz, 1H), 4.59 (dd, *J* = 9.6, 3.6 Hz, 1H), 2.57 (dd, *J* = 15.7, 9.6 Hz, 1H), 2.43 (dd, *J* = 15.7, 3.6 Hz, 1H);

(2*S*,4*S*)-**5** ^{1}H NMR (D_2O/LiOH, 600 MHz): δ 7.52 (d, *J* = 7.5 Hz, 1H), 7.42 (t, *J* = 7.7 Hz, 1H), 7.22 (t, *J* = 7.6 Hz, 1H), 7.07 (d, *J* = 6.9 Hz, 1H), 4.36 (dd, *J* = 9.5, 3.7 Hz, 1H), 2.57 (dd, *J* = 15.6, 3.7 Hz, 1H), 2.33 (dd, *J* = 15.5, 9.3 Hz, 1H);

(2*S*,4*R*)-**5** ^{13}C NMR (D_2O/LiOH, 600 MHz): δ 180.1, 172.0, 139.7, 130.5, 130.4, 123.7, 123.6, 111.1, 74.6, 49.4, 36.3;

(2*S*,4*S*)-**5** ^{13}C NMR (D_2O/LiOH, 600 MHz): δ 180.7, 172.4, 140.0, 130.6, 129.8, 123.9, 123.6, 111.4, 74.9, 50.2, 36.5;

(2*S*,4*R*)-**5** HRMS (ESI+): $[M+H]^+ = C_{11}H_{13}N_2O_4^+$. Calculated m/z: 237.0870, found 237.1867.

(2*S*,4*S*)-**5** HRMS (ESI+): $[M+H]^+ = C_{11}H_{13}N_2O_4^+$. Calculated m/z: 237.0870, found 237.1865.

Synthesis of **6** standards

Authentic standards of **6** were prepared by method of Murashige et al.[4]

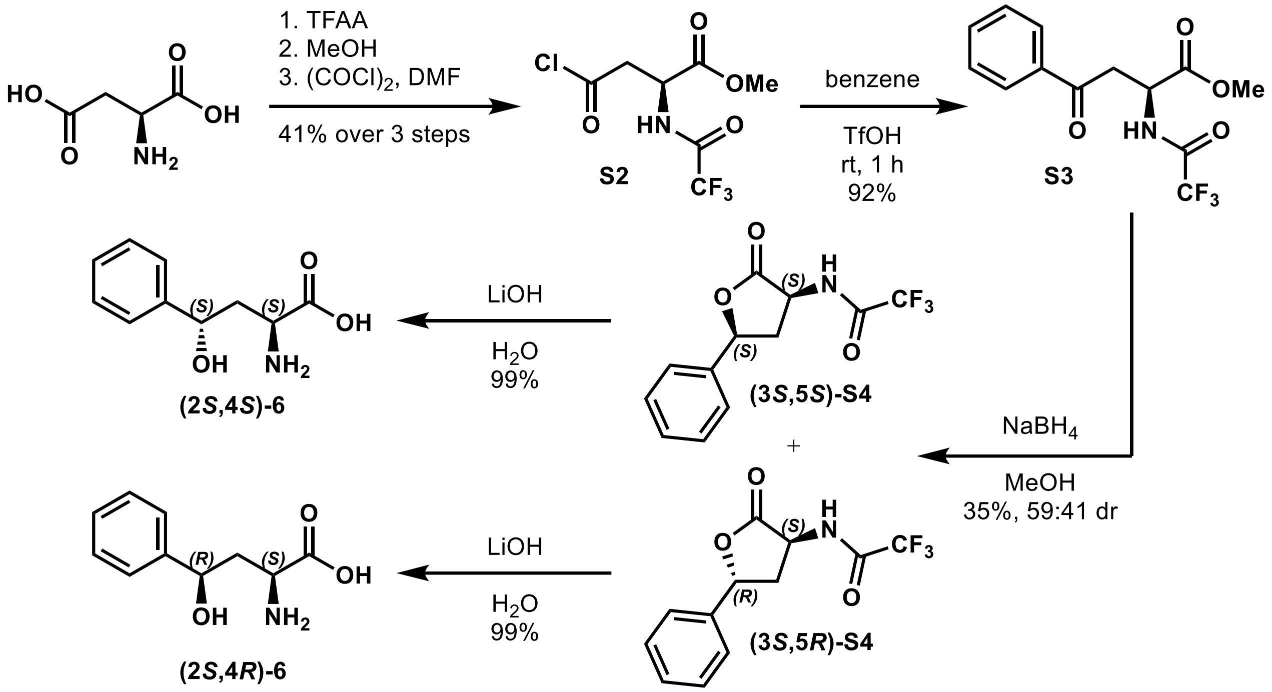

Scheme S9. Synthesis of **6**.

methyl (*S*)-4-chloro-4-oxo-2-(2,2,2-trifluoroacetamido)butanoate (S2): L-aspartic acid (10.0 g, 75.1 mmol, 1.00 equiv) was added to a 3-necked 250 mL round-bottom flask equipped with a reflux condenser and addition funnel. The flask was cooled to -78 °C. While vigorously stirring, trifluoroacetic anhydride (26.5 mL, 188 mmol, 2.50 equiv) was added slowly. The reaction was slowly warmed to 40 °C and held at this temperature for 2 h. A chalky white solid precipitated from solution, which was filtered and washed with petroleum ether.

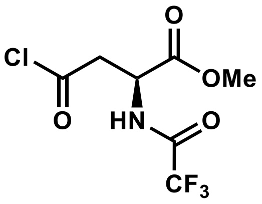

S2

The solid was then dissolved in 30 mL anhydrous methanol and stirred at reflux for 1 h. The solution was concentrated under vacuum and triturated with petroleum ether to yield a white solid.

The solid was dissolved in toluene (50 mL). To this solution was added oxalyl chloride (15.7 mL, 179 mmol, 3.00 equiv) and dimethylformamide (0.462 mL, 6.00 mmol, 0.100 equiv). The solution was heated at reflux for 2 h, then slowly cooled to room temperature to precipitate product as a yellow solid. The solid was filtered and washed with cold toluene, then recrystallized from refluxing benzene to yield yellow needle-like crystals (8.06 g, 41% yield from L-aspartic acid).

^{1}H NMR ($CDCl_3$, 600 MHz): δ 7.19 (br s, 1H), 4.78-4.74 (dt, *J* = 4.34, 4.34, 7.25 Hz, 1H), 3.85 (s, 3H), 3.71-3.56 (m, 2H);

^{13}C NMR ($CDCl_3$, 151 MHz): δ 47.4, 49.2, 53.8, 118.9-111.8 (m), 157.8-156.5 (q, 38.4, 38.4, 38.5), 168.3, 172.2;

^{19}F NMR ($CDCl_3$, 563 MHz): δ -75.76 (s).

methyl-(*S*)-4-oxo-4-phenyl-2-(2,2,2-trifluoroacetamido)butanoate (S3): methyl (*S*)-4-chloro-4-oxo-2-(2,2,2-trifluoroacetamido)butanoate **S2** (0.500 g, 1.91 mmol, 1.00 equiv) was added to a flame-dried round bottom flask. The flask was cooled to 0 °C, then triflic acid (7.00 mL) was added slowly. While stirring, benzene (0.170 mL, 1.91 mmol, 1.00 equiv) was added dropwise. The reaction was slowly warmed to room temperature and stirred for 1 h. The reaction was added to an ice cold mixture of 100 mL water/100 mL EtOAc. The organic layer was washed sequentially with 1 M HCl (100 mL), saturated sodium bicarbonate (3x 100 mL), 1 M HCl (100 mL), brine (3x 100 mL), dried over sodium sulfate, filtered, and concentrated to an off-white solid (533 mg, 92% yield).

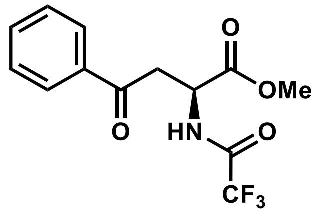

S3

^1H NMR ($CDCl_3$, 600 MHz): δ 8.02-7.83 (d, *J* = 7.03 Hz, 2H), 7.68-7.59 (t, *J* = 7.43, 7.43 Hz, 1H), 7.54-7.46 (m, 2H), 5.02-4.93 (dt, *J* = 3.89, 3.89, 8.00 Hz, 1H), 3.92-3.84 (dd, *J* = 3.87, 18.41 Hz, 1H), 3.79 (s, 3H), 3.64-3.54 (dd, *J* = 3.95, 18.40 Hz, 1H);

^{13}C NMR ($CDCl_3$, 151 MHz): δ 39.9, 48.7, 53.4, 111.7-121.8 (m), 128.4, 129.0, 134.4, 135.6, 153.4-160.9 (q, *J* = 37.9, 37.9, 37.9 Hz), 170.0, 197.5;

^{19}F NMR ($CDCl_3$, 563 MHz): δ -75.88 (s);

HRMS (ESI+): $[M+H]^+ = C_{13}H_{13}F_3NO_4^+$. Calculated m/z 304.0792, found 304.0804.

2,2,2-trifluoro-N-((3*S*)-2-oxo-5-phenyltetrahydrofuran-3-yl)acetamide (S4): methyl-(*S*)-4-oxo-4-phenyl-2-(2,2,2-trifluoroacetamido)butanoate **S3** (377 mg,1.24 mmol, 1.00 equiv) was dissolved in methanol (5 mL) and cooled to -40 °C. Sodium borohydride (56.4 mg, 1.49 mmol, 1.20 equiv) was added portionwise. Reaction was stirred for 2 h, then concentrated under vacuum. The residue was dissolved in EtOAc and water, and the organic layer was washed sequentially with saturated sodium bicarbonate (3x 5 mL) and brine (3x 5 mL). The organic layer was dried over sodium sulfate, filtered, and concentrated to a yellow oil. The oil was purified by DCVC (0-35% EtOAc in hexanes, 20 mL per fraction, incrementing 2.5% EtOAc per fraction). The diastereomers were collected separately and concentrated to white solids (69.8 mg (3*S,*5*R*), 47.9 mg (3*S,*5*S*), 118 mg total, 35% yield). The diastereomers are easily differentiable by the chemical shifts of the C-3 protons.[5]

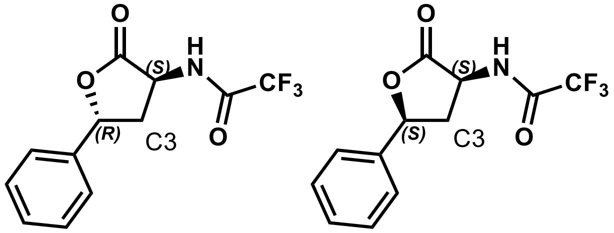

S4

TLC: eluted in 25% EtOAc in hexanes, stained in permanganate. (3*S,*5*R*) R_f = 0.42, (3*S,*5*S*) R_f = 0.30;

(3*S,*5*R*)-**S4** ^1H NMR ($CDCl_3$, 600 MHz): δ 7.42-7.36 (m, 5H), 5.53-5.48 (dd, *J* = 5.20, 11.09 Hz, 1H), 4.83-4.77 (ddd, *J* = 5.68, 8.11, 12.46 Hz, 1H), 3.35-3.26 (ddd, *J* = 5.22, 8.14, 13.09 Hz, 1H), 2.28-2.18 (td, *J* = 11.09, 12.47, 12.48 Hz, 1H);

(3*S*,5*S*)-**S4** ^{1}H NMR ($CDCl_3$, 600 MHz): δ 7.45-7.29 (m, 5H), 5.82-5.78 (d, *J* = 8.32 Hz, 1H), 4.65-4.58 (ddd, *J* = 5.83, 8.76, 11.10 Hz, 1H), 3.05-2.98 (ddd, *J* = 1.77, 8.76, 12.90 Hz, 1H), 2.74-2.66 (ddd, *J*= 8.63, 11.11, 12.88 Hz, 1H);

(3*S*,5*R*)-**S4** ^{13}C NMR ($CDCl_3$, 151 MHz): δ 38.4, 51.4, 79.5, 125.9, 129.2, 129.5, 136.9, 157.9, 172.9;

(3*S*,5*S*)-**S4** ^{13}C NMR ($CDCl_3$, 151 MHz): δ 36.3, 48.6, 78.8, 124.9, 129.0, 129.3, 138.0, 157.8, 173.5;

(3*S*,5*R*)-**S4** LRMS (ESI+): $[M+H]^+ = C_{12}H_{11}F_3NO_3^+$. Calculated m/z: 274.1, found 274.1;

(3*S*,5*S*)-**S4** LRMS (ESI+): $[M+H]^+ = C_{12}H_{11}F_3NO_3^+$. Calculated m/z: 274.1, found 274.1.

(2*S*,4*R*)-2-amino-4-hydroxy-4-phenylbutanoic acid ((2*S*,4*R*)-6): 2,2,2-trifluoro-N-((3*S*,5*R*)-2-oxo-5-phenyltetrahydrofuran-3-yl)acetamide **(3*S*,5*R*)-S4** (69.8 mg, 0.255 mmol, 1.00 equiv) was dissolved in 0.5 mL of methanol and 1 mL of 1 M LiOH. The reaction was stirred at room temperature for 1 h, then lyophilized to a white solid. No starting material remained by NMR.

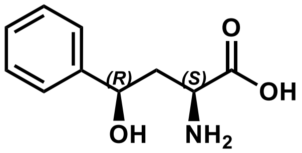

(2*S*,4*R*)-6

^{1}H NMR (D_2O/LiOH, 600 MHz): δ 7.40 – 7.24 (m, 5H), 4.72 (m, 1H), 3.25 (s, 1H), 3.09 (m, 1H), 2.04 – 1.96 (m, 1H), 1.93 – 1.83 (m, 1H);

^{13}C NMR (D_2O/LiOH, 151 MHz): δ 182.8, 143.3, 128.7, 128.0, 126.3, 72.1, 54.0, 48.8, 43.1;

HRMS (ESI+): $[M+H]^+ = C_{10}H_{14}NO_3^+$. Calculated m/z: 196.0969, found 196.0970.

(2*S*,4*S*)-2-amino-4-hydroxy-4-phenylbutanoic acid ((2*S*,4*S*)-6): 2,2,2-trifluoro-N-((3*S*,5*S*)-2-oxo-5-phenyltetrahydrofuran-3-yl)acetamide **(3*S*,5S)-S4** (47.9 mg, 0.175 mmol, 1.00 equiv) was dissolved in 0.5 mL of methanol and 1 mL of 1 M LiOH. The reaction was stirred at room temperature for 1 h, then lyophilized to a white solid. No starting material remained by NMR.

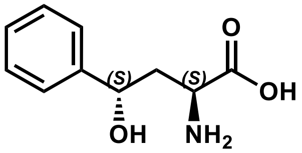

(2*S*,4*S*)-6

^{1}H NMR (D_2O/LiOH, 600 MHz): δ 7.34 – 7.22 (m, 6H), 4.73 – 4.69 (m, 1H), 3.25 – 3.20 (m, 1H), 2.03 (qd, *J* = 7.4, 2.9 Hz, 1H), 1.71 (ddt, *J* = 14.5, 7.7, 3.1 Hz, 1H);

^{13}C NMR (D_2O/LiOH, 151 MHz): δ 183.0, 144.0, 128.7, 127.8, 126.0, 71.2, 53.4, 43.3;

HRMS (ESI+): $[M+H]^+ = C_{10}H_{14}NO_3^+$. Calculated m/z: 196.0969, found 196.0968.

Synthesis of (2*S*,3*R*,4*S*)-**14** and (2*S*,3*R*,4*R*)-**14** standards

Authentic standards of (2*S*,3*R*,4*S*)-**14** and (2*S*,3*R*,4*S*)-**14** were prepared by method of Berkeš et al.[6]

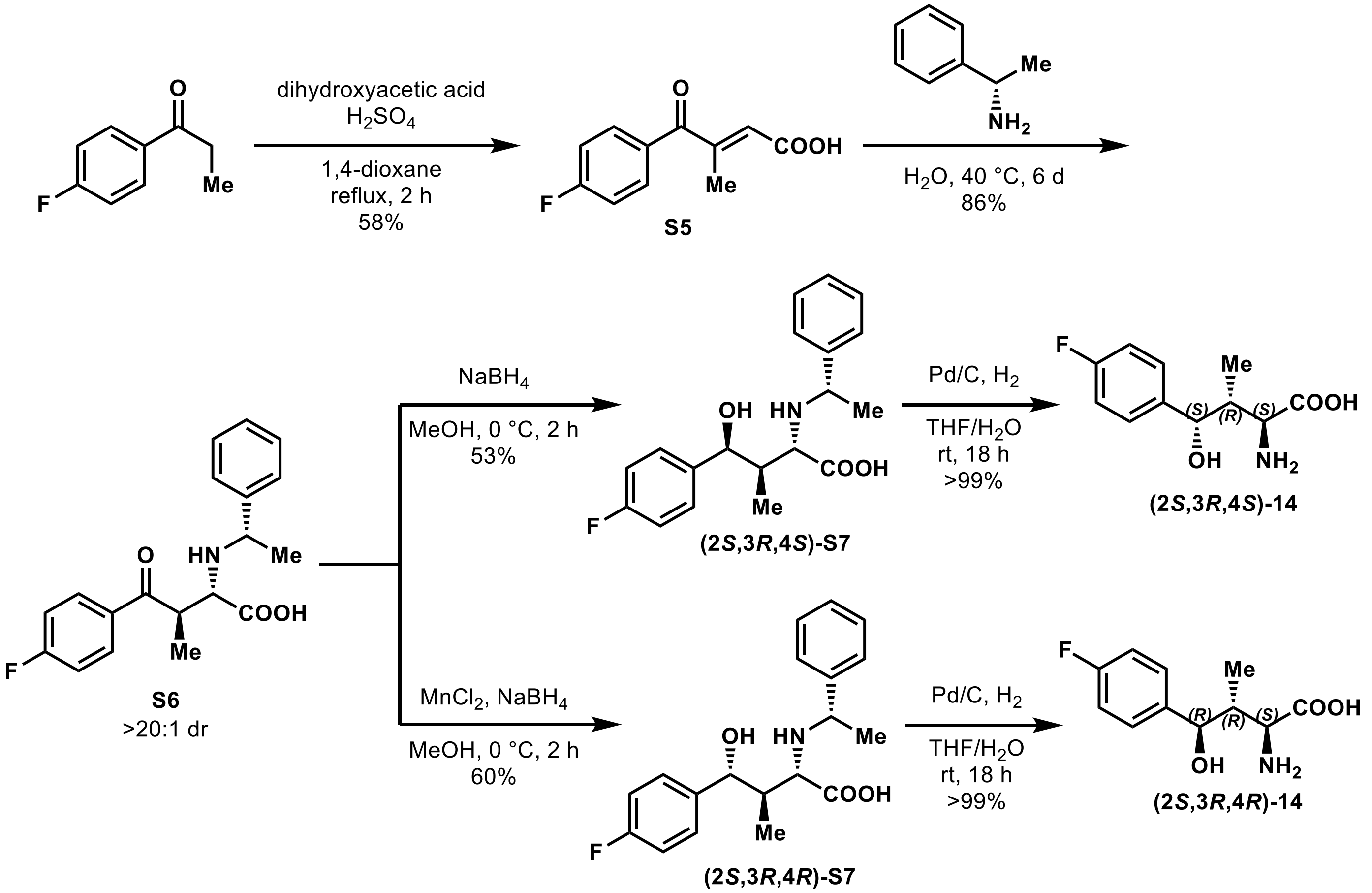

Scheme S10. Synthesis of (2*S*,3*R*,4*S*)-**14** and (2*S*,3*R*,4*S*)-**14**.

(*E*)-4-(4-fluorophenyl)-3-methyl-4-oxobut-2-enoic acid (S5): 4-fluoropropiophenone (2.0 g, 13 mmol, 1.0 equiv) was dissolved in 20 mL 1,4-dioxane. To this solution was added 2,2-dihydroxyacetic acid (1.8 g, 20 mmol, 1.5 equiv) and concentrated sulfuric acid (3.0 mL, 5.5 g, 56 mmol, 4.3 equiv). The reaction was heated to reflux and stirred under nitrogen for 2 h. After cooling to room temperature, the solution was poured into water. The mixture was extracted with EtOAc (3x, 10 mL). The combined organic layers were extracted with saturated sodium bicarbonate (3x, 10 mL). The combined aqueous layers were acidified to pH 2 and extracted with EtOAc (3x, 10 mL). The combined organic layers were dried over anhydrous sodium sulfate and concentrated to a yellow solid. Crude product was recrystallized from EtOAc/hexanes to yield off-white needles (1.6 g, 58% yield).

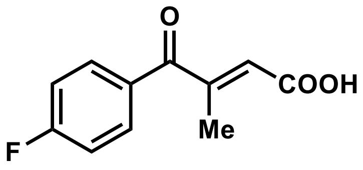

S5

^{1}H NMR ($CDCl_3$, 600 MHz): δ 7.90 – 7.84 (m, 2H), 7.17 (t, *J* = 8.5 Hz, 2H), 6.13 (d, *J* = 1.6 Hz, 1H), 2.44 (d, *J* = 1.5 Hz, 3H);

^{13}C NMR ($CDCl_3$, 151 MHz): δ 196.2, 170.9, 167.0, 165.3, 154.9, 132.7 (d, *J* = 9.5 Hz), 131.8 (d, *J* = 3.0 Hz), 123.5, 116.1 (d, *J* = 22.2 Hz), 16.3.

(2*S*,3*R*)-4-(4-fluorophenyl)-3-methyl-4-oxo-2-(((*S*)-1-phenylethyl)amino)butanoic acid (S6): (*E*)-4-(4-fluorophenyl)-3-methyl-4-oxobut-2-enoic acid **S5** (632 mg, 3.04 mmol, 1.00 equiv) was suspended in 11 mL water and heated to 40 °C. To the stirred suspension was added (*S*)-1-phenylethan-1-amine (0.430 mL, 405 mg, 3.34 mmol, 1.10 equiv). The yellow solution was stirred at 40 °C for 6 d. After 6 d, the white precipitate was filtered and washed with water, then hexanes, to yield an off-white powder. (862 mg, 86% yield).

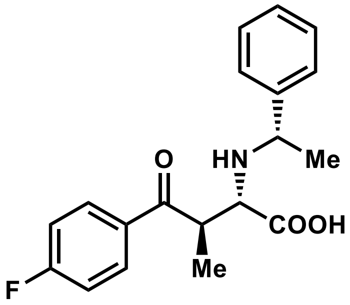

S6

^{1}H NMR ($(CD_3)_2CO$/DCl, 600 MHz) δ 7.94 (t, *J* = 7.1 Hz, 2H), 7.76 (d, *J* = 8.0 Hz, 2H), 7.42 (dt, *J* = 22.8, 7.2 Hz, 3H), 7.16 (t, *J* = 8.5 Hz, 2H), 6.40 (s, 1H), 4.77 (dd, *J* = 6.8, 3.7 Hz, 1H), 4.61 (d, *J* = 6.8 Hz, 1H), 3.89 (d, *J* = 3.5 Hz, 1H), 1.94 (d, *J* = 6.8 Hz, 3H), 1.29 (d, *J* = 6.8 Hz, 3H);

^{13}C NMR ($(CD_3)_2CO$/DCl, 151 MHz): δ 197.3, 167.6, 165.3, 136.4, 132.9, 132.1, 130.4, 130.3, 129.5, 116.5 (d, *J* = 22.1 Hz), 60.2, 42.6, 20.7, 11.3;

^{19}F NMR (563 MHz, $(CD_3)_2CO$/DCl) δ -102.21 (m);

LRMS (ESI+): $[M+H]^+ = C_{19}H_{21}FNO_3^+$. Calculated m/z: 330.0, found 330.1.

(2*S*,3*R*,4*S*)-4-(4-fluorophenyl)-4-hydroxy-3-methyl-2-(((*S*)-1-phenylethyl)amino)butanoic acid ((2*S*,3*R*,4*S*)-S7): (2*S*,3*R*)-4-(4-fluorophenyl)-3-methyl-4-oxo-2-(((*S*)-1-phenylethyl)amino)butanoic acid **S6** (168 mg, 510 μmol, 1.00 equiv) was suspended in 10 mL MeOH, then cooled to 0 °C. $NaBH_4$ (57.9 mg, 1.53 mmol, 3.00 equiv) was added portionwise over 2 h. The reaction was stirred for an additional 30 min after the last addition, then concentrated under vacuum. The residue was dissolved in 5 mL water and the pH was adjusted to pH ~6 with 2 N HCl. The product precipitated as a clear gel, which was filtered and dried under vacuum to yield an off-white solid (89.7 mg, 53% yield. Isolated as a 5:1 mixture of diastereomers).

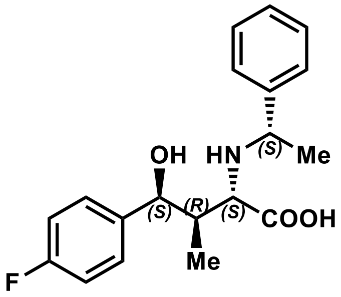

(2*S*,3*R*,4*S*)-S7

^{1}H NMR (D_2O/NaOD, 600 MHz, Major): δ 7.41 – 7.26 (m, 3H), 7.22 (dd, *J* = 6.6, 3.0 Hz, 2H), 6.84 (d, *J* = 7.3 Hz, 4H), 4.62 (d, *J* = 6.6 Hz, 1H), 3.50 (q, *J* = 6.6 Hz, 1H), 2.62 (d, *J* = 6.6 Hz, 1H), 1.99 (h, *J* = 6.8 Hz, 1H), 1.32 (d, *J* = 6.5 Hz, 3H), 0.69 (d, *J* = 7.0 Hz, 3H);

^{13}C NMR (D_2O/NaOH, 151 MHz, Major): δ 180.2, 162.5, 160.9, 143.5, 137.4 (d, *J* = 2.9 Hz), 128.8, 128.6 (d, *J* = 8.2 Hz), 127.6, 127.5, 114.7 (d, *J* = 21.5 Hz), 76.3, 62.7, 56.7, 41.8, 22.9, 12.1;

^{19}F NMR (D_2O/NaOH, 563 MHz, Major): δ -116.31 (p, *J* = 7.4 Hz);

LRMS (ESI+): $[M+H]^+ = C_{19}H_{23}FNO_3^+$. Calculated m/z: 332.2, found 332.2.

(2*S*,3*R*,4*R*)-4-(4-fluorophenyl)-4-hydroxy-3-methyl-2-(((*S*)-1-phenylethyl)amino)butanoic acid ((2*S*,3*R*,4*R*)-S7): (2*S*,3*R*)-4-(4-fluorophenyl)-3-methyl-4-oxo-2-(((*S*)-1-phenylethyl)amino)butanoic acid **S6** (106 mg, 322 μmol, 1.00 equiv) and $MnCl_2 \cdot 4H_2O$ (12.7 mg, 64.4 μmol, 0.200 equiv) were suspended in 6 mL MeOH, then cooled to 0 °C. $NaBH_4$ (36.5 mg, 965 μmol, 3.00 equiv) was added portionwise over 2 h. The reaction was stirred for an additional 30 min after the last addition, then concentrated under vacuum. The residue was dissolved in 1 mL 3 wt% K_2CO_3. The suspension was centrifuged in a 1.5 mL conical tube at 17,000 xg for 5 min and the supernatant was transferred to a clean vial and acidified to pH ~6 with 2 N HCl. The white precipitate was filtered to yield a gel-like solid, which was filtered and dried under vacuum to yield an off-white solid (64.4 mg, 60% yield. Isolated as a 2:1 mixture of diastereomers).

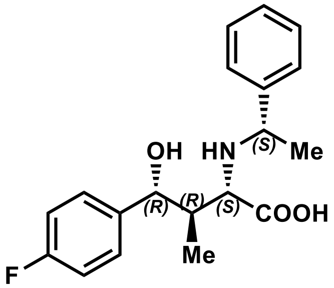

(2*S*,3*R*,4*R*)-S7

^{1}H NMR (D_2O/NaOH, 600 MHz, Major): δ 7.44 – 7.27 (m, 5H), 7.18 (d, *J* = 5.5 Hz, 2H), 7.03 (t, *J* = 8.3 Hz, 2H), 4.28 (d, *J* = 7.0 Hz, 1H), 3.62 (d, *J* = 6.0 Hz, 1H), 2.78 (dd, *J* = 8.4, 3.9 Hz, 1H), 1.86 (d, *J* = 8.7 Hz, 1H), 1.34 (s, 3H), 0.50 (d, *J* = 5.8 Hz, 3H);

^{13}C NMR (D_2O/NaOH, 151 MHz, Major): δ 180.6, 162.8, 161.2, 143.5, 138.1, 128.8, 128.8 (d, *J* = 8.4 Hz), 115.1 (d, *J* = 21.4 Hz), 79.2, 66.0, 56.7, 41.4, 23.3, 12.9;

^{19}F NMR (D_2O/NaOH, 563 MHz, Major): δ -115.50 – -115.66 (m);

LRMS (ESI+): $[M+H]^+ = C_{19}H_{23}FNO_3^+$. Calculated m/z: 332.2, found 332.2.

(2*S*,3*R*,4*S*)-2-amino-4-(4-fluorophenyl)-4-hydroxy-3-methylbutanoic acid ((2*S*,3*R*,4*S*)-14): (2*S*,3*R*,4*S*)-4-(4-fluorophenyl)-4-hydroxy-3-methyl-2-(((*S*)-1-phenylethyl)amino)butanoic acid **(2*S*,3*R*,4*S*)-S7** (17 mg, 51 μmol, 1.0 equiv) was suspended in 1 mL THF, 500 μL water, and acetic acid (5.9 μL, 6.2 mg, 100 μmol, 2.0 equiv). The suspension was degassed with N_2 for 15 min, then Pd/C (1.1 mg, 20 mol %) was added. The reaction was stirred at room temperature overnight under H_2 atmosphere. Reaction was quenched with 100 μL saturated sodium bicarbonate, syringe filtered, then concentrated to a white solid. The residue was dissolved in 1 mL and purified by flash chromatography (reverse phase on CombiFlash, SiliaSep C4 80 g 40-63 μm, 60 Å column, 100% Solvent C for 3 column volumes, then 0-100% Solvent D over 10 column volumes). The fractions were analyzed by LCMS and those containing the product were combined and lyophilized to a white solid (12 mg, >99% yield. Isolated as a 32:1 mixture of diastereomers).

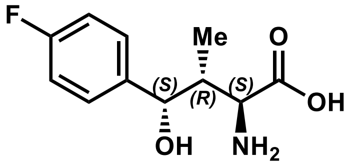

(2*S*,3*R*,4*S*)-14

^{1}H NMR (D_2O/LiOH/FCH_2CN, 600 MHz, Major): δ 7.23 (dd, *J* = 8.4, 5.5 Hz, 2H), 6.98 (t, *J* = 8.7 Hz, 2H), 3.22 (d, *J* = 5.2 Hz, 1H), 1.89 (h, *J* = 7.0 Hz, 1H), 0.51 (d, *J* = 7.0 Hz, 3H);

^{13}C NMR (D_2O/LiOH/FCH_2CN, 151 MHz, Major): δ 181.8, 128.6 (d, J = 8.2 Hz), 115.1, 114.9, 76.6, 58.1, 43.5, 12.2.

^{19}F NMR (D_2O/LiOH/FCH_2CN, 563 MHz, Major): δ -115.65 (td, J = 9.0, 4.7 Hz);

HRMS (ESI+): $[M+H]^+ = C_{11}H_{15}FNO_3^+$. Calculated m/z: 228.1031, found 228.1035

(2*S*,3*R*,4*R*)-2-amino-4-(4-fluorophenyl)-4-hydroxy-3-methylbutanoic acid ((2*S*,3*R*,4*R*)-14): (2*S*,3*R*,4*R*)-4-(4-fluorophenyl)-4-hydroxy-3-methyl-2-(((*S*)-1-phenylethyl)amino)butanoic acid **(2*S*,3*R*,4*R*)-S7** (12 mg, 36 µmol, 1.0 equiv) was suspended in 1 mL THF, 500 µL water, and acetic acid (4.1 µL, 4.3 mg, 72 µmol, 2.0 equiv). The suspension was degassed with N_2 for 15 min, then Pd/C (0.77 mg, 20 mol %) was added. The reaction was stirred at room temperature overnight under H_2 atmosphere. Reaction was quenched with 100 µL saturated sodium bicarbonate, syringe filtered, then concentrated to a white solid. The residue was dissolved in 1 mL and purified by flash chromatography (reverse phase on CombiFlash, SiliaSep C4 80 g 40-63 µm, 60 Å column, 100% Solvent C for 3 column volumes, then 0-100% Solvent D over 10 column volumes). The fractions were analyzed by LCMS and those containing the product were combined and lyophilized to a white solid (8.3 mg, >99% yield. Isolated as a 16:1 mixture of diastereomers).

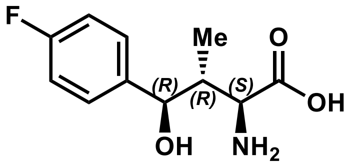

(2*S*,3*R*,4*R*)-14

^{1}H NMR (D_2O/LiOH/FCH_2CN, 600 MHz, Major): δ 7.24 – 7.19 (m, 12), 6.98 (t, *J* = 8.9 Hz, 12), 3.08 (d, *J* = 5.7 Hz, 1H), 1.84 (dq, *J* = 12.8, 6.8 Hz, 1H), 0.65 (d, *J* = 7.0 Hz, 3H);

^{13}C NMR (D_2O/LiOH/FCH_2CN, 151 MHz, Major): δ 181.7, 162.2, 160.6, 138.7, 127.6 (d, *J* = 8.2 Hz), 114.6 (d, *J* = 21.4 Hz), 73.7, 59.0, 43.6, 9.3;

^{19}F NMR (D_2O/LiOH/FCH_2CN, 563 MHz, Major): δ -116.38 (dq, *J* = 13.8, 6.8 Hz);

HRMS (ESI+): $[M+H]^+ = C_{11}H_{15}FNO_3^+$. Calculated m/z: 228.1031, found 228.1041.

Synthesis of (2*S*,3*S*,4*S*)-**14** and (2*R*,3*S*,4*S*)-**14** standards

Synthesis of (2*S*,3*S*,4*S*)-**14** and (2*R*,3*S*,4*S*)-**14** standards was prepared by method of Zimmermann et al.[7]

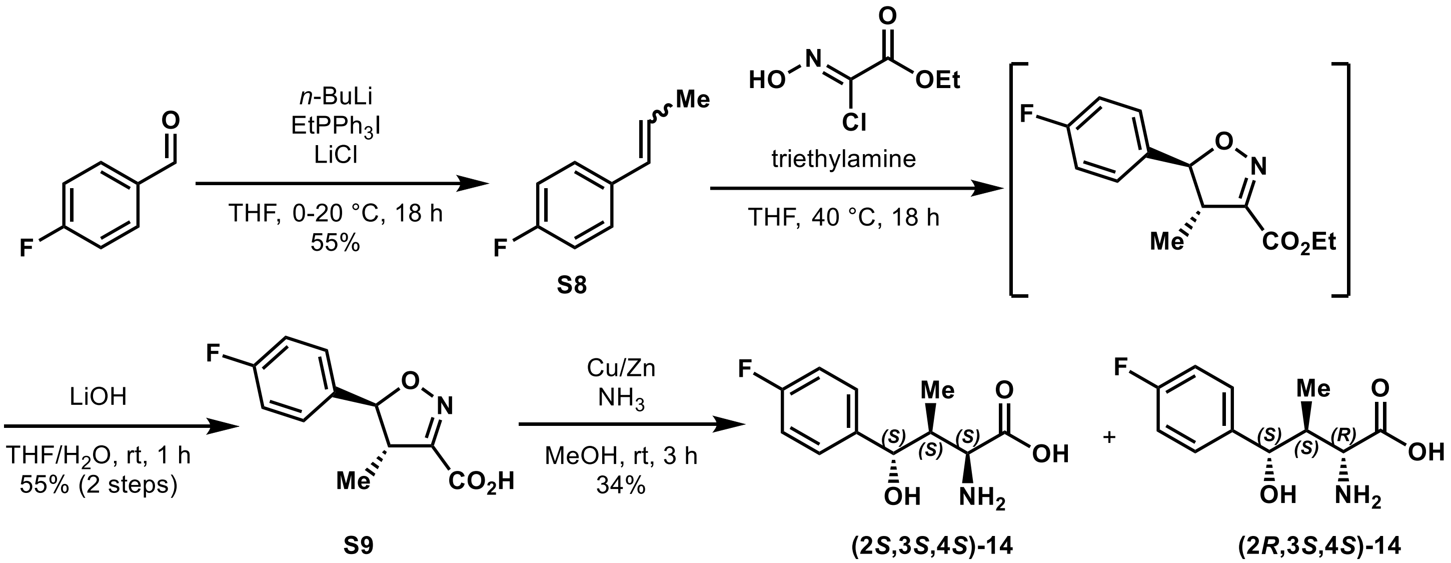

Scheme S11. Synthesis of (2*S*,3*S*,4*S*)-**14** and (2*R*,3*S*,4*S*)-**14**.

1-fluoro-4-(prop-1-ene-1-yl)benzene (S8): $EtPPh_3I$ (20 g, 48 mmol, 1.2 equiv) and LiCl (1.7 g, 40 mmol, 1.0 equiv) were suspended in 100 mL anhydrous THF. The suspension was cooled to 0 °C under N_2. To the stirred suspension was added *n*-BuLi (18 mL of 2.7 M solution in hexanes, 3.1 g, 48 mmol, 1.2 equiv) dropwise. The red suspension was stirred for 30 min. Then, 4-fluorobenzaldehyde (5.0 g, 4.3 mL, 40 mmol, 1.0 equiv) was added dropwise. The reaction turned orange and was stirred at room temperature for 18 h. The reaction was diluted with hexanes (100 mL) and quenched with saturated sodium bicarbonate (25 mL). The reaction was filtered through a silica plug. The organic layer was washed with brine (3x, 25 mL), dried over sodium sulfate, and concentrated *in vacuo* to a yellow liquid. The crude material was re-dissolved in hexanes and passed through another silica plug, eluting with hexanes, and concentrated *in vacuo* to a colorless liquid as a mixture of *E*/*Z* isomers (3.0 g, 55% yield).

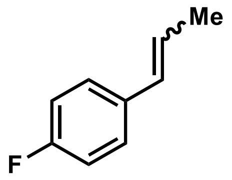

S8

^{1}H NMR (599 MHz, $CDCl_3$) δ 7.32 – 7.24 (mixture of isomers, m, 10H), 7.04 (minor, t, *J* = 8.8 Hz, 2H), 6.99 (major, t, *J* = 8.7 Hz, 8H), 6.45 – 6.35 (mixture of isomers, m, 5H), 6.21 – 6.12 (major, m, 4H), 5.84 – 5.75 (minor, m, 1H), 1.89 (mixture, d, *J* = 7.0 Hz, 15H);

^{13}C NMR (151 MHz, $CDCl_3$) δ 162.8, 162.3, 161.2, 160.7, 134.2 (d, *J* = 3.2 Hz), 130.5 (d, *J* = 7.9 Hz), 130.0, 128.9, 127.3 (d, *J* = 7.9 Hz), 126.8, 125.6 (d, *J* = 2.2 Hz), 18.6, 14.6;

^{19}F NMR (563 MHz, $CDCl_3$) δ -115.9 – -116.0 (m), -116.0 (p, *J* = 7.1 Hz).

(4S,5S)-5-(4-fluorophenyl)-4-methyl-4,5-dihydroisoxazole-3-carboxylic acid (S9): ethyl-(*Z*)-2-chloro-2-(hydroxyamino)acetate (100 mg, 0.660 mmol, 1.00 equiv) was dissolved in 1-fluoro-4-(prop-1-ene-1-yl)benzene **S8** (899 mg, 6.60 mmol, 10.0 equiv) and heated to 40 °C under N_2. To the stirred solution was added a solution of triethylamine (92.0 µL, 66.8 mg, 0.660 mmol, 1.00 equiv) in 20 mL anhydrous THF over 6 h with a syringe pump. The reaction was stirred at 40 °C overnight. The reaction was quenched with 5 mL half-saturated ammonium chloride, extracted with EtOAc (3x, 10 mL), washed with brine (3x, 5mL) dried over sodium sulfate, and concentrated to a clear liquid. The crude material was dissolved in minimal hexanes and passed through a silica plug. Remaining olefin was eluted with hexanes, then crude product was eluted with 25% EtOAc in hexanes. The crude product was concentrated *in vacuo* to a yellow oil and taken to the next step without further purification.

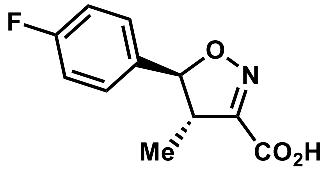

S9

The crude material was dissolved in 2 mL MeOH. To the stirred solution was added 2 mL of 1.5 M LiOH in water. The reaction was stirred at room temperature for 1 h. The colorless solution turned yellow over time. After 1 h, the reaction was directly purified by flash chromatograph (reverse phase on CombiFlash, RediSep Silver C18 86 g 40-63 µm, 60 Å column, 90% Solvent C for 3 column volumes, then 10-100% Solvent D over 10 column volumes). The product was lyophilized to a white solid (81.7 mg, 55% yield over two steps).

^{1}H NMR (599 MHz, CD_3OD) δ 7.4 (dd, *J* = 8.4, 6.0 Hz, 2H), 7.1 (t, *J* = 8.6 Hz, 2H), 5.3 (d, *J* = 7.7 Hz, 1H), 3.4 (p, *J* = 7.4 Hz, 1H), 1.4 (d, *J* = 7.5 Hz, 3H);

^{13}C NMR (151 MHz, CD_3OD) δ 165.1, 163.4, 163.0, 156.8, 137.0 (d, *J* = 3.1 Hz), 129.0 (d, *J* = 8.6 Hz), 116.6 (d, *J* = 21.8 Hz), 92.8, 51.2, 17.3;

^{19}F NMR (563 MHz, CD_3OD) δ -115.4 – -115.9 (m);

LRMS (ESI+): $[M+H]^+$ = $C_{11}H_{11}FNO_3^+$. Calculated m/z: 224.1, found 224.1.

(2S,3S,4S)-2-amino-4-(4-fluorophenyl)-4-hydroxy-3-methylbutanoic acid ((2*S*,3*S*,4*S*)-14): Zn powder (30 mg) was suspended in 1 mL concentrated ammonium hydroxide. To the stirred suspension was added $CuSO_4 \cdot 5H_2O$. The solids were allowed to settle and the suspension was decanted. The solids were washed with 7 M NH_3 in MeOH (5x, 1 mL) before finally suspended in 1 mL of 7 M NH_3 in MeOH. This suspension was added to a solution of (4S,5S)-5-(4-fluorophenyl)-4-methyl-4,5-dihydroisoxazole-3-carboxylic acid **S9** (17.2 mg, 77.1 µmol, 1.00 equiv) in 2 mL of 7 M NH_3 in MeOH. The reaction was stirred at room temperature for 3 h and the solution turned clear blue. The reaction was concentrated *in vacuo*, then re-dissolved in water. The suspension was acidified to pH ~5 until fully dissolved, then purified by flash chromatography (reverse phase on CombiFlash, SiliaSep C4 80 g 40-63 µm, 60 Å column, 100% Solvent C for 3 column volumes, then 0-100% Solvent D over 10 column volumes). The product was lyophilized to a white solid as a 2:1 mixture of diastereomers (major (2*S*,3*S*,4*S*), minor (2*R*,3*S*,4*S*), 6.0 mg, 34% total yield).

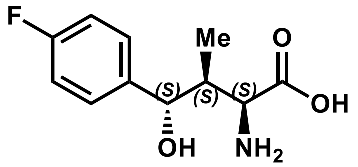

(2*S*,3*S*,4*S*)-14

Major ^{1}H NMR (599 MHz, D_2O/LiOH) δ 7.2 (q, *J* = 6.3 Hz, 2H), 7.0 (t, *J* = 8.9 Hz, 2H), 4.3 (d, *J* = 9.2 Hz, 1H), 3.5 (s, 1H), 2.2 – 2.1 (m, 1H), 0.4 (d, *J* = 7.0 Hz, 3H);

Minor ^1H NMR (599 MHz, D_2O/LiOH) δ 7.2 (q, *J* = 6.3 Hz, 2H), 7.0 (t, *J* = 8.9 Hz, 2H), 4.5 (d, *J* = 9.5 Hz, 1H), 3.2 (d, *J* = 5.3 Hz, 1H), 1.9 – 1.8 (m, 1H), 0.5 (d, *J* = 7.0 Hz, 3H);

Major ^{13}C NMR (151 MHz, D_2O/LiOH): δ 182.7, 128.6 (d, *J* = 8.2 Hz), 115.2 (d, *J* = 21.6 Hz), 75.6, 55.4, 42.3, 10.1;

Minor ^{13}C NMR (151 MHz, D_2O/LiOH): δ 181.9, 138.9, 115.0, 76.8, 58.2, 43.4, 12.2;

Major ^{19}F NMR (563 MHz, D_2O/LiOH) δ -115.6 – -115.6 (m);

Minor ^{19}F NMR (563 MHz, D_2O/LiOH) δ -115.7 (m);

HRMS (ESI+): $[M+H]^+$ = $C_{11}H_{15}FNO_3^+$. Calculated m/z: 228.1031, found 228.1043.

Synthesis of (2S,3S,4R)-**14** standard

Synthesis of (2*S*,3*S*,4*R*)-**14** standard was prepared by method of Cao et al.[8]

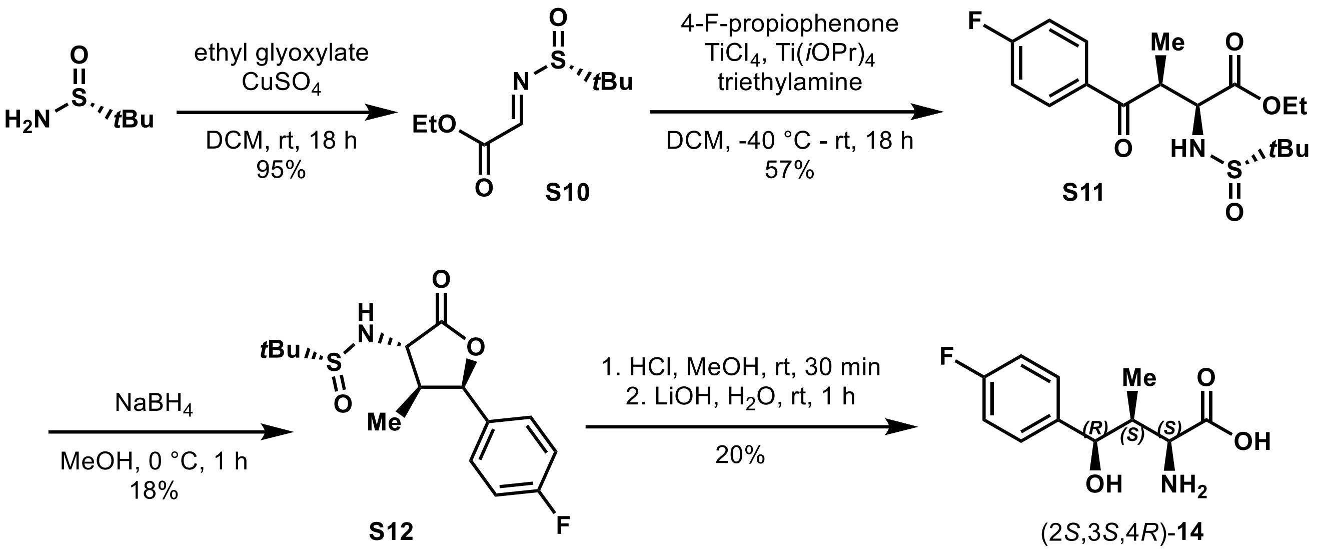

Scheme S12. Synthesis of (2*S*,3*S*,4*R*)-**14**.

Ethyl-(*R*,*E*)-2-((*tert*-butylsulfinyl)imino)acetate (S10): ethyl glyoxylate (50 wt% in toluene, 727 mg, 7.12 mmol, 1.50 equiv) was depolymerized by heating with a heat gun for 1 min. The mixture was dissolved in 15 mL anhydrous DCM and anhydrous $CuSO_4$ (1.51 g, 9.49 mmol, 2.00 equiv)was added. The the stirred suspension, under N_2, was added (*R*)-2-methylpropane-2-sulfinamide (575 mg, 4.74 mmol, 1.00 equiv). The reaction was stirred at room temperature for 18 h and the suspension turned blue over time. The reaction was filtered through celite and the filtrate was concentrated *in vacuo* to a yellow solid. The mixture was purified by flash chromatography (normal phase on Biotage, 25 g column, 0-100% EtOAc over 20 column volumes) and concentrated to a yellow oil (929 mg, 95% yield).

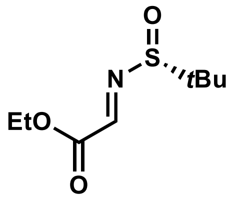

S10

^{1}H NMR (599 MHz, $CDCl_3$) δ 7.5 (s, 1H), 7.3 (d, *J* = 7.6 Hz, 2H), 7.2 (s, 1H), 7.1 (t, *J* = 7.7 Hz, 2H), 7.1 – 7.0 (m, 1H), 4.1 (q, *J* = 6.6 Hz, 1H), 4.0 (qd, *J* = 7.1, 4.7 Hz, 2H), 1.3 (d, *J* = 6.6 Hz, 3H), 0.9 (t, *J* = 7.1 Hz, 3H);

^{13}C NMR (151 MHz, $CDCl_3$) δ 163.4, 152.0, 144.0, 128.8, 127.5, 127.0, 70.3, 61.2, 24.8, 14.1

HRMS (ESI+): $[M+H]^+ = C_8H_{16}NO_3S^+$. Calculated m/z: 206.0846, found 206.0849.

Ethyl-(2S,3S)-2-(((S)-*tert*-butylsilfinyl)amino)-4-(4-fluorophenyl)-3-methyl-4-oxobutanoate (S11): Ti(*i*OPr)4 (234 µL, 224 mg, 0.788 mmol, 0.280 equiv) was added dropwise to a solution of TiCl4 (291 µL, 448 mg, 2.36 mmol, 0.840 equiv) in 20 mL anhydrous DCM at -40 °C. Then, 4-F-propiophenone (391 µL, 428 mg, 2.81 mmol, 1.00 equiv) was added dropwise, followed by triethylamine (431 µL, 313 mg, 3.09 mmol, 1.10 equiv). The reaction was stirred under N_2 for 30 min at -40 °C. Then, a solution of ethyl-(*R*,*E*)-2-((*tert*-butylsulfinyl)imino)acetate **S10** (866 mg, 4.22 mmol, 1.5 equiv) in 3 mL anhydrous DCM was added. The reaction was stirred overnight allowing to

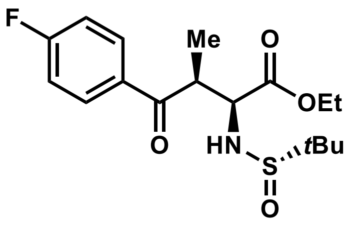

S11

warm to room temperature. The reaction was quenched with saturated sodium bicarbonate (10 mL) and stirred vigorously for 15 min. A white precipitate formed, which was removed by filtration. The filtrate was extracted with EtOAc (3x, 15 mL). The organic layers were combined and washed with brine (3x, 10 mL), dried over sodium sulfate, and concentrated *in vacuo*. The crude material was purified by DCVC (0-100% EtOAc in hexanes, 20 mL per fraction, incrementing 2.5% EtOAc per fraction). Product was concentrated *in vacuo* to an orange solid as a 2.5:1 mixture of diastereomers (577 mg, 57% total yield).

TLC: Eluted in 2:1 hexanes:EtOAc, stained in permanganate. R*f*: 0.3 (yellow spot);

^{1}H NMR (599 MHz, $CDCl_3$) δ 8.0 – 7.9 (m, 12), 7.2 – 7.1 (m, 2H), 4.3 (dd, *J* = 7.3, 5.5 Hz, 1H), 4.3 – 4.2 (m, 3H), 4.1 (d, *J* = 7.4 Hz, 1H), 3.9 – 3.9 (m, 1H), 1.3 – 1.2 (m, 6H), 1.2 (s, 9H), 1.1 (s, 3H);

^{13}C NMR (151 MHz, $CDCl_3$) δ 199.4, 198.6, 172.1, 171.8, 166.7 (d, *J* = 6.6 Hz), 165.0 (d, *J* = 6.6 Hz), 132.7 (d, *J* = 3.1 Hz), 131.2 (d, *J* = 9.3 Hz), 131.0 (dd, *J* = 9.4, 6.9 Hz), 116.0 (dd, *J* = 21.9, 7.4 Hz), 62.2, 61.9, 59.6, 56.3, 44.5, 44.2, 22.6, 22.5, 14.5, 14.0, 12.7;

^{19}F NMR (563 MHz, $CDCl_3$) δ -104.5 – -104.5 (m), -104.6 (h, *J* = 7.5 Hz);

HRMS (ESI+): $[M+H]^+$ = $C_{17}H_{25}FNO_4S^+$. Calculated m/z 358.1483, found 358.1473.

Ethyl (2*S*,3*S*)-2-(((*S*)-*tert*-butylsulfinyl)amino)-4-(4-fluorophenyl)-3-methyl-4-oxobutanoate (S12):

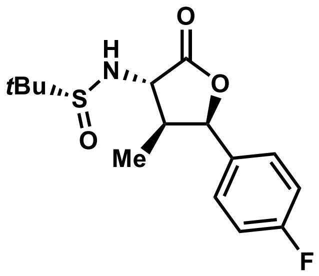

S12

ethyl-(2S,3S)-2-(((S)-*tert*-butylsilfinyl)amino)-4-(4-fluorophenyl)-3-methyl-4-oxobutanoate **S-11** (47 mg, 0.13 mmol, 1 equiv) was dissolved in 5 mL MeOH and cooled to 0 °C. To the stirred solution, under N_2, was added $NaBH_4$ (15 mg, 0.39 mmol, 3.0 equiv). The reaction was stirred for 1 h at 0 °C. The reaction was quenched with brine and extracted with EtOAc (3x, 5 mL). The organic layers were combined and washed with brine (10 mL), dried over sodium sulfate, and concentrated to a clear oil. The crude material was purified by flash chromatography (normal phase, 0-100% EtOAc) and concentrated to a clear oil (7.4 mg, 18% yield).

TLC: Eluted in 2:1 EtOAc:hexanes, stained in CAM. R_f = 0.23 (blue spot). Note that the product does not appear under UV;

^{1}H NMR (599 MHz, $CDCl_3$) δ 7.2 – 7.1 (m, 1H), 5.6 (d, *J* = 8.1 Hz, 0H), 3.9 (dd, *J* = 10.9, 7.9 Hz, 0H), 2.8 (dp, *J* = 10.6, 7.1 Hz, 0H), 1.3 (s, 2H), 1.0 (d, *J* = 6.9 Hz, 1H);

^{13}C NMR (151 MHz, $CDCl_3$) δ 174.8, 127.7 (d, *J* = 8.5 Hz), 116.0 (d, *J* = 21.8 Hz), 81.4, 60.2, 57.2, 42.6, 22.6, 13.6;

^{19}F NMR (563 MHz, $CDCl_3$) δ -112.9 – -112.9 (m);

LRMS (ESI+): $[M+H]^+$ = $C_{15}H_{21}FNO_3S^+$. Calculated m/z: 314.1, found 314.1.

(2*S*,3*S*,4*R*)-2-amino-4-(4-fluorophenyl)-4-hydroxy-3-methylbutanoic acid ((2*S*,3*S*,4*R*)-14): ethyl (2*S*,3*S*)-2-(((*S*)-*tert*-butylsulfinyl)amino)-4-(4-fluorophenyl)-3-methyl-4-oxobutanoate **S-12** (8.1 mg, 26 μmol, 1.0 equiv) was dissolved in 1 mL MeOH. 1 M HCl (52 μL, 1.9 mg, 52 μmol, 2.0 equiv) was added. The reaction was stirred at room temperature for 30 min, then concentrated *in vacuo*. The residue was dissolved in 0.5 mL water and 1 M LiOH (0.13 mL, 3.1 mg, 0.13 mmol, 5.0 equiv) was added. The reaction was stirred at room temperature for 1 h until completely dissolved. The pH was adjusted to ~7 with glacial acetic acid, then purified directly by preparative HPLC (Kinetex 5 μm C18 100 Å column, 100% Solvent C for 5 min, then 0-5% Solvent D over 30 min). Product was lyophilized to a white solid (0.9 mg, 20% yield).

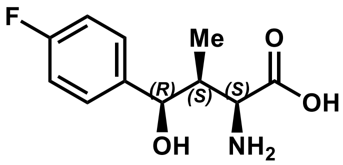

(2*S*,3*S*,4*R*)-14

^{1}H NMR (599 MHz, D_2O) δ 7.4 – 7.4 (m, 1H), 7.2 (t, *J* = 8.9 Hz, 1H), 4.7 – 4.6 (m, 1H), 3.0 (d, *J* = 3.1 Hz, 1H), 2.3 (dddd, *J* = 10.0, 7.9, 6.9, 3.1 Hz, 0H), 0.9 (d, *J* = 6.9 Hz, 1H);

^{13}C NMR (151 MHz, D_2O) δ 182.1, 176.3 (d, *J* = 18.6 Hz), 162.8, 161.2, 138.4 (d, *J* = 3.2 Hz), 128.3 (d, *J* = 8.3 Hz), 115.2 (d, *J* = 21.3 Hz), 79.9, 78.7, 76.3, 57.5, 42.8, 8.7;

^{19}F NMR (563 MHz, D_2O) δ -115.52 (ddd, *J* = 14.82, 9.48, 5.53 Hz);

HRMS (ESI+): $[M+H]^+ = C_{11}H_{15}FNO_3^+$. Calculated m/z: 228.1031, found 228.1040.

Computational Details

General Computational Details

All calculations and simulations were performed on resources provided and maintained by the Center for High Performance Computing (CHPC) at the University of Utah. All singlepoint computations were performed using either xTB (VERSION NUMBER) or Gaussian 16 (VERSION NUMBER). All simulations were performed using the AMBER simulation suite on NVIDIA H100 graphics processing units.

Preparation of Docking Hosts for Aro8 and TyrB

The crystal structure aromatic aminotransferase Aro8 (PDB 4JE5) was downloaded from the Protein Data Bank and repaired by adding missing residues using the CHARMM-GUI PDB Reader & Manipulator. Chains A and B were extracted from the repaired PDB file along with the cocrystals PLP (chain A) and PMP (chain B). PDB entries from the buffer solution (EPE) as well as crystallographic waters were removed. Following the repair procedure, the PDB file was subjected to the *pdb4amber* (AmberTools version 23.6) command line tool followed by *tleap* (AmberTools version 23.6) to add explicit hydrogens and atoms unresolved in the crystal structure. The protonation state of Aro8 was estimated using the *PROPKA* program within the *PDB2PQR* command line tool (version 3.6.2). PLP and PMP were removed from the structure before protonation state estimation at pH 8.0 with AMBER formatting for residue and atom names. The resultant structure was minimized (imin=1) for 1,500 cycles of steepest descent followed by 1,500 conjugate gradient steps. The generalized-Born implicit solvent (igb=1) and an infinite nonbonded interaction cutoff were employed with no positional restraints. An identical procedure was used for TyrB (PDB 3TAT).

Flexible Docking of Ketimines to Aro8 (PDB 4JE5)

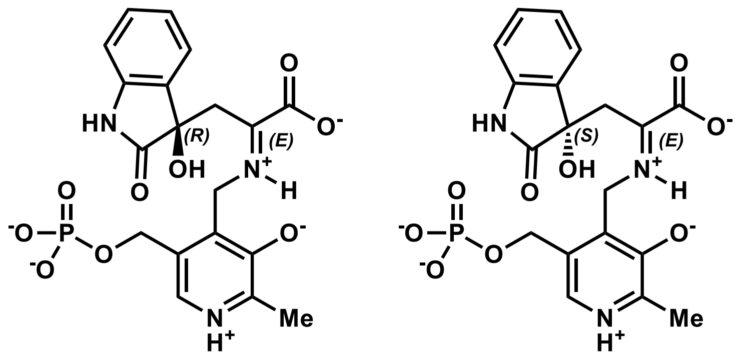

Figure S28. Structures and 3-letter PDB codes used for docking.

The initial 3D structures of the guests (in Tripos Mol2 format) were generated from SMILES using *OpenBabel* (version 3.1.0) and optimized using the xTB program (version 6.6.1) with the GFN2-xTB method. The guests were converted to the PDBQT format required for AutoDock Vina with the *prepare_ligand* command line tool provided by ADFRsuite (version 1.0). The receptor was prepared similarly with the *mk_prepare_receptor.py* from the Meeko Python package. Residues within 6 Å of the centroid of the PLP cocrystal were designated as flexible during the docking procedure except for Ala, Pro, and Gly residues (Table S1). The geometric centroid of the co-crystallized PMP was used as the docking center with box edges of 20.0 Å in all dimensions. The default AutoDock Vina settings were modified with a grid spacing of 0.275 Å, exhaustiveness of 32, and a minimum RMSD between poses of 0.25 Å. The resulting poses and flexible residues were combined with the remaining rigid portion of the docking host and minimized (imin=1) for 1,500 cycles of steepest descent followed by 1,500 conjugate gradient steps. The generalized-Born implicit solvent (igb=1) and an infinite nonbonded interaction cutoff and no positional restraints.

Table S6. Residues designated as flexible during docking procedure for Aro8

Res. Num.	Chain	Res	Res. Num.	Chain	Res
141	A	Asn	251	A	Tyr
142	A	Thr	302	A	Ser
143	A	Asn	304	A	Ser
166	A	Phe	305	A	Lys
169	A	Ser	312	A	Arg
213	A	Tyr	399	A	Phe
215	A	Ile	105	B	Tyr
220	A	Asn	335	B	Gln
248	A	Asp			

The large box size produces many high-scoring poses that do not preserve interactions observed in the crystal structure. To identify likely poses, candidate poses were compared to the crystal structure to identify the minimum RMSD pose for the docked guest. The RDKit Python package (version 2024.3.5) was first used to identify the maximum common substructure between the guest pose and PLP from the crystal structure (Figure S19).

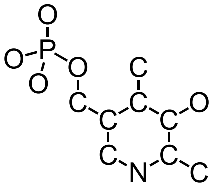

Figure S29. Maximum common substructure between PMP and ketimine intermediates.

The common substructure was used to construct an atom map between the guest pose and crystal structure PLP. The RDKit function *CalcRMS* was used to compute the RMSD of between the two species to identify the pose that aligns most closely to PMP in the crystal structure.

Flexible Docking of Ketimines to TyrB (PDB 3TAT)

Docking of guests to TyrB was performed using the same method described for Aro8. A different set of flexible residues were permitted to move flexible during the docking procedure (Table S2).

Table S7. Residues designated as flexible during docking procedure for TyrB

Res. Num.	Chain	Res	Res. Num.	Chain	Res
102	A	Thr	184	A	Ser
103	A	Ile	212	A	Leu
104	A	Gln	214	A	Ala
105	A	Thr	215	A	Arg
106	A	Leu	255	A	Ser
134	A	Trp	349	A	Asn
179	A	Thr			

Preparation of Forcefield Parameters for Nonstandard Residues

Nonstandard residue forcefield parameters were defined using GAFF2 atom types and parameters. The *antechamber* command line tool was used to compute AM1-BCC partial charges and determine atom types.

Preparing and relaxing explicitly solvated Aro8 and TyrB

GaMD simulations were performed using a modified reported procedure for both Aro8 and TyrB. The starting structure was generated by identifying the minimum RMSD pose of the aminoacrylate relative to the cocrystallized PMP residue using the previously described flexible docking procedure. The electrophile (isatin) was then added to the combined structure of the enzyme and aminoacrylate using the same procedure except the greatest affinity pose was selected. The residue protonation states of the enzyme docked with both aminoacrylate and isatin were determined using *PROPKA* program within the *PDB2PQR* command line tool (version 3.6.2). Within the tleap program, the library files from the previously described procedure for preparing forcefield parameters for nonstandard residues were loaded alongside the protein.ff19SB forcefield. The tleap program was used to solvate the structure in a 12 Å truncated octahedron using the OPC water model, and the system was neutralized with Na^+ ions using the addions2 command. The final coordinate and topology files were exported for equilibration.

The system was relaxed using a 9-step protocol described in the AMBER tutorials.

1. Minimization (cycles = 1,000) with the protein and cofactor atoms fixed using a 100 kcal mol^{-1} $Å^{-2}$ positional restraint.
2. Heating to 298.15 K using 1,000,000 steps (1 fs time step) with the protein and cofactor atoms fixed using a 100 kcal mol^{-1} $Å^{-2}$ positional restraint.
3. Constant pressure MD simulation using the Monte Carlo barostat for 1,000,000 steps (1 fs time step) with a 10 kcal mol^{-1} $Å^{-2}$ positional restraint on the protein and cofactor atoms.
4. Constant pressure MD simulation using the Monte Carlo barostat for 1,000,000 steps (1 fs time step) with a 10 kcal mol^{-1} $Å^{-2}$ positional restraint on the protein and cofactor atoms.
5. Minimization (cycles = 1,000) with a 10 kcal mol^{-1} $Å^{-2}$ positional restraint on only the backbone atoms of the protein (atoms @CA,N,C).
6. Constant pressure MD simulation using the Monte Carlo barostat for 1,000,000 steps (1 fs time step) with a 10 kcal mol^{-1} $Å^{-2}$ backbone constraint (atoms @CA,N,C).
7. Constant pressure MD simulation using the Monte Carlo barostat for 1,000,000 steps (1 fs time step) with a 1 kcal mol^{-1} $Å^{-2}$ backbone constraint (atoms @CA,N,C).
8. Constant pressure MD simulation using the Monte Carlo barostat for 1,000,000 steps (1 fs time step) with a 0.1 kcal mol^{-1} $Å^{-2}$ backbone constraint (atoms @CA,N,C).
9. Constant pressure MD simulation using the Monte Carlo barostat for 1,000,000 steps (1 fs time step) with no positional restraints.

Gaussian accelerated molecular dynamics (GaMD)

Following relaxation, gaussian accelerated molecular dynamics was performed using the dual-boost scheme (igamd=3) with the threshold energy mode (iE) was set to 1. The following formulas were used to determine the equilibration length. The equilibration procedure proceeds by first performing a conventional MD simulation (ntcmdprep) to equilibrate the system, a second conventional MD simulation to collect statistics for computing boosts (ntcmd), and a GaMD equilibration period in which boost parameters are computed (nteb). The following formulas were used to compute the required settings for both the Aro8 and TyrB systems.

natoms = number of protein atoms in the system

natoms_max = total number of atoms in the system

ntave = 4 * natoms_max

ntcmd = 10 * ntave

ntcmdprep = 2 * ntave

ntebprep = 2 * ntave

nteb = 40 * ntave

nstlim = nteb + ntcmd

The systems were equilibrated using the above GaMD settings and upper bounds of the standard deviation of the boosts set to 6.0 kcal mol^{-1}. A timestep of 0.002 ps was used along with SHAKE control ntc=2 and ntf=2 with a 1e-5 relative geometric tolerance for resetting SHAKE. The system was simulated under constant pressure at 1.0 bar using the Monte Carlo barostat with isotropic scaling.

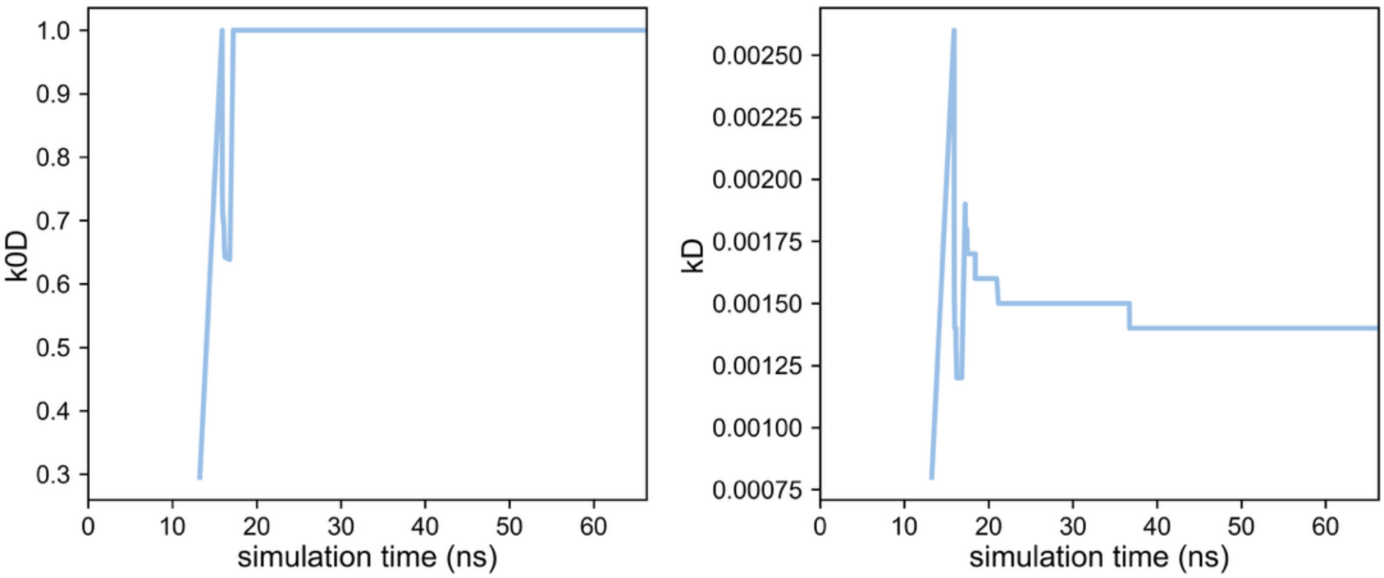

Figure S30. Convergence of dihedral boost parameters for Aro8 over 66 ns.

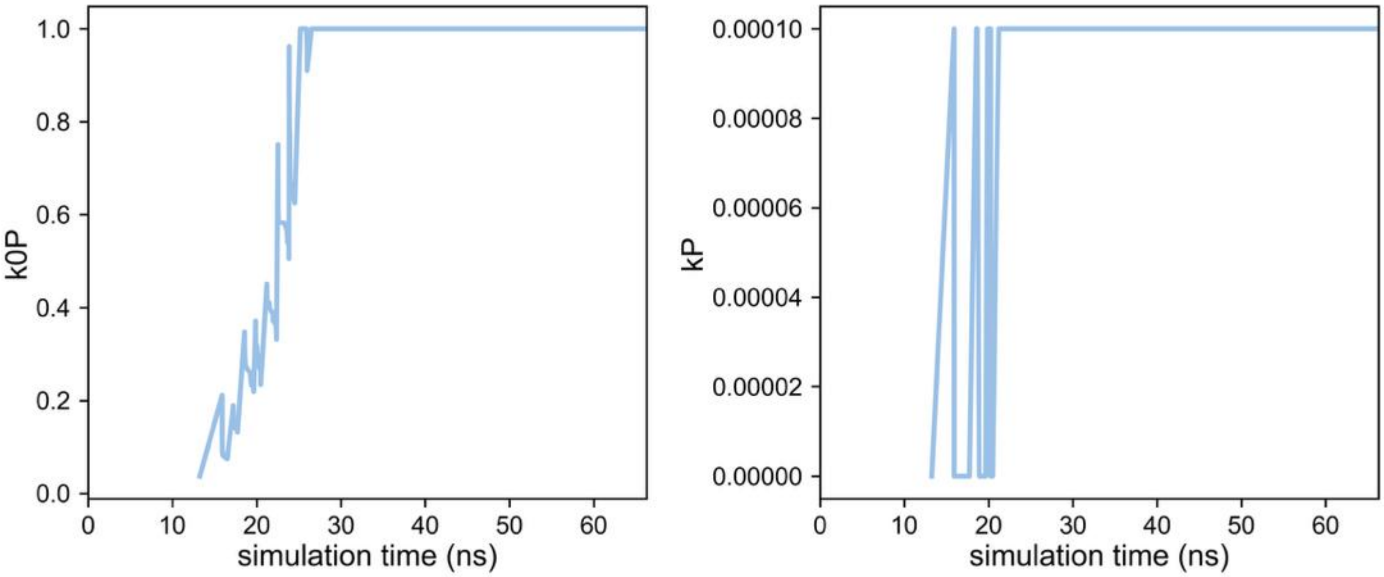

Figure S31. Convergence of total potential boost parameters for Aro8 over 66 ns.

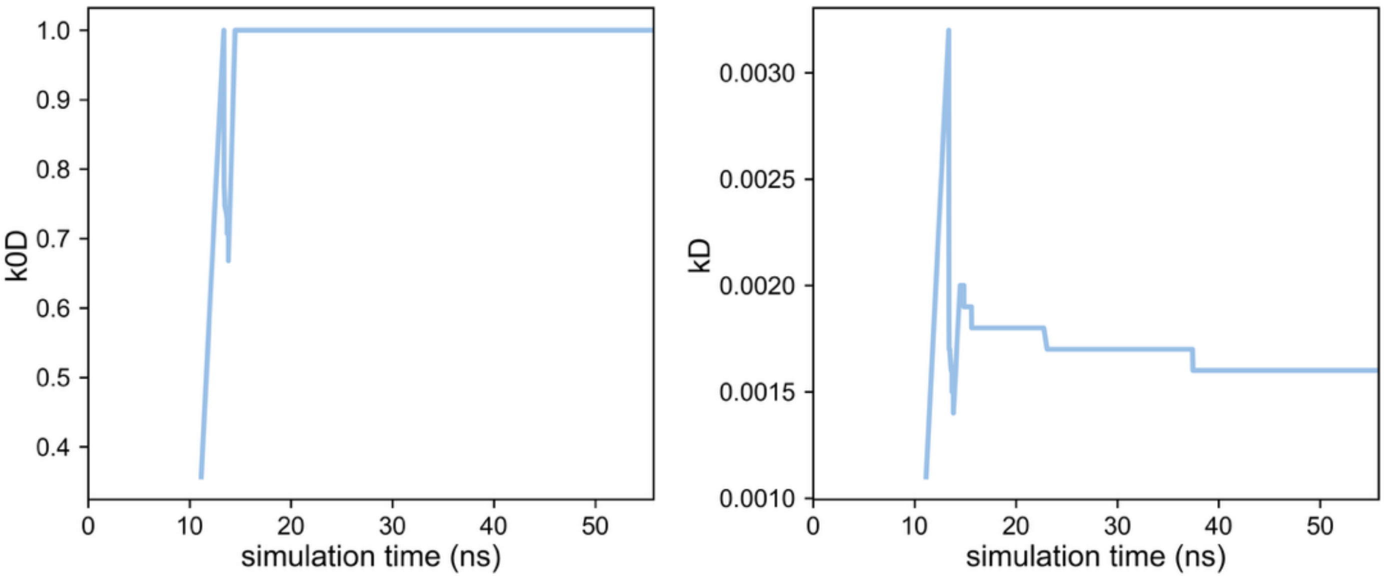

Figure S32. Convergence of dihedral boost parameters for TyrB over 60 ns.

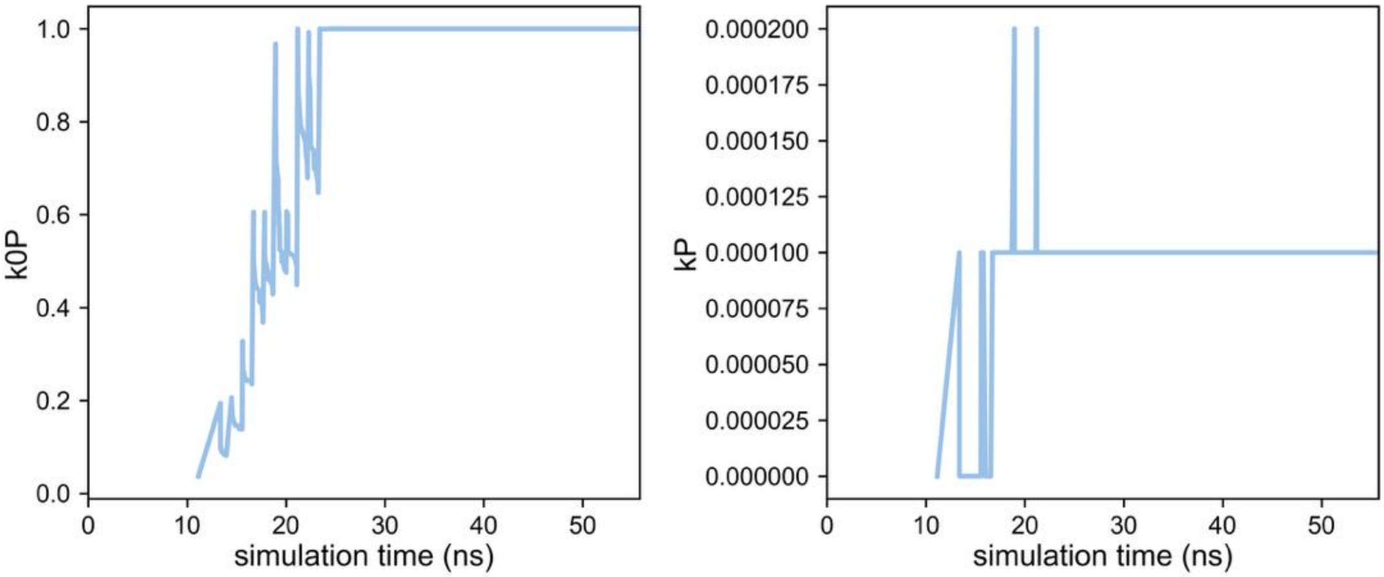

Figure S33. Convergence of total potential boost parameters for TyrB over 60 ns.

Following equilibration, the final coordinates of the equilibration run were used to start the production GaMD run (200 ns, 0.002 ps time step) with the same settings as the equilibration. In addition to the GaMD boost, a single flat-welled parabolic restraint was added between the two reacting atoms of the aminoacrylate and isatin to prevent the electrophile from diffusing out of the active site. The r1, r2, r3, and r4 values for the potential were 0.0 Å, 2.0 Å, 6.5 Å, and 8.0 Å, respectively. The rk2 and rk3 values were both 10.0 kcal mol^{-1} $Å^{-1}$. Features from the production run were computed using the MDAnalysis Python package by iterating over the frames of the simulation and extracting the coordinates of the reacting atoms. These atomic positions were used to compute the distance and attack angle descriptors that reflect the facial selectivity. Replicate simulations with different random seeds were performed for both enzymes and were in good agreement with each other.

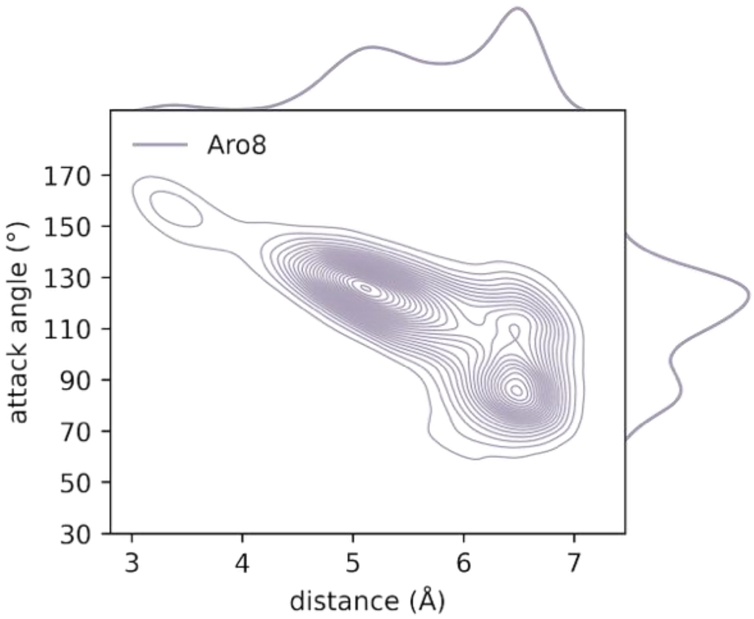

Figure S34. Topographical plot of GaMD simulation for Aro8 replicate 1.

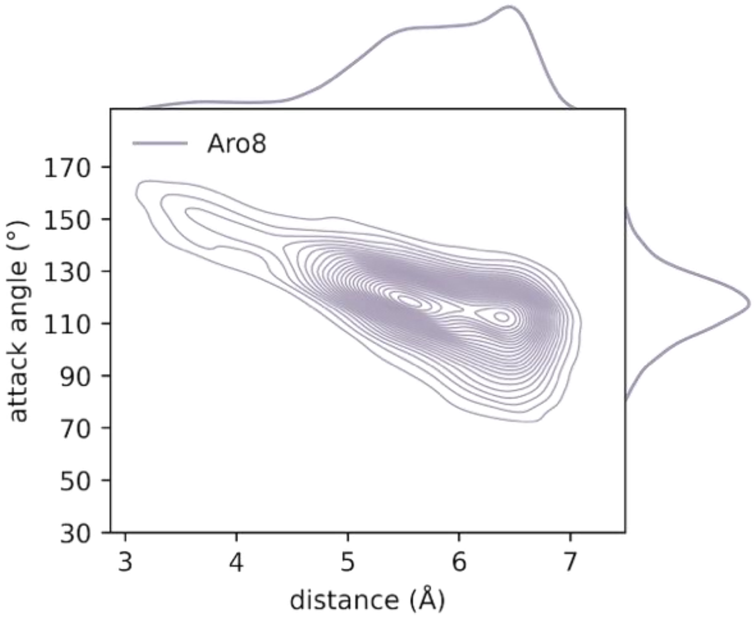

Figure S35. Topographical plot of GaMD simulation for Aro8 replicate 2.

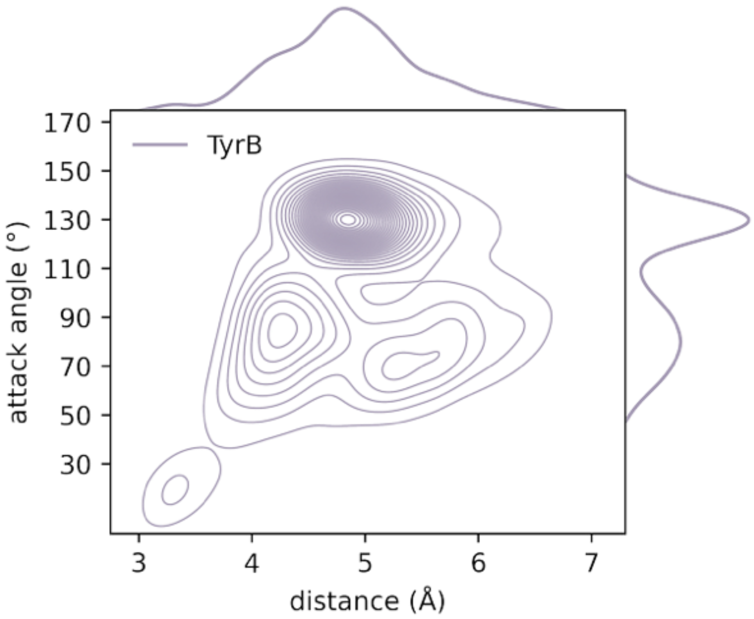

Figure S36. Topographical plot of GaMD simulation for TyrB replicate 1.

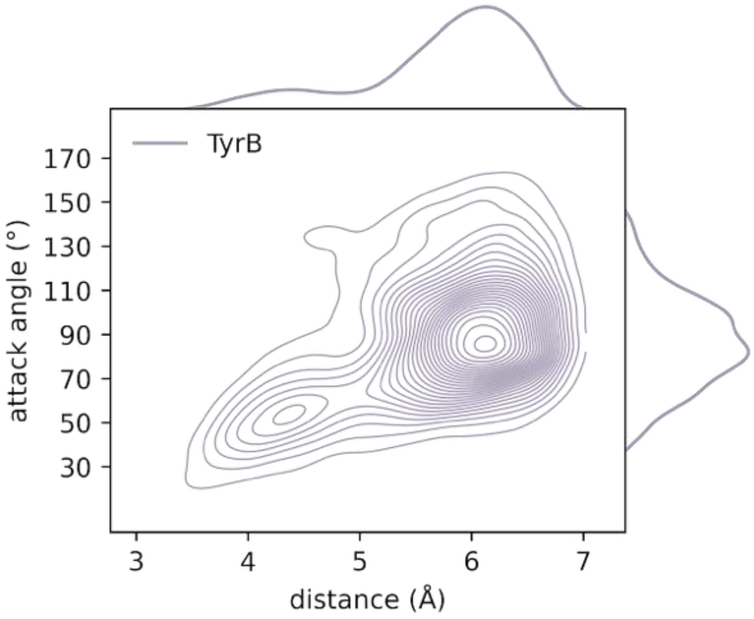

Figure S37. Topographical plot of GaMD simulation for TyrB replicate 2.

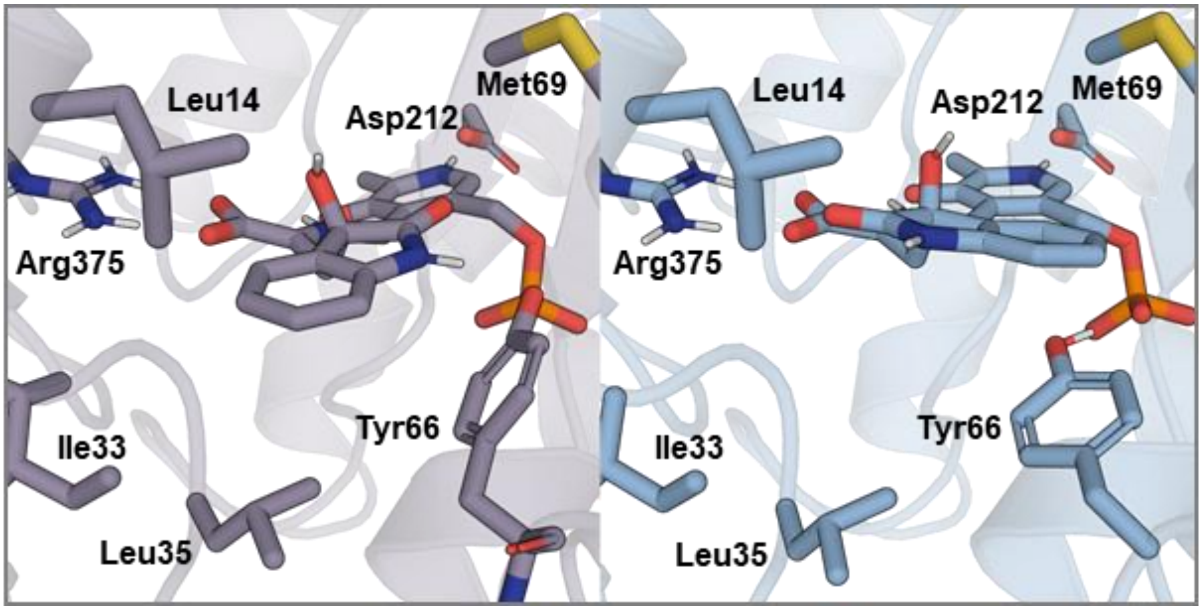

Figure S38. Active site of TyrB with minimum RMSD docked poses of ketimines (*R*)-**VI** (left) and (*S*)-**VI** (right).

References

1. *Rowan Scientific*. https://www.rowansci.com (accessed 2025-04-10).
2. Anstine, D.; Zubatyuk, R.; Isayev, O. AIMNet2: A Neural Network Potential to Meet your Neutral, Charged, Organic, and Elemental-Organic Needs. *Chem. Sci.* **2025**, *16* (23), 10228-10244.
3. Graboski, A. L.; Kowalewski, M. E.; Simpson, J. B.; Cao, X.; Ha, M.; Zhang, J.; Walton, W. G.; Flaherty, D. P.; Redinbo, M. R. Mechanism-based inhibition of gut microbial tryptophanases reduces serum indoxyl sulfate. *Cell. Chem. Biol.* **2023,** *30* (11), 1402-1413.e1407.
4. Murashige, R.; Hayashi, Y.; Hashimoto, M. Asymmetric and efficient synthesis of homophenylalanine derivatives via Friedel–Crafts reaction with trifluoromethanesulfonic acid. *Tetrahedron Lett.* **2008,** *49* (46), 6566-6568.
5. Jackson, R. F. W.; Rettie, A. B.; Wood, A.; Wythes, M. J. Reduction of 4-oxo α-amino acids as a route to 4-hydroxylated α-amino acids. Concise approaches to the synthesis of clavalanine, erythro-4-hydroxyornithine and (+)-bulgecinine. *J. Chem. Soc., Perkin Trans. 1* **1994,** *13,* 1719-1726.
6. Berkeš, D.; Jakubec, P.; Winklerová, D.; Považanec, F.; Daich, A. CIAT with simultaneous epimerization at two stereocenters. Synthesis of substituted β-methyl-α-homophenylalanines. *Org. Biomol. Chem.* **2007,** *5* (1), 121-124.
7. Zimmermann, G.; Hass, W.; Faasch, H.; Schmalle, H.; König, W. A. Synthese reiner Stereoisomerer der N-terminalen Aminosäure von Nikkomycin B. *Liebigs Ann. Chem.* **1985,** *1985* (11), 2165-2177.
8. Cao, H. T.; Roisnel, T.; Valleix, A.; Grée, R. A Tandem Isomerization–Mannich Reaction for the Enantioselective Synthesis of β-Amino Ketones and β-Amino Alcohols with Applications as Key Intermediates for ent-Nikkomycins and ent-Funebrine. *Eur. J. Org. Chem.* **2011,** *2011* (19), 3430-3436.

NMR Spectra

S1 ^{1}H-NMR (D_2O/LiOH, 599 MHz)

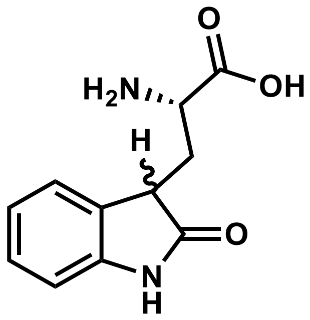

S1 (mixture of diastereomers)

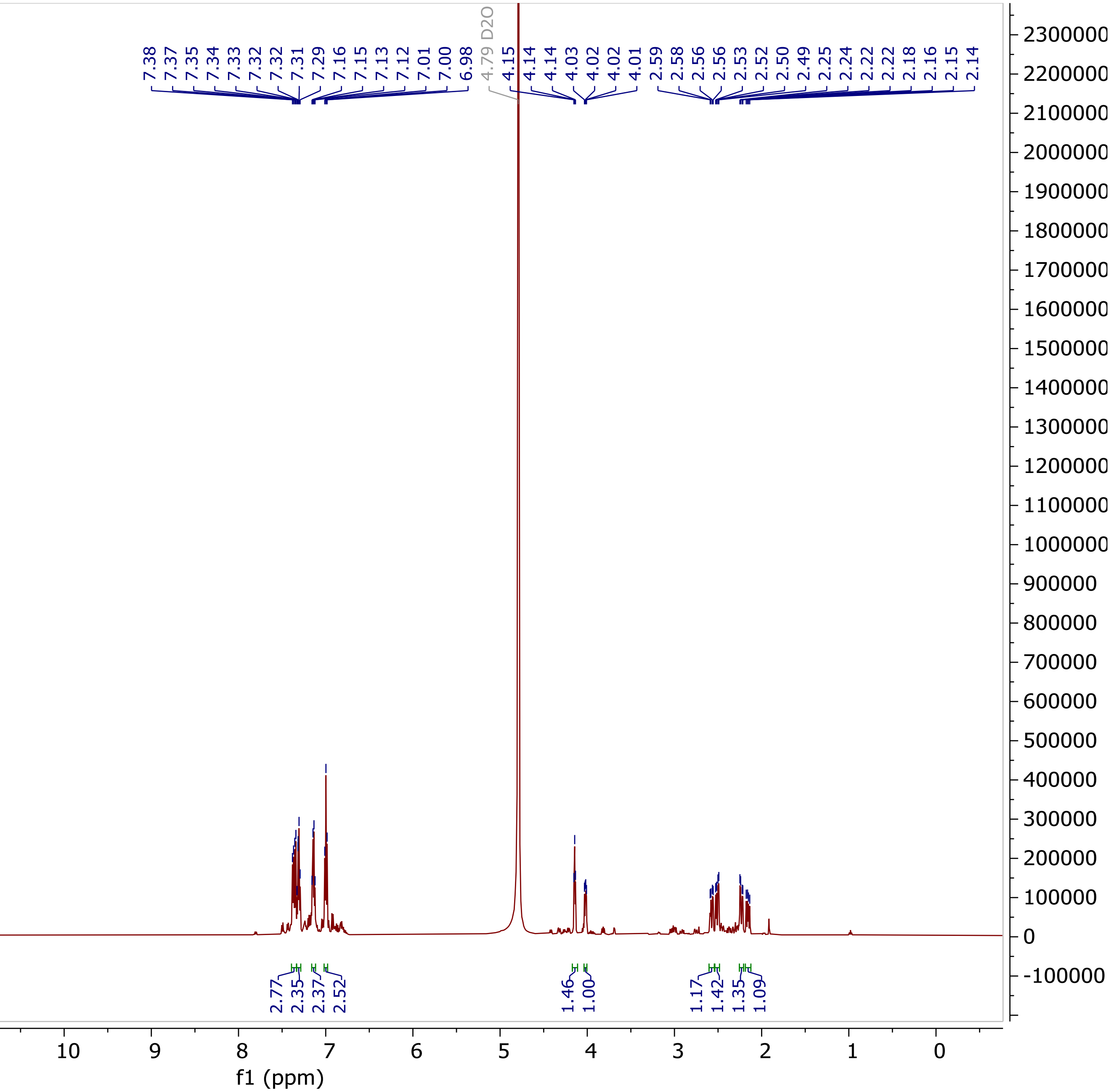

S1 ^{13}C-NMR (D_2O/LiOH, 151 MHz)

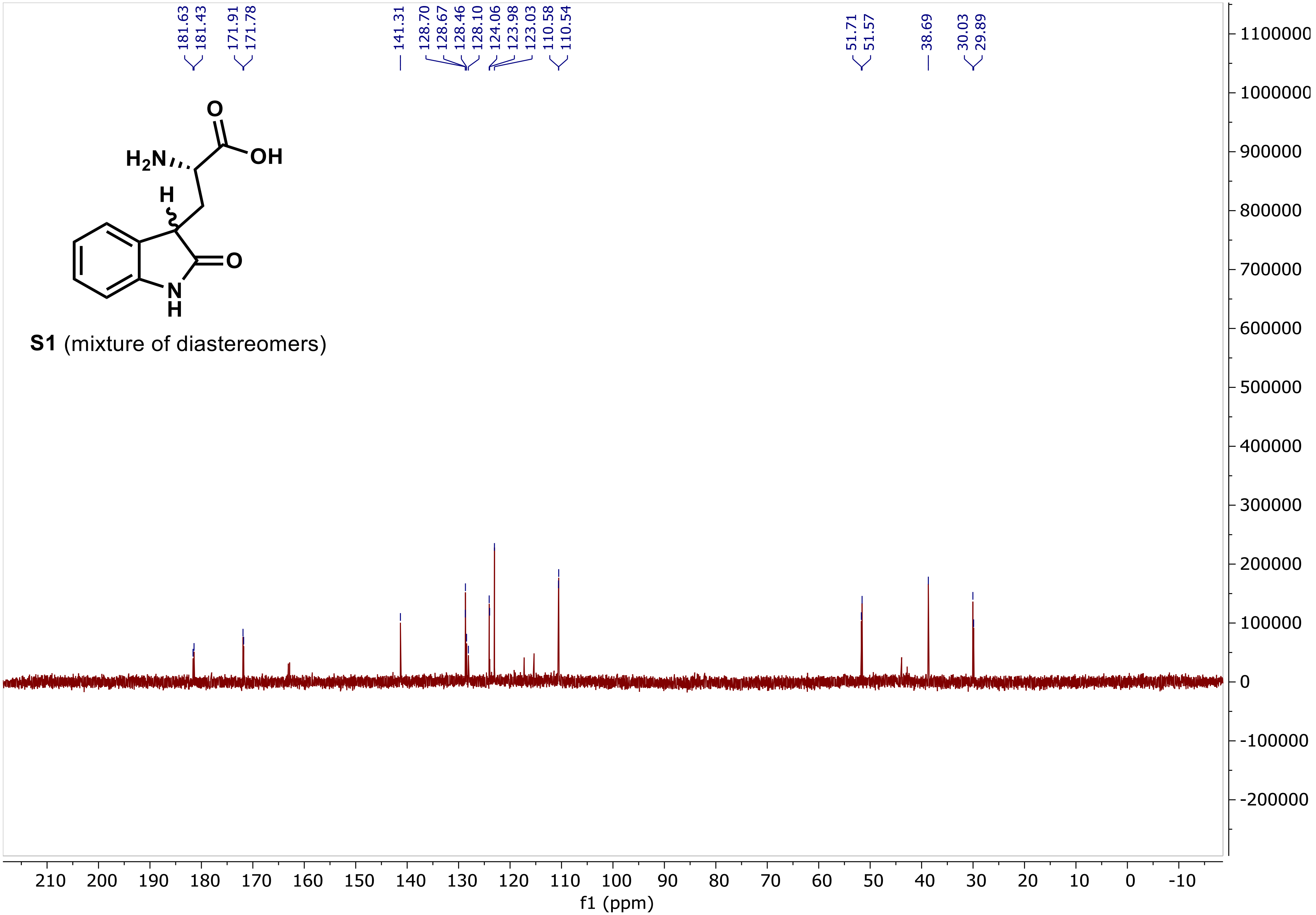

S1 (mixture of diastereomers)

(2*S*,4*R*)-5 ^{1}H-NMR (D_2O/LiOH, 599 MHz)

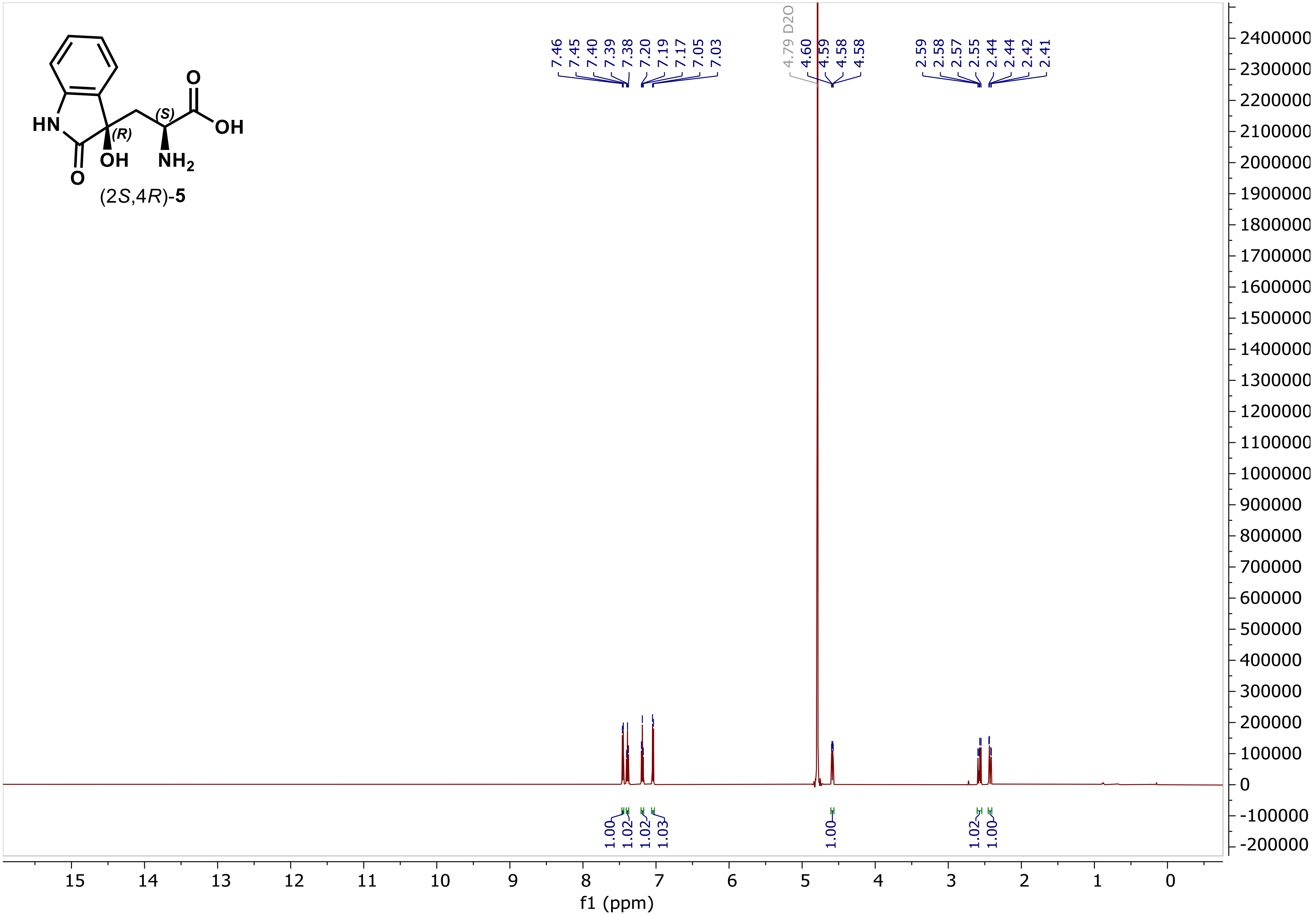

(2*S*,4*S*)-5 ^{1}H-NMR (D_2O/LiOH, 599 MHz)

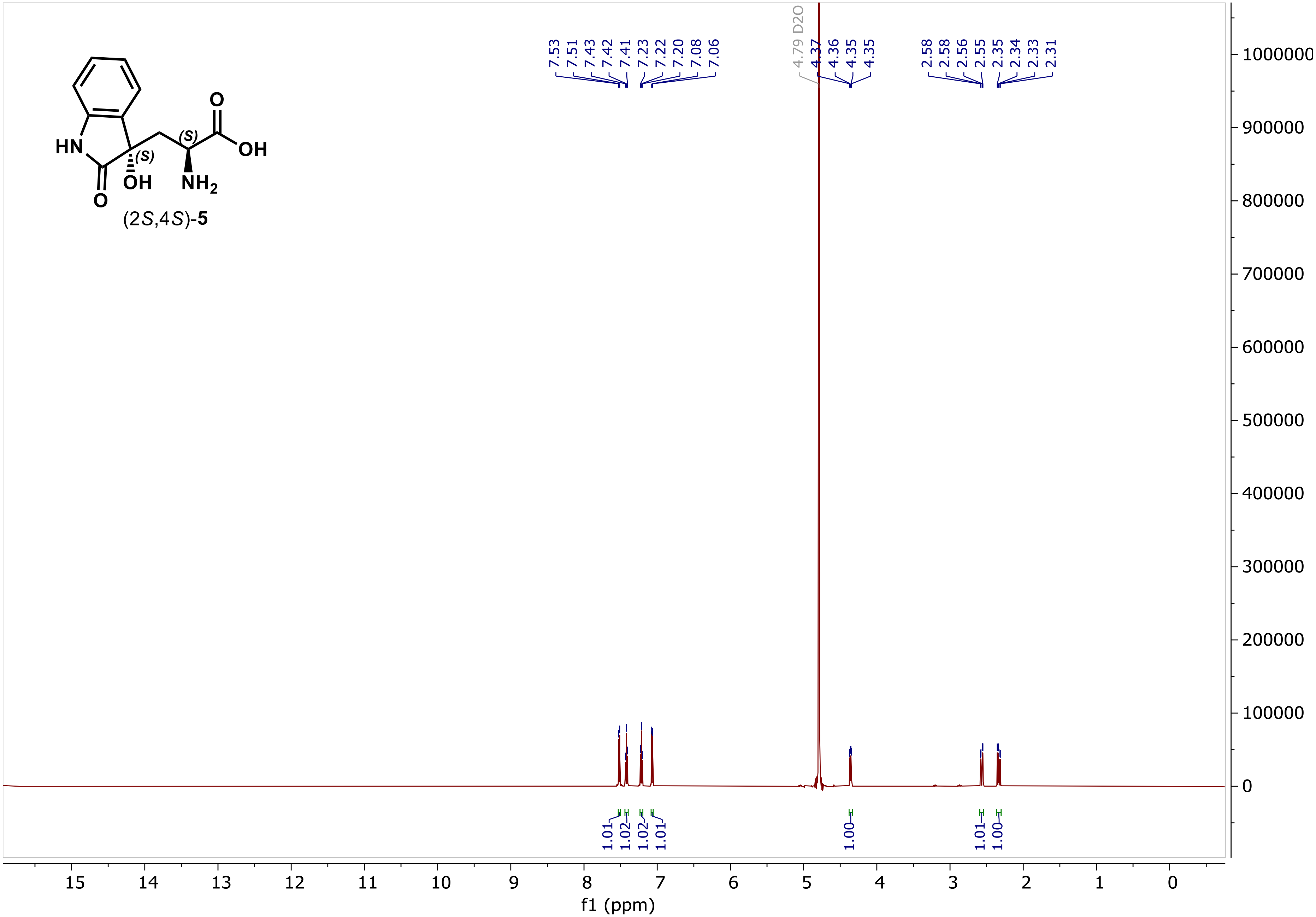

(2*S*,4*R*)-5 ^{13}C-NMR (D_2O/LiOH, 151 MHz)

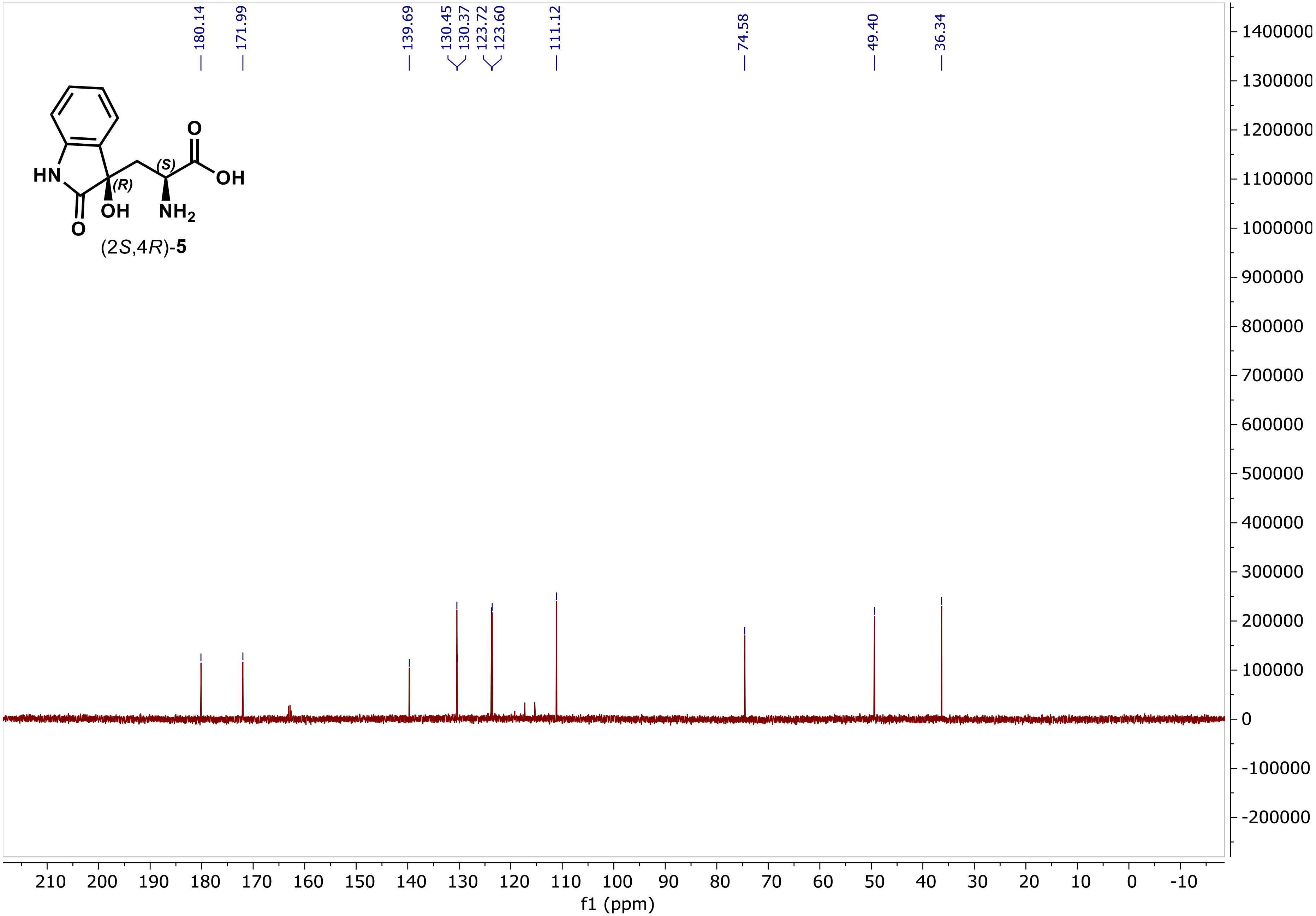

(2*S*,4*S*)-5 ^{13}C-NMR (D_2O/LiOH, 151 MHz)

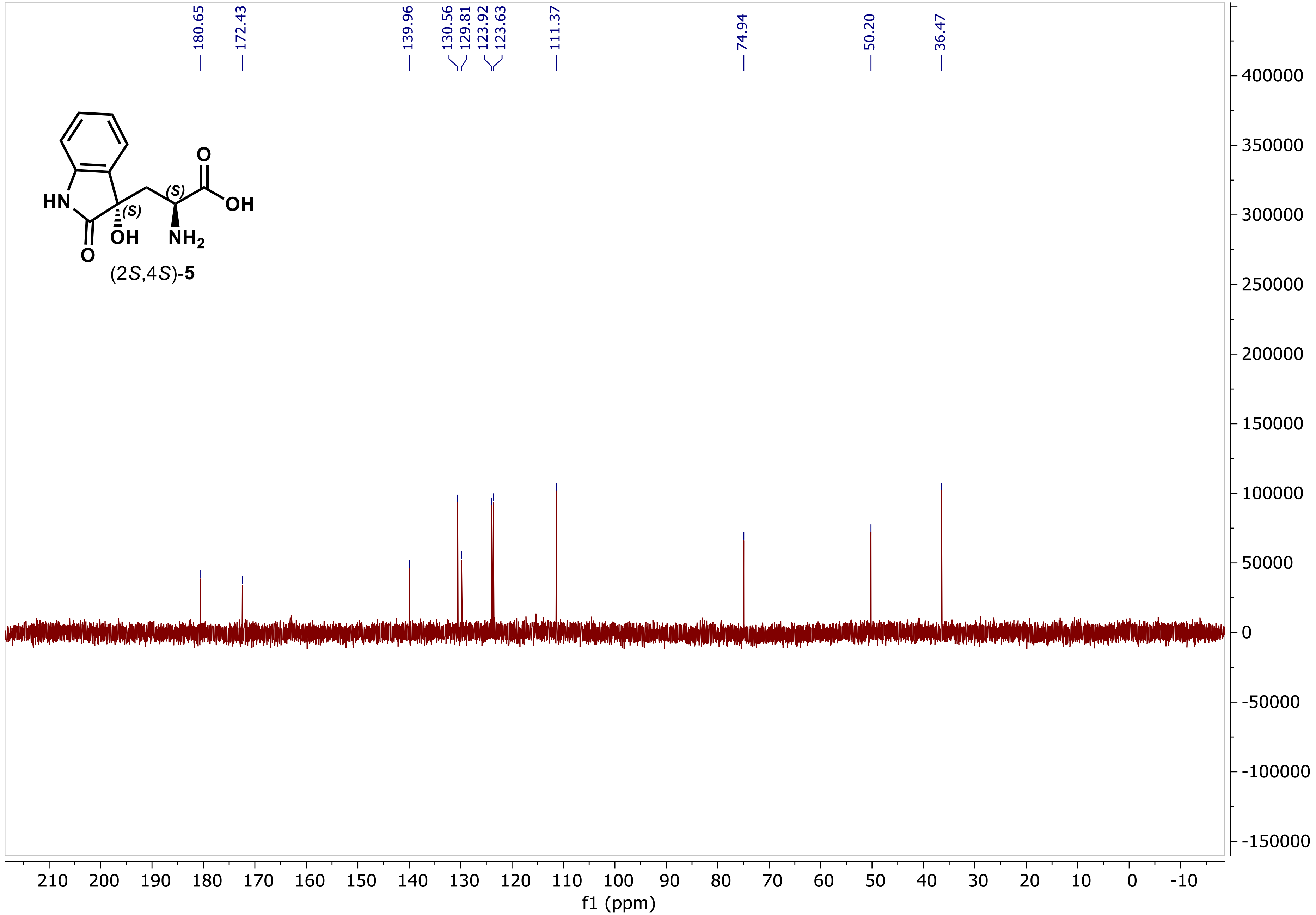

S2 ^{1}H-NMR ($CDCl_3$, 600 MHz)

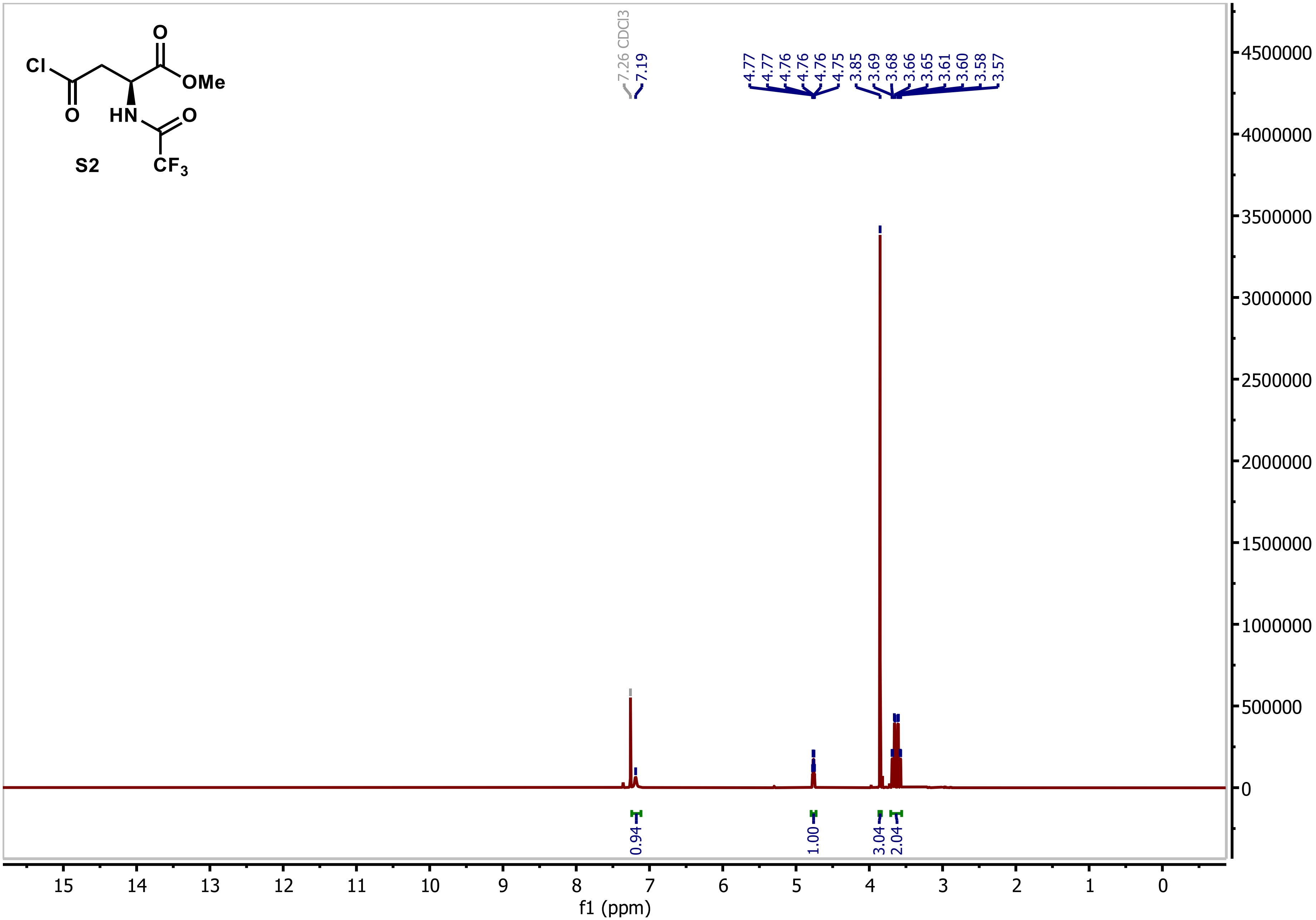

S2 ^{13}C-NMR ($CDCl_3$, 151 MHz)

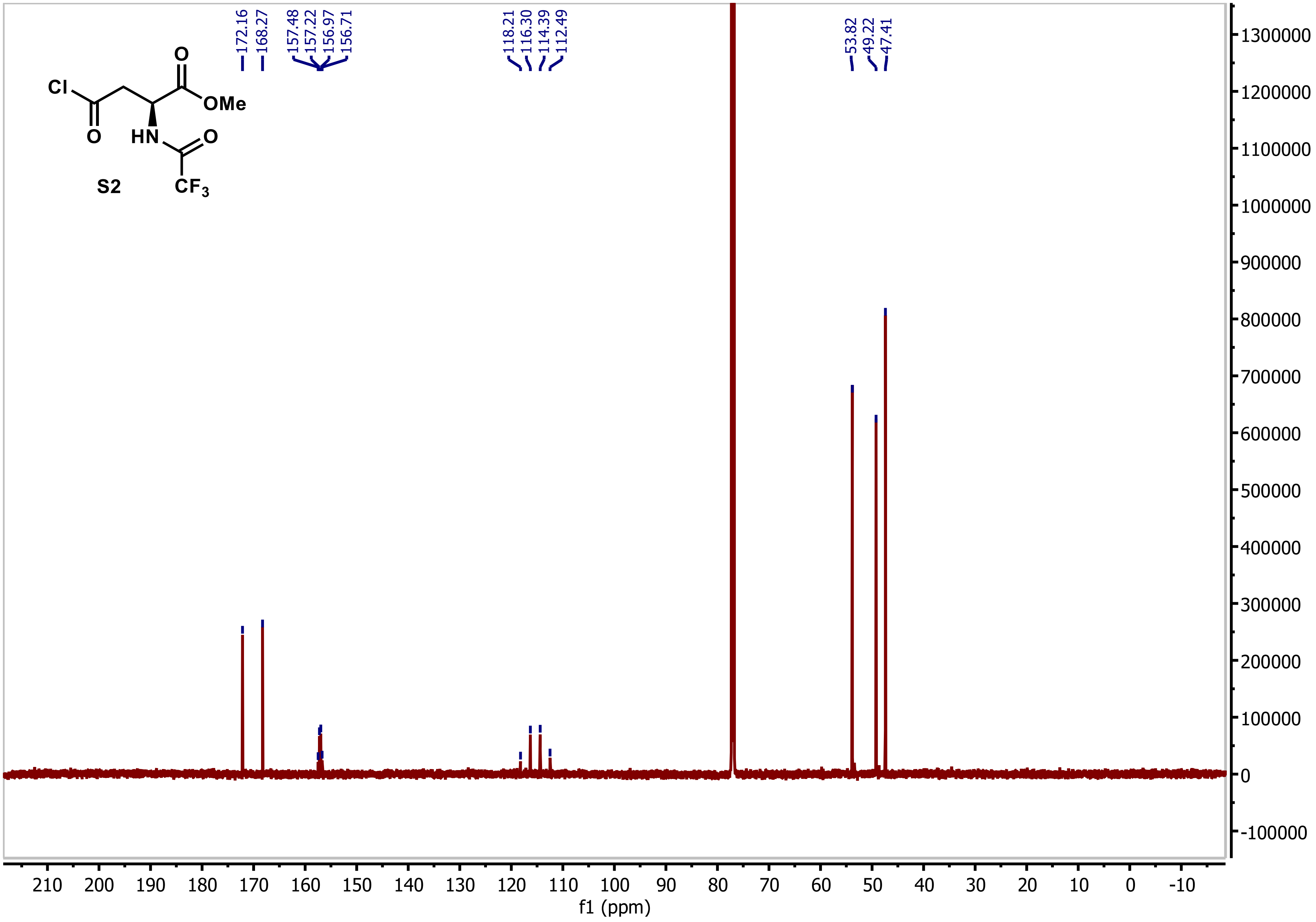

S2 ^{19}F-NMR ($CDCl_3$, 563 MHz)

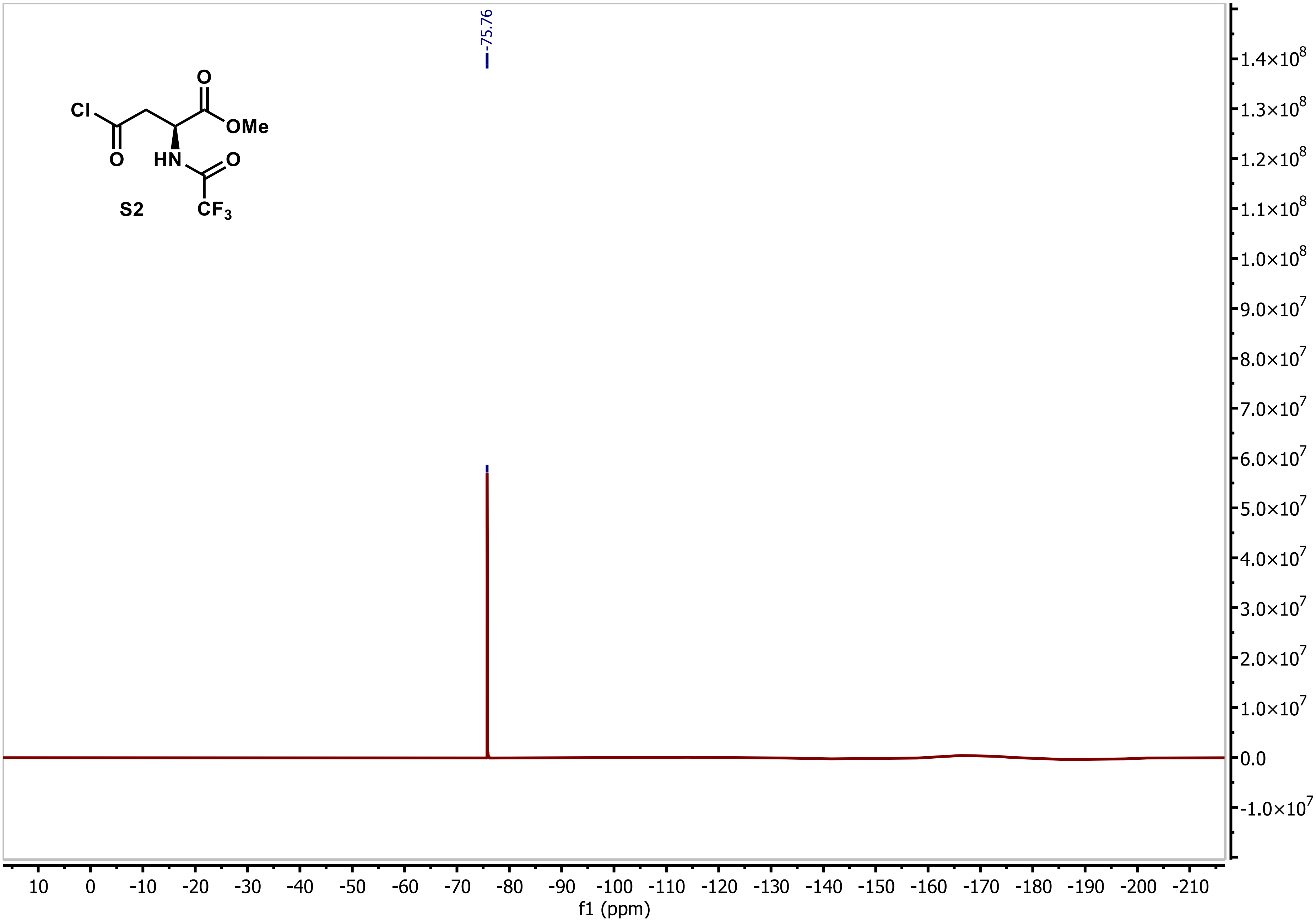

S3 ^{1}H-NMR ($CDCl_3$, 600 MHz)

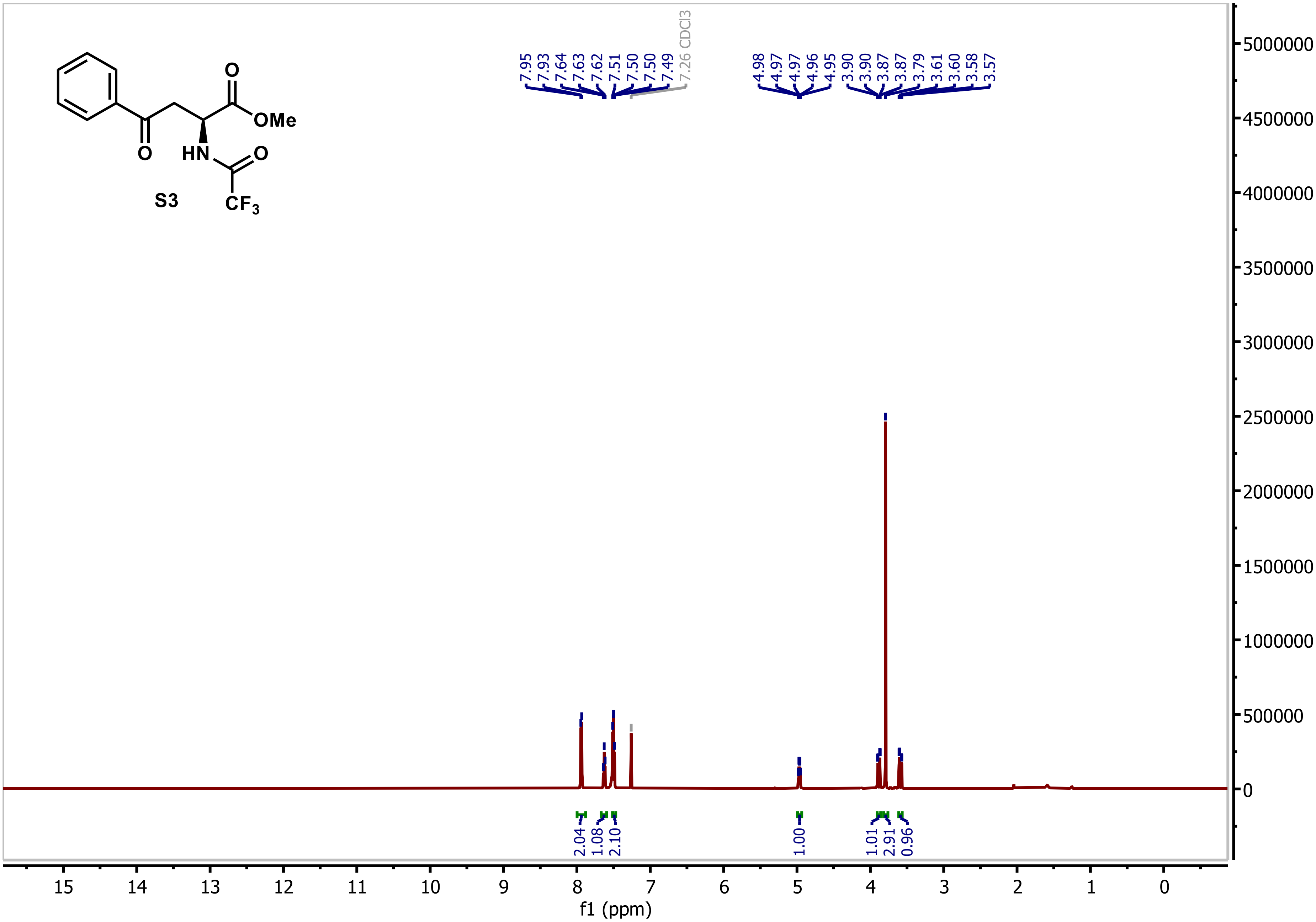

S3 ^{13}C-NMR ($CDCl_3$, 151 MHz)

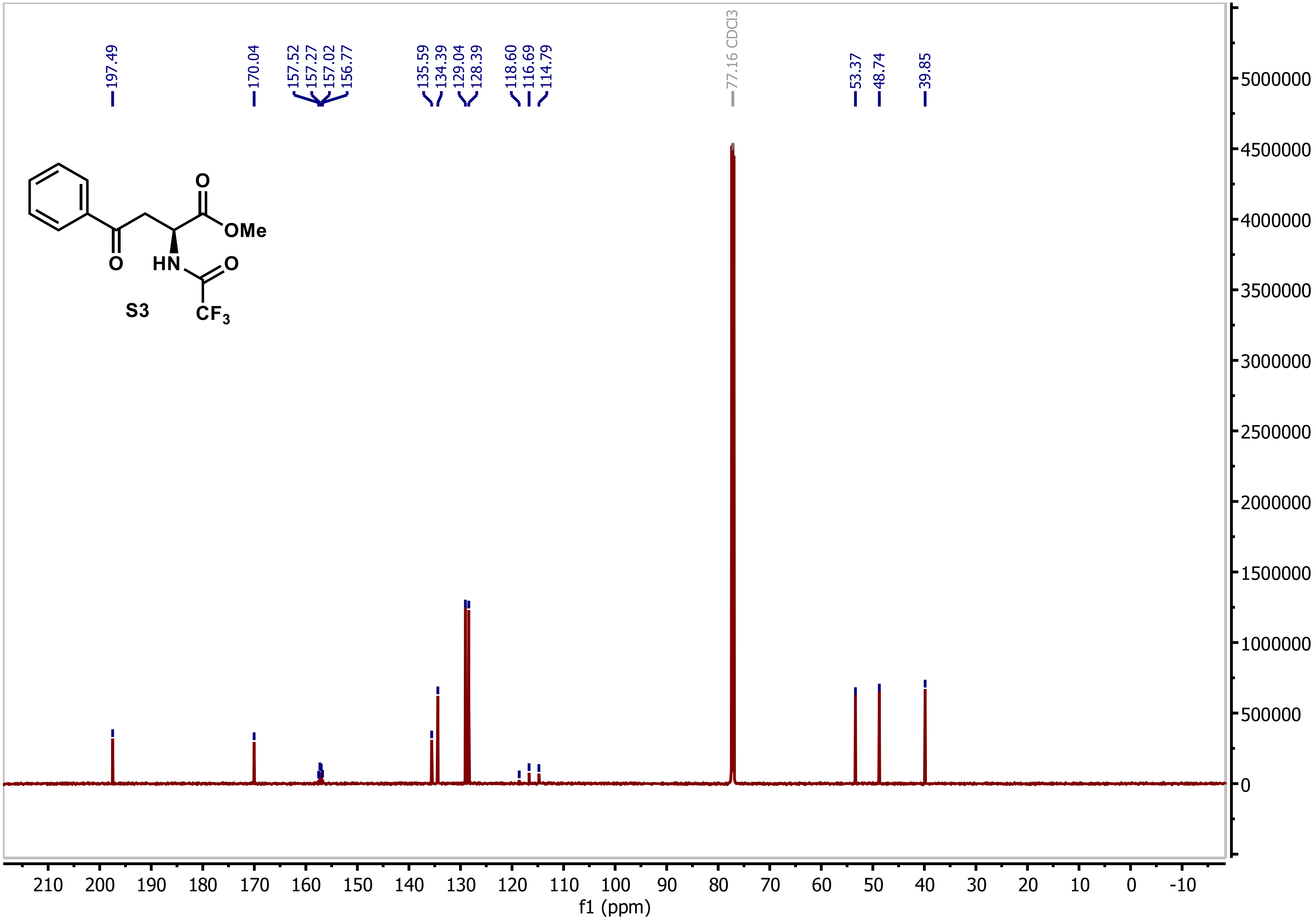

S3 ^{19}F-NMR ($CDCl_3$, 563 MHz)

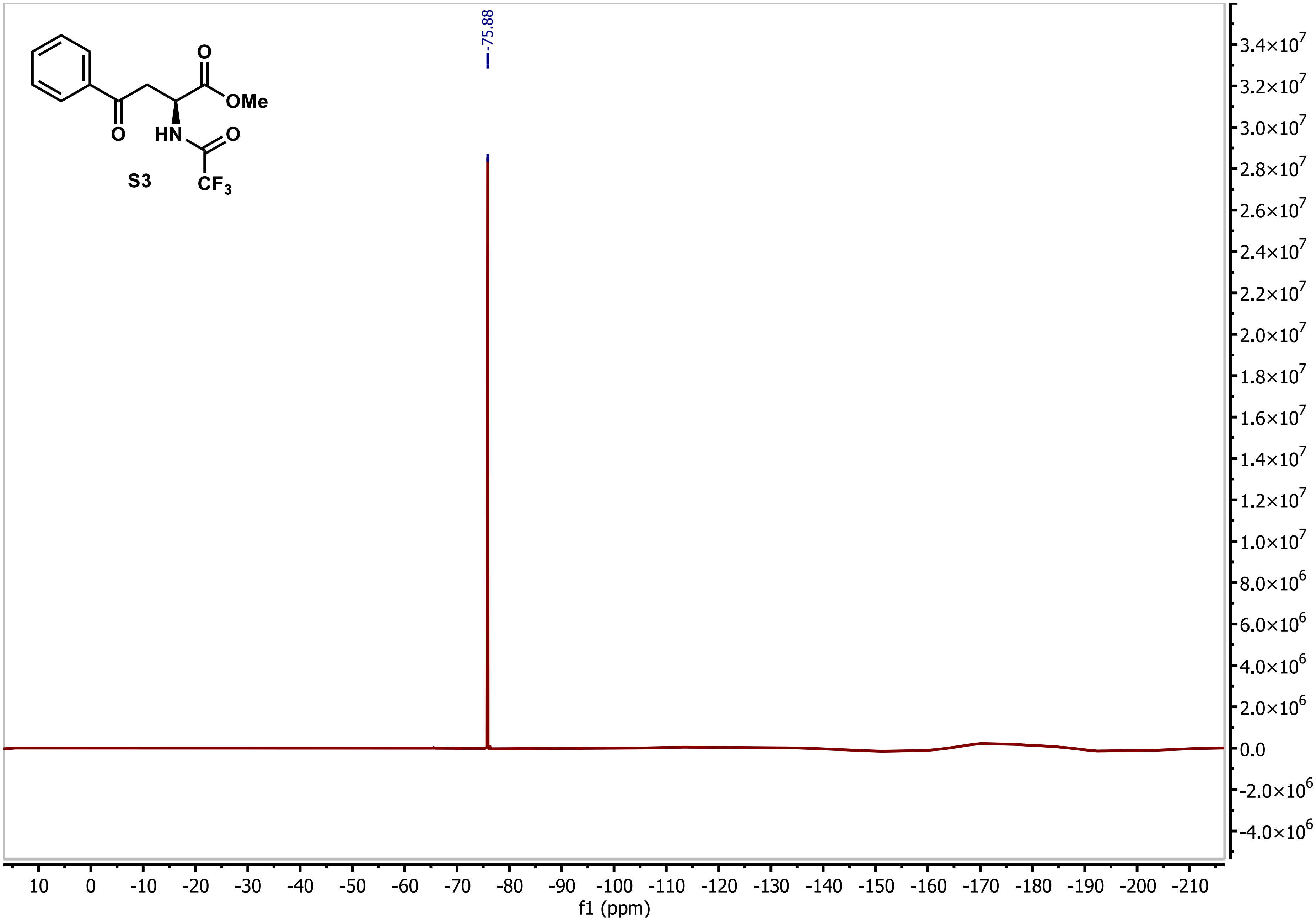

(3*S*,5*R*)-S4 ^{1}H-NMR ($CDCl_3$, 600 MHz)

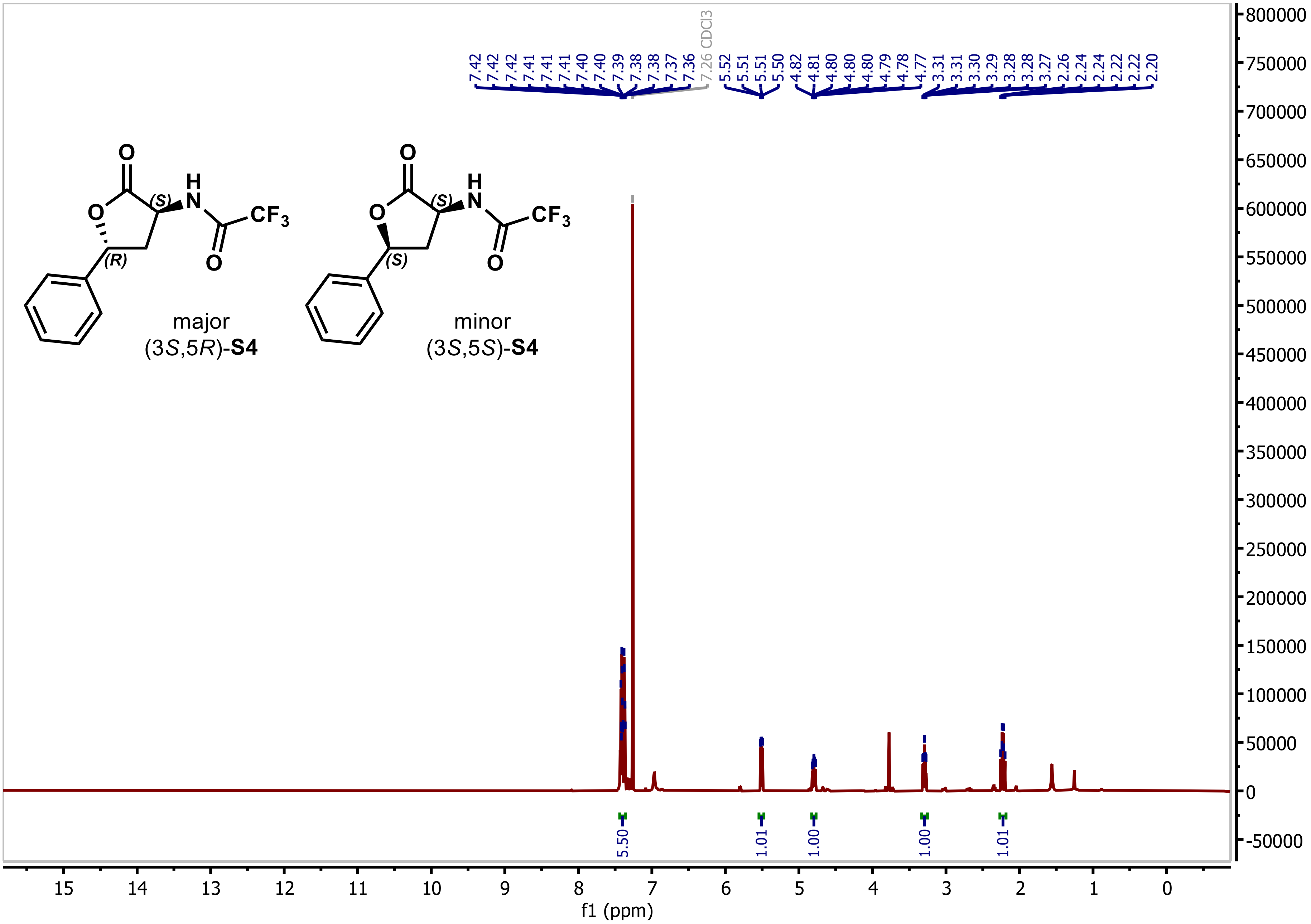

(3*S*,5*S*)-S4 ^{1}H-NMR ($CDCl_3$, 600 MHz)

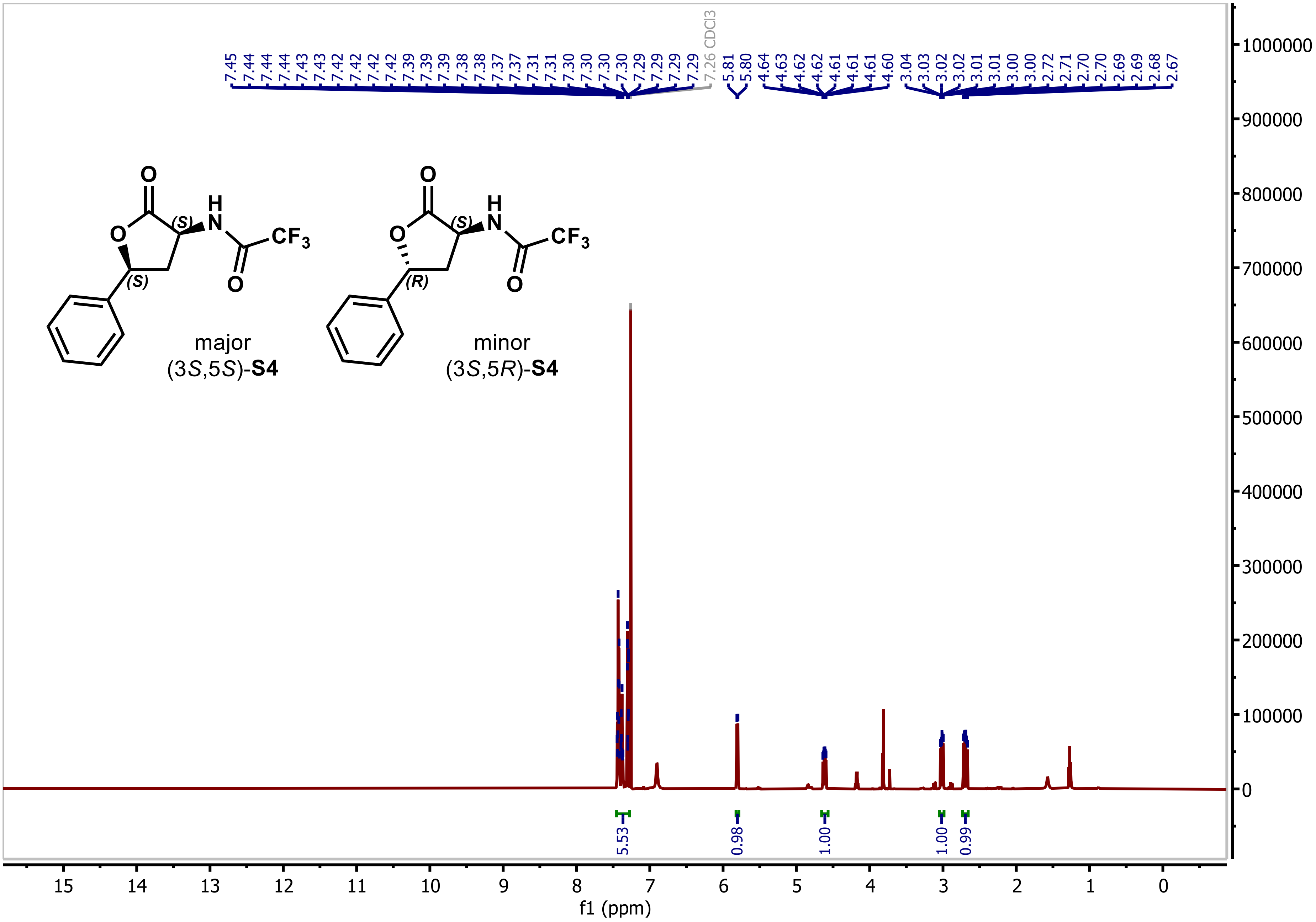

(3*S*,5*R*)-S4 ^{13}C-NMR ($CDCl_3$, 151 MHz)

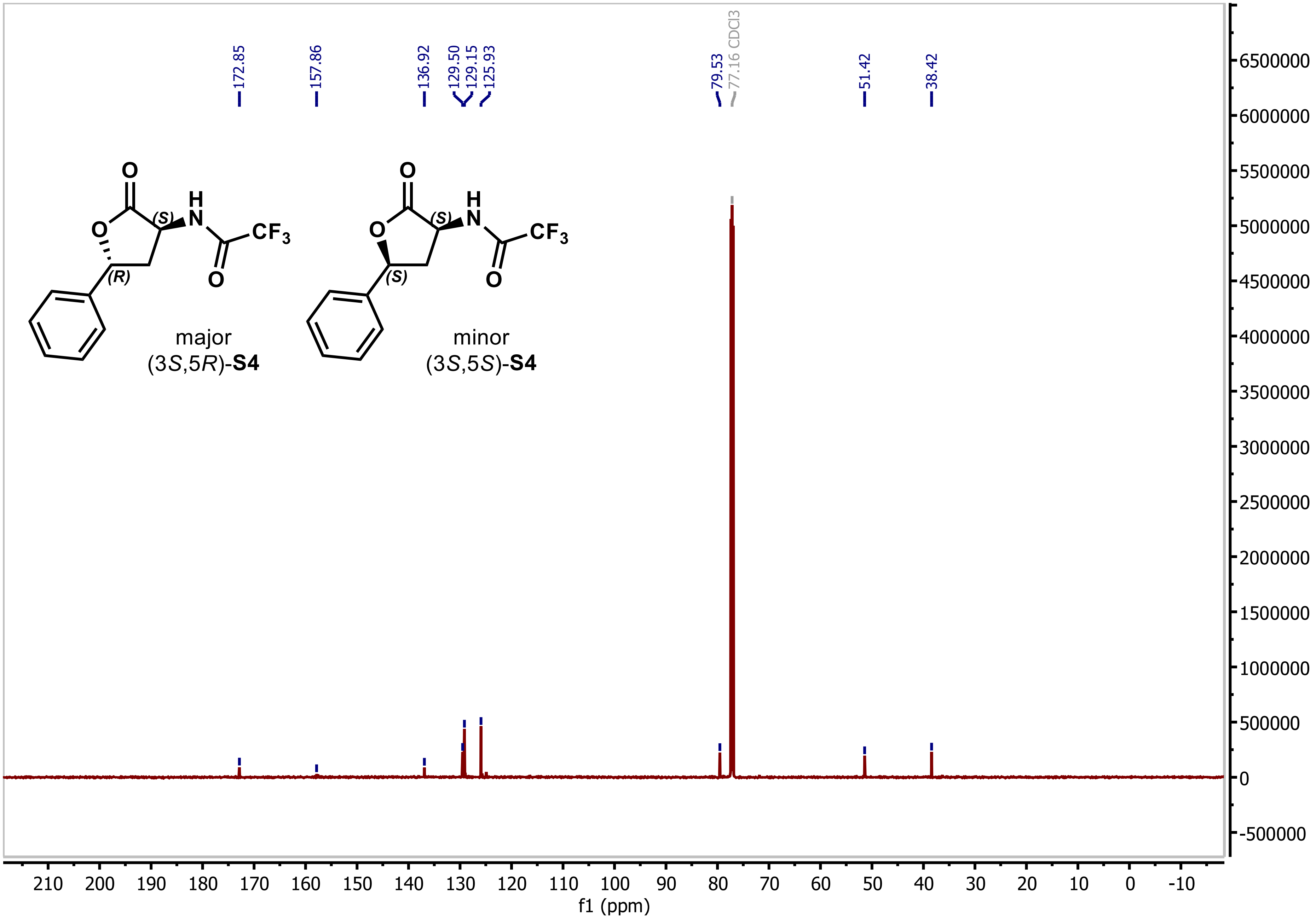

(3*S*,5*S*)-S4 ^{13}C-NMR ($CDCl_3$, 151 MHz)

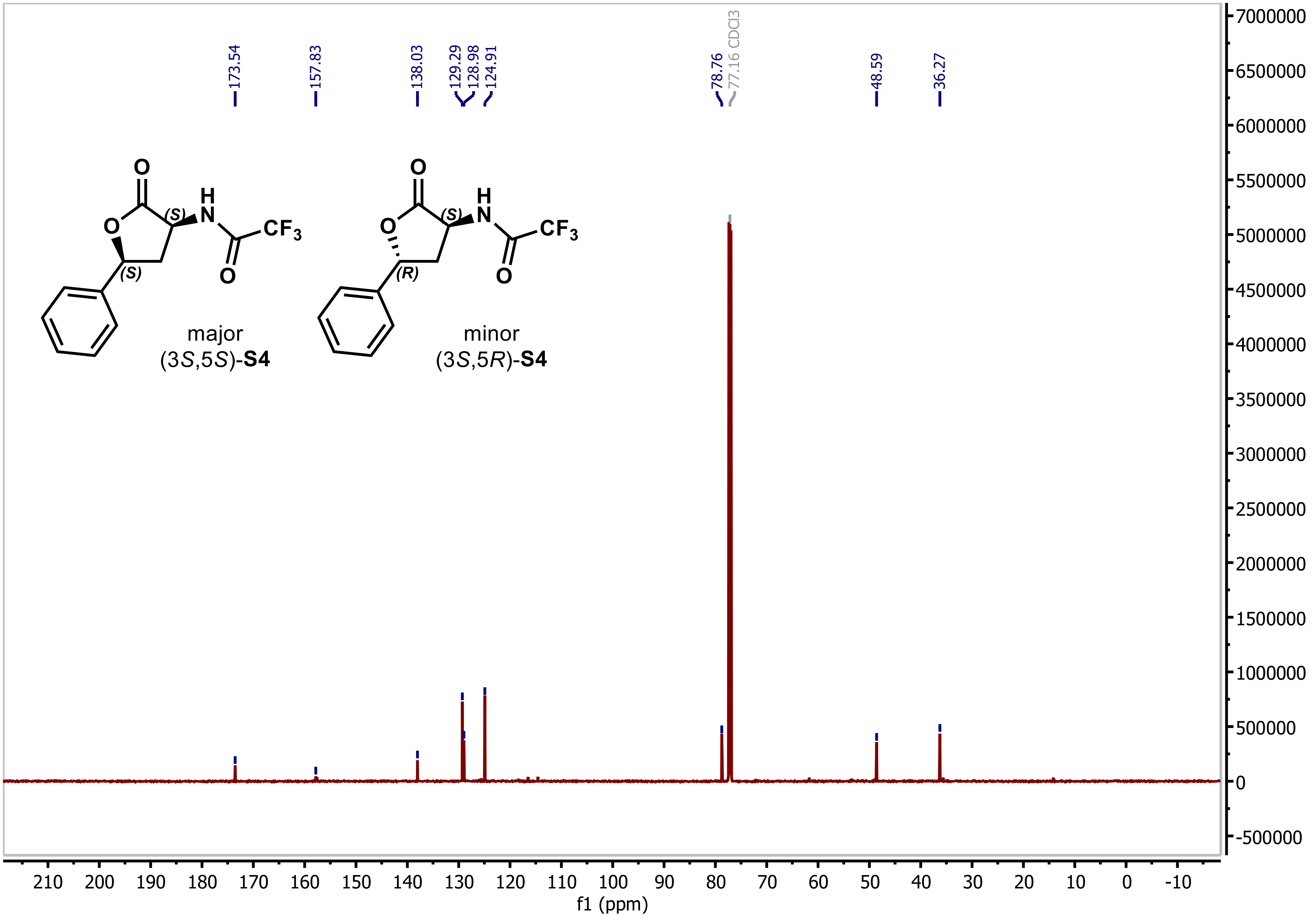

(2*S*,4*S*)-6 ^{1}H-NMR (D_2O/LiOH, 600 MHz)

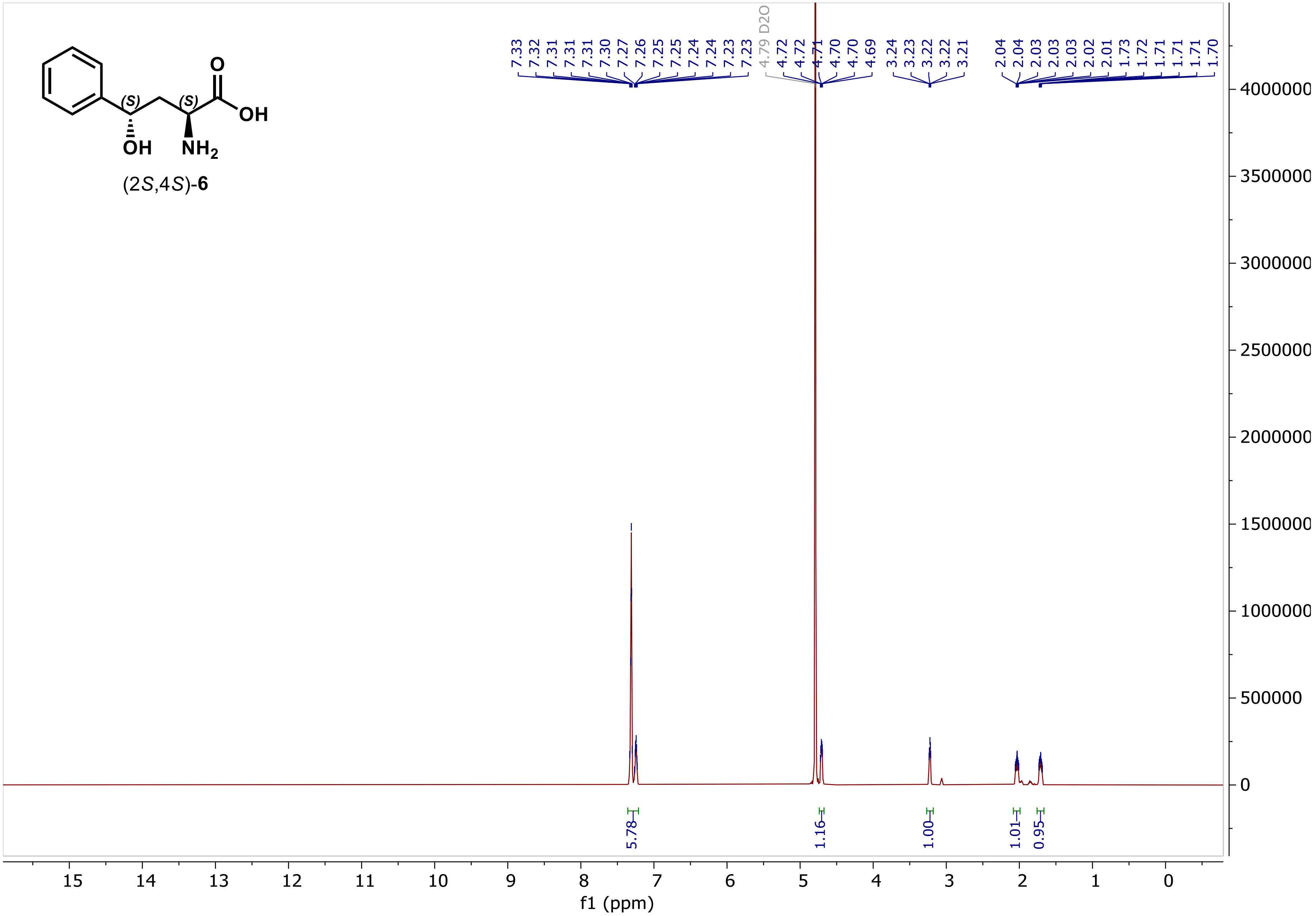

(2*S*,4*S*)-6 ^{13}C-NMR (D_2O/LiOH, 151 MHz)

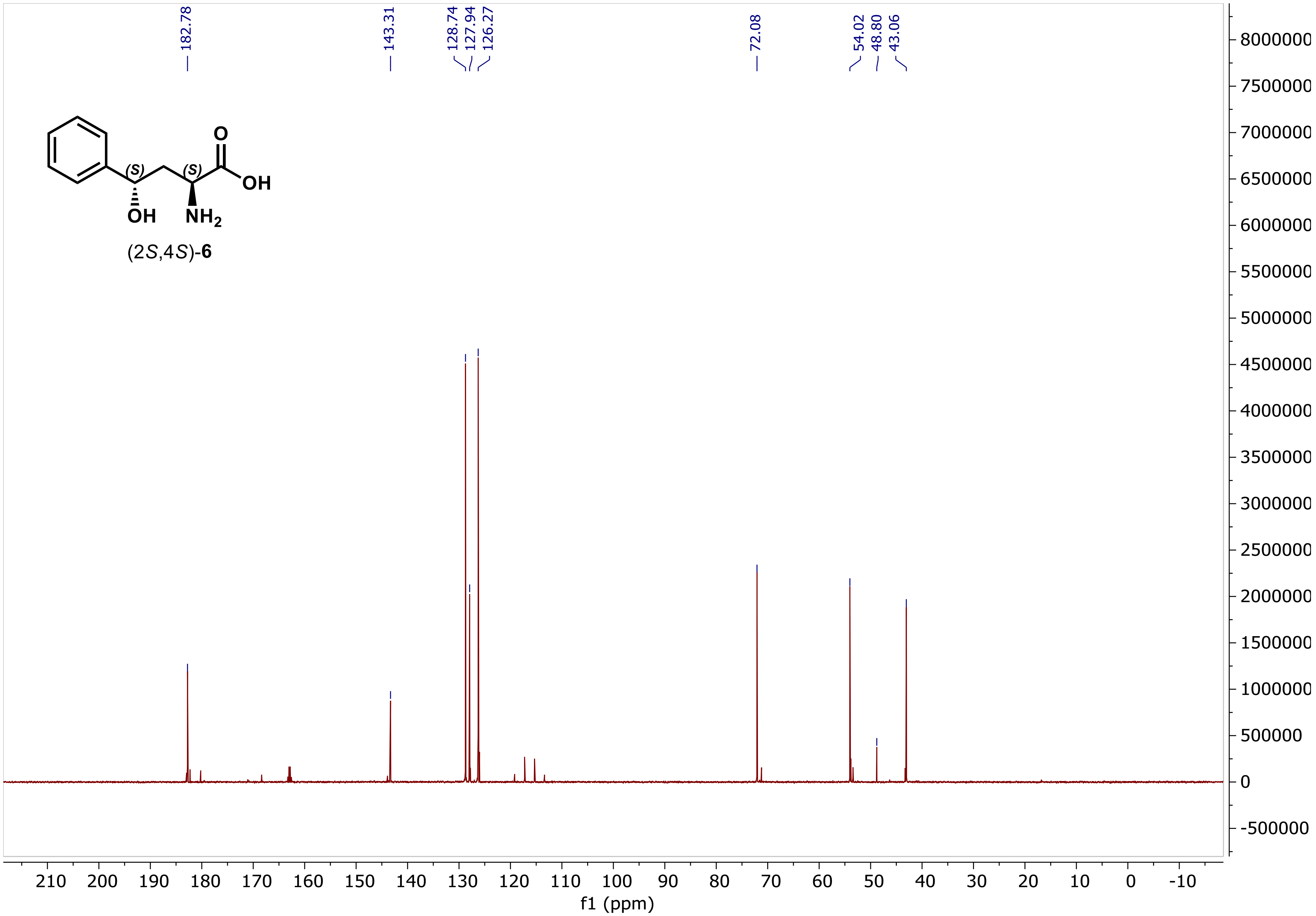

(2*S*,4*R*)-6 ^{1}H-NMR (D_2O/LiOH, 600 MHz)

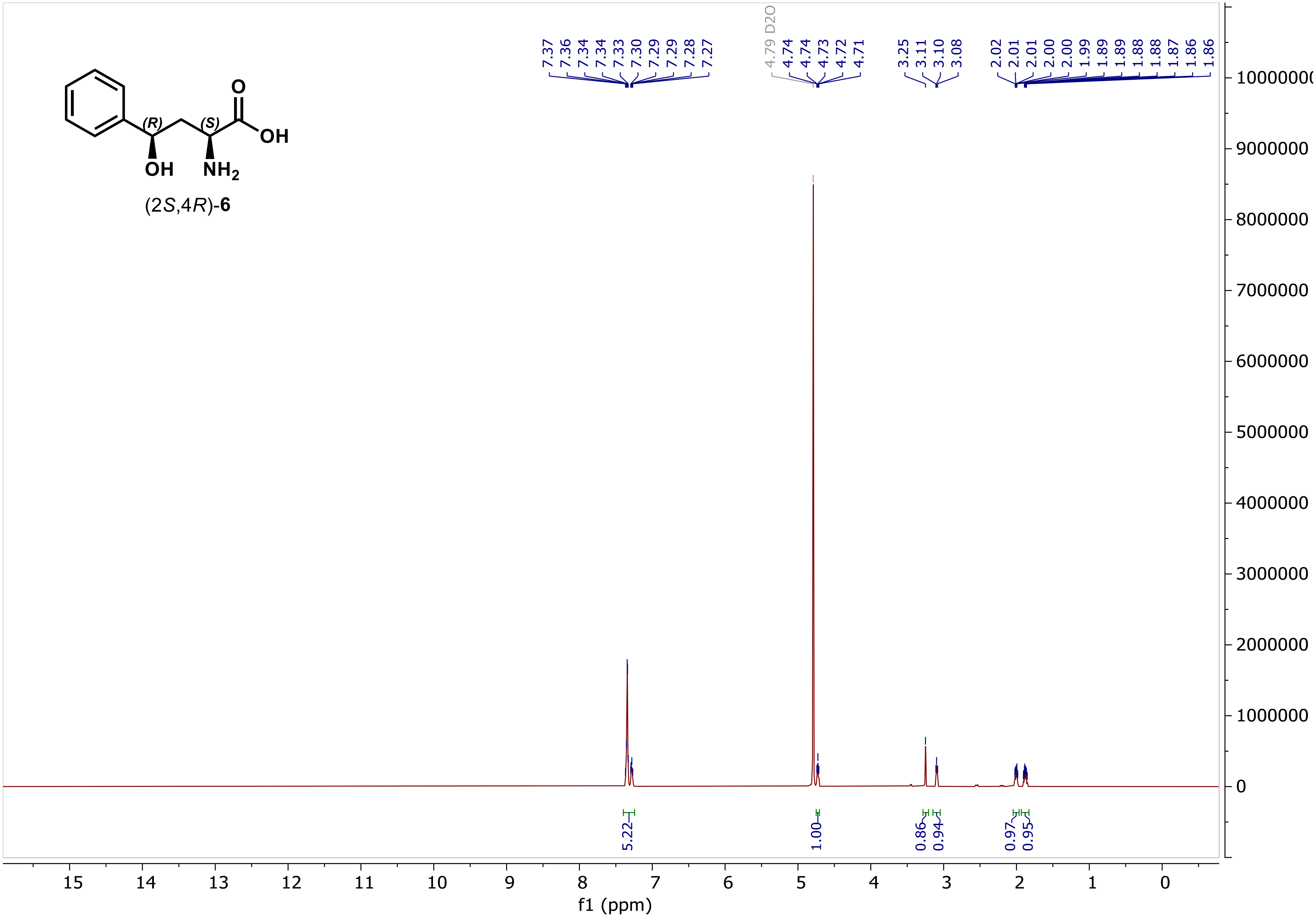

(2*S*,4*R*)-6 ^{13}C-NMR (D_2O/LiOH, 151 MHz)

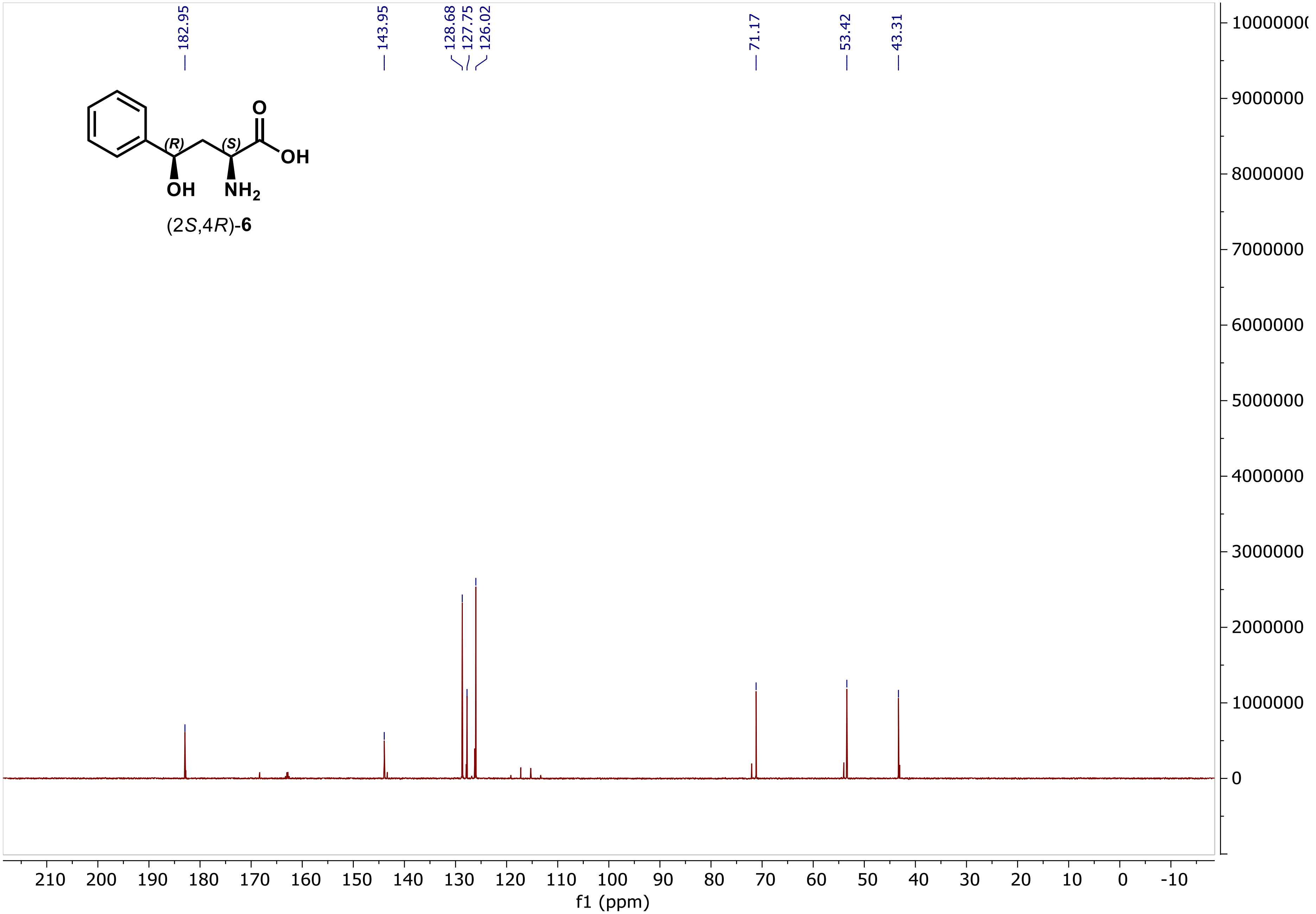

(2*S*,4*R*)-**6**

S5 ^{1}H-NMR ($CDCl_3$, 600 MHz)

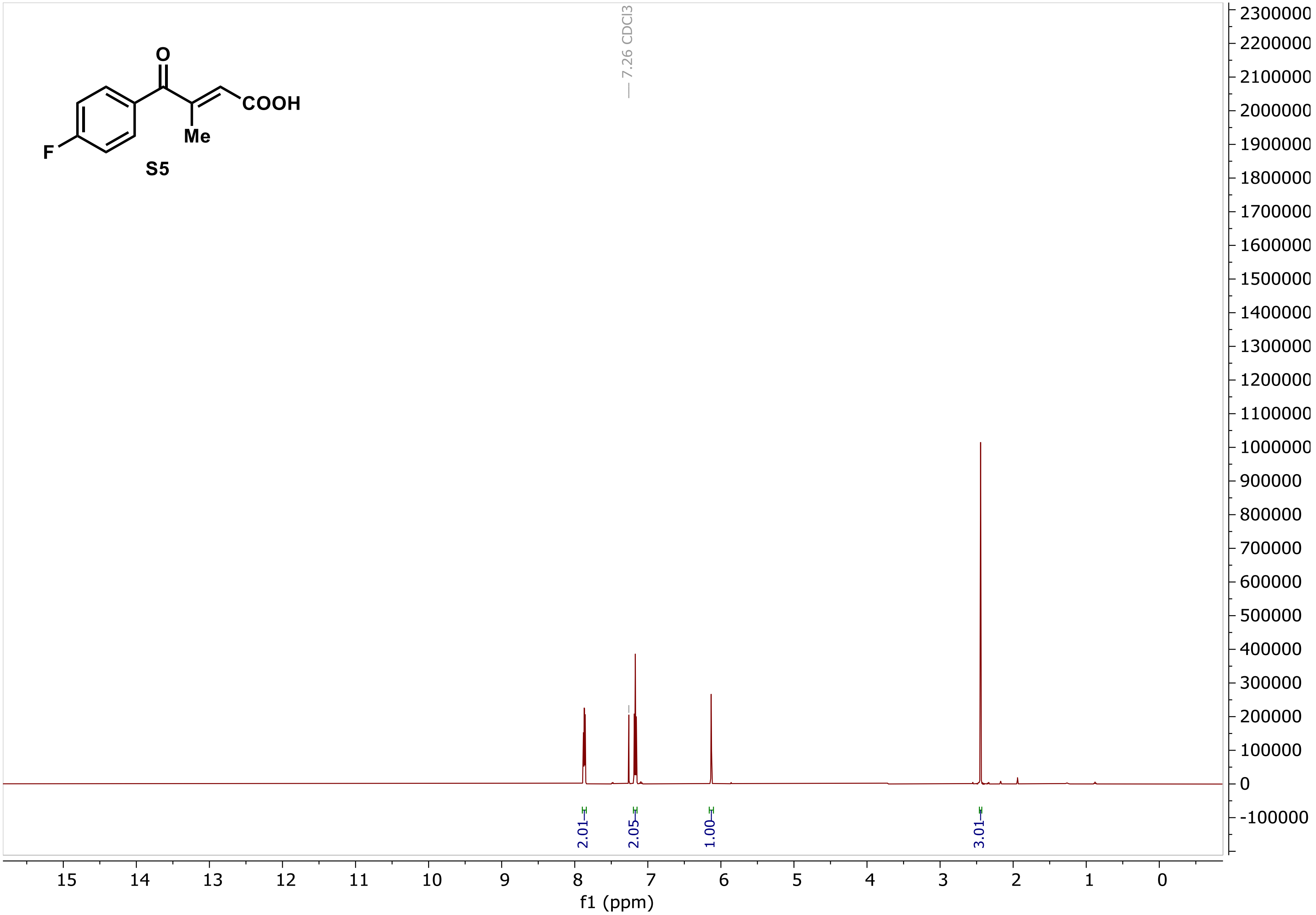

S5 ^{13}C-NMR ($CDCl_3$, 151 MHz)

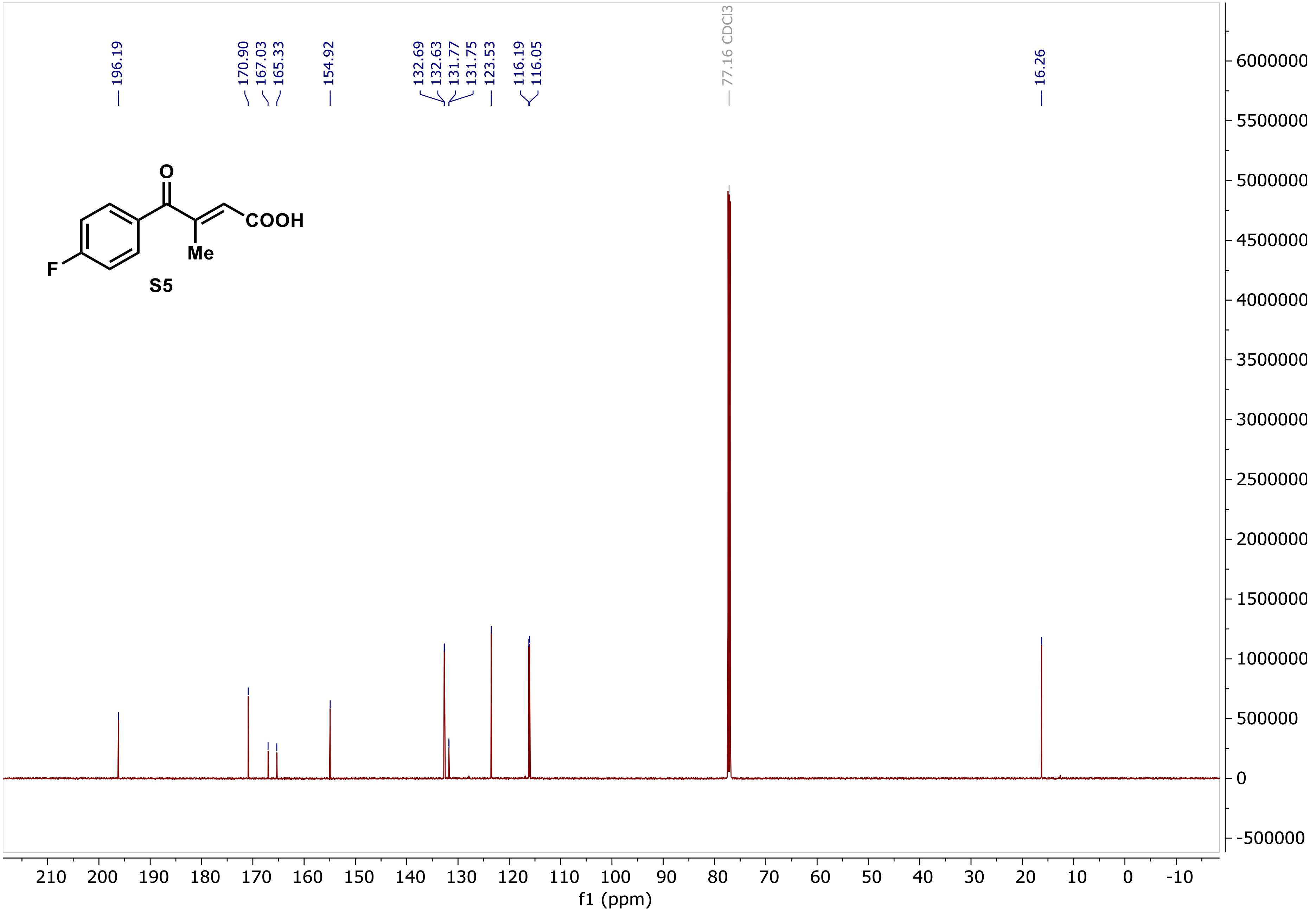

S6 ^{1}H-NMR $(CD_3)_2CO/DCl$, 600 MHz)

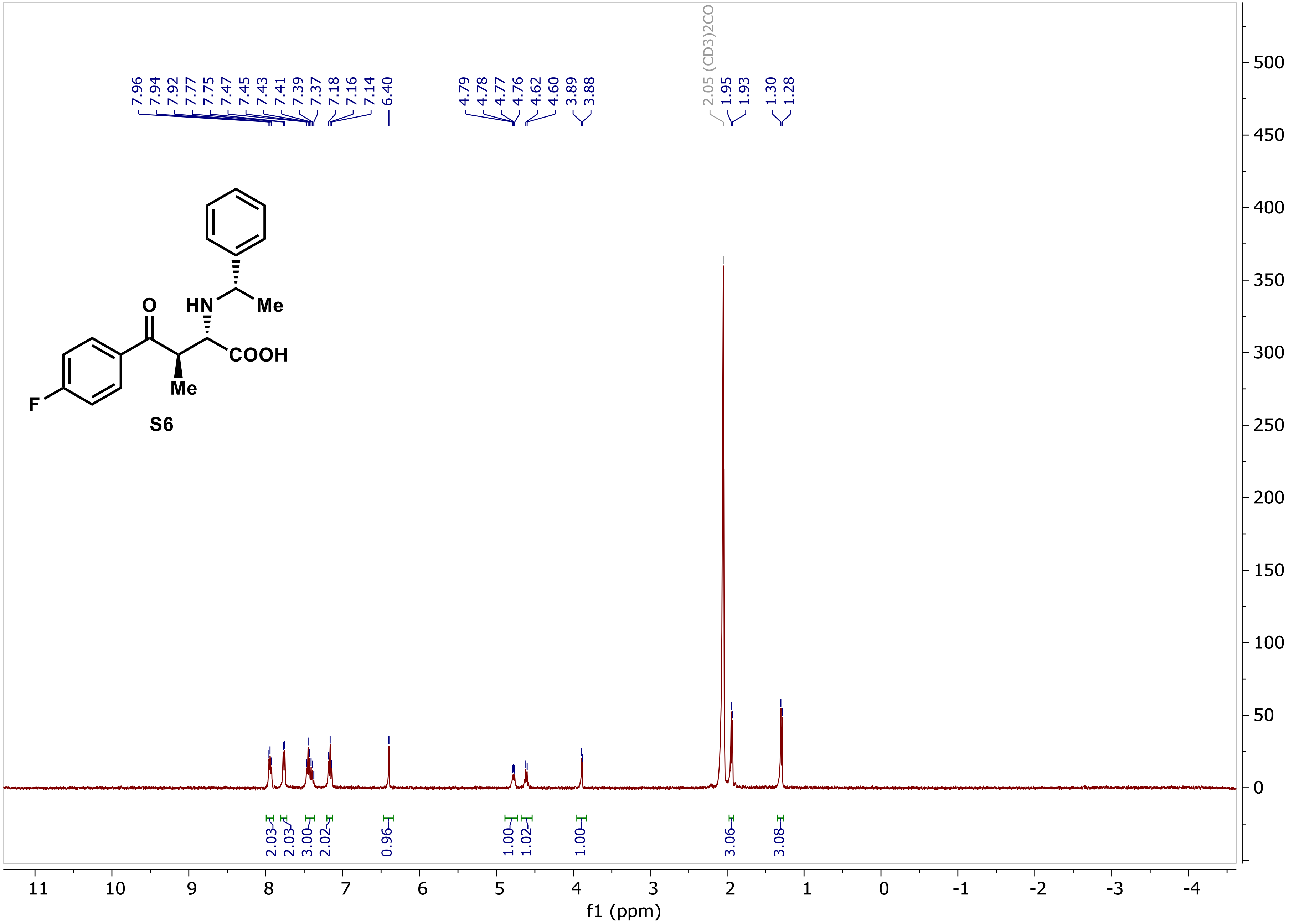

S6 ^{13}C-NMR $(CD_3)_2CO$/DCl, 151 MHz)

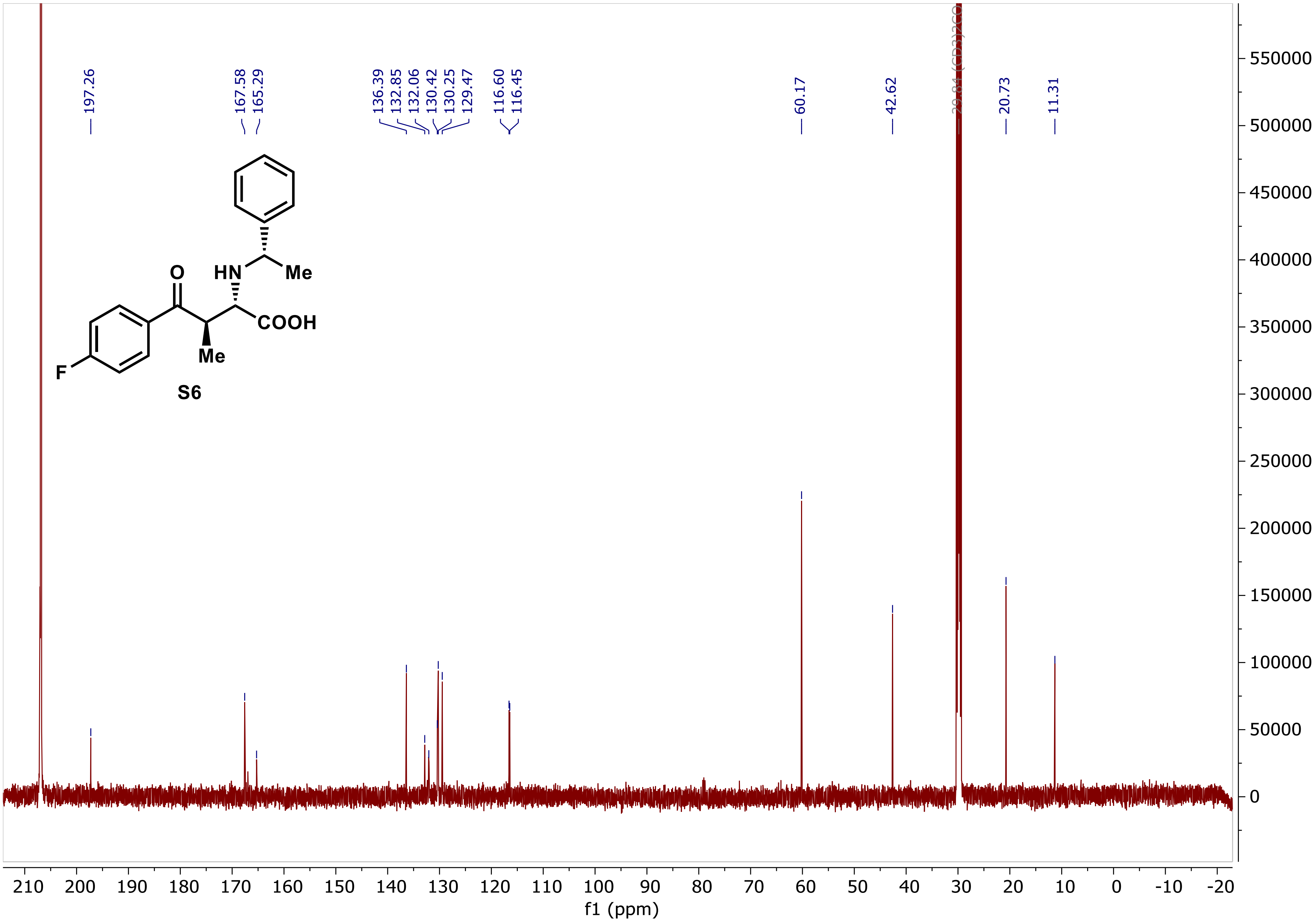

(2*S*,3*R*,4*S*)-S7 ^{1}H-NMR (D_2O/NaOH, 600 MHz)

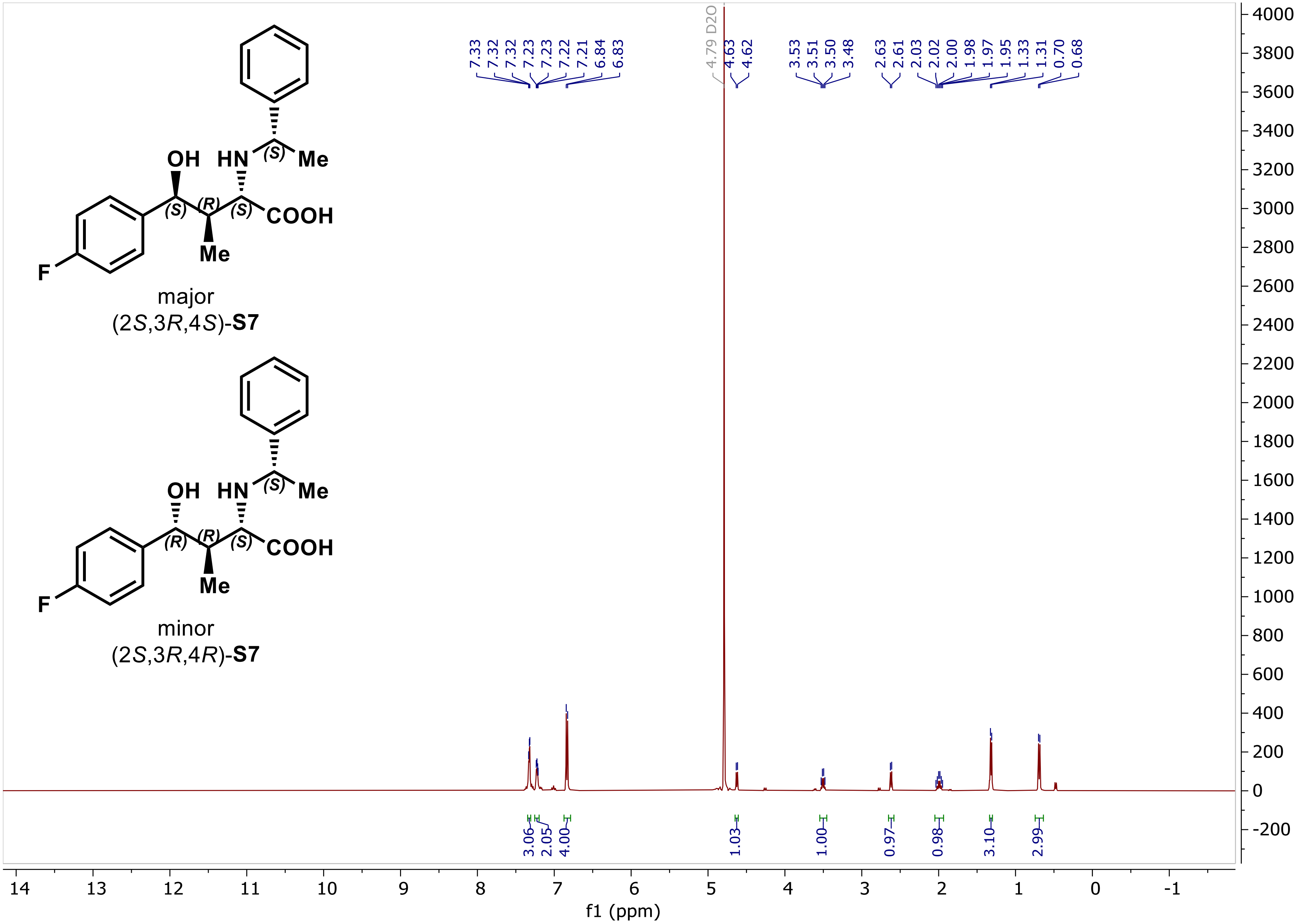

(2*S*,3*R*,4*S*)-S7 ^{13}C-NMR (D_2O/NaOH, 151 MHz)

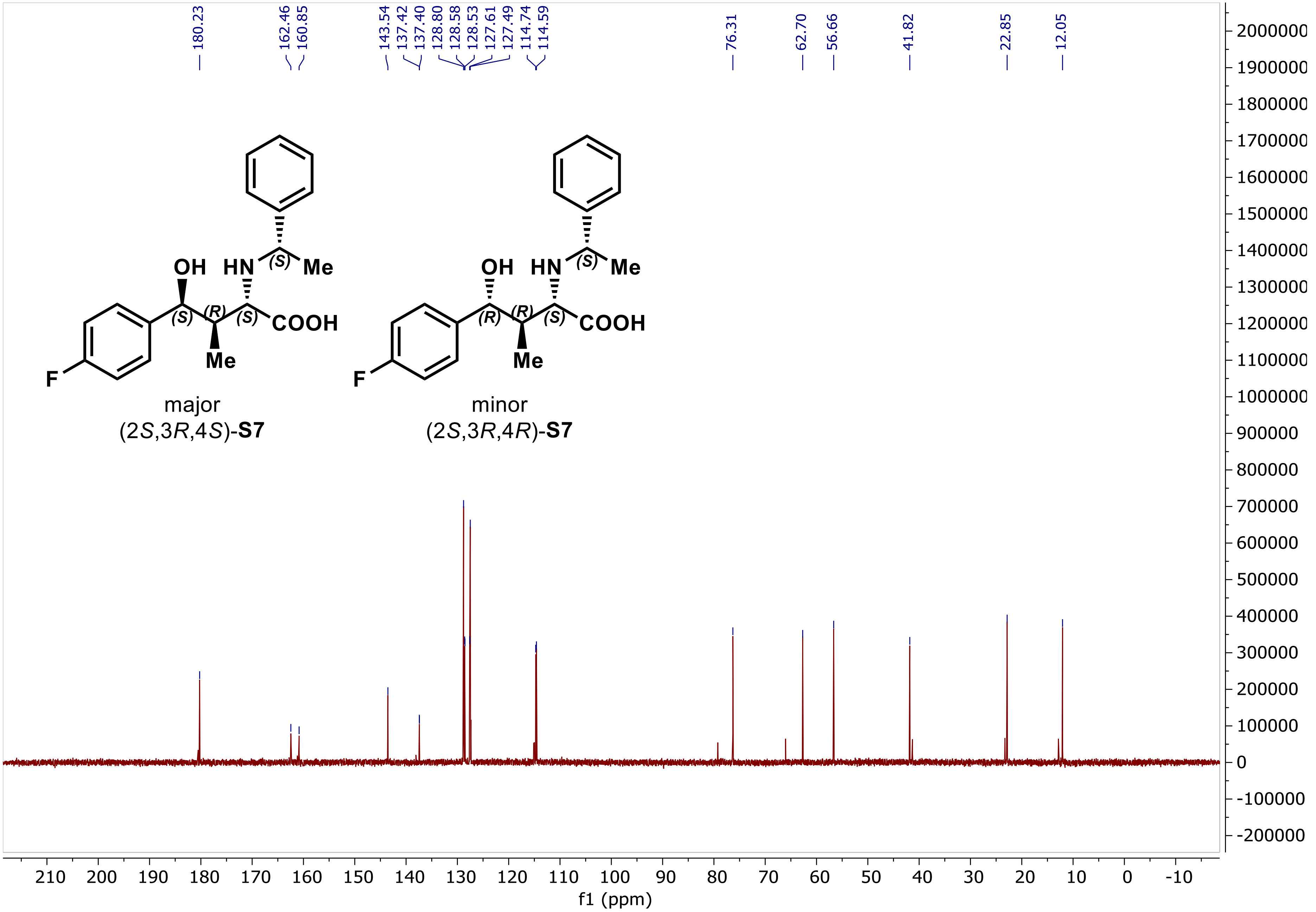

(2*S*,3*R*,4*S*)-S7 ^{19}F-NMR (D_2O/NaOH, 563 MHz)

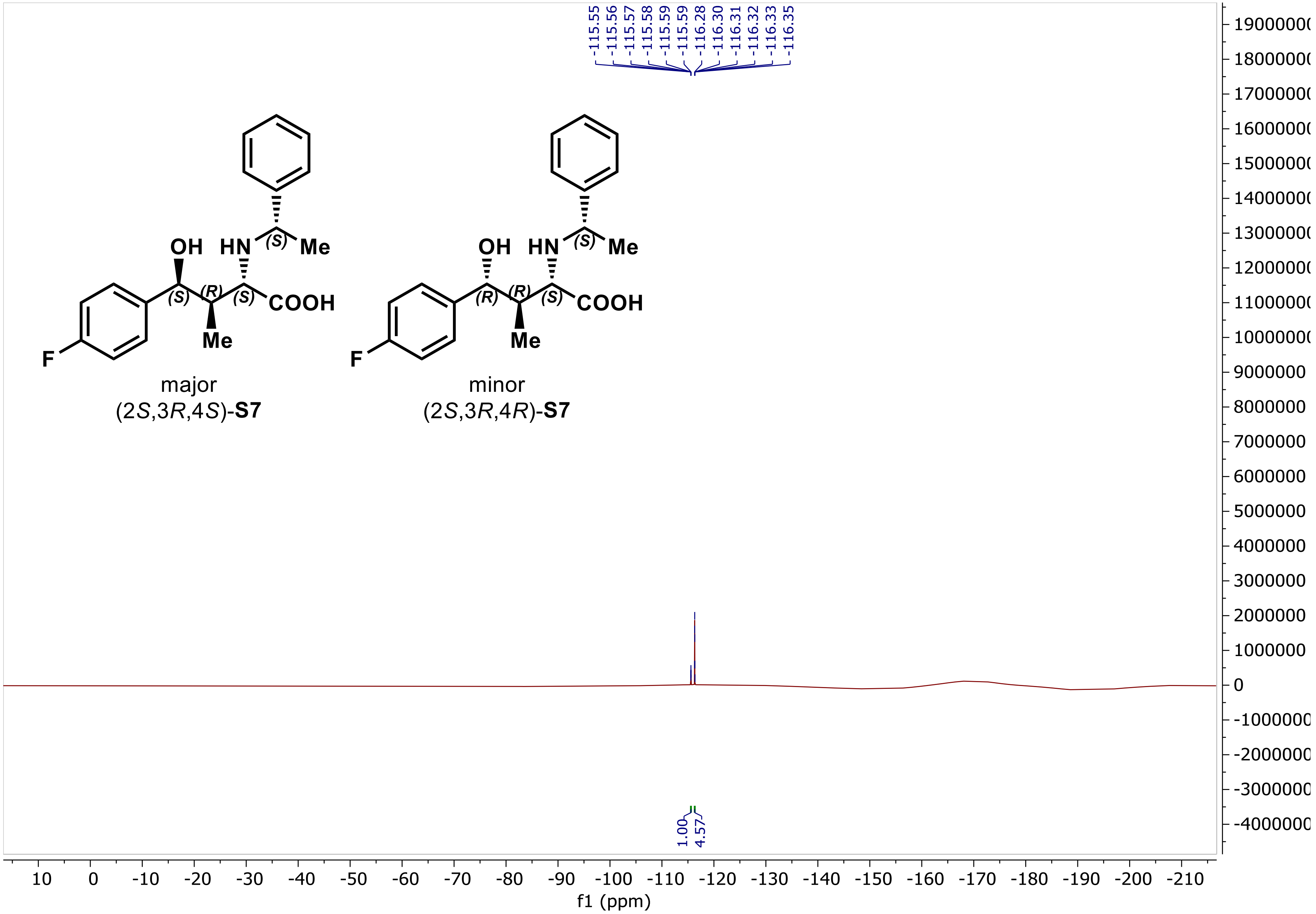

(2*S*,3*R*,4*R*)-S7 ^{1}H-NMR (D_2O/NaOH, 600 MHz)

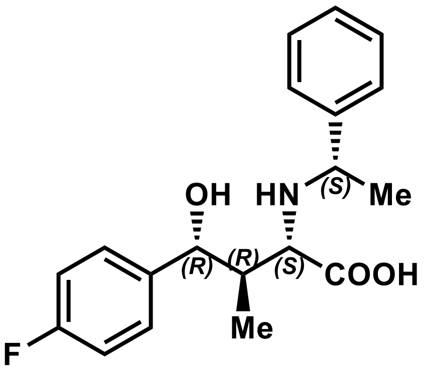

major
(2*S*,3*R*,4*R*)-**S7**

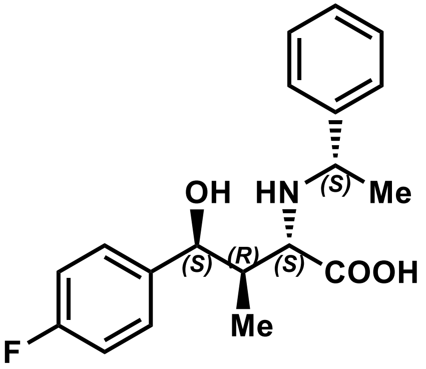

minor
(2*S*,3*R*,4*S*)-**S7**

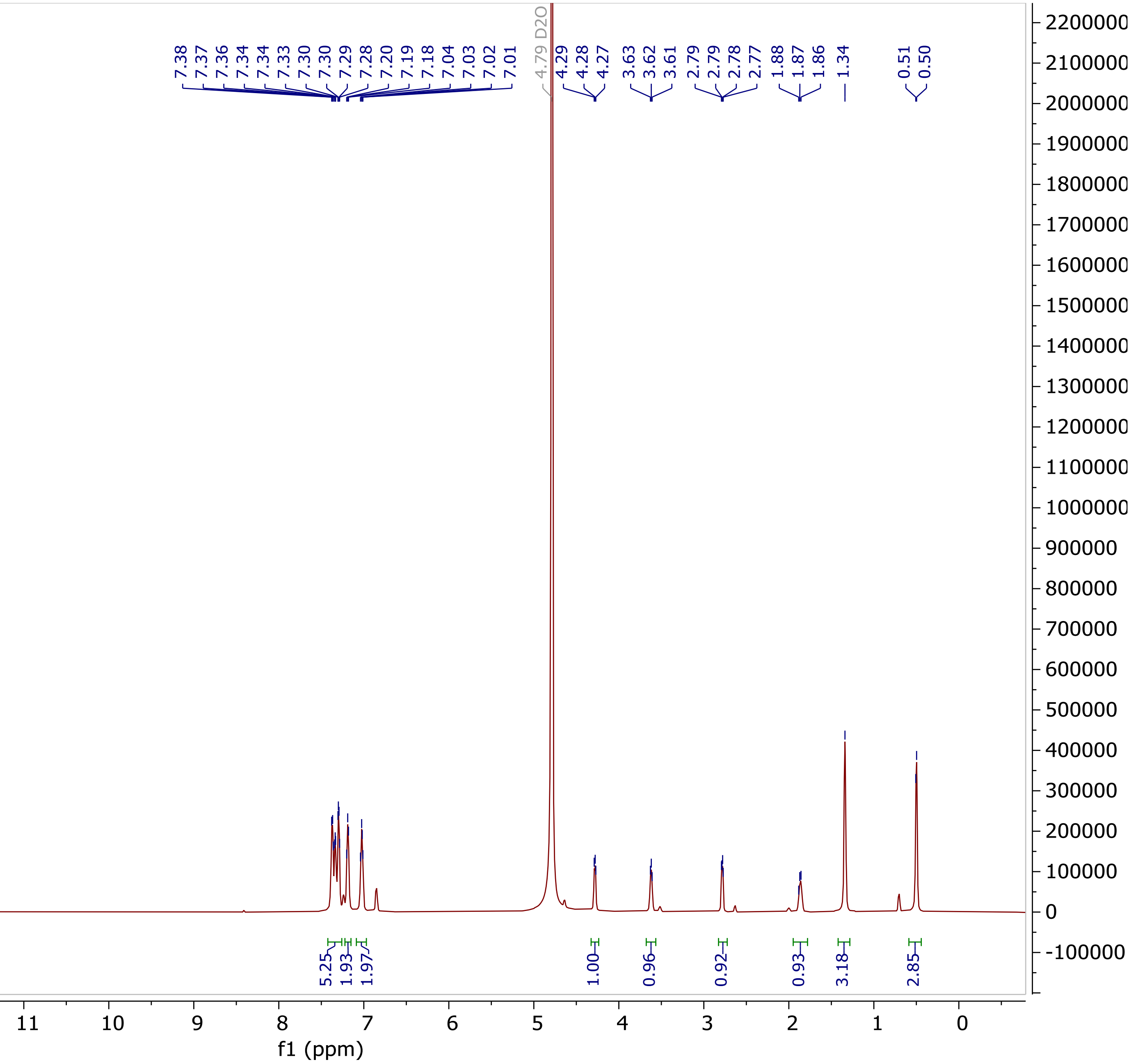

(2*S*,3*R*,4*R*)-S7 ^{13}C-NMR (D_2O/NaOH, 151 MHz)

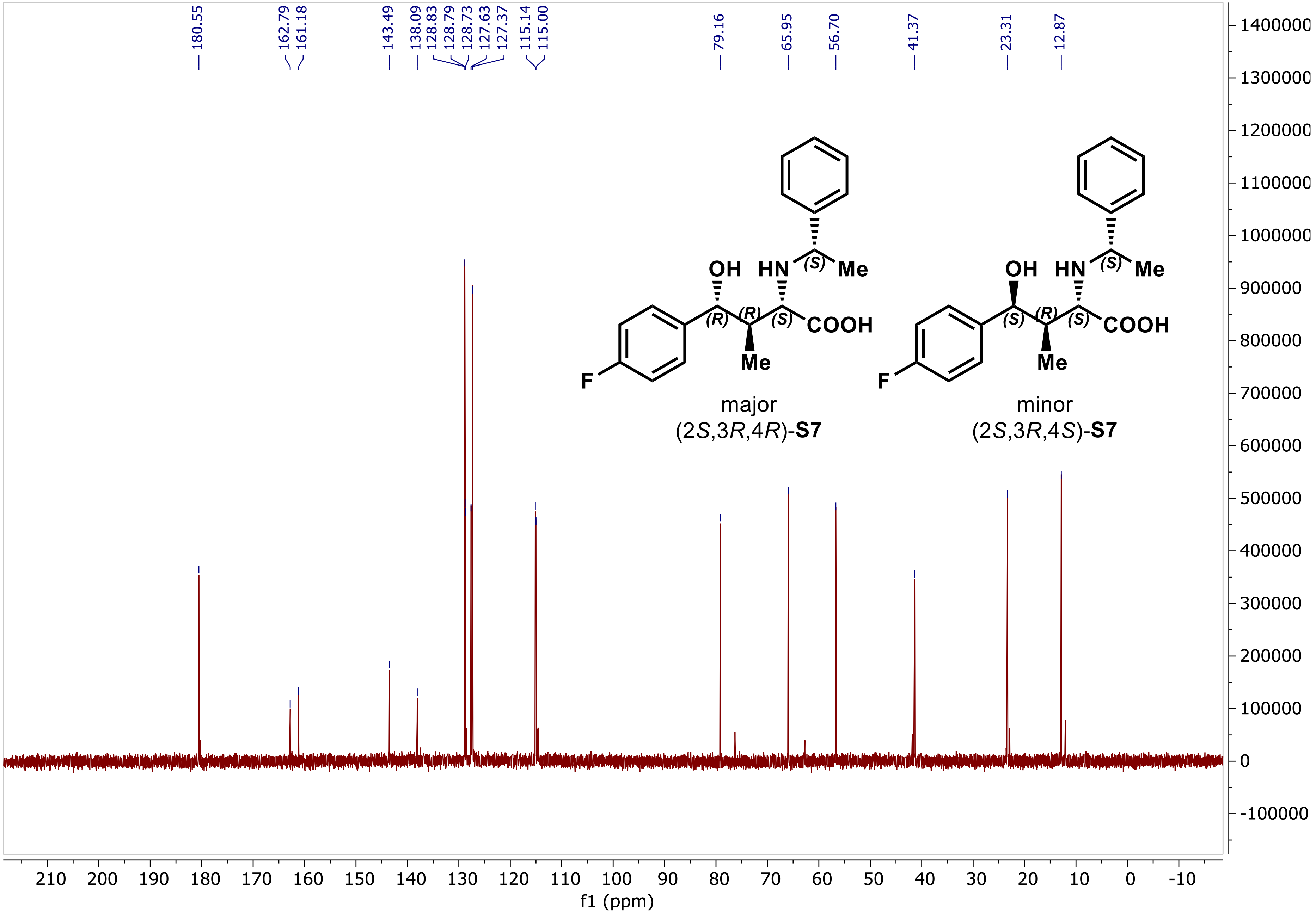

(2*S*,3*R*,4*R*)-S7 ^{19}F-NMR (D_2O/NaOH, 563 MHz)

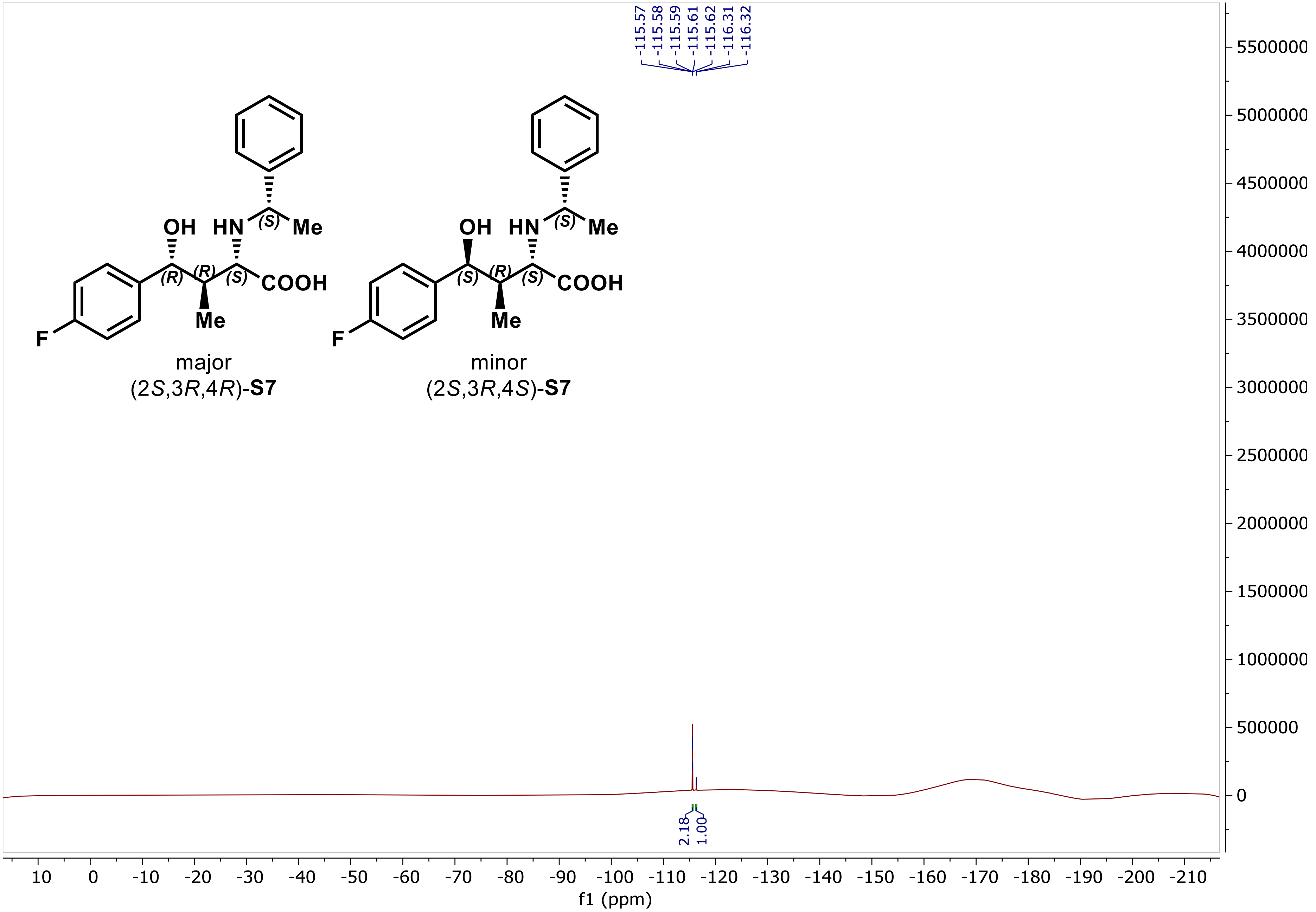

(2*S*,3*R*,4*S*)-14 ^{1}H-NMR (D_2O/LiOH, 600 MHz)

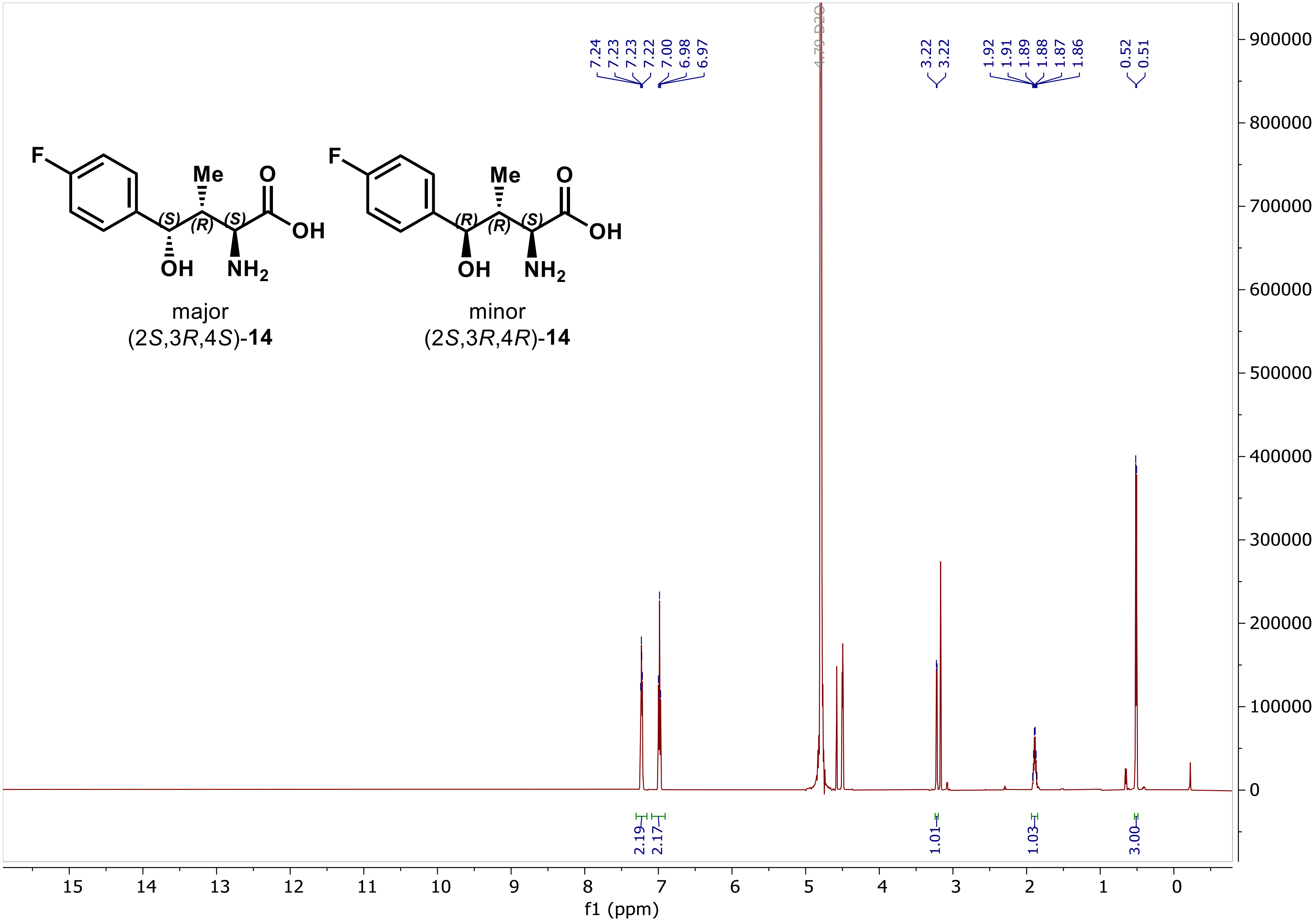

(2*S*,3*R*,4*S*)-14 ^{13}C-NMR (D_2O/LiOH, 151 MHz)

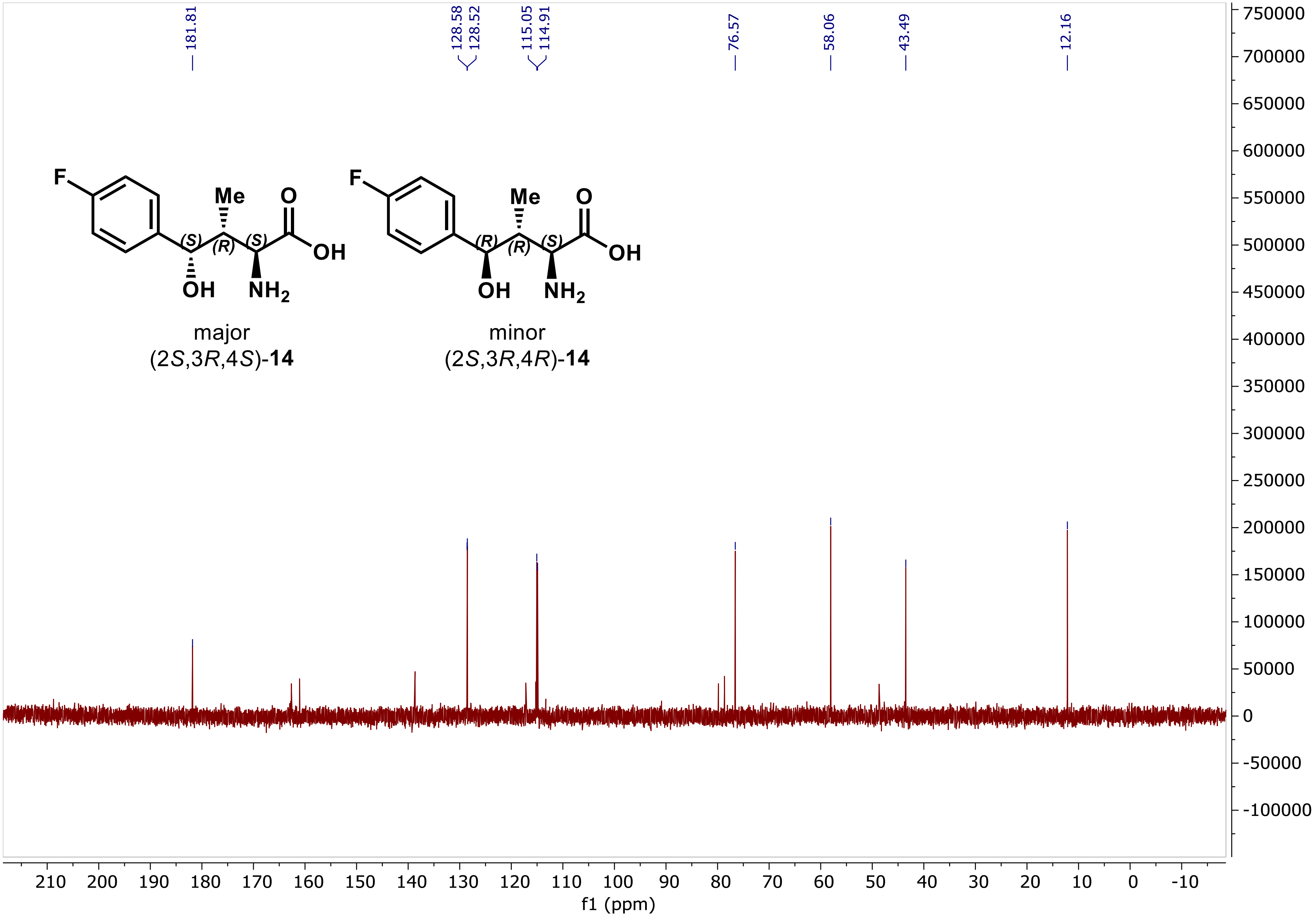

(2*S*,3*R*,4*S*)-14 ^{19}F-NMR (D_2O/LiOH/FCH_2CN, 563 MHz)

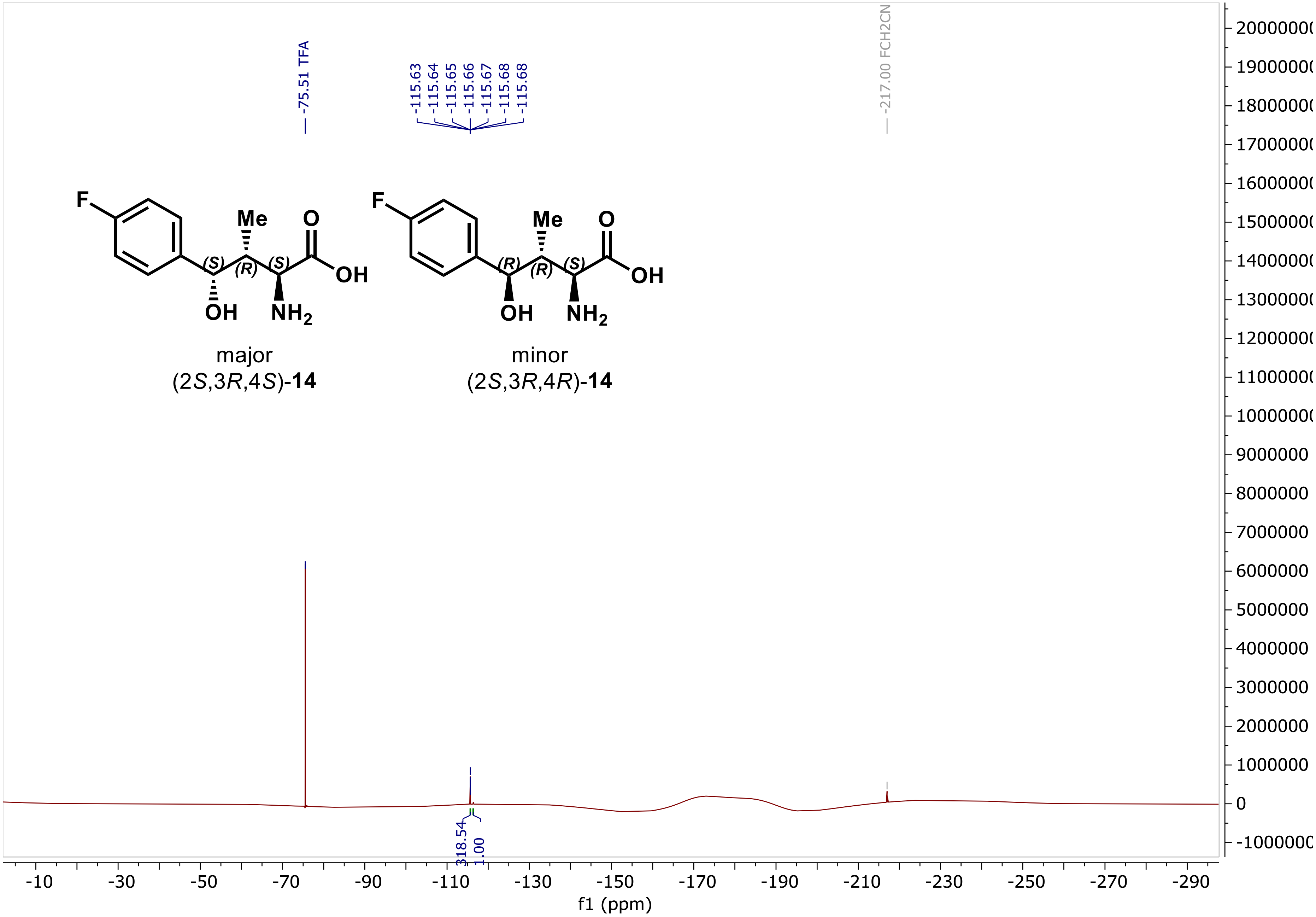

(2*S*,3*R*,4*R*)-14 ^{1}H-NMR (D_2O/LiOH, 600 MHz)

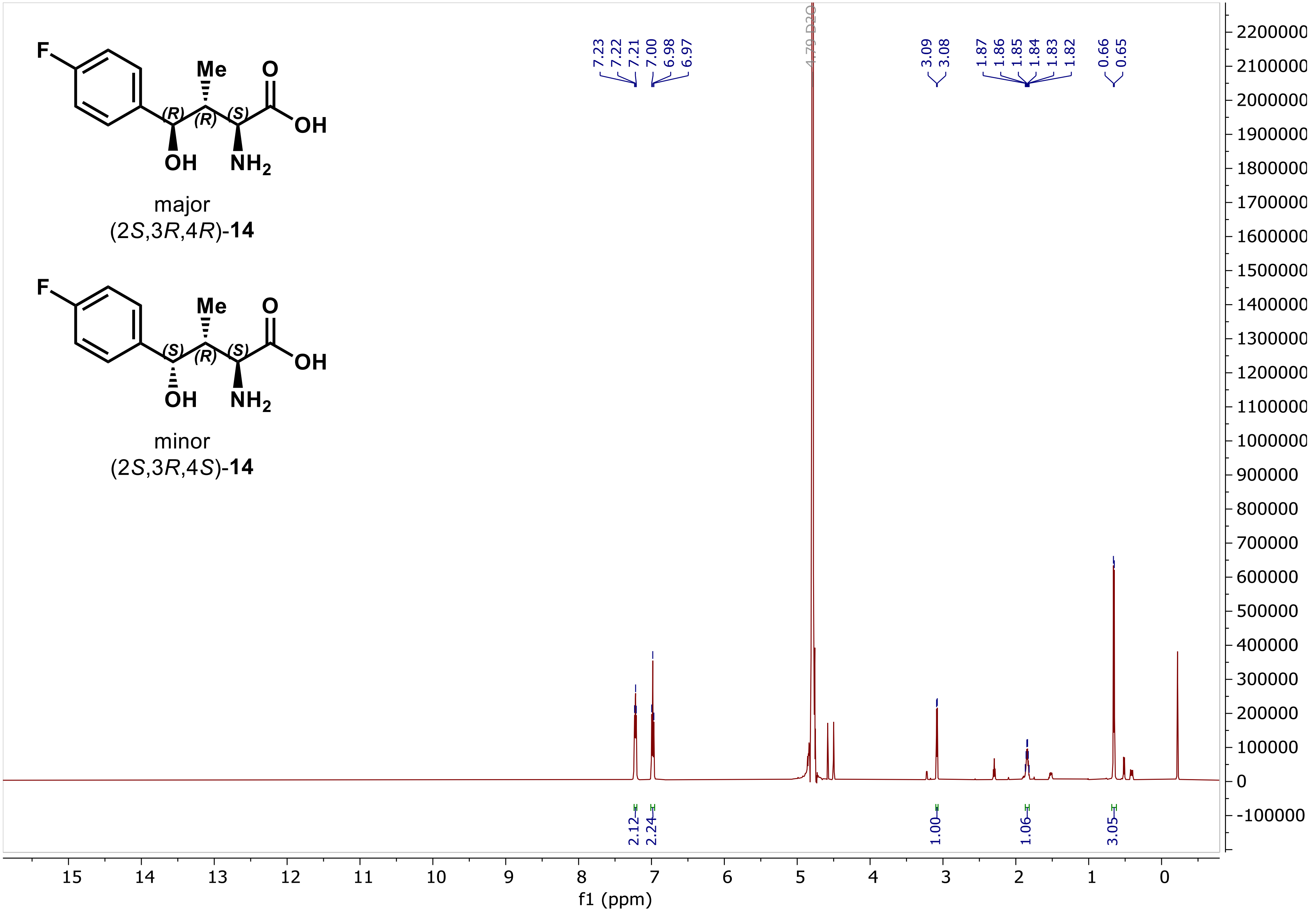

(2*S*,3*R*,4*R*)-14 ^{13}C-NMR (D_2O/LiOH, 151 MHz)

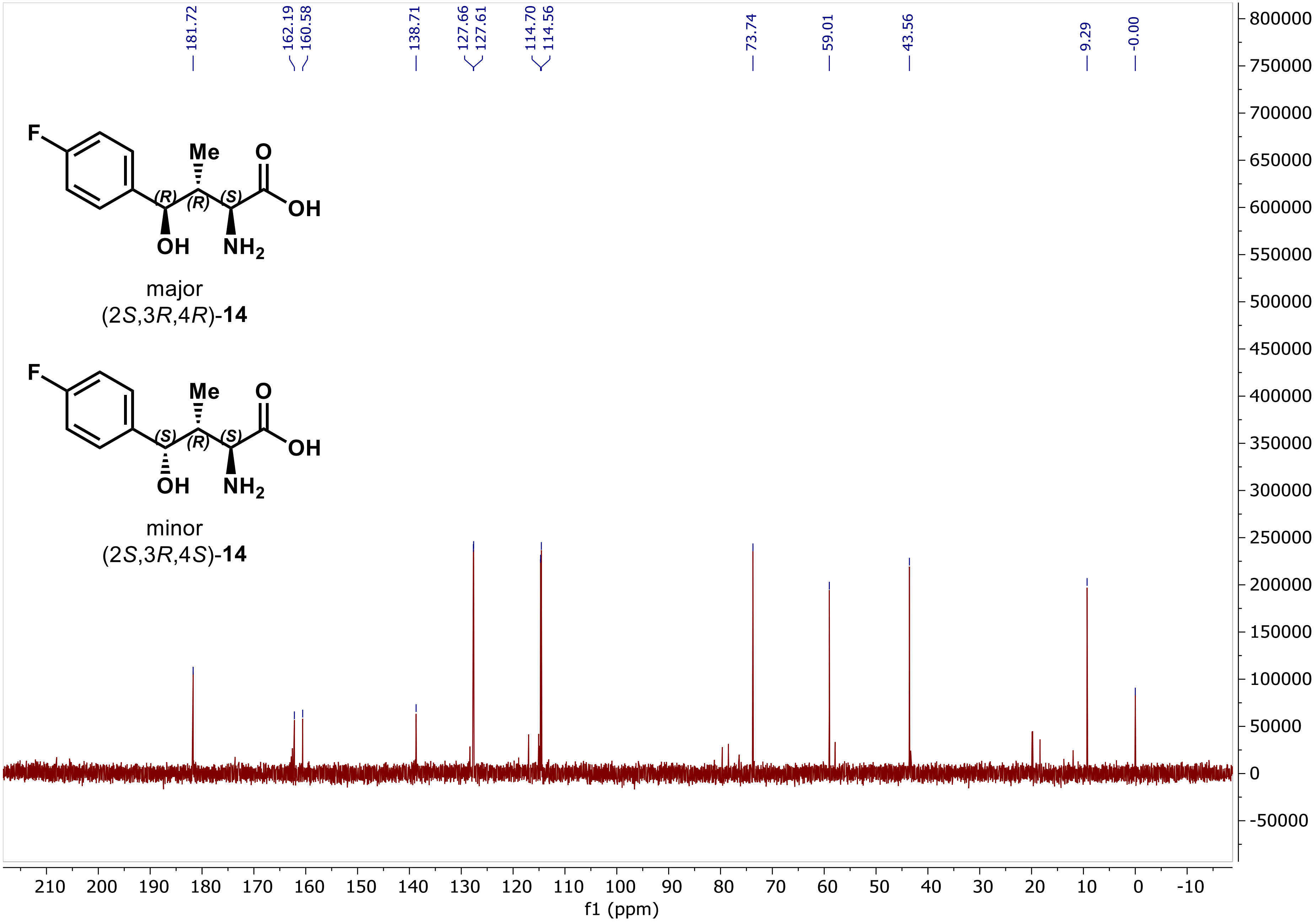

major
(2*S*,3*R*,4*R*)-**14**

minor
(2*S*,3*R*,4*S*)-**14**

(2*S*,3*R*,4*R*)-14 ^{19}F-NMR (D_2O/LiOH/FCH_2CN, 563 MHz)

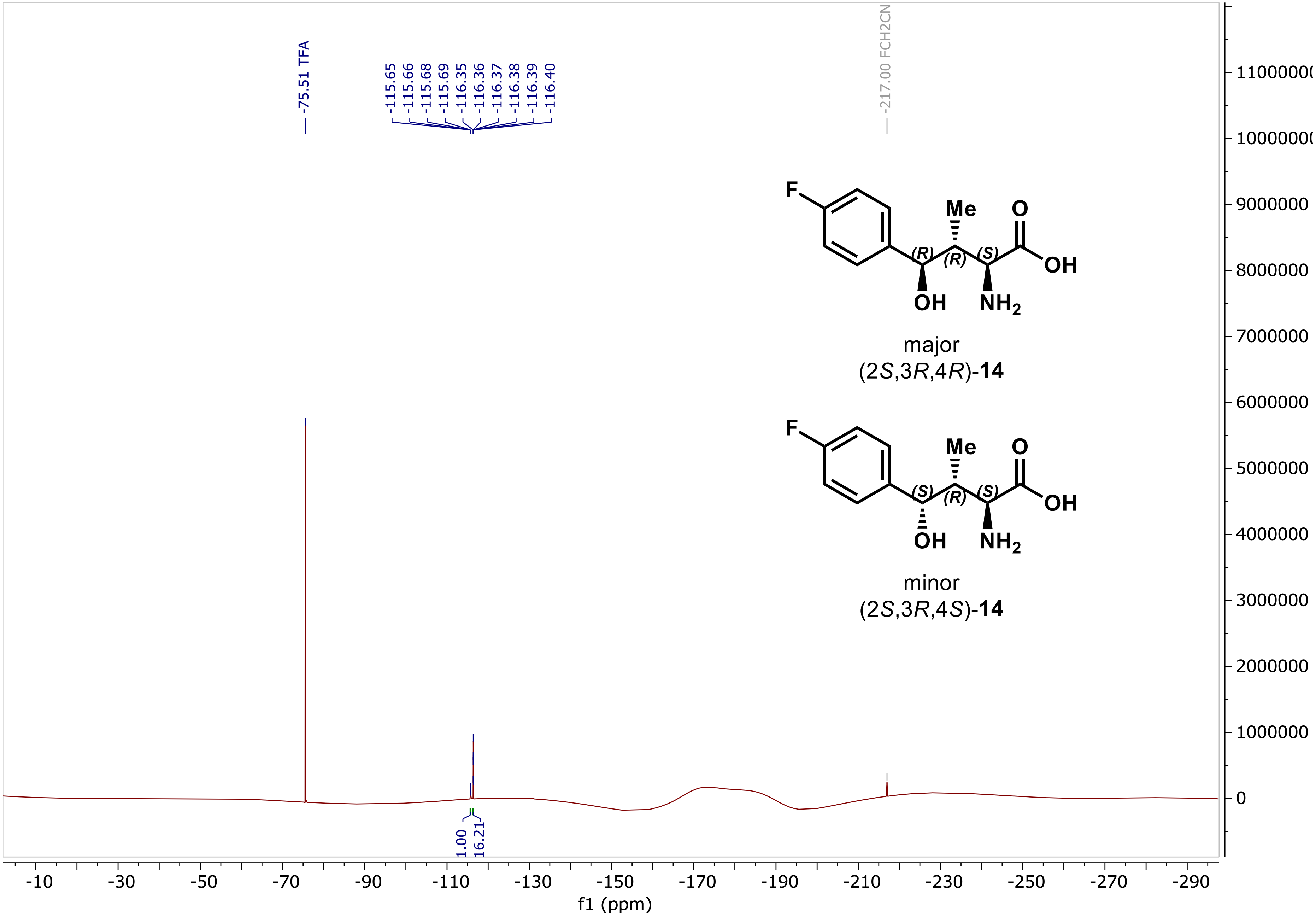

S8 ^{1}H-NMR ($CDCl_3$, 599 MHz)

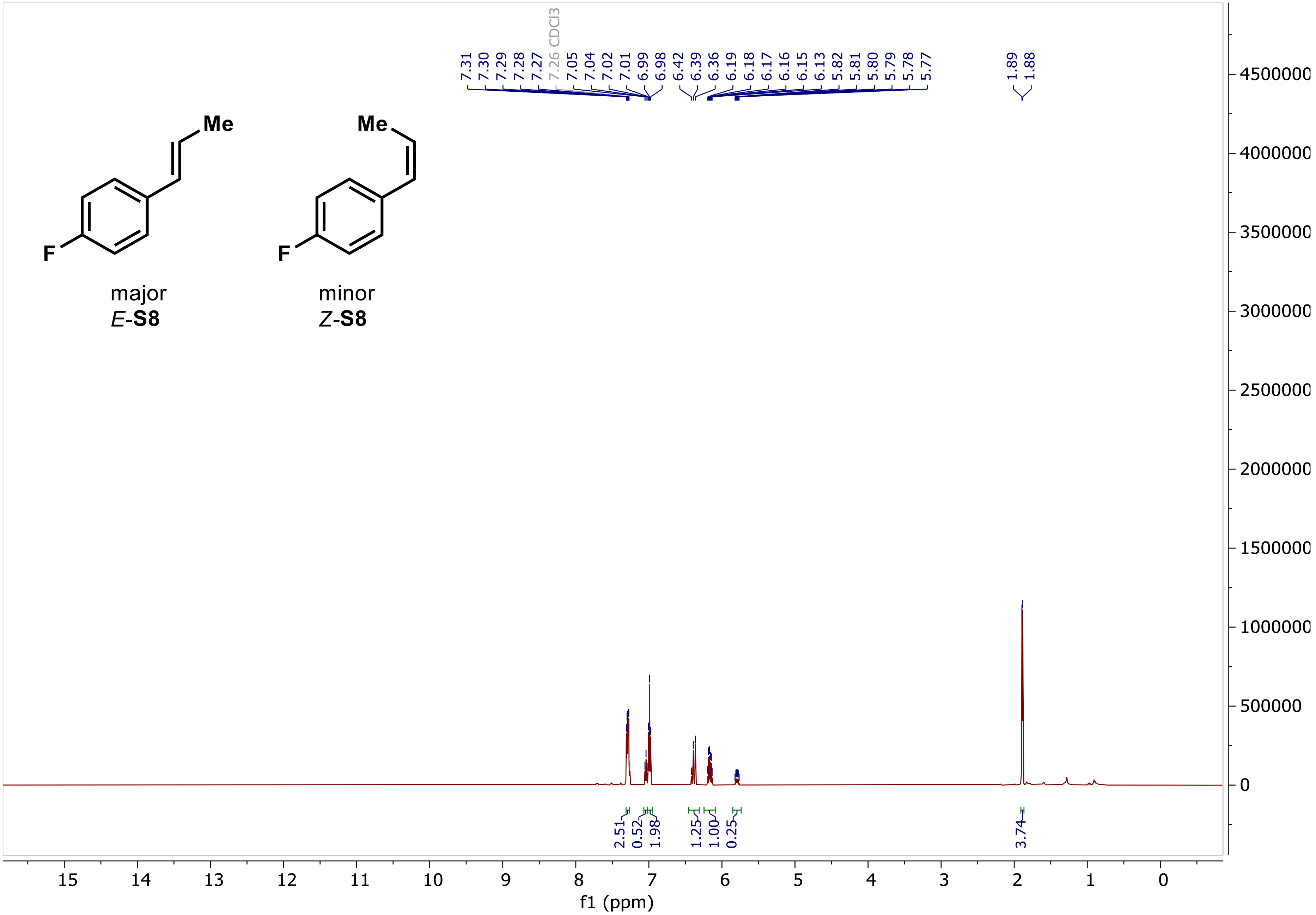

S8 ^{13}C-NMR ($CDCl_3$, 151 MHz)

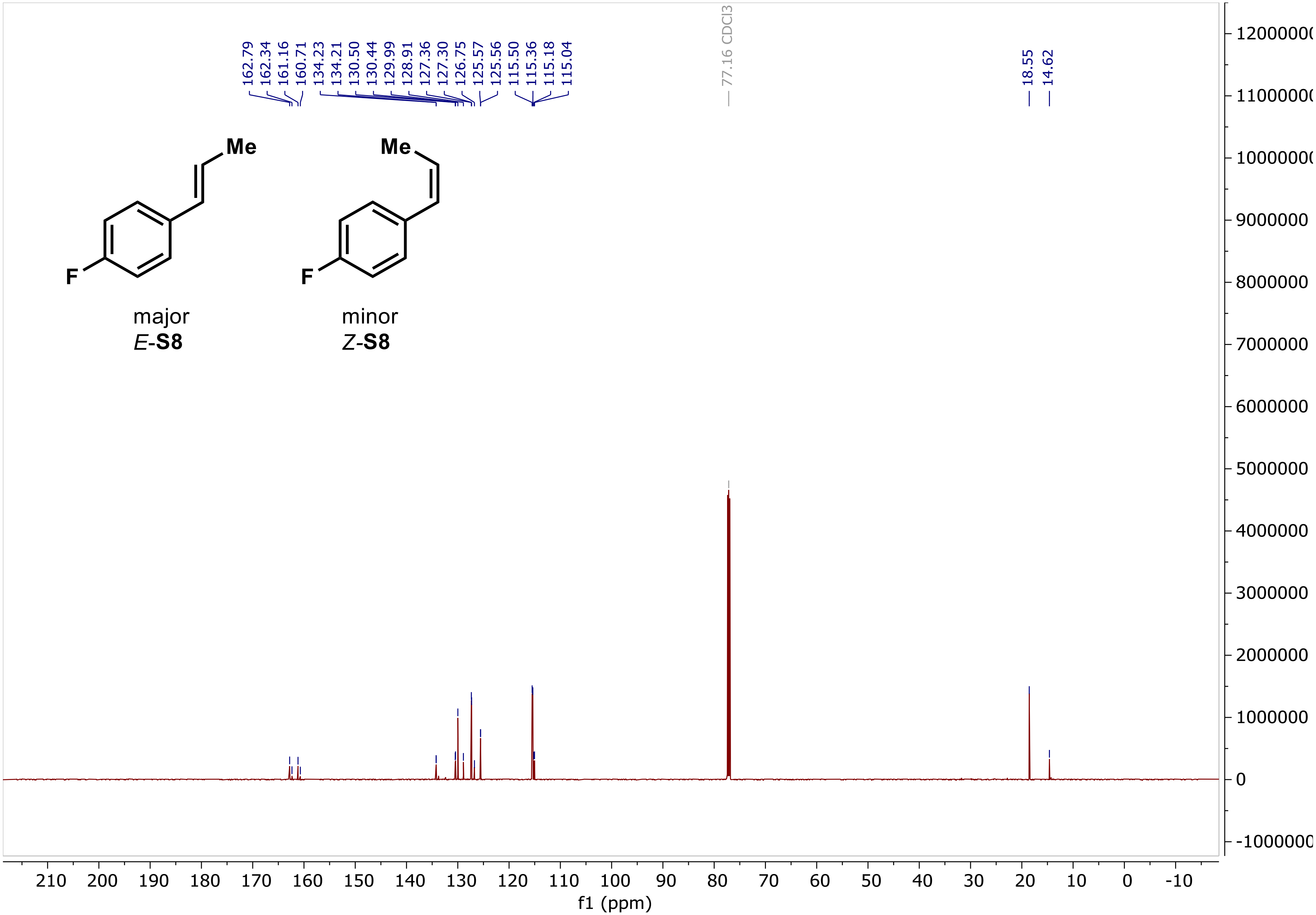

S8 ^{19}F-NMR ($CDCl_3$, 563 MHz)

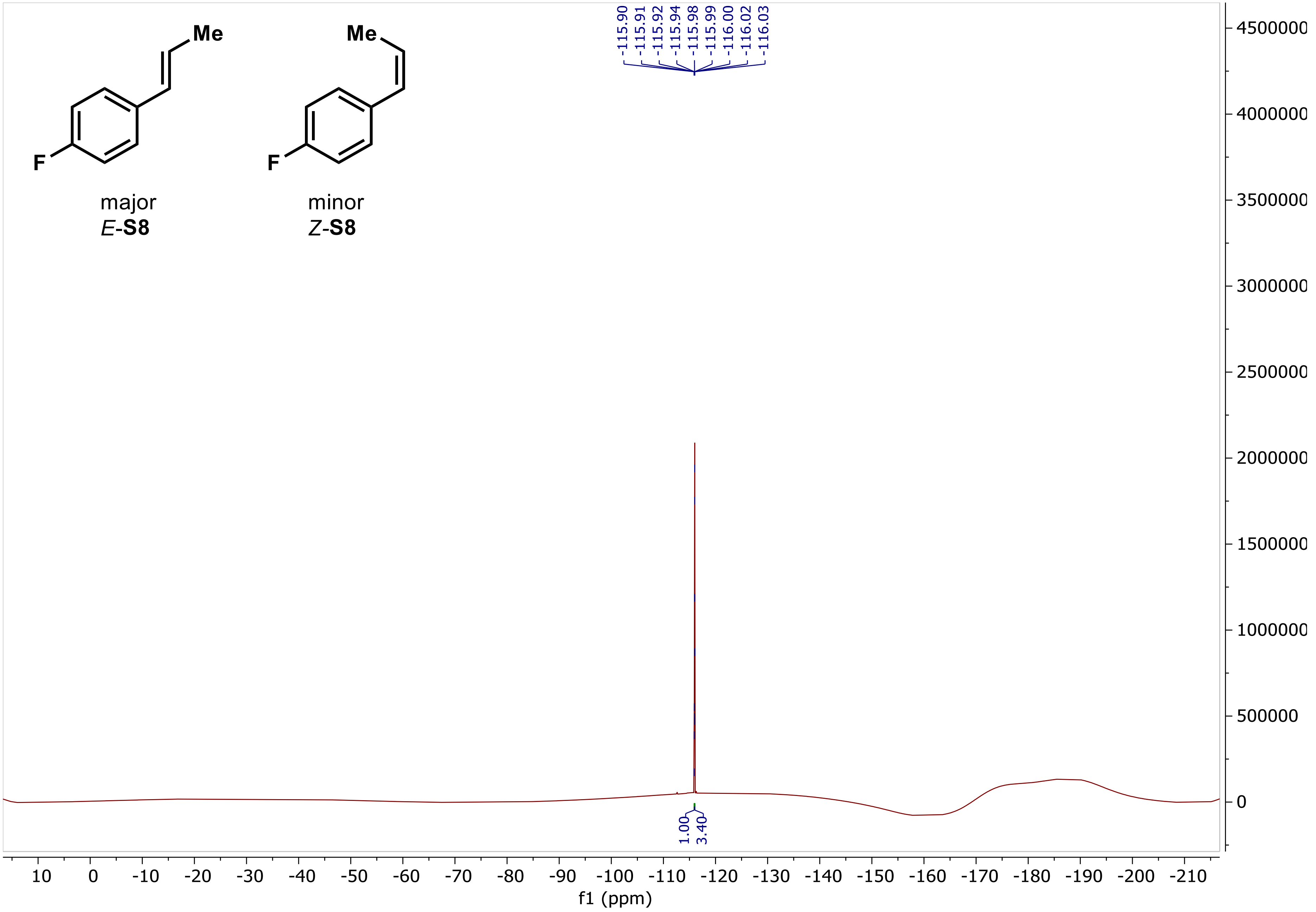

S9 ^{1}H-NMR (CD_3OD, 599 MHz)

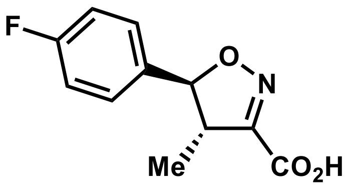

S9

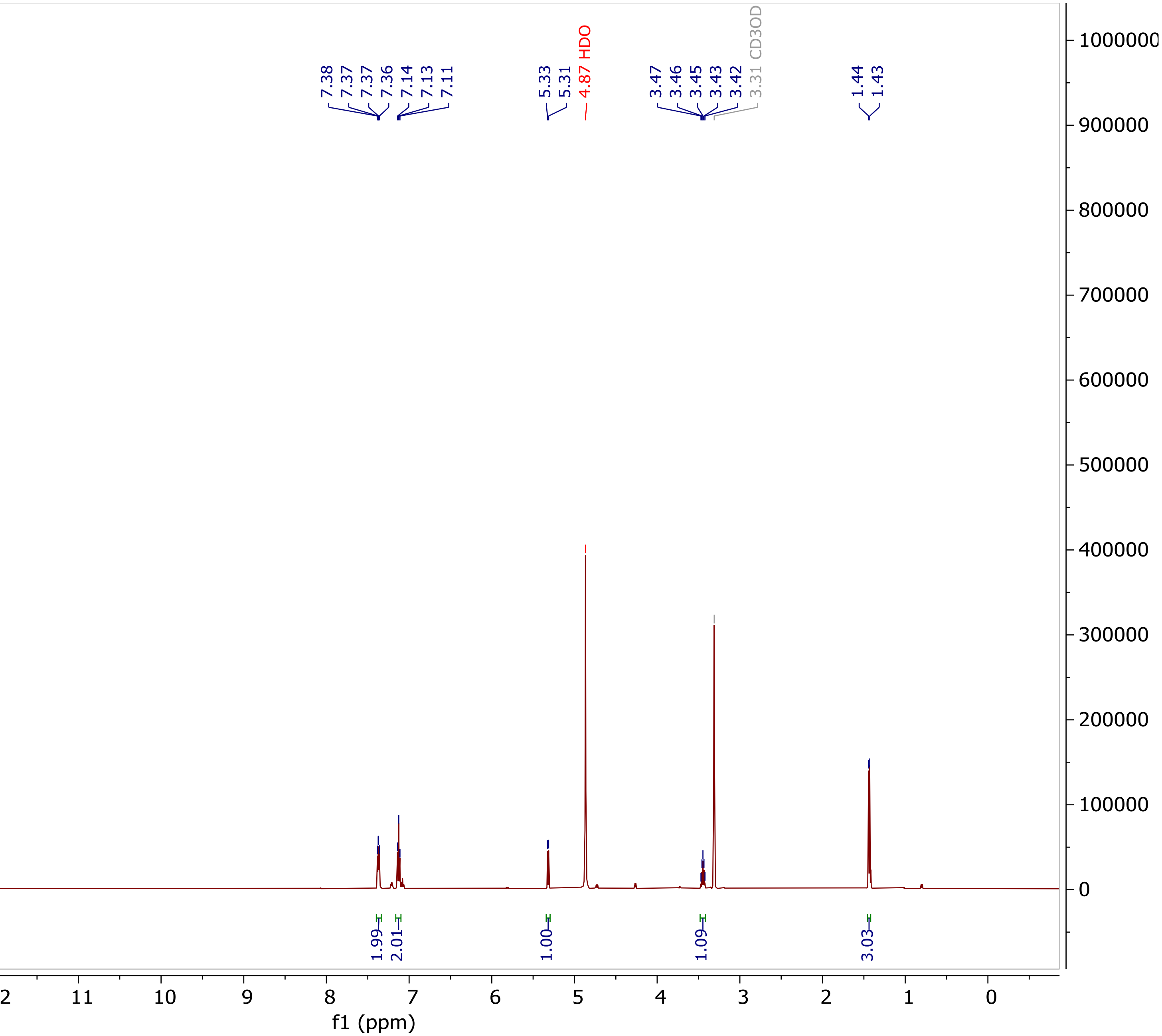

S9 ^{13}C-NMR (CD_3OD, 151 MHz)

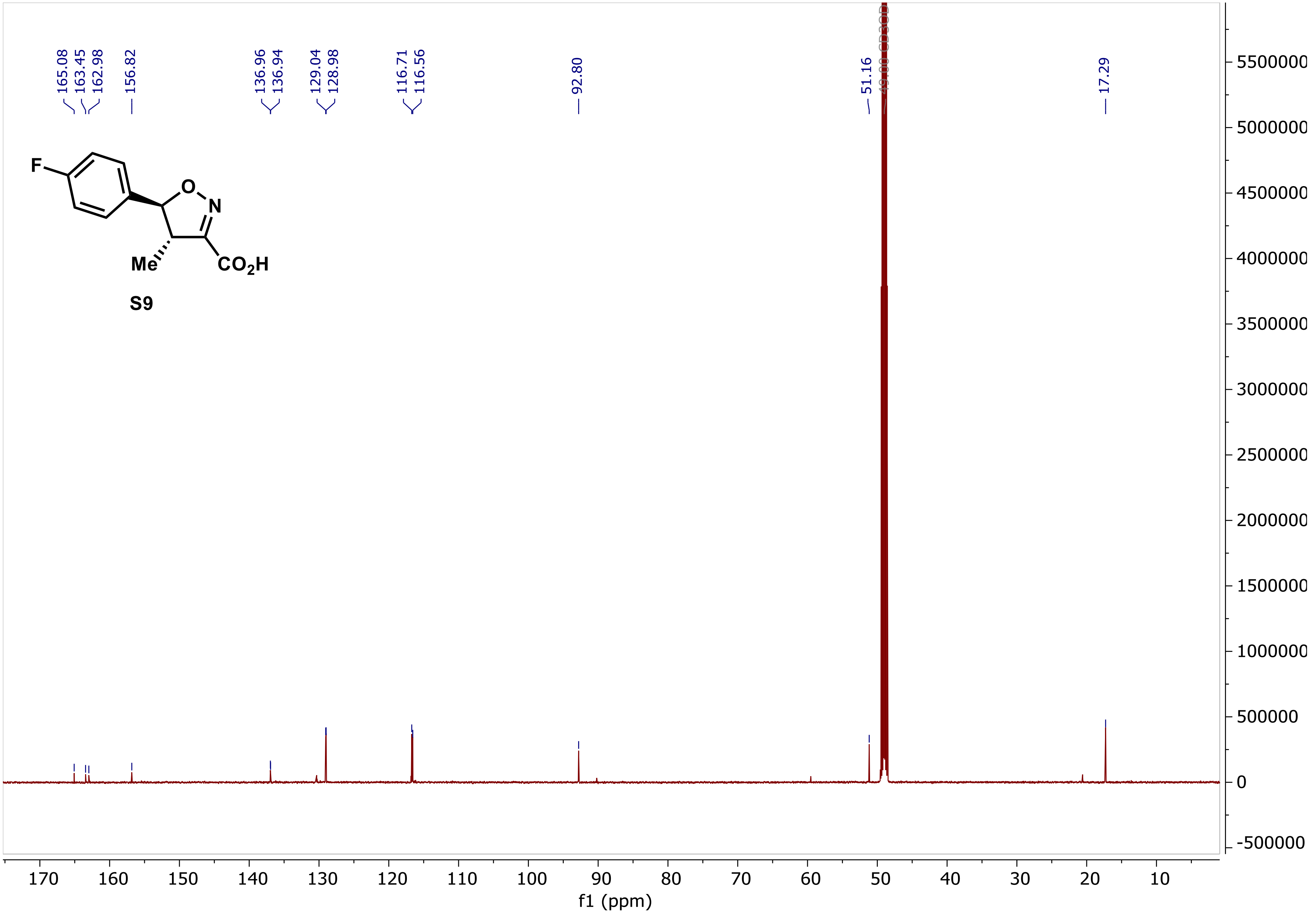

S9 ^{19}F-NMR (CD_3OD, 563 MHz)

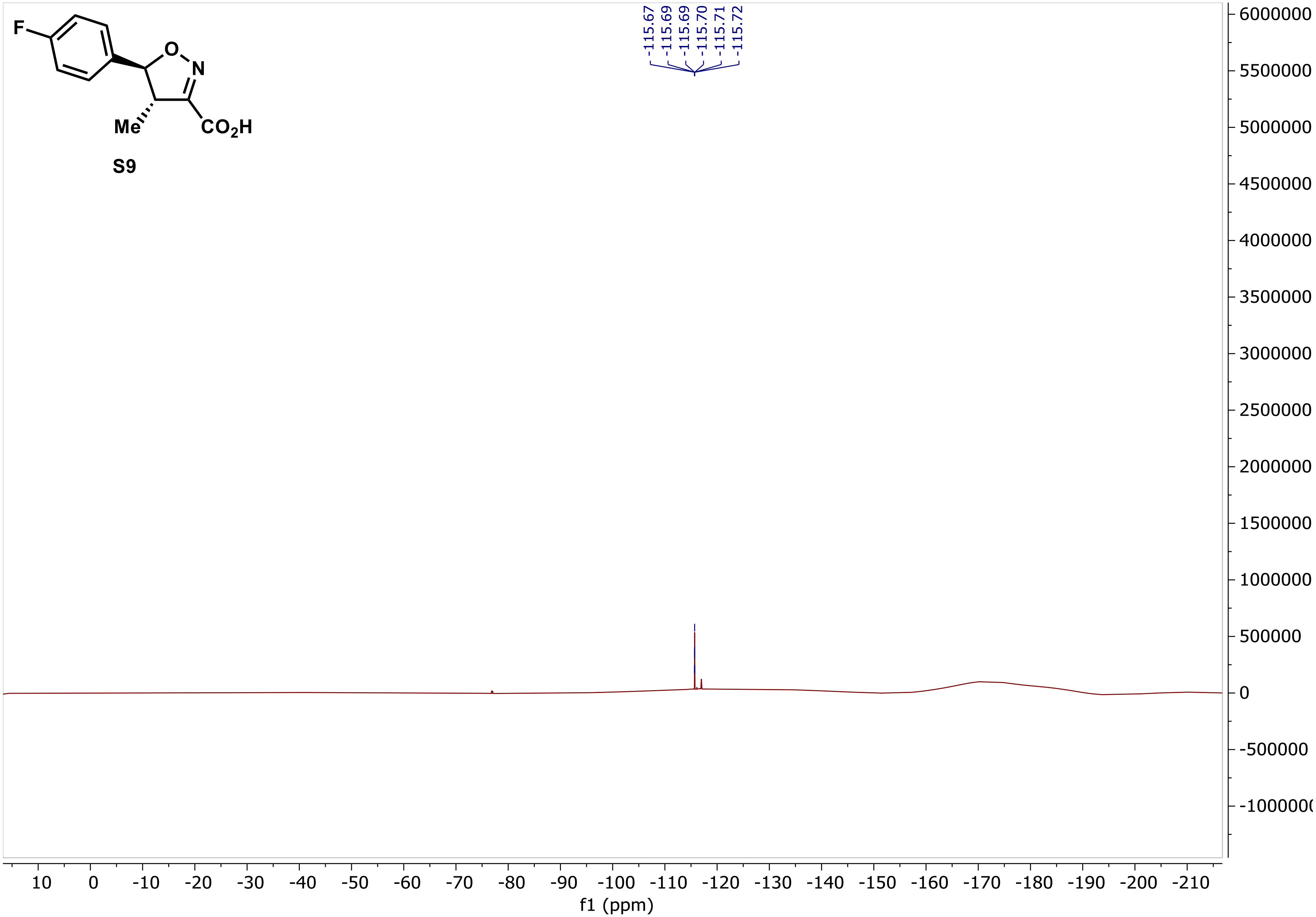

S9 NOESY-NMR (CD_3OD, 599 MHz)

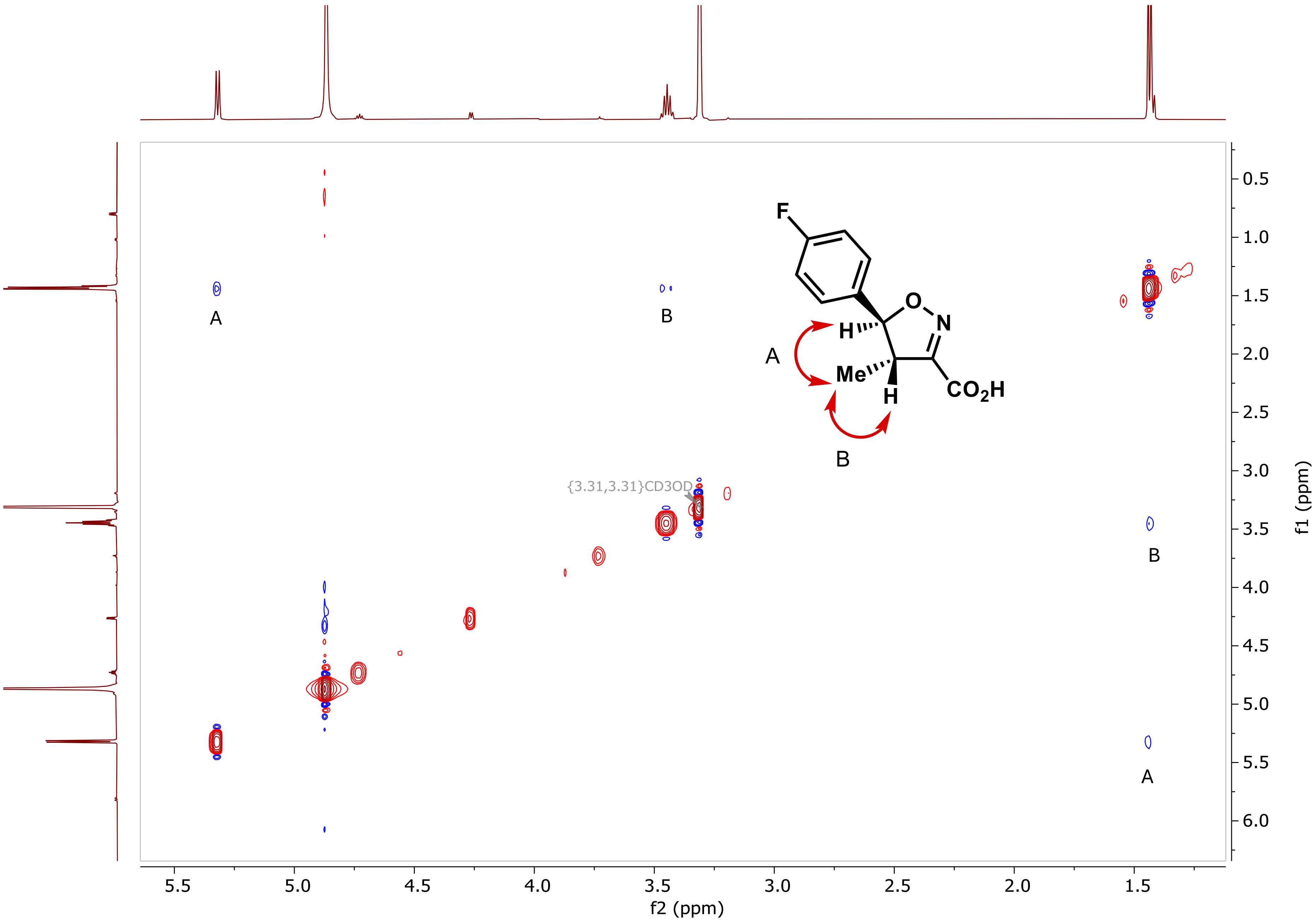

(2*S*,3*S*,4*S*)-14 ^{1}H-NMR (D_2O/LiOH, 599 MHz)

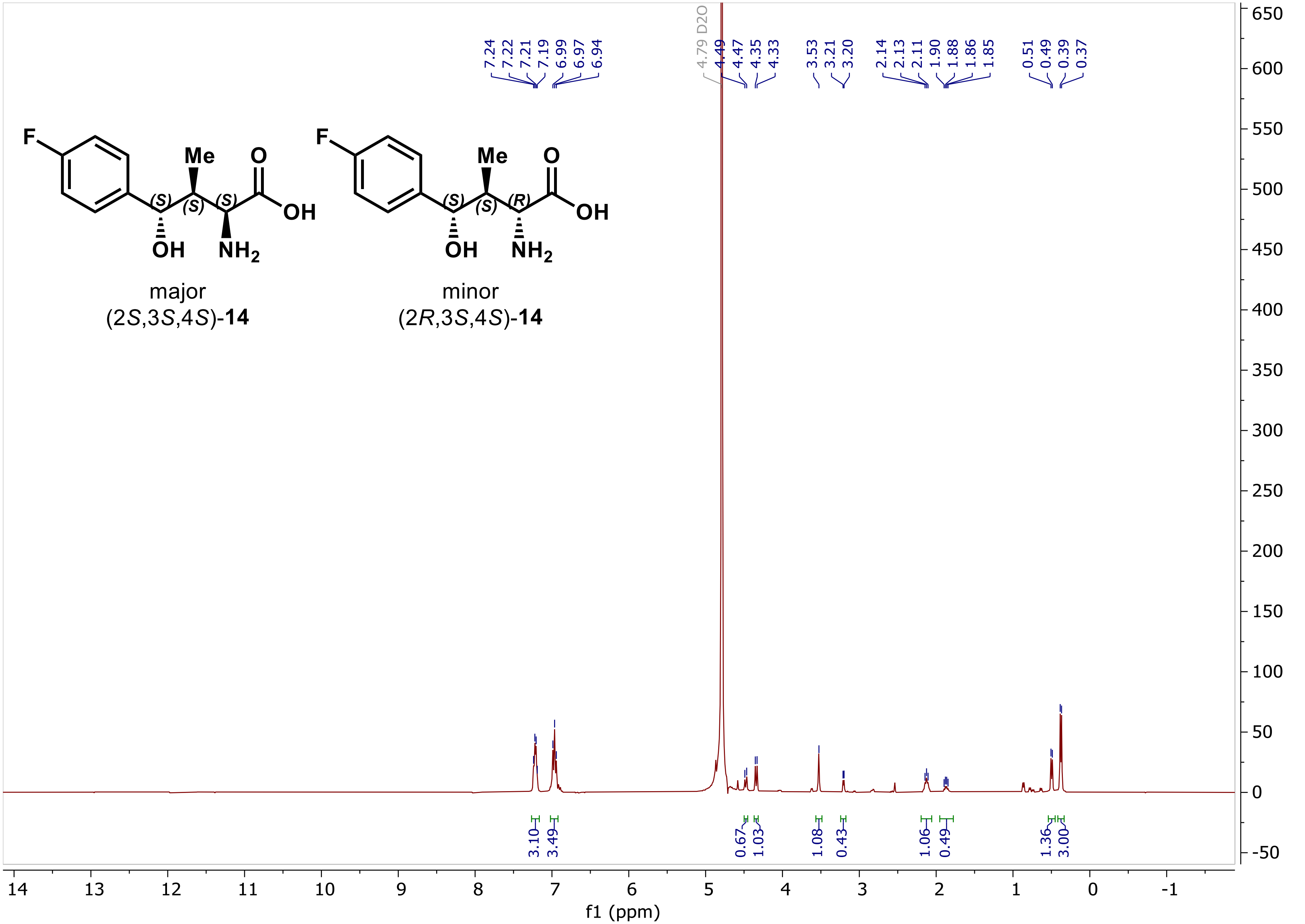

(2*S*,3*S*,4*S*)-14 ^{13}C-NMR (D_2O/LiOH, 151 MHz)

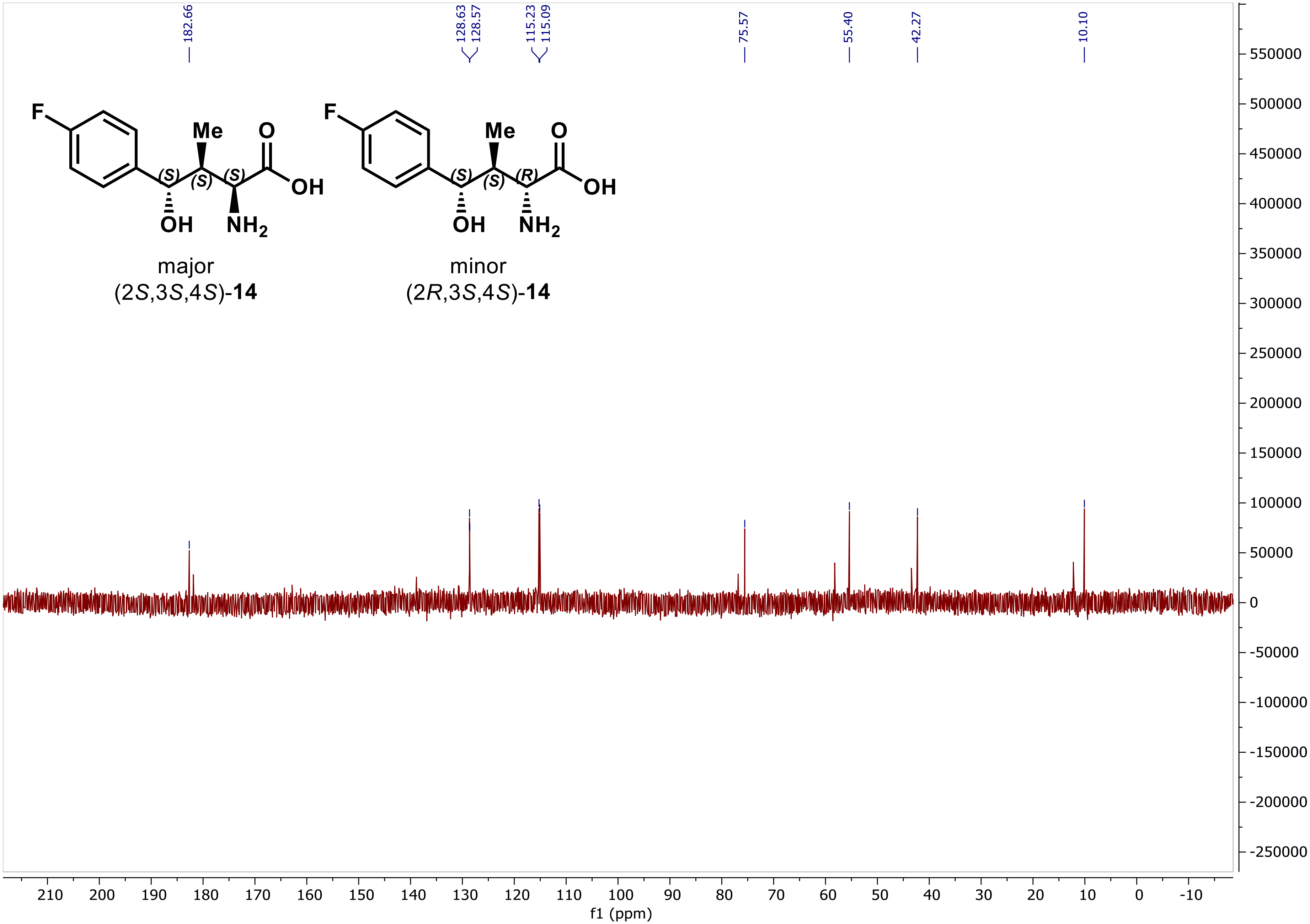

(2*S*,3*S*,4*S*)-14 ^{19}F-NMR (D_2O/LiOH/FCH_2CN, 563 MHz)

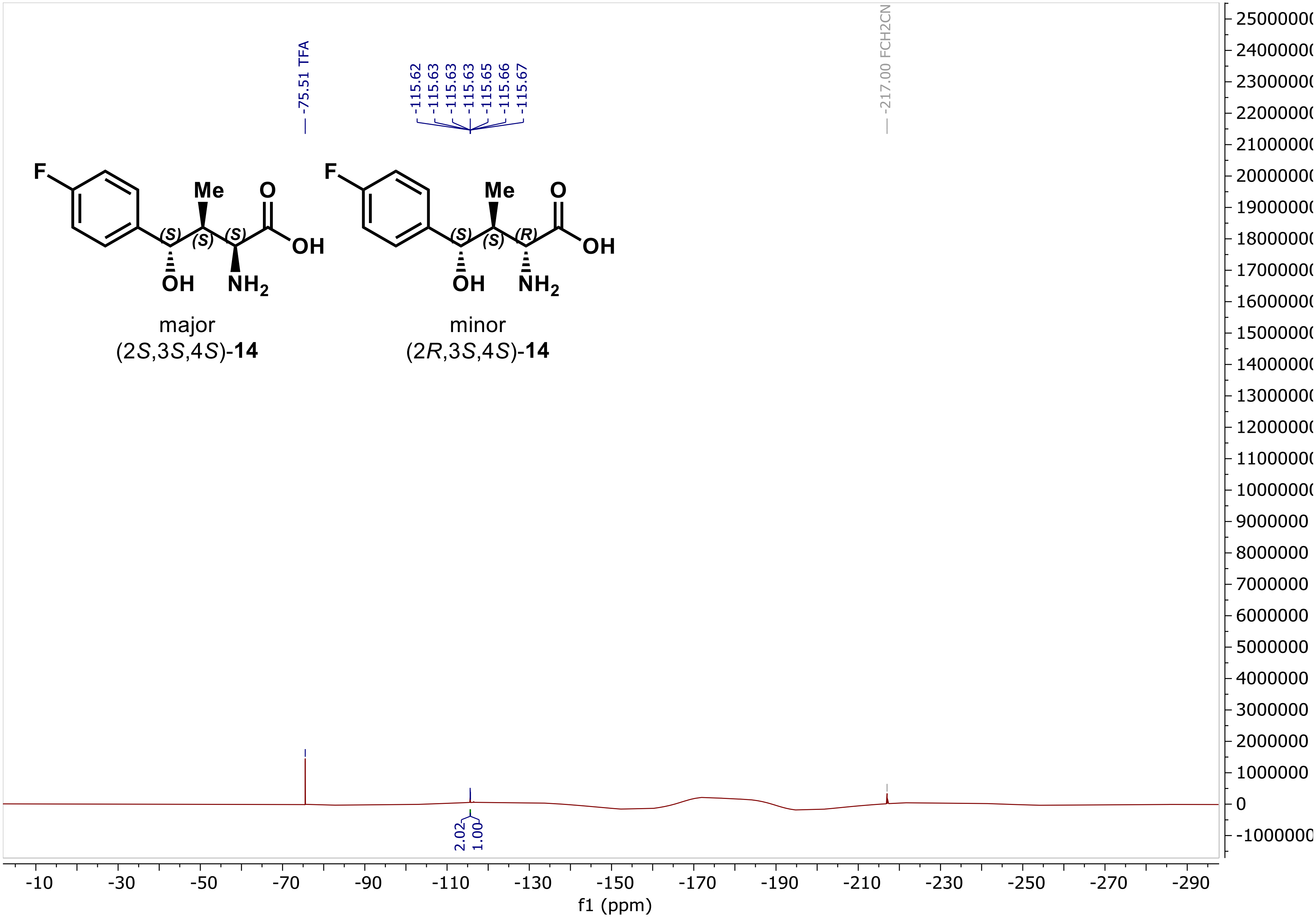

S10 ^{1}H-NMR ($CDCl_3$, 599 MHz)

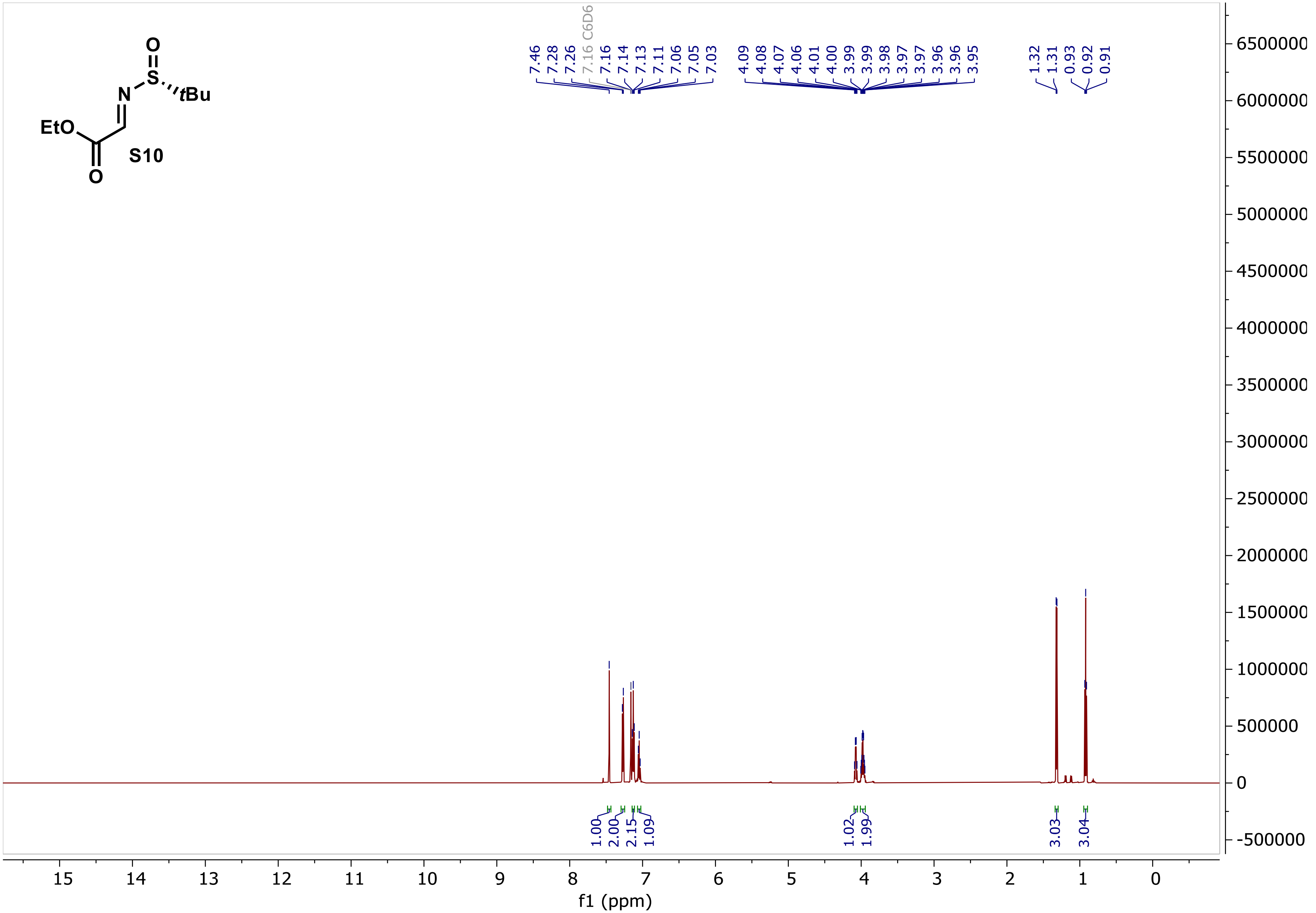

S10 ^{13}C-NMR ($CDCl_3$, 151 MHz)

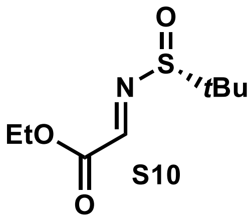

S10

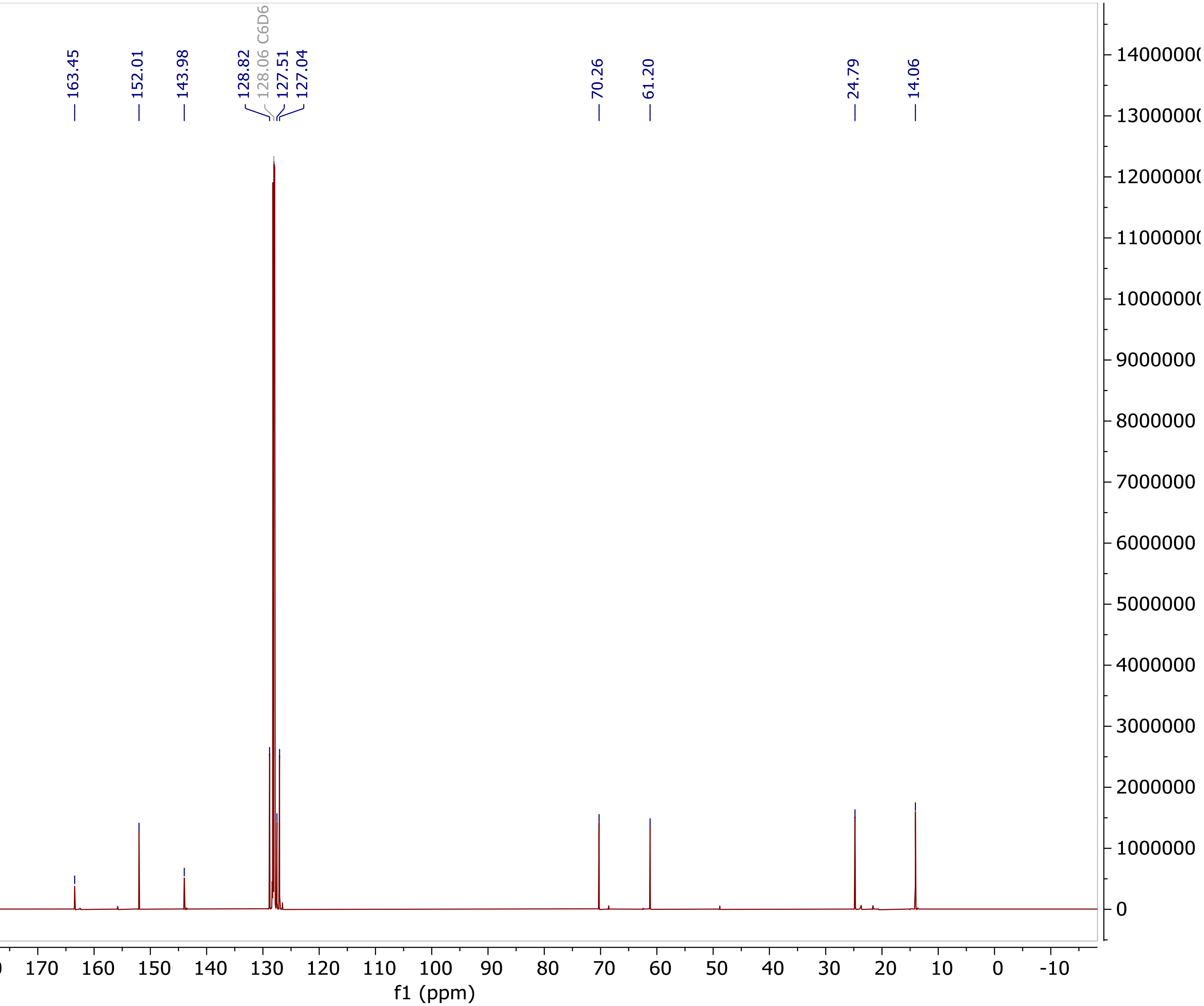

S11 ¹H-NMR ($CDCl_3$, 599 MHz)

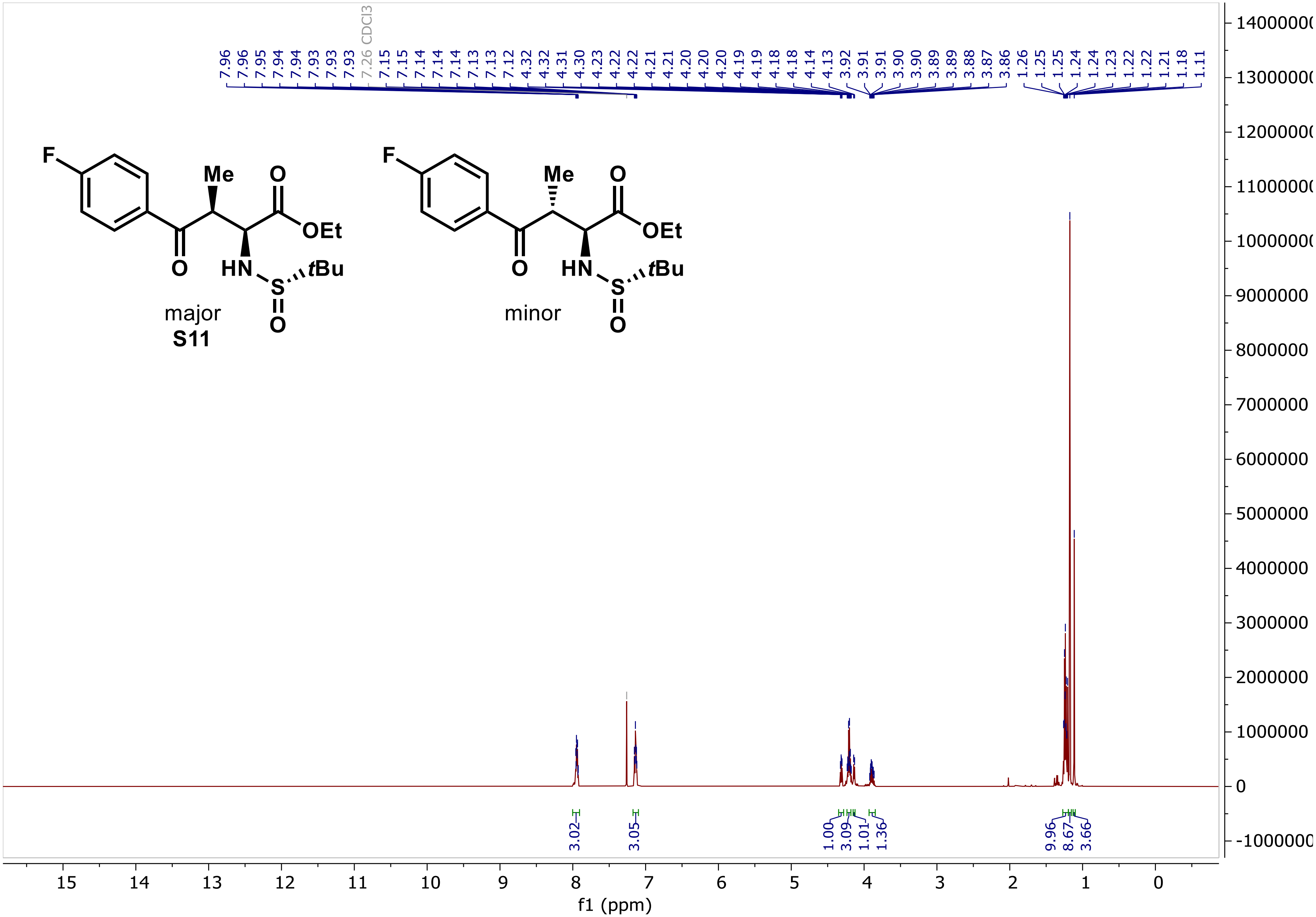

S11 ^{13}C-NMR ($CDCl_3$, 151 MHz)

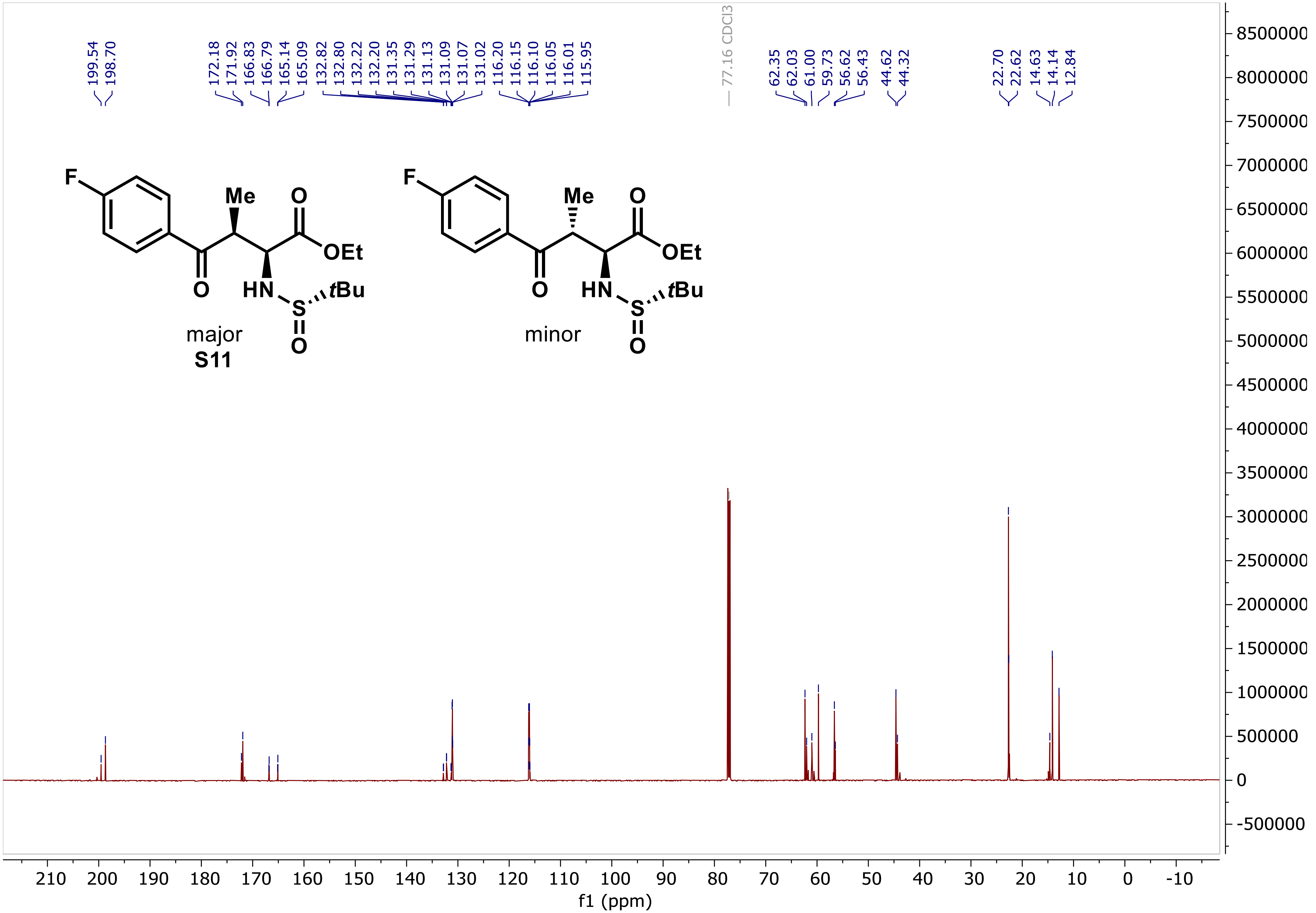

S11 ^{19}F-NMR ($CDCl_3$, 563 MHz)

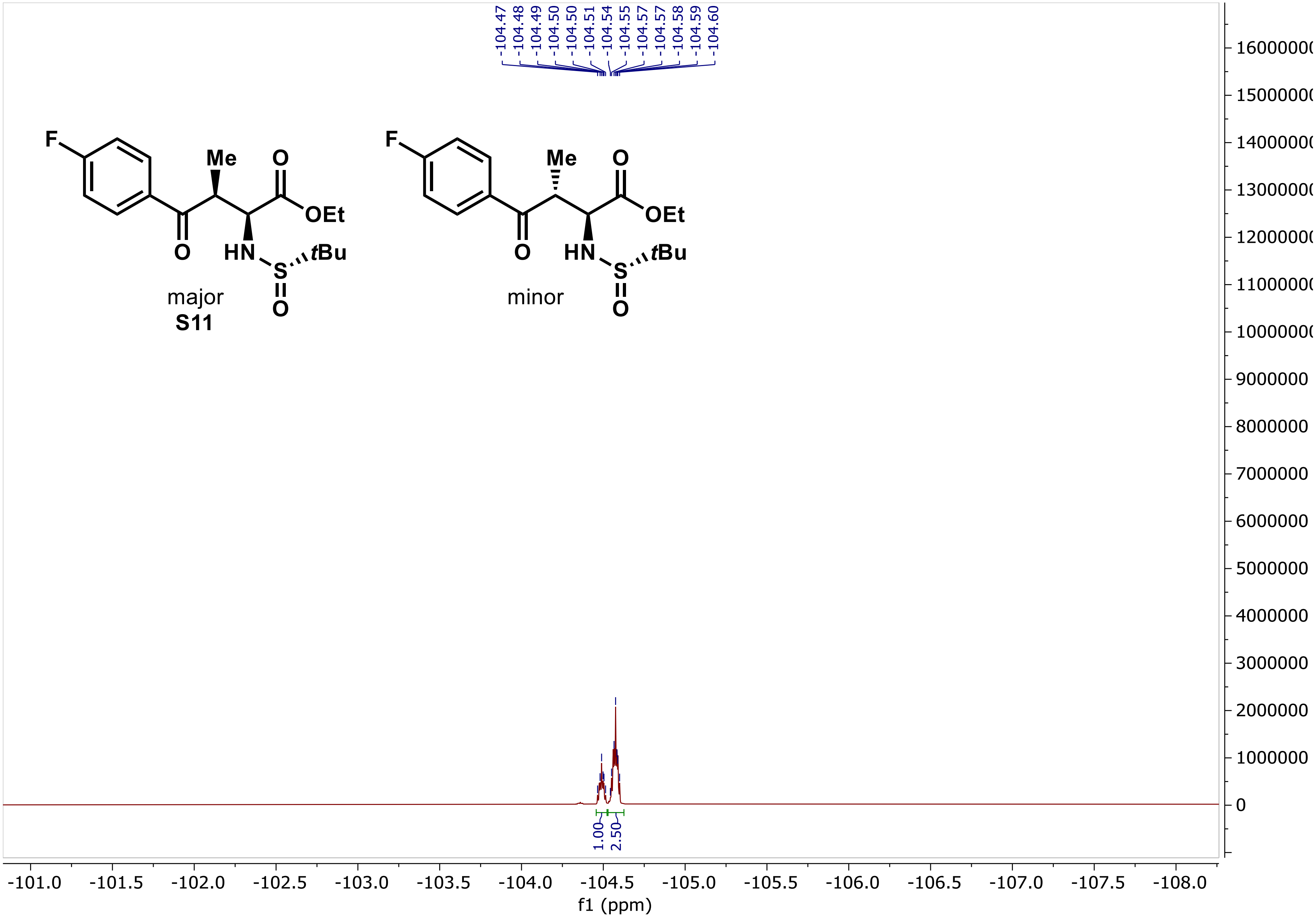

S12 ^{1}H-NMR ($CDCl_3$, 599 MHz)

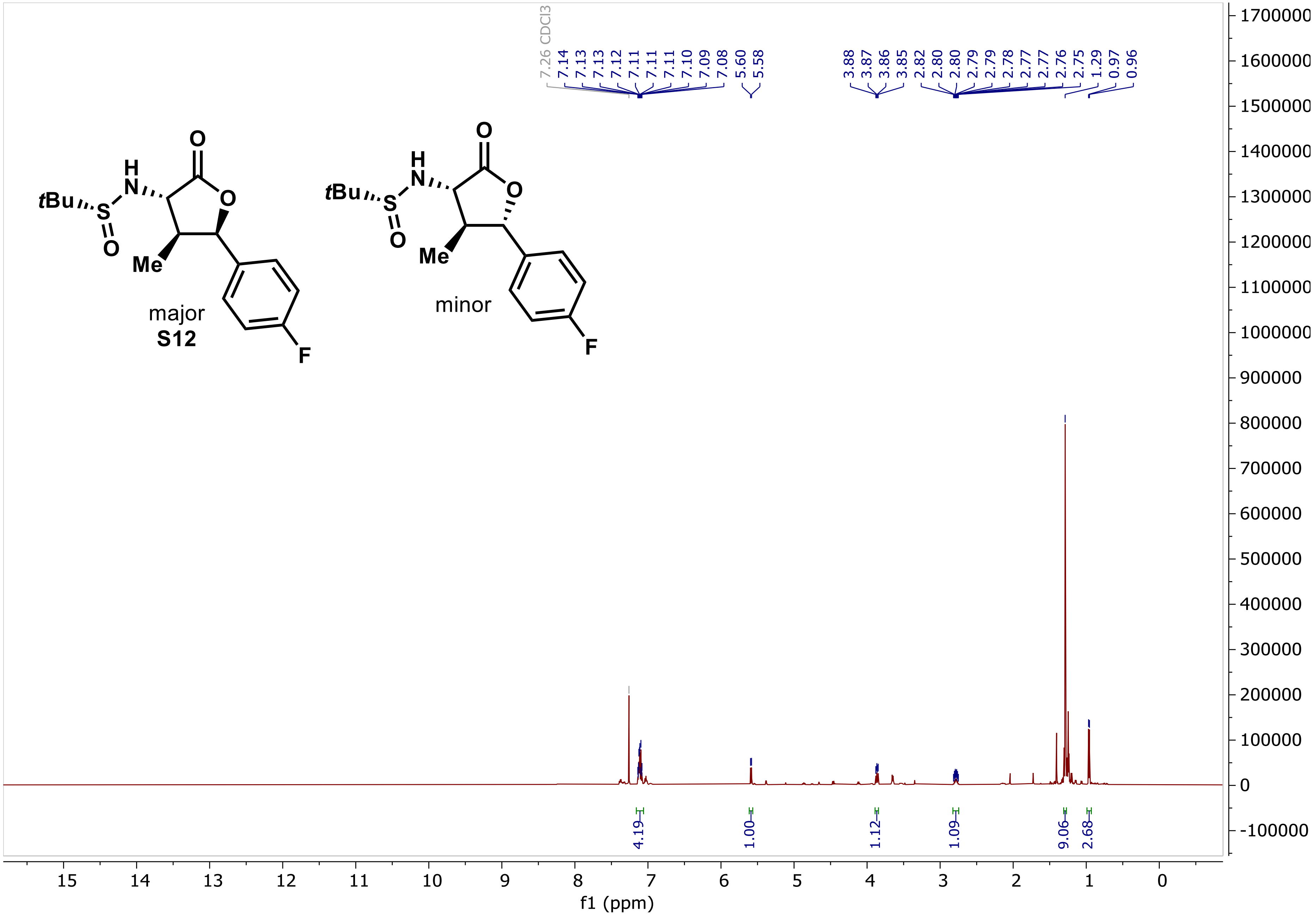

S12 ^{13}C-NMR ($CDCl_3$, 151 MHz)

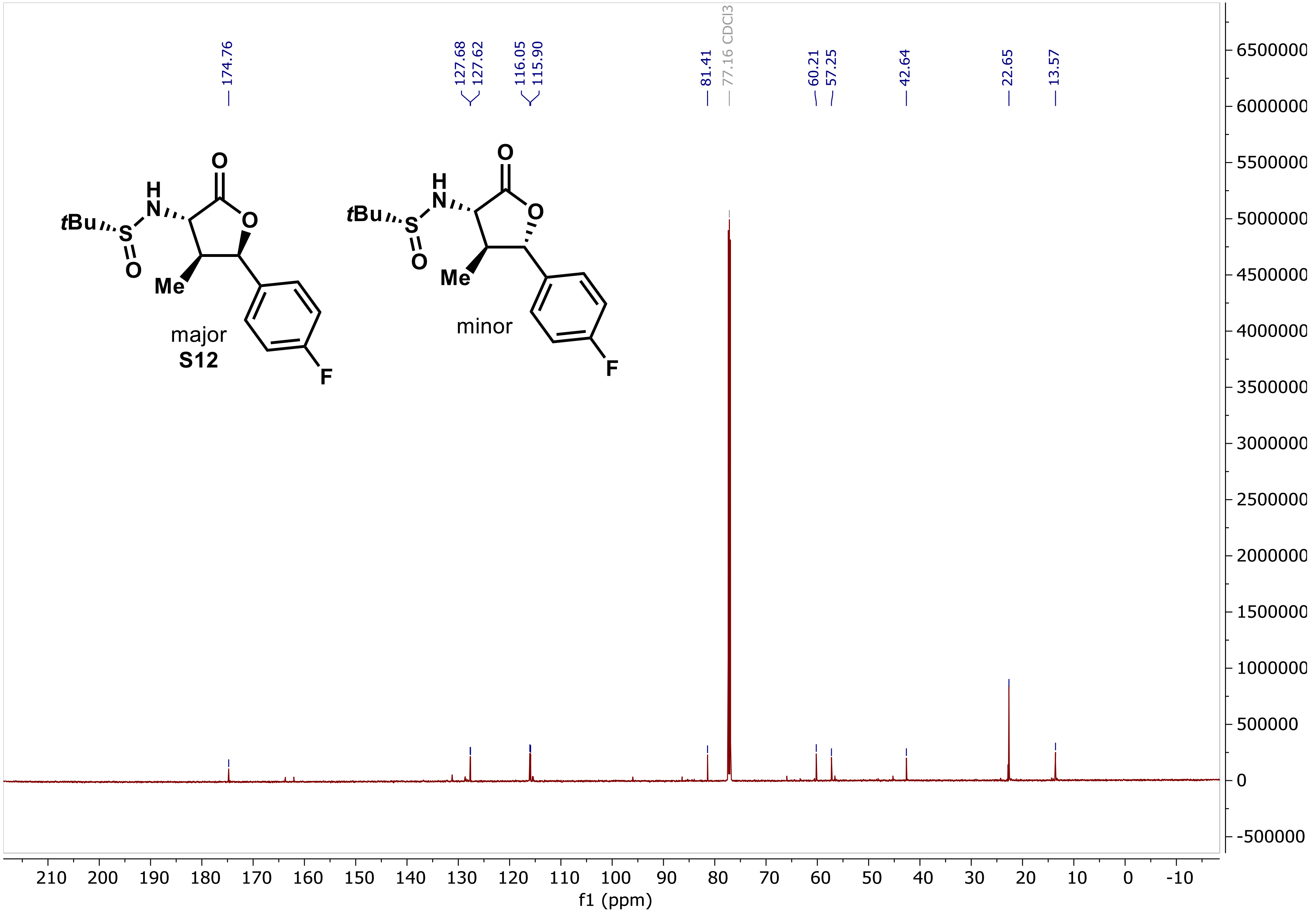

S12 ^{19}F-NMR ($CDCl_3$, 563 MHz)

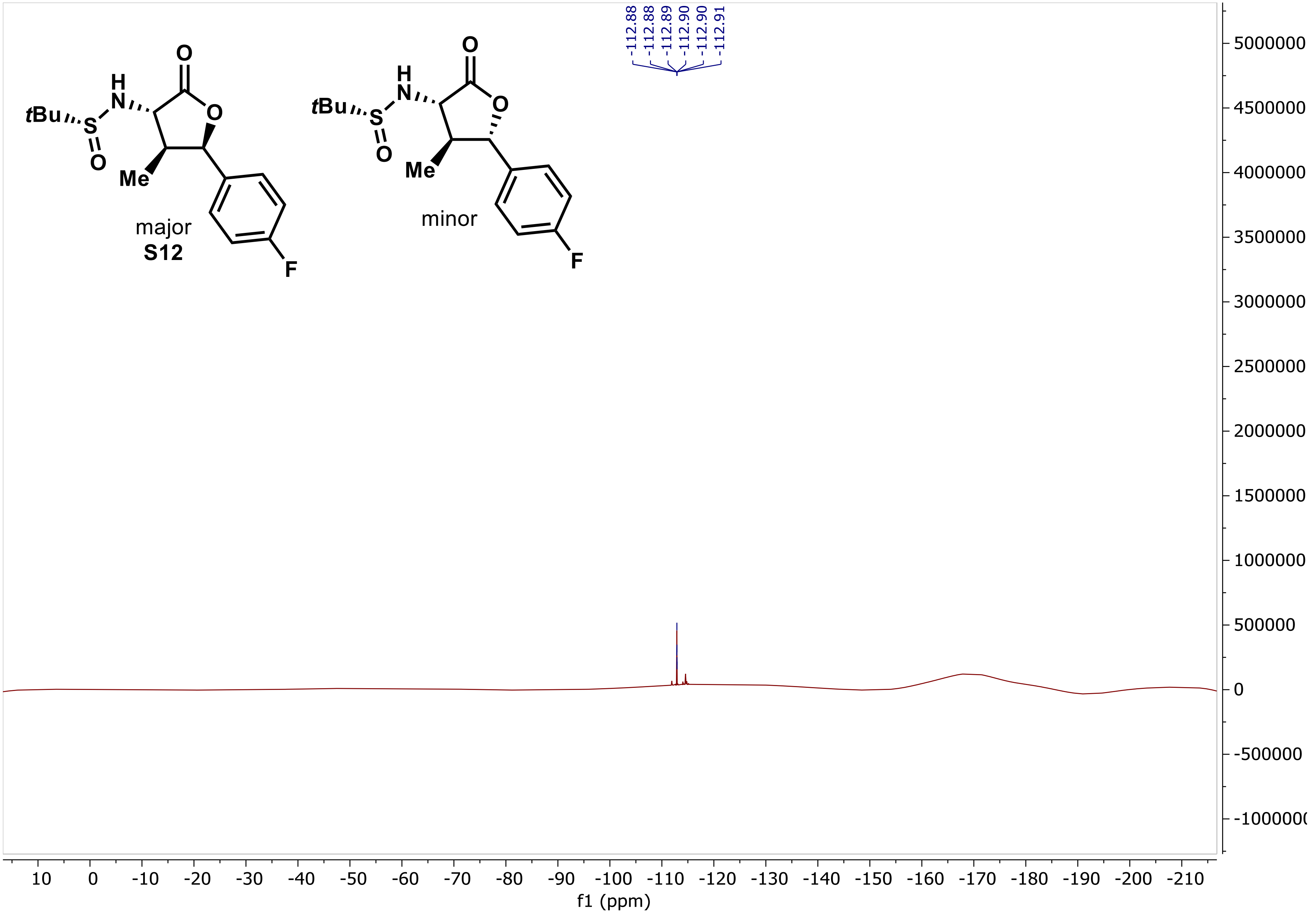

S12 NOESY-NMR ($CDCl_3$, 599 MHz)

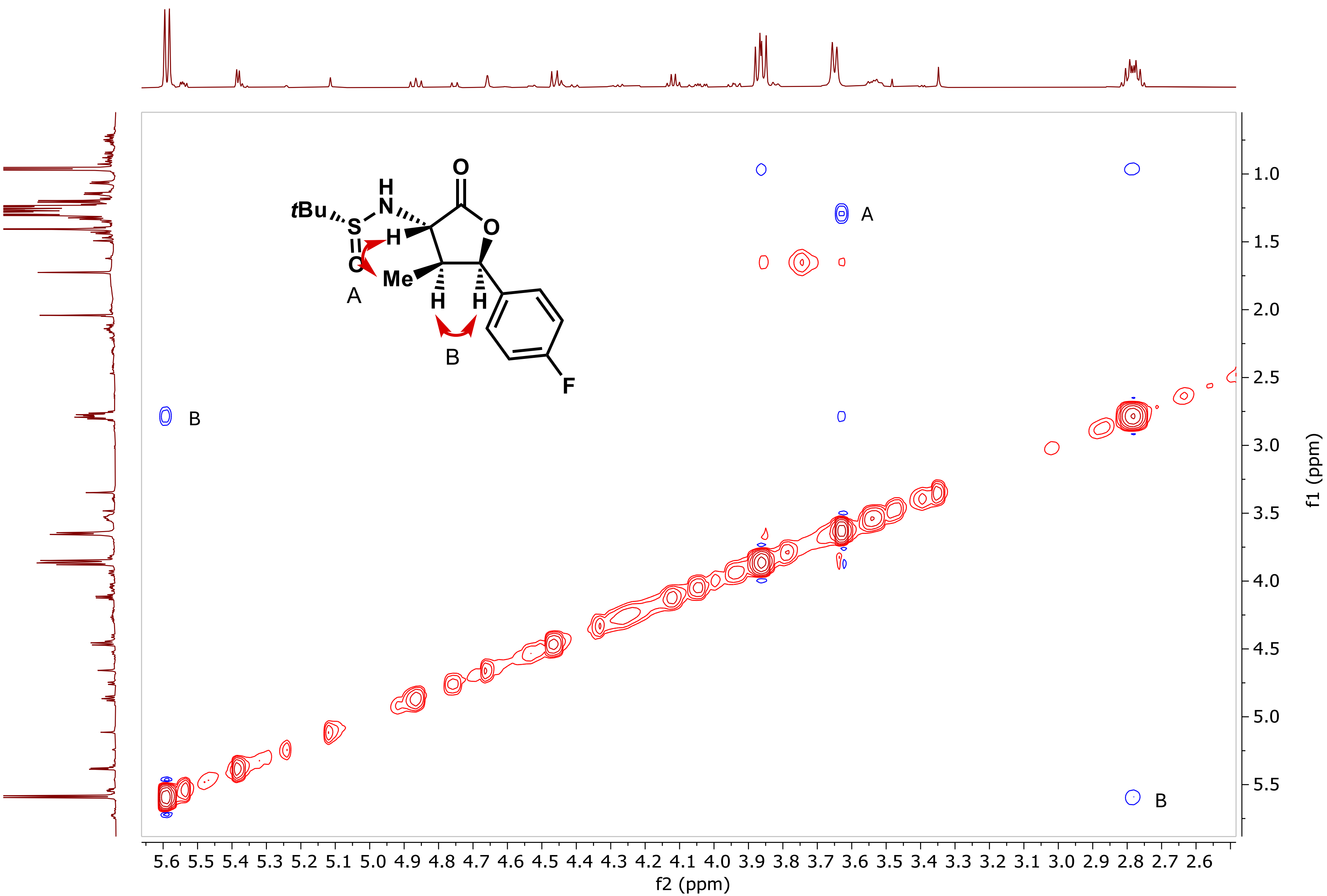

(2*S*,3*S*,4*R*)-14 ^{1}H-NMR (D_2O/LiOH, 599 MHz)

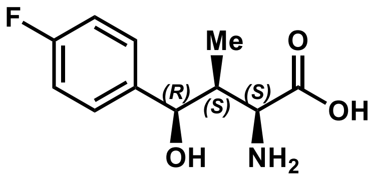

(2*S*,3*S*,4*R*)-**14**

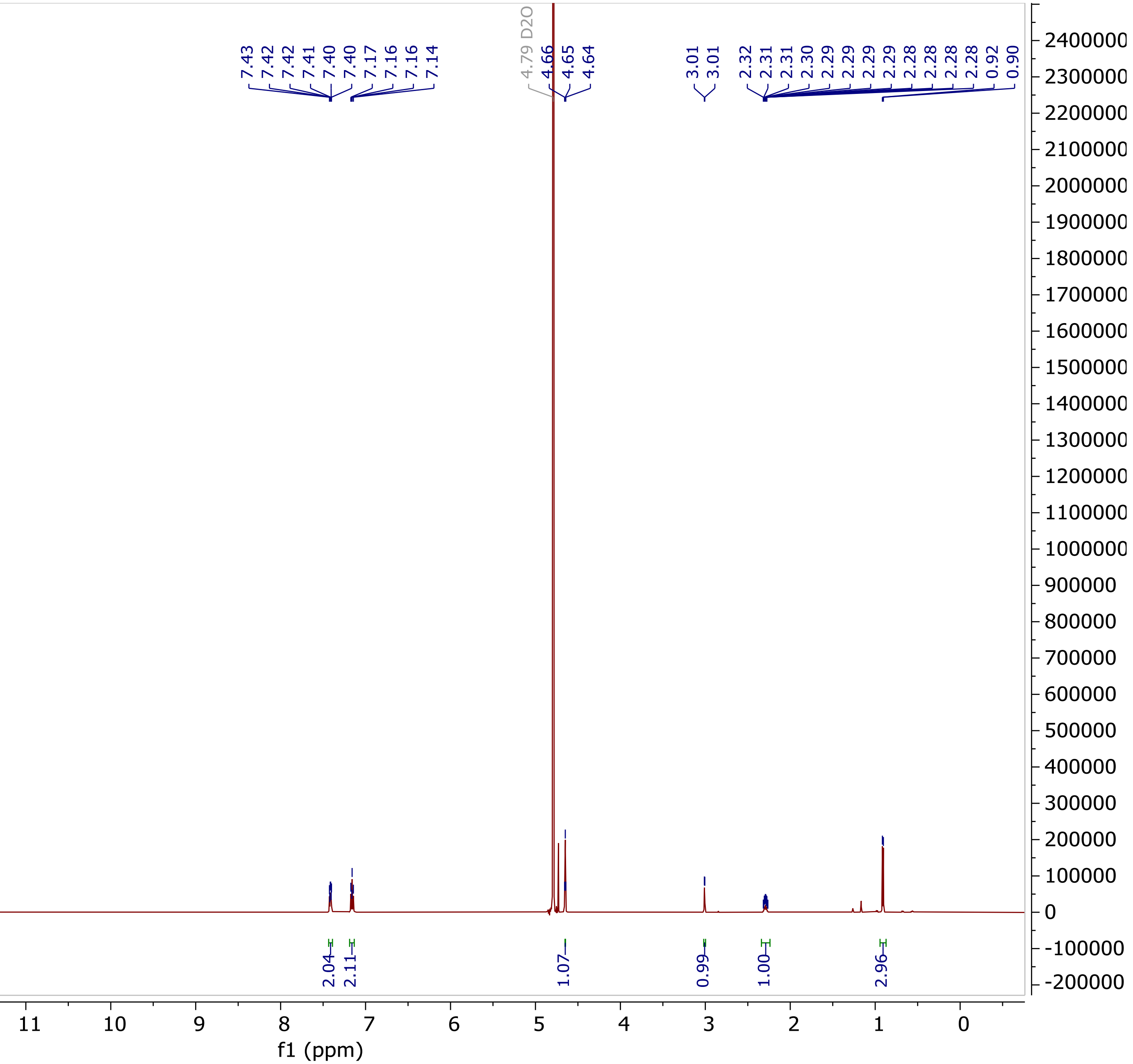

(2*S*,3*S*,4*R*)-14 ^{13}C-NMR (D_2O, 151 MHz)

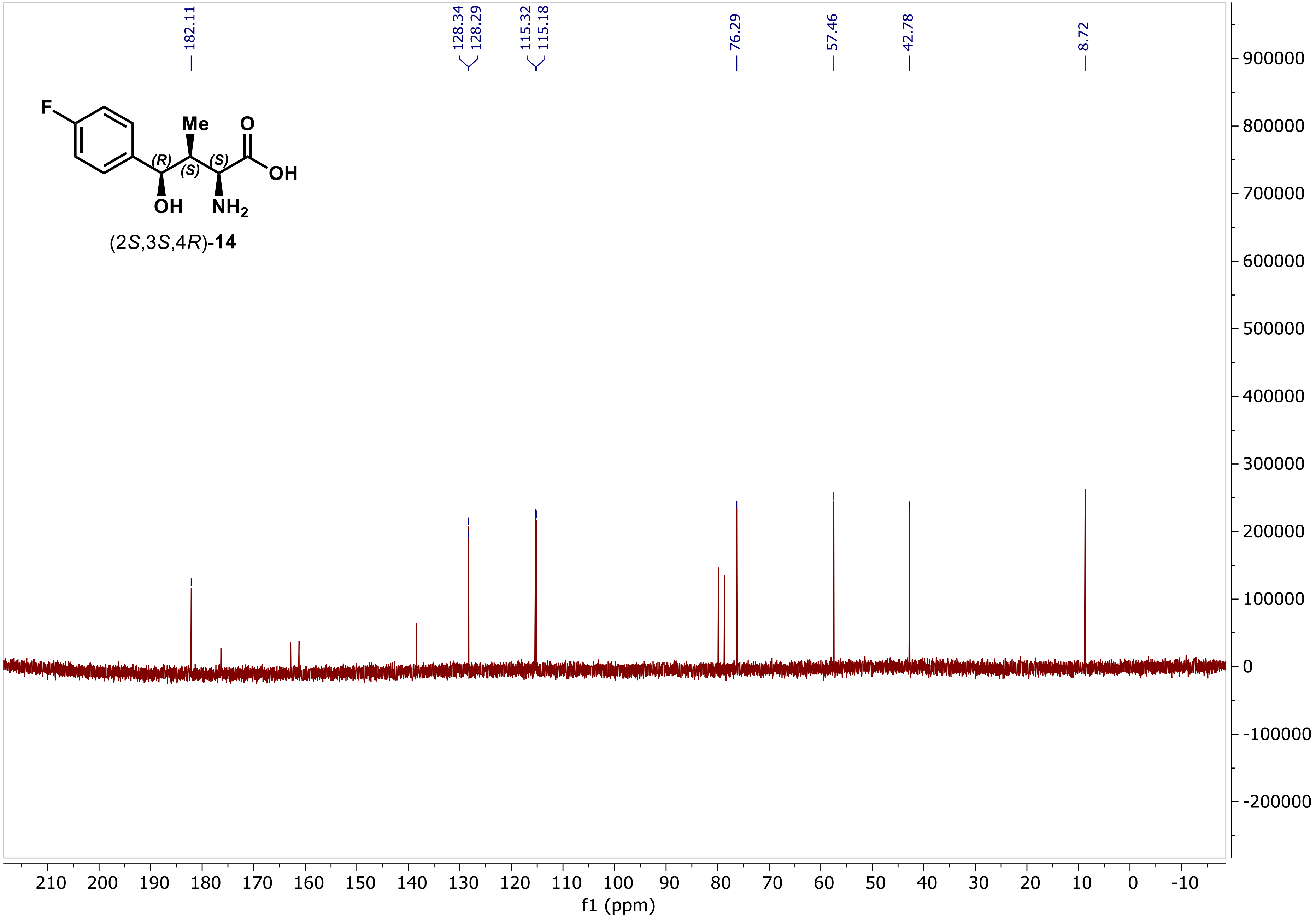

(2*S*,3*S*,4*R*)-14 ^{19}F-NMR (D_2O/LiOH/FCH_2CN, 563 MHz)

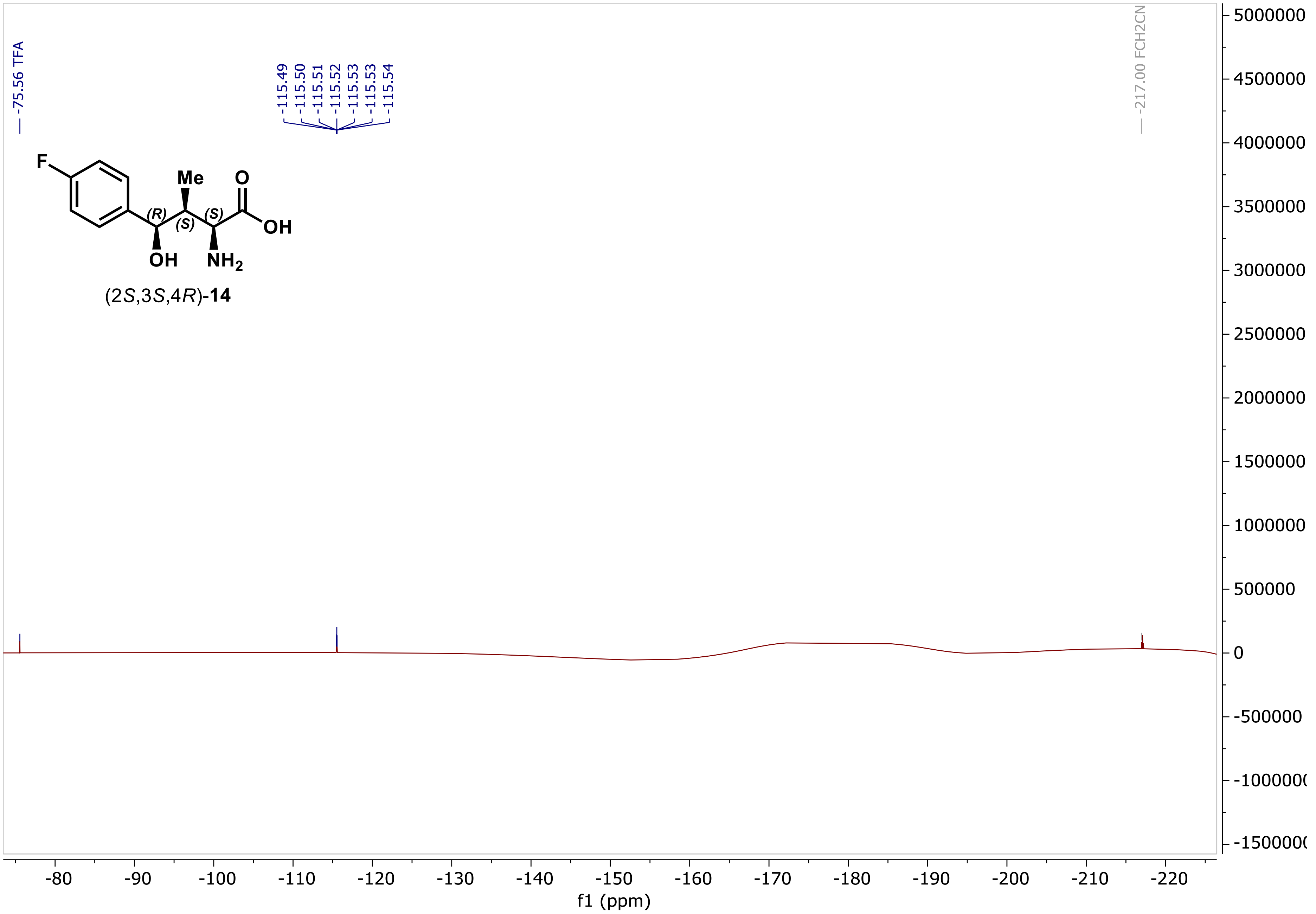

12 ^{1}H-NMR (D_2O/LiOH, 564 MHz)

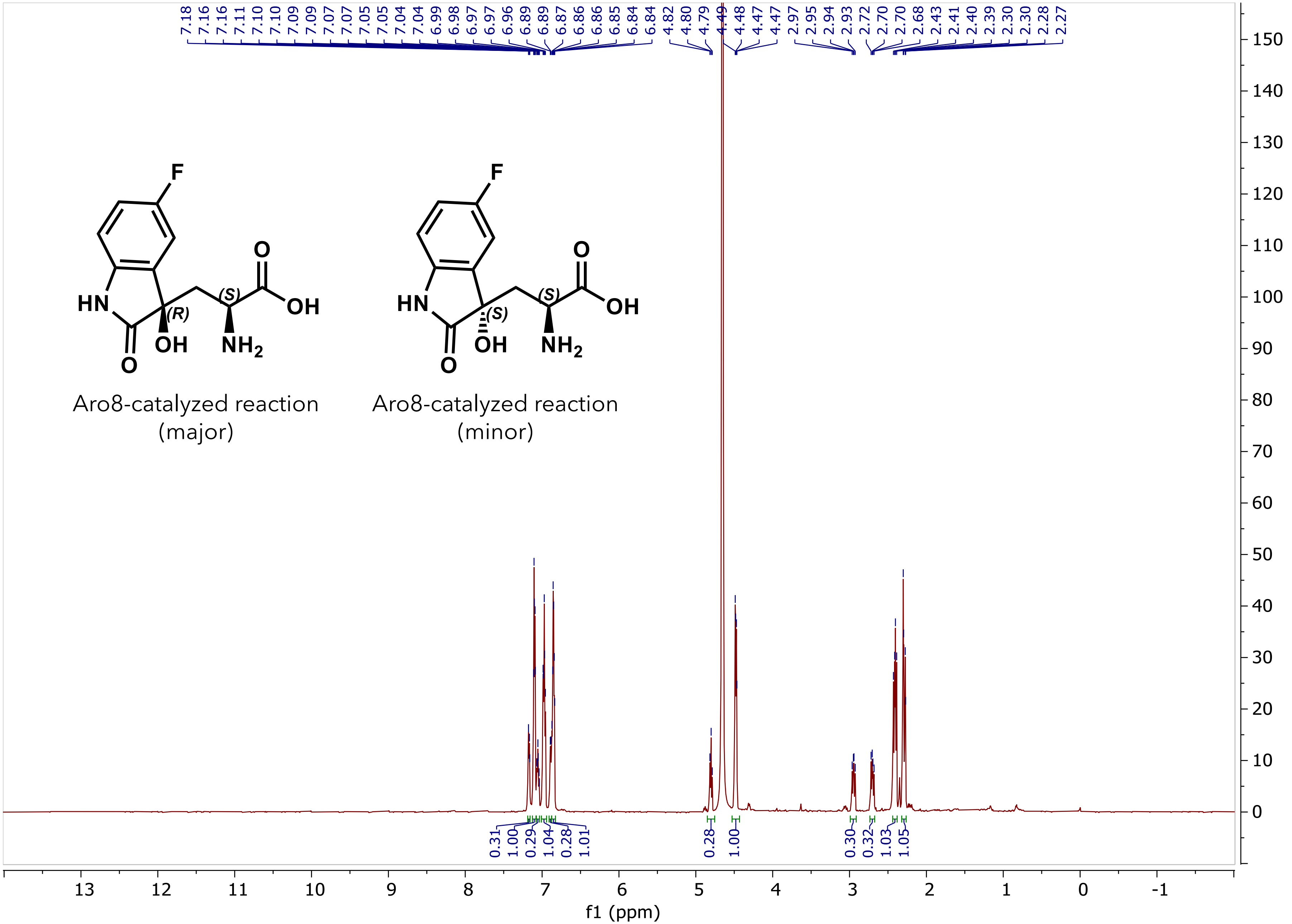

12 ^{13}C-NMR (D_2O/LiOH, 151 MHz)

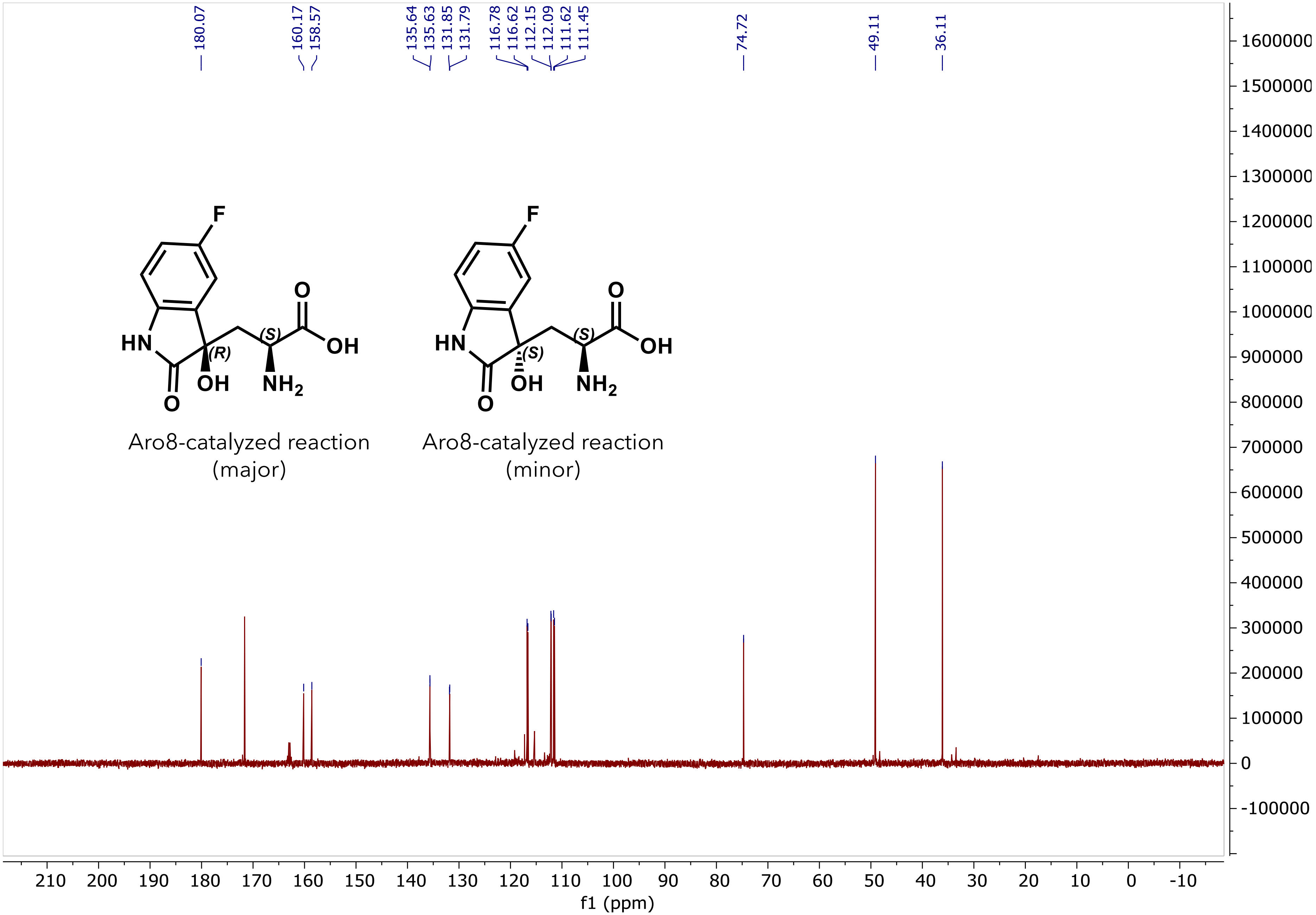

12 ^{19}F-NMR (D_2O/LiOH, 564 MHz)

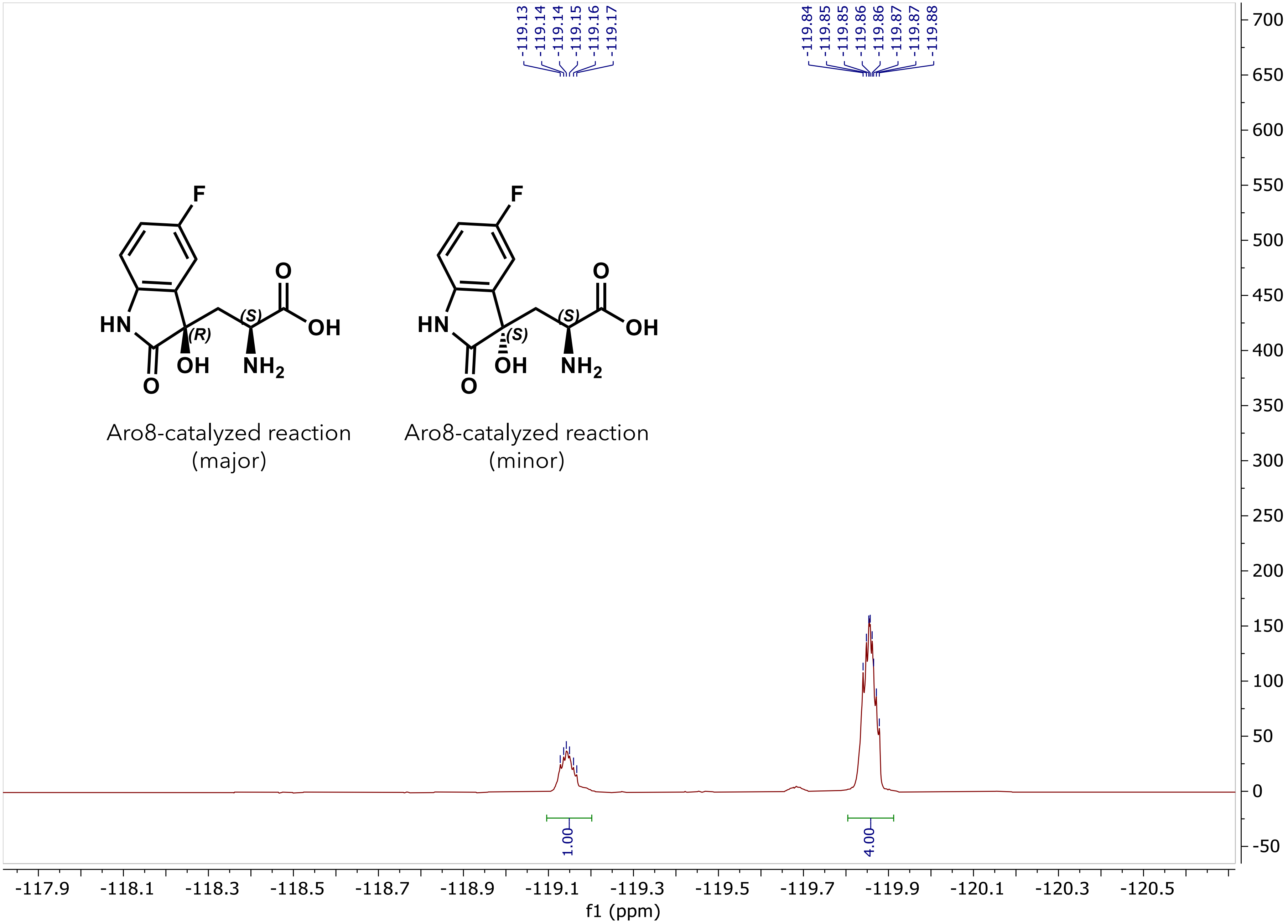

12 ^{1}H-NMR (D_2O/LiOH, 564 MHz)

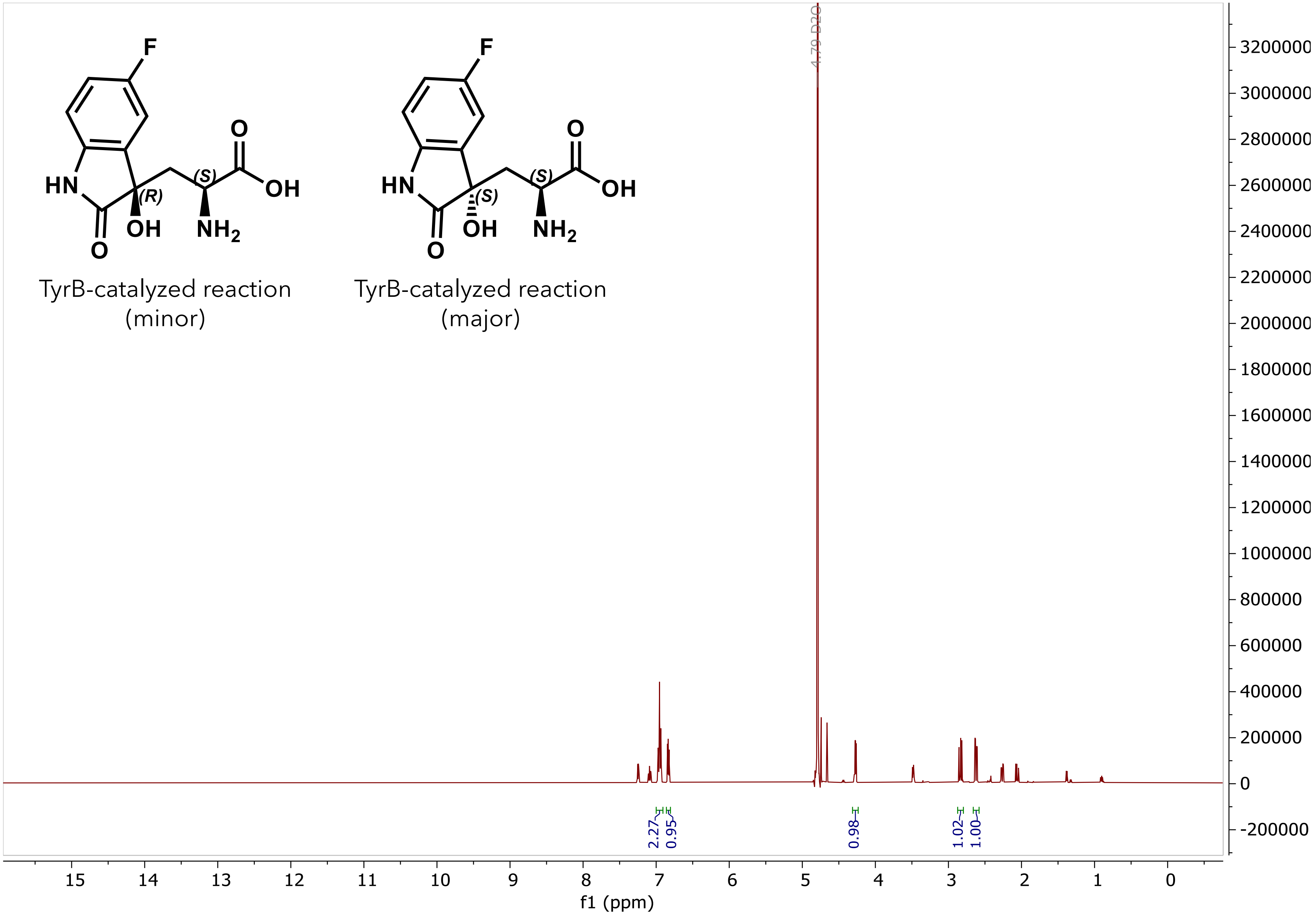

12 ^{13}C-NMR (D_2O/LiOH, 151 MHz)

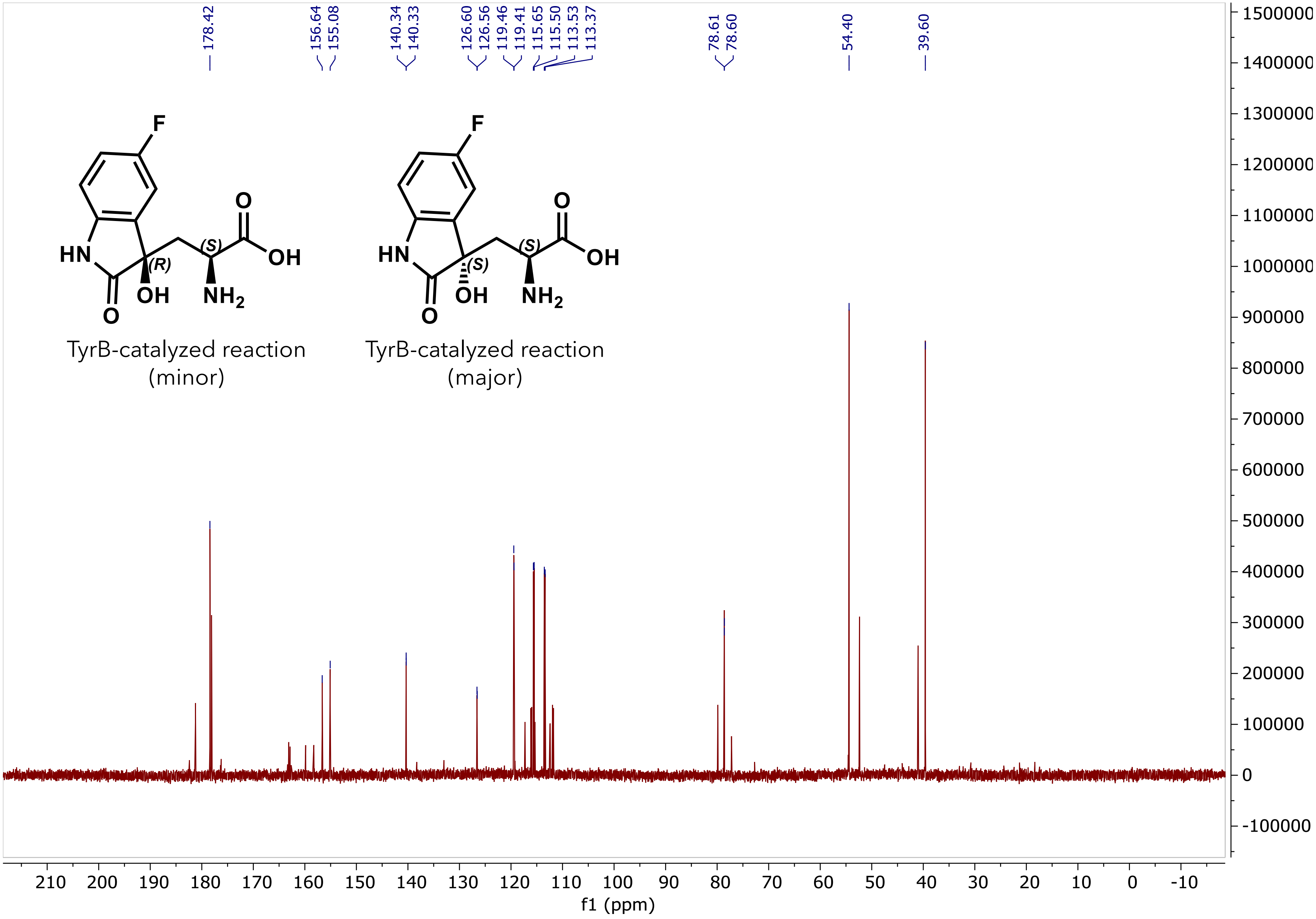

13 ^{1}H-NMR (D_2O/LiOH, 600 MHz)

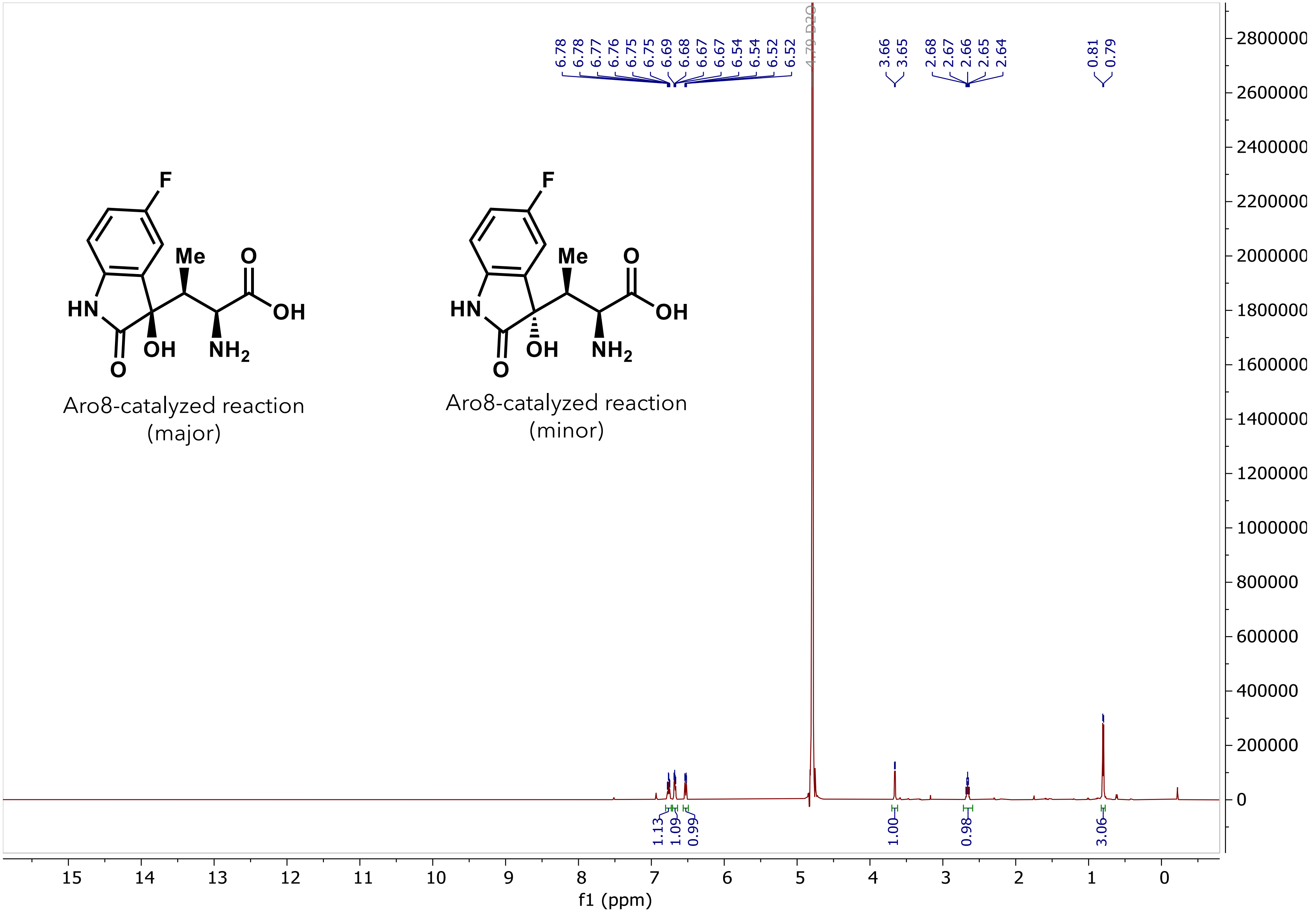

13 ^{13}C-NMR (D_2O/LiOH, 151 MHz)

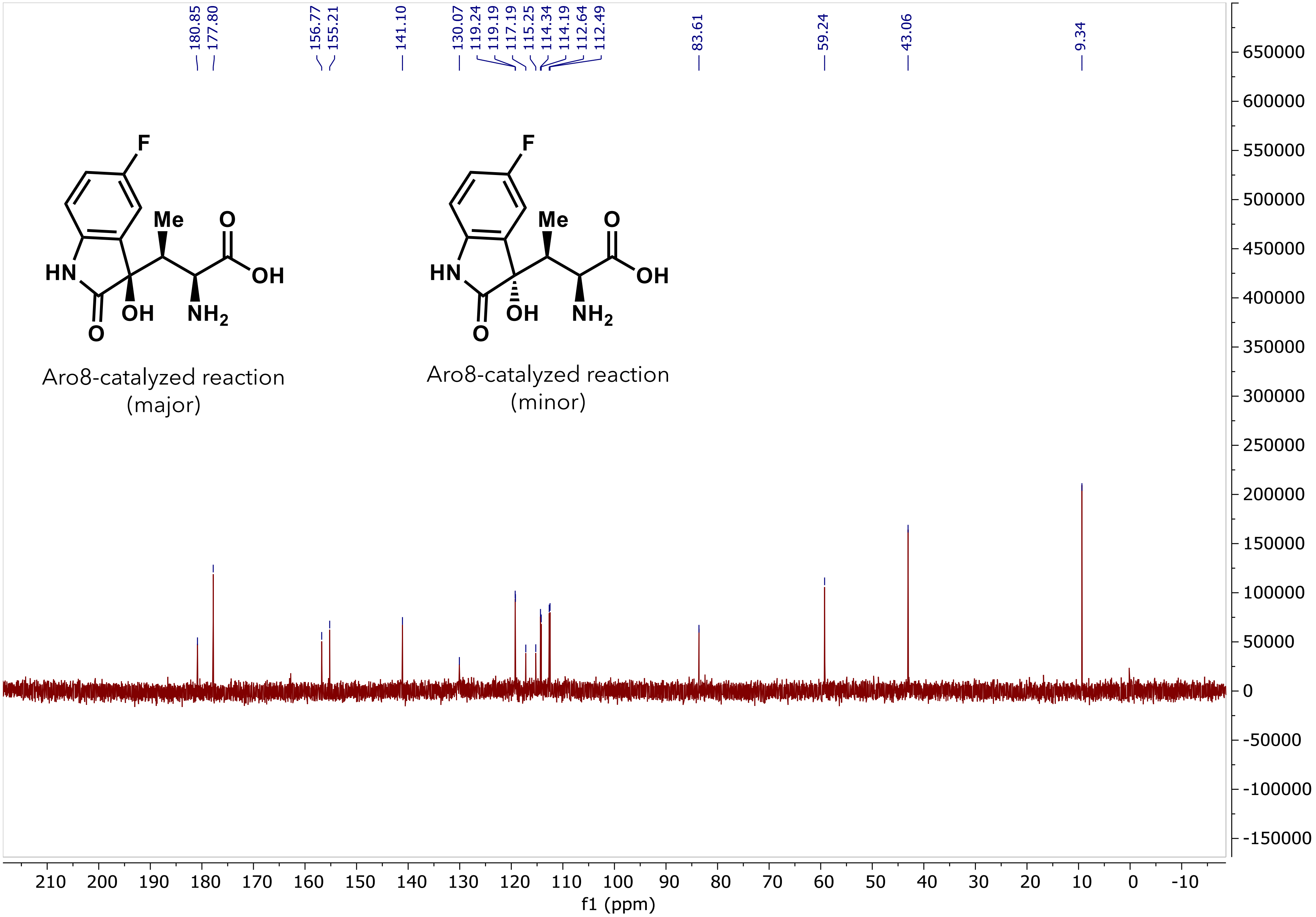

13 ^{19}F-NMR (D_2O/LiOH, 563 MHz)

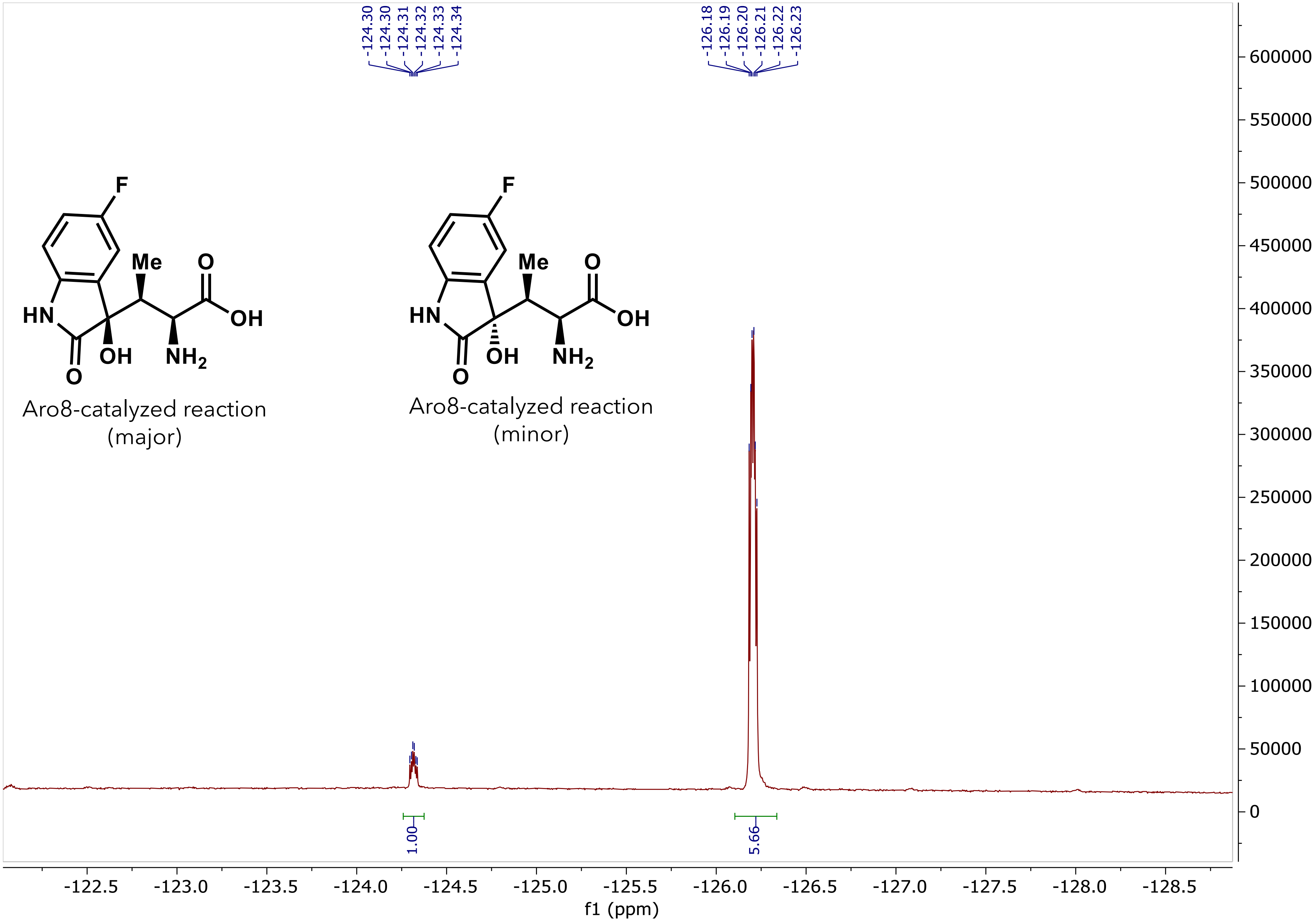

22 ^{19}F-NMR (CD_3CN, 563 MHz)

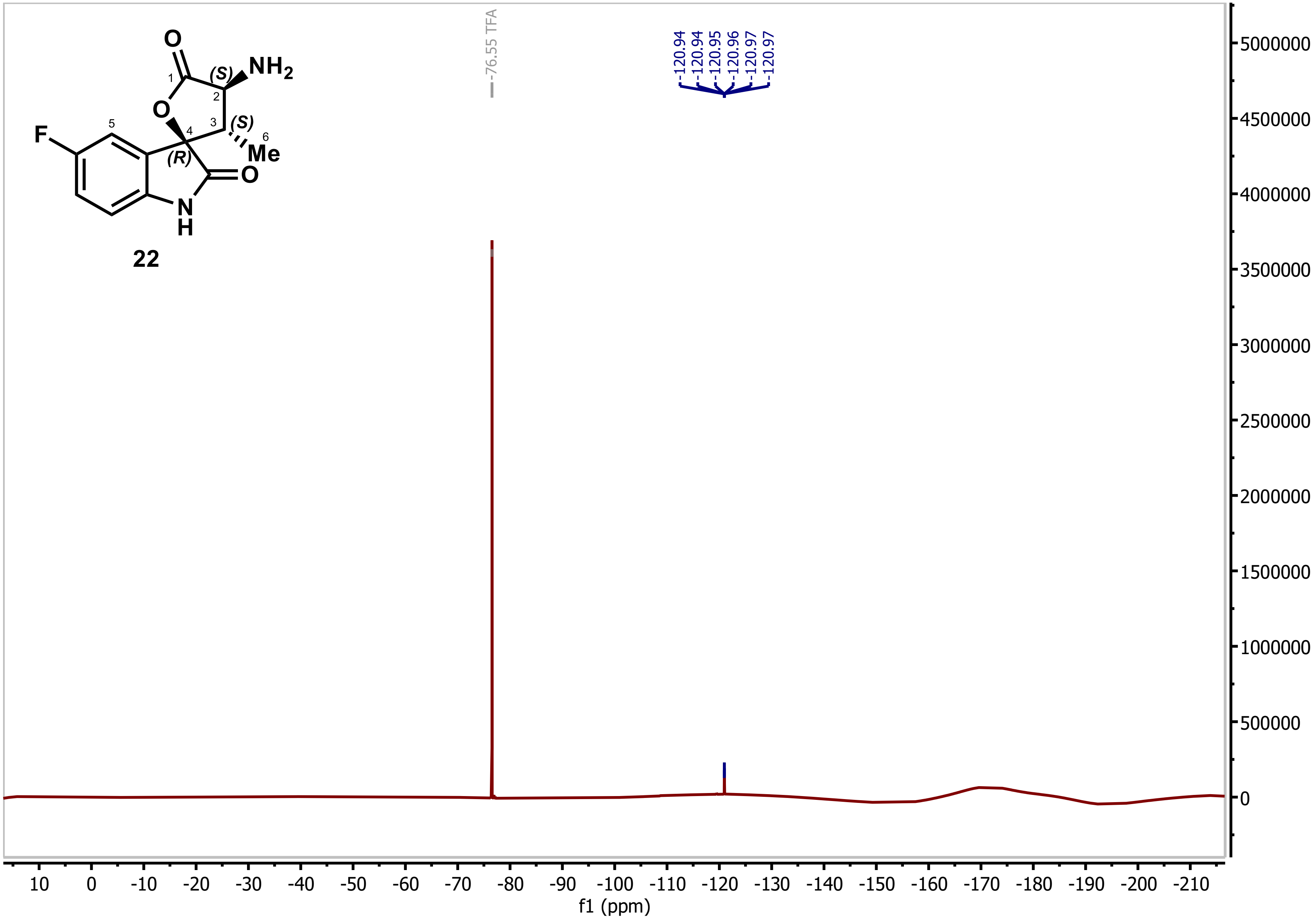

22 ^{1}H-NMR (CD_3CN, 600 MHz)

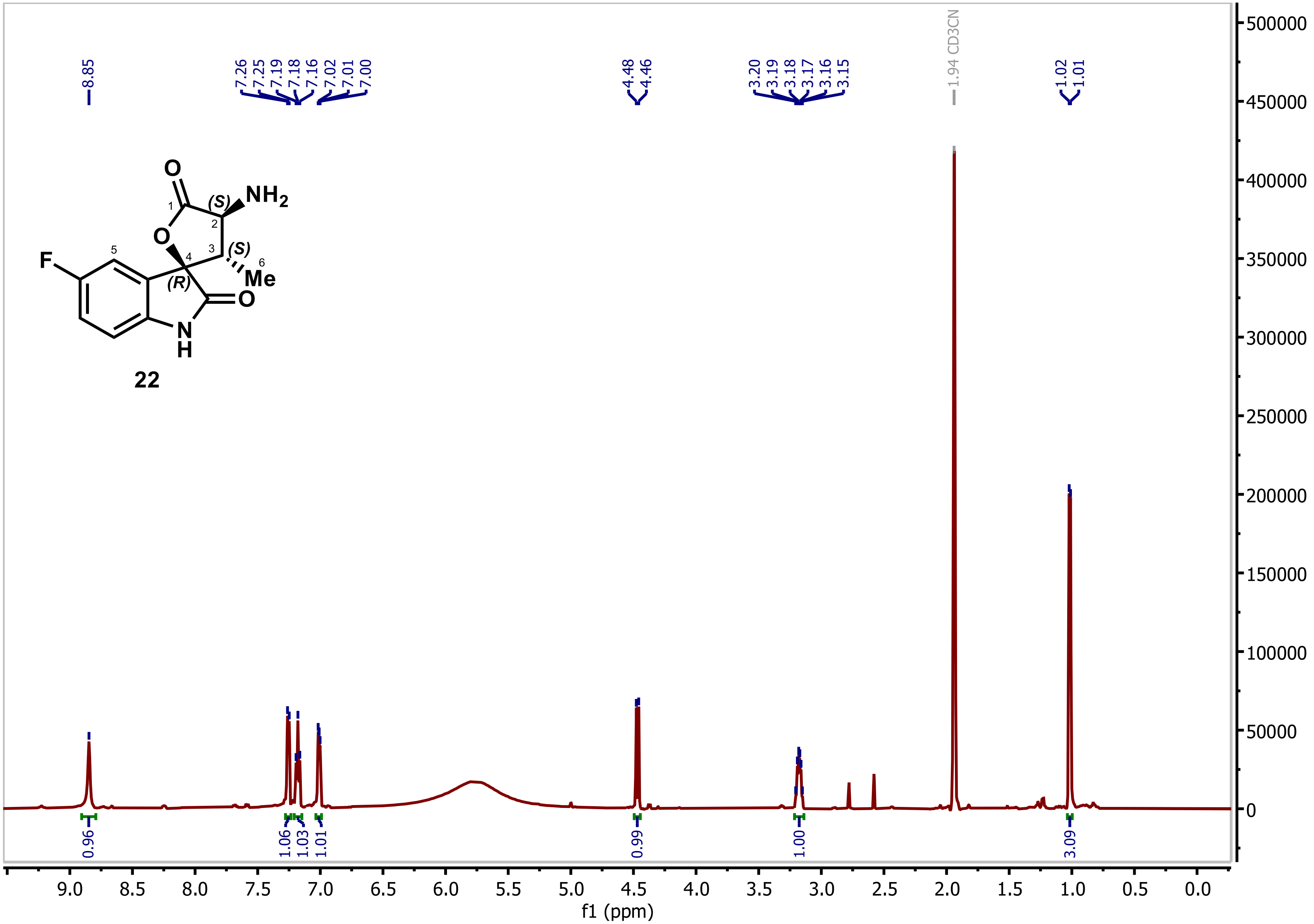

22 ^{13}C-NMR (CD_3CN, 151 MHz)

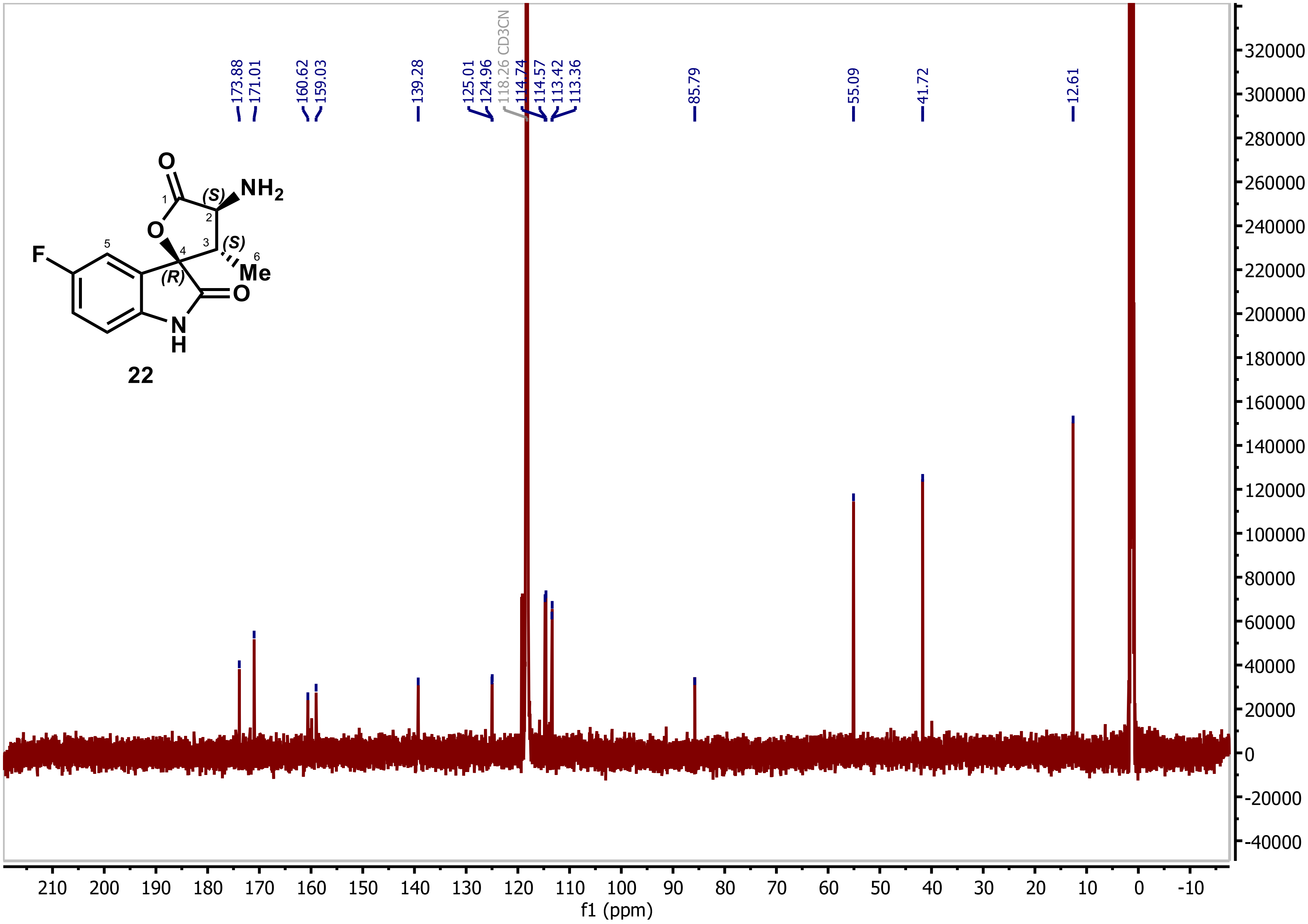

22 1D-NOESY (CD_3CN, 600 MHz)

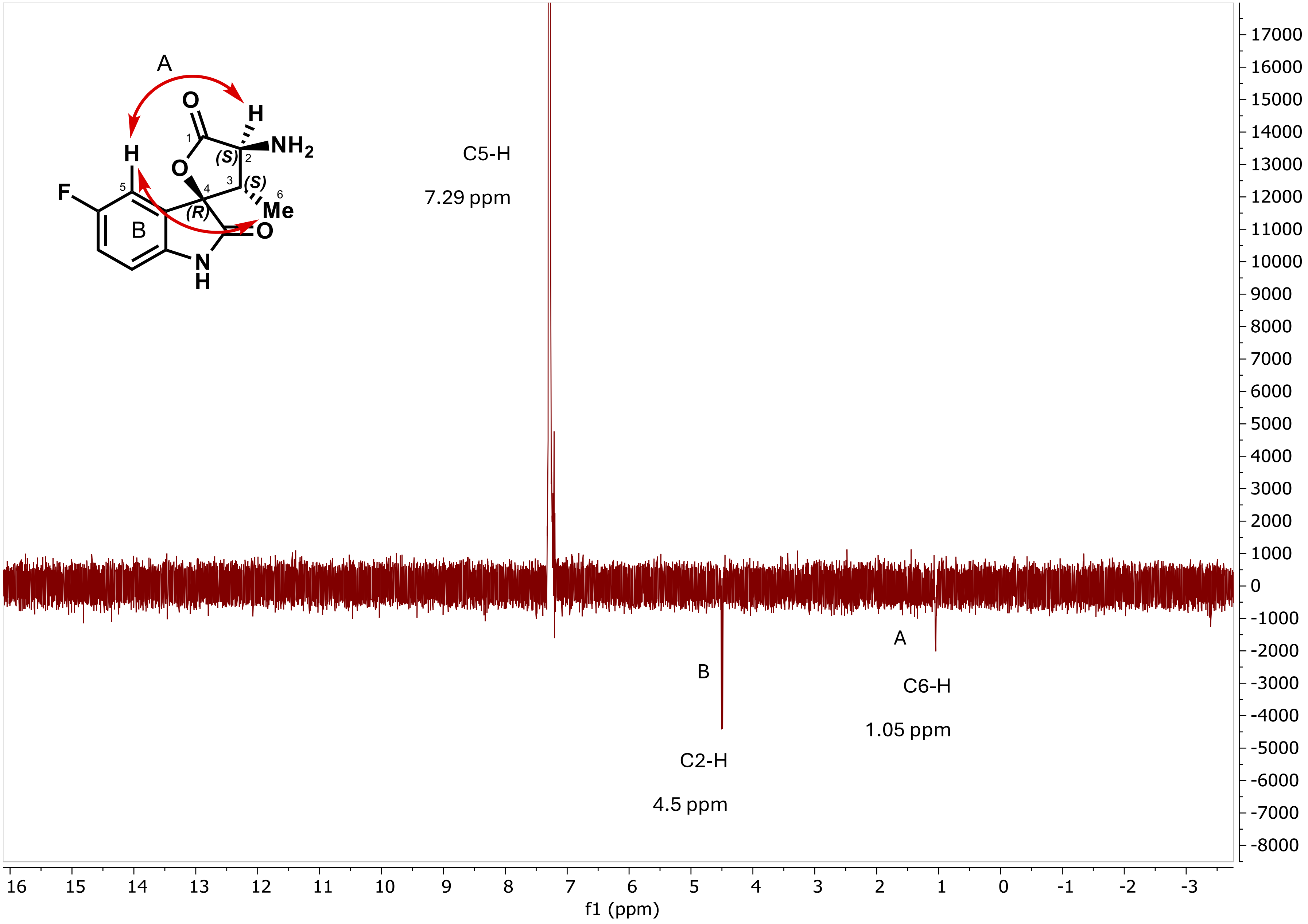

22 1D-NOESY (CD_3CN, 600 MHz)

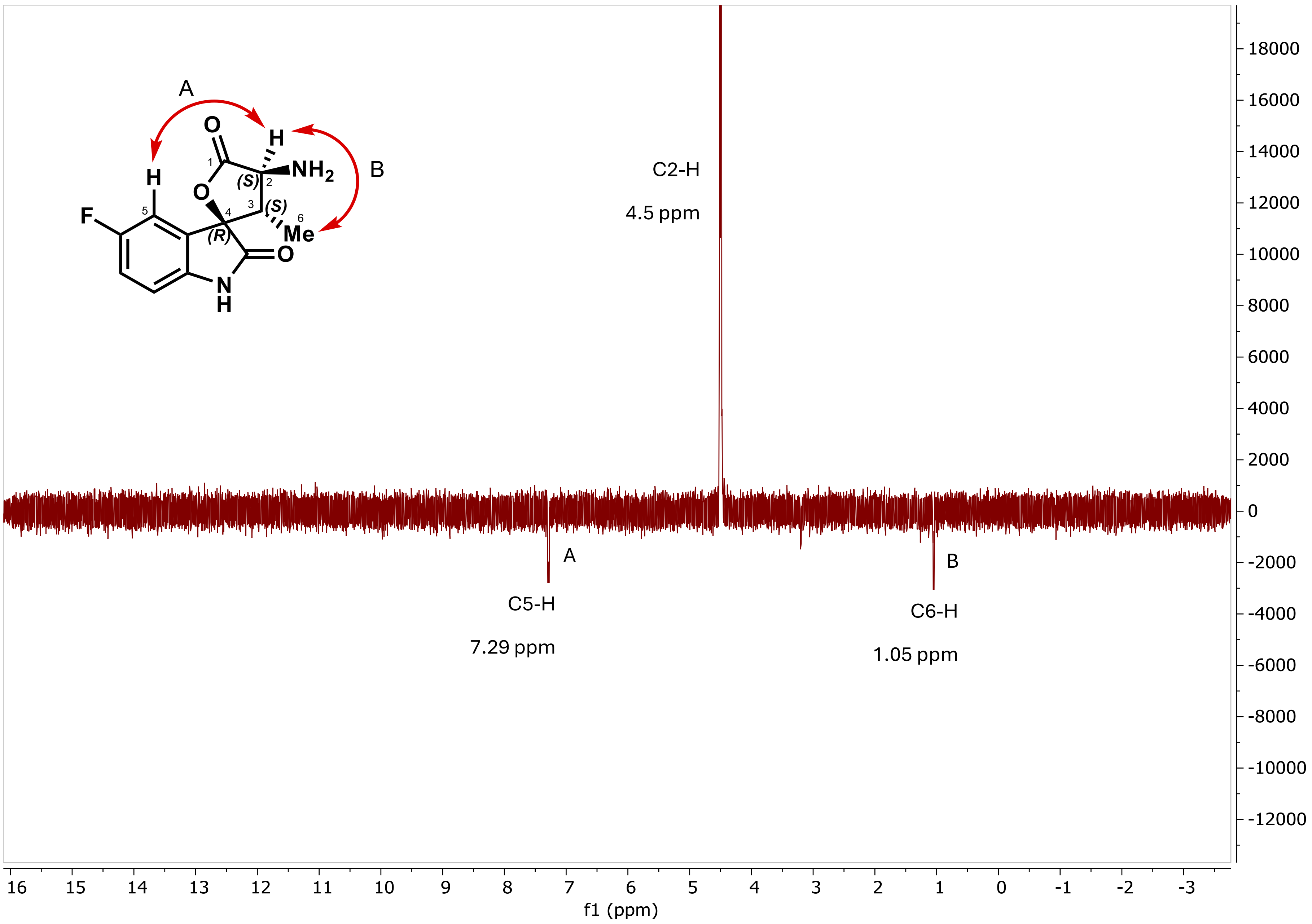

22 1D-NOESY (CD_3CN, 600 MHz)

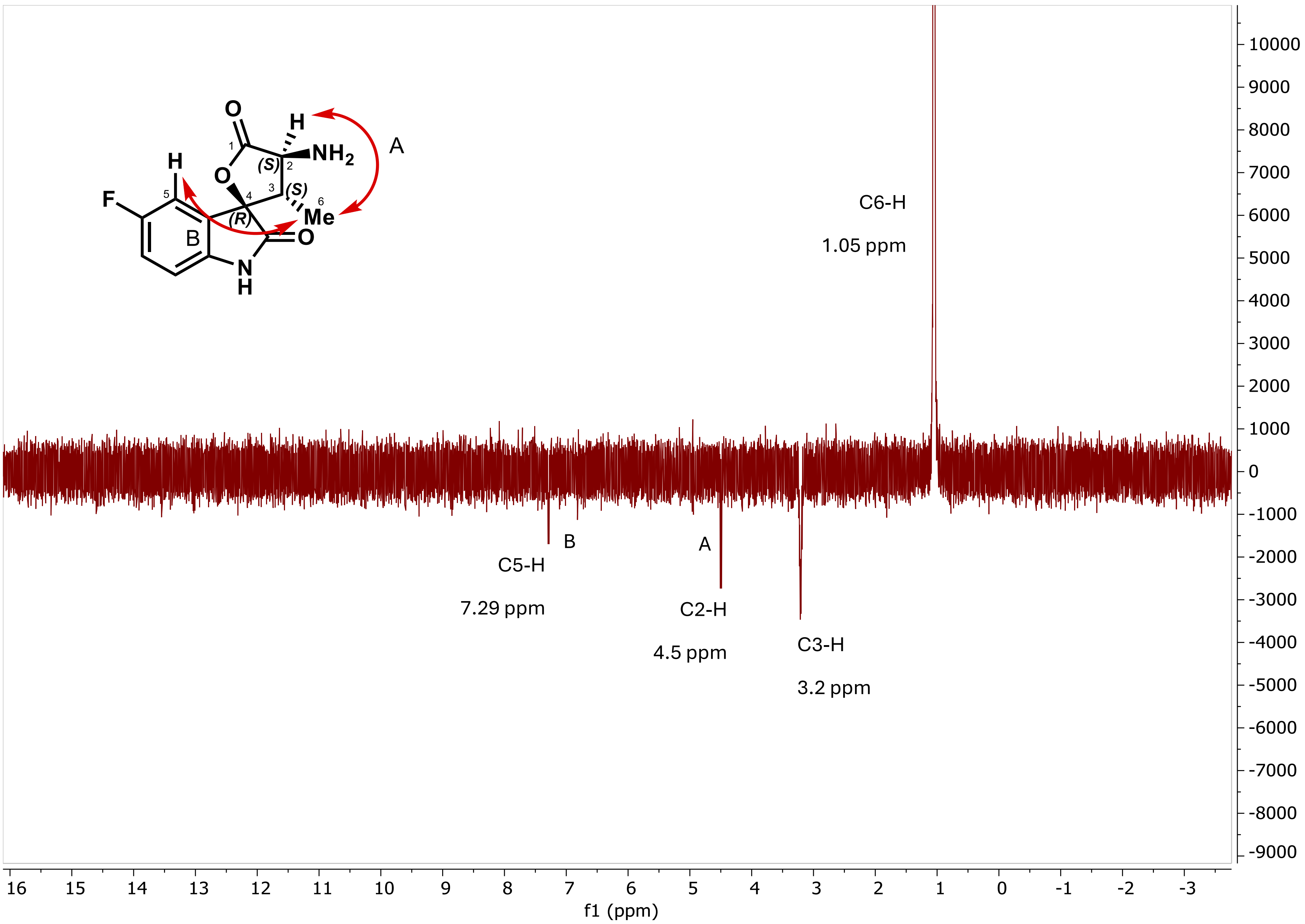

Supplement: Supplementary file 1 [file oc6c00531_si_001.pdf]
